# Interplay of secondary and tertiary folding in abiotic foldamers†
†Electronic supplementary information (ESI) available: Synthetic methods, characterization of new compounds, detailed of the crystallographic and NMR investigations, and of molecular dynamics simulations. CCDC 1901969–1901971. For ESI and crystallographic data in CIF or other electronic format see DOI: 10.1039/c9sc01322a


**DOI:** 10.1039/c9sc01322a

**Published:** 2019-06-10

**Authors:** Daniela Mazzier, Soumen De, Barbara Wicher, Victor Maurizot, Ivan Huc

**Affiliations:** a Department of Pharmacy , Centre for Integrated Protein Science , Ludwig-Maximilians-Universität , Butenandtstraße 5-13 , D-81377 Munich , Germany . Email: ivan.huc@cup.lmu.de; b CBMN Laboratory , Université de Bordeaux , CNRS , IPB , Institut Européen de Chimie et Biologie , 2 rue Escarpit , 33600 Pessac , France; c Department of Chemical Technology of Drugs , Poznan University of Medical Sciences , Grunwaldzka 6 , 60-780 Poznan , Poland

## Abstract

The first true abiotic tertiary folded structures, *i.e.* at the exclusion of any aliphatic amino acid, have recently been introduced under the form of aromatic oligoamide helix–turn–helix foldamers stabilized by hydrogen bonds in organic solvents. We present an investigation of the interplay of secondary and tertiary folding and of some cooperative effects in these systems. A solid phase synthesis approach to the preparation of these sequences was developed to facilitate systematic variation. Flexible pyridine-based units were introduced in various proportions in replacement of more rigid quinoline-based units. Conformational behaviour was assessed in solution by NMR, in the solid state by X-ray crystallography, and computationally through molecular dynamics simulations. Altogether, our results demonstrate that tertiary folding stabilizes otherwise flexible secondary structures, and that the disruption of tertiary folds upon adding polar solvents follows different mechanisms depending on whether secondary structures are inherently stable or not. These findings constitute a solid basis on which to further increase the size and complexity of abiotic folded structures and to eventually orchestrate folding dynamics and responsiveness.

## Abstract 2

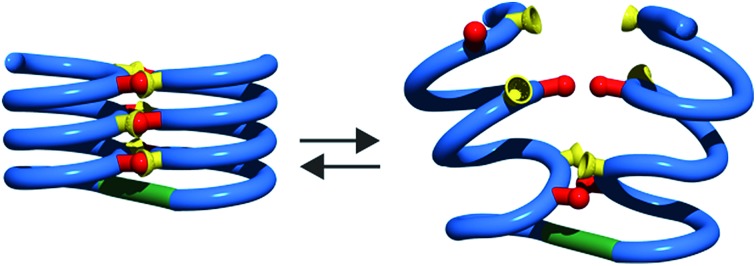
Helical secondary folds are stabilized within abiotic helix–turn–helix tertiary structures in organic solvents.

## Introduction

Foldamer research is based on the premise that non-natural backbones will give access to folded structures and functions beyond the reach of biopolymers. Researchers in the field have been successful in eliciting folding of secondary motifs such as single helices or sheets in a great variety of synthetic oligomers.^1^–^4^ However, the *ab initio* design of tertiary structures is found to be far more complex and constitutes a major challenge ahead. Motivation to meet this challenge comes from the fact that biopolymer functions emerge at the tertiary structure level. In comparison to a full length protein, a simple α-helix cannot achieve much. Tertiary folds may thus be the level at which foldamers will reveal their full potential.

Current progress in protein design^5^–^10^ allows one to be optimistic about its extension to foldamers. Some important milestones have indeed been achieved. For example, α-peptidic tertiary folds and α-helix bundles have been shown to tolerate several β-amino-acids.^11^–^16^ Furthermore, helix bundles comprised of β-amino-acids^17^,^18^ or β-ureas,^19^ at the exclusion of any α-amino acid have also been described. In contrast, until recently, progress in the area of abiotic backbones was less advanced and limited to a few reports describing structures produced by connecting several secondary elements without well-defined interactions over extended surfaces between them.^20^–^24^ Yet, there are good reasons to invest efforts in abiotic foldamer development. Because their chemical nature is by definition remote from that of biopolymers, the structures that can be obtained and by extension their properties and functions are also expected to be distinct. In this respect, aromatic foldamers,^25^–^27^ *i.e.* foldamers with aryl groups in their main chain, represent a promising and rapidly developing family of abiotic backbones. Illustrations of their distinctiveness with respect to biopolymers include their strong folding propensity in all kinds of media other than water and the production of novel shapes such as helices with a reduced diameter at the end of the sequence and a wider diameter in the centre of the sequence that eventually give rise to cavities suitable for molecular recognition.^28^–^31^

**Fig. 1:**
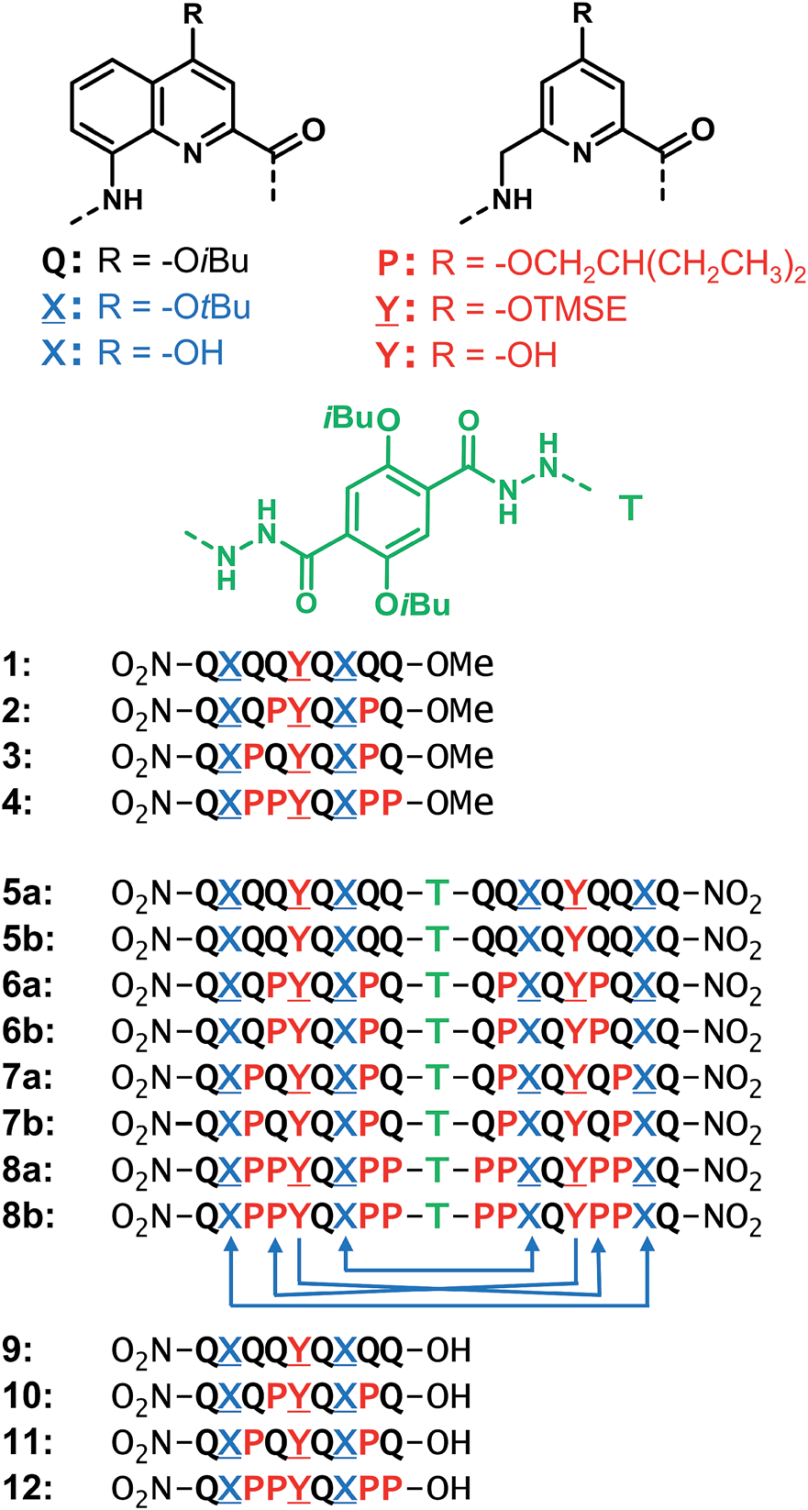
Structures of units **Q**, **X**, **P**, **Y**, **T** and foldamer sequences. **X[combining low line]** and **Y[combining low line]** are the protected precursors of hydrogen bonding units **X** and **Y**. All flexible units **P**, **Y[combining low line]**, or **Y** are shown in red. Sequences are labelled ‘**a**’ when protected and ‘**b**’ when deprotected. Sequences end with 8-nitro groups; this group is noted in the replacement of the NH group at terminal **Q** units. The **T** unit constitutes an inversion of C → N sequence polarity; sequences that contain **T** thus have two N termini. The arrows below **8b** indicate the hydrogen bonds pattern between the helices. TMSE = 2-trimethylsilylethyl.

We recently reported the *ab initio* computational design, solution phase synthesis and structural characterization of the first true abiotic tertiary structures.^32^ We used the well-characterized helically folded oligoamides of 8-amino-2-quinolinecarboxylic acid **Q** (Fig. 1) as secondary modules.^33^,^34^ Upon introducing monomers **X** and **Y** at specific positions in a sequence, the aryl-OH groups borne by these monomers were shown to promote the formation of well-defined inter-helix arrays of hydrogen-bonds. When two helices were combined with a turn unit **T** (Fig. 1), we observed that sequences such as **5b** fold into a helix–turn–helix structure with high stability in non-polar organic solvents. Remarkably, tertiary folding proved to follow an all-or-nothing mechanism: the progressive addition of a polar solvent that competes for hydrogen bonding caused a sharp transition towards a non-hydrogen-bonded helix–turn–helix structure.

Cooperativity lies at the core of protein functions in that it allows for key dynamics and structural changes to take place. Controlling tertiary structure dynamics through cooperative effects may thus bring critical advantages to implement function. We thus endeavoured to investigate further this aspect in an abiotic context. In the following, we report that abiotic tertiary folds based on aromatic oligoamides tolerate a great deal of destabilization of their secondary helical modules. Specifically, we first developed a new synthetic approach based on solid phase synthesis and post-synthetic fragment assembly that allows for systematic variations of the sequences. Upon introducing an increasing number of flexible units in the helices that reduce inherent conformational stability, we show that the tertiary structures still fold and promote helicity in the secondary units. We also find that the destabilizing effect of polar solvents leads to different unfolding pathways depending on the number of flexible units. In addition, computations show that thermal denaturation of the tertiary structures is expected to occur, but at temperatures higher than what can be reasonably reached in experiments. Altogether, these results validate a rational approach to design tertiary structure dynamics, starting from rigid modules and progressively adding degrees of freedom to the system.

## Results and discussion

### Foldamer sequence design and synthesis

For the design of the helix–turn–helix conformation of **5b**, an aliphatic amine unit, 6-aminomethyl-4-hydroxy-2-pyridinecarboxylic acid **Y**, was introduced as structural analogue of **X**.^32^**Y**, which lacks the benzenic ring of quinolines, is required to prevent steric clashes between the helices while it imparts the same strand curvature as **X**. In addition, its aliphatic methylene unit brings an additional rotatable bond that enhances flexibility. We have already shown that the introduction of several of these aliphatic amine monomers into aromatic oligoamides may promote non-canonical conformations,^35^ and enhance conformational dynamics. Although these aliphatic amine units possess an overall poor folding propensity, they adopt the canonical aromatic helix conformation in the presence of a sufficient number of aromatic units.^36^–^38^

**Fig. 2:**
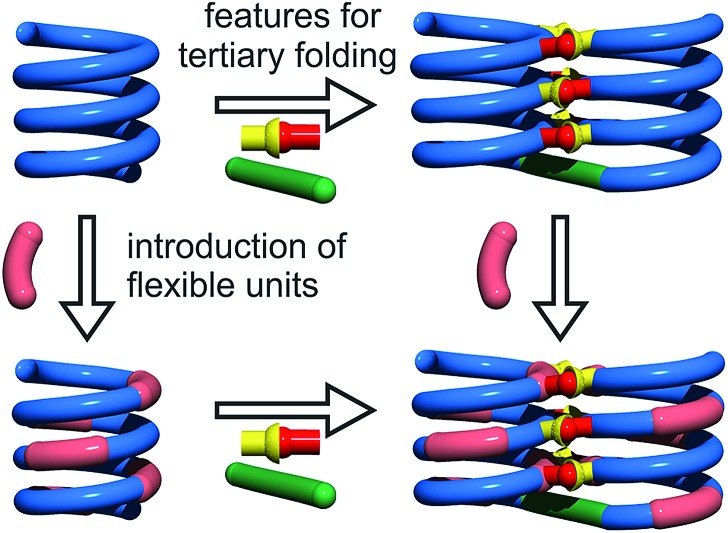
Double mutant cycle designed to assess cooperative effects in abiotic tertiary structures. The green rod and the red-and-yellow connector depict the turn unit and hydrogen bonding functions, respectively.

By analogy with **Y** which replaces **X**, we designed aliphatic unit **P** to replace **Q** (Fig. 1). We expected that this substitution would enhance flexibility, handedness dynamics, and folding entropy: **P** units should alter helix stability, yet this effect may be compensated when helices are held together in a tertiary motif. Thus, sequences **1–4** were designed so as to adopt single helical conformations having an increasing number – one, three or five – of aliphatic amine units (Fig. 1). Sequences **2** and **3** have the same number of **P** units and differ only by their position in the sequence. Sequences **2** and **4** contain adjacent aliphatic amine units, respectively **PY** or **PPY** and **PP**, but the other two sequences only possess non-adjacent **P** or **Y** monomers. In order to evaluate the effect of tertiary folding on the stability of the secondary helical modules sequences, deprotected **6b**, **7b** and **8b** were designed (Fig. 1) as potential helix–turn–helix analogues of **5b**. In principle, these compounds altogether allow one to build so-called double mutant cycles in order to assess cooperative effects (Fig. 2).^39^,^40^ If compared to reference compound **1**, a stable isolated helical secondary motif (side chains are all protected), compounds **2–4** are made more flexible through the introduction of **P** units, and compound **5b** has the features that promote tertiary folding (turn unit and hydroxyl groups). In compounds, **6b**, **7b** and **8b**, both flexible units and features to promote tertiary folding are present.

For their use in the solid phase synthesis (SPS) of the sequences, each new monomer (**X[combining low line]**, **P** and **Y[combining low line]**) was produced as an Fmoc-amine protected carboxylic acid. Their preparation is presented in detail in the ESI.† Monomers **X[combining low line]** and **Y[combining low line]** also possess a TFA labile protection of their 4-hydroxy group, *i.e. tert*-butyl ether and trimethylsilyl ethyl ether, respectively. **P** is functionalized with a 2-ethylbutan-1-ol solubilizing group. Fmoc-**Q**-OH has been described previously.^38^ Because the direct acid chloride activation of Fmoc-**Y[combining low line]**-OH and Fmoc-**P**-OH, as required for coupling onto aromatic amines, proved to be troublesome, Fmoc-**Y[combining low line]Q**-OH and Fmoc-**PQ**-OH dimers were pre-assembled and incorporated as such during SPS. The preparation of oligomers **9–12** was then carried out using previously reported SPS methods.^38^,^41^ The use of a SASRIN resin allowed for the cleavage from the resin while preserving side chain protection using hexafluoro-isopropanol (see ESI†). Acid terminated oligomers **9–12** were then converted into methyl esters **1–4**, respectively, under mild conditions using trimethylsilyl-diazomethane. Protected sequences **5a–8a**, were synthesized in solution by coupling foldamer acids **9–12** with the terephthalic acid bis-hydrazide turn unit (**T**). Finally, deprotected compounds **5b–8b** were obtained after TFA treatment of **5a–8a**, respectively.

### Secondary folding

**Fig. 3:**
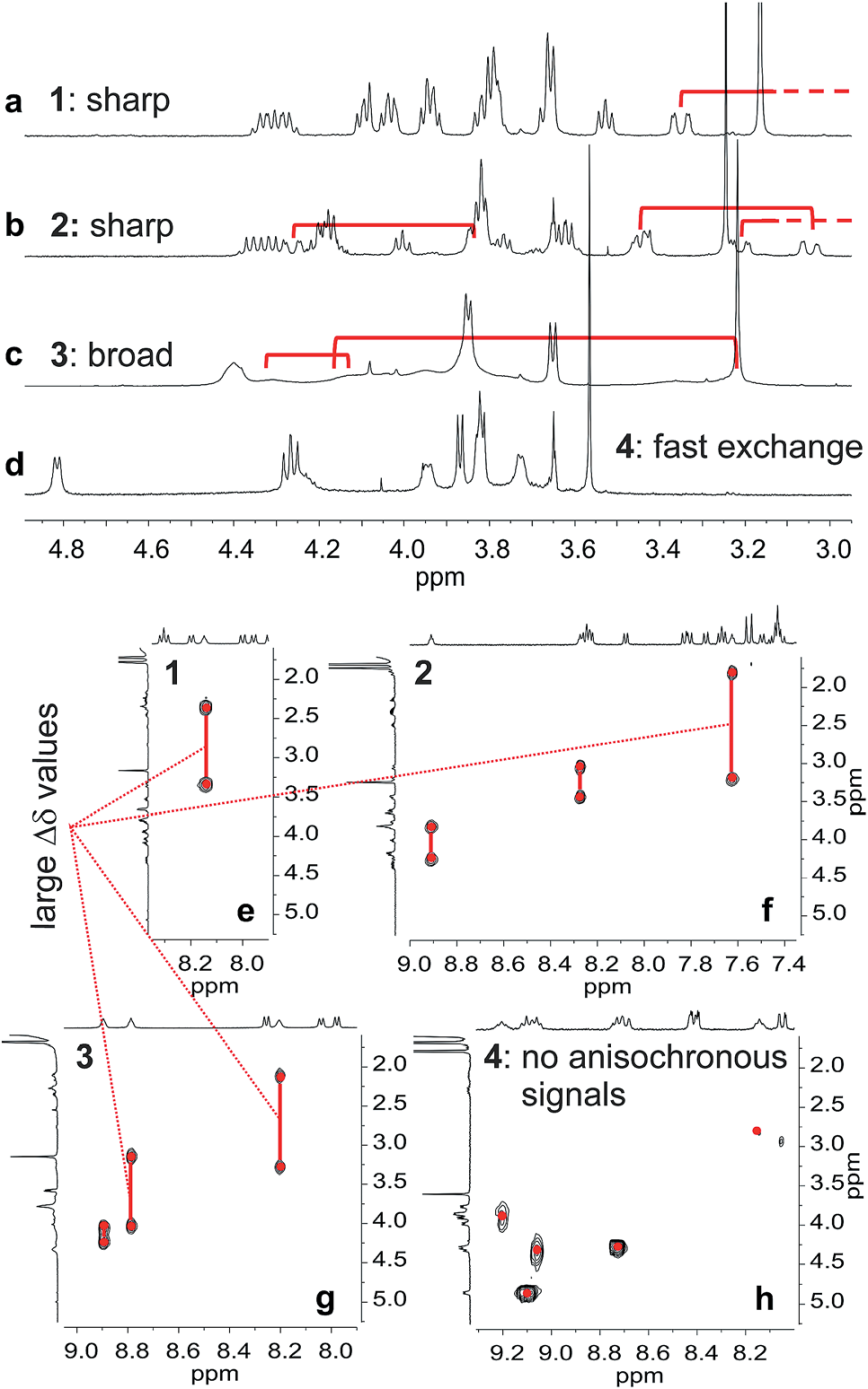
Extracts of ^1^H NMR spectra in CDCl_3_ at 25 °C of **1** (a), **2** (b), **3** (c) and **4** (d) showing side chain and main chain CH_2_ protons. Part of 2D TOCSY NMR spectra in CDCl_3_ of **1** (e), **2** (f), **3** (g) and **4** (h) showing NH–CH_2_ scalar couplings. Red brackets in (a)–(c) indicate pairs of main chain diastereotopic benzylic CH_2_ protons as observed in (e)–(g), respectively.

The behaviour of oligomers **1–4** in solution was studied by NMR spectroscopy. The ^1^H NMR spectra of **1** and **2** in CDCl_3_ exhibited features typical of the expected single helical folding. The diastereotopic motifs of the CH_2_ signals of the isobutoxy side chains of the **Q** units indicate a slow helix handedness inversion on the NMR timescale (Fig. 3a and b). For **1** no coalescence was observed upon heating up to 55 °C (Fig. S1†) but for **2** signals start to coalesce at 45 °C (Fig. S2†). In contrast, the ^1^H NMR spectrum of **3**, that differs from **2** only for the position of **P** units in the sequence, shows broad signals at 25 °C (Fig. 3c). Upon heating these broad signals sharpen and, upon cooling, all signals coalesce before splitting into sharp diastereotopic motifs (Fig. S3†). In the case of compound **4**, the broad ^1^H NMR spectrum at 25 °C (Fig. 3d) sharpens upon heating, but becomes broader without showing the emergence of any diastereotopic pattern at low temperature (Fig. S4†). These results reflect an enhancement of conformational dynamics when the number of **P** units increases, and also depending on their position.

**Fig. 4:**
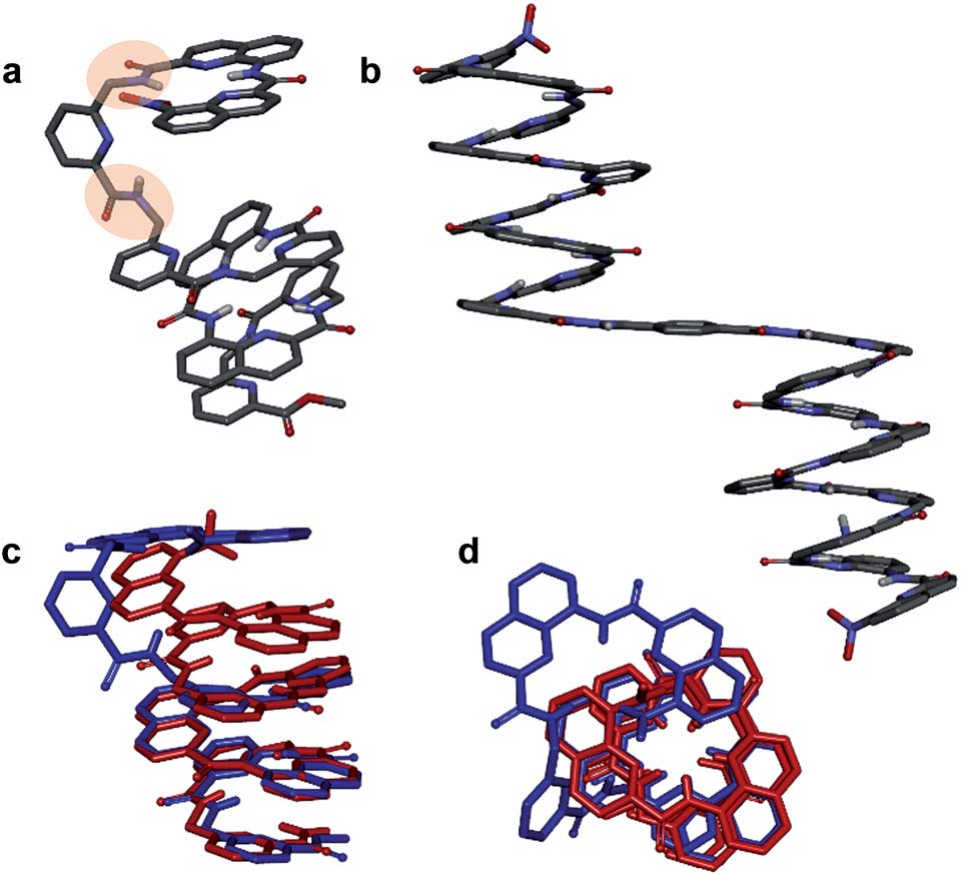
Crystal structures of compounds **4** (a) and **8a** (b). Overlay of the structure of **4** (blue) and of the *M* helix fragment of **8a** (red) (c and d). 90° kinks in the backbone of **4** occur at main chain methylene groups and are highlighted in (a). Side chains of **Q**, **P**, **T**, **X[combining low line]** and **Y[combining low line]**, included solvent molecules and hydrogen atoms other than NH have been omitted for clarity.

Helix folding was also assessed from the chemical shift values of the benzylic methylene protons of each **P** or **Y[combining low line]** monomer. Within an aromatic helix, these protons are diastereomeric and may be both strongly anisochronous and significantly upfield shifted.^36^ Distinct signals are then observed provided helix handedness inversion is slow, notably in 2D NMR (TOCSY) experiments monitoring CH_2_–NH scalar couplings (Fig. 3e–h). The spectra of compounds **1–3** indeed showed that the signals of these protons are spread over almost three ppm from 4.3 to 1.8 ppm, and that anisochronicity (Δ*δ*) can be as high as 1.4 ppm. This contrasts with the spectrum of **4** where no anisochronicity (*i.e.* fast dynamics or no chiral conformation) and less significant upfield shifts (signals clustered between 4 and 5 ppm) are observed (Fig. 3h). Solution data thus suggest different stabilities of the helical conformations **1–3** and no strong evidence of the helical folding of **4**. A crystallographic solid state structure provided valuable information, showing **4** with part of its sequence folded in a helix, and part of it unfolded (Fig. 4a). Though this structure probably simply represents one of many conformations available to **4** in solution, it is illustrative of the additional flexibility introduced by the insertion of several aliphatic amine units in the backbone and of the fact that its helical folding may not be complete.

We also studied the solution phase folding of protected sequences **5a–8a**. All these compounds comprise two potentially helical segments that can each have *P* or *M* handedness, separated by a turn unit **T**. The ^1^H NMR spectra of sequences **5a** and **6a** show two sets of signals consistent with the coexistence of *PM* and *PP*/*MM* conformers in slow exchange on the NMR timescale (Fig. S5a and b†). Based on earlier studies^32^,^42^ we assume the favoured and most abundant species to be *PM* (see also below). Compound **7a** shows a broad NMR spectrum suggesting a faster exchange between *PM* and *PP*/*MM* conformations (Fig. S5c†), consistent with the dynamics of oligomer **3**. One set of sharper NMR signals are observed in the case of **8b** (Fig. S5d†) indicating a fast exchange regime, as confirmed by the absence of diasterotopic motifs in the signals of isobutoxy side chains. An X-ray crystal structure of **8a** shows the most stable *PM* conformer (Fig. 4b). As opposed to **4**, **8a** crystallized with both its segment folded into a canonical aromatic helix despite the high number of aliphatic amine units. The overlay of the X-ray structures of **4** and **8a** makes it clear that conformation variations occur due to rotations at the methylene moieties of **P** units (Fig. 4c and d).

### Tertiary folding

**Fig. 5:**
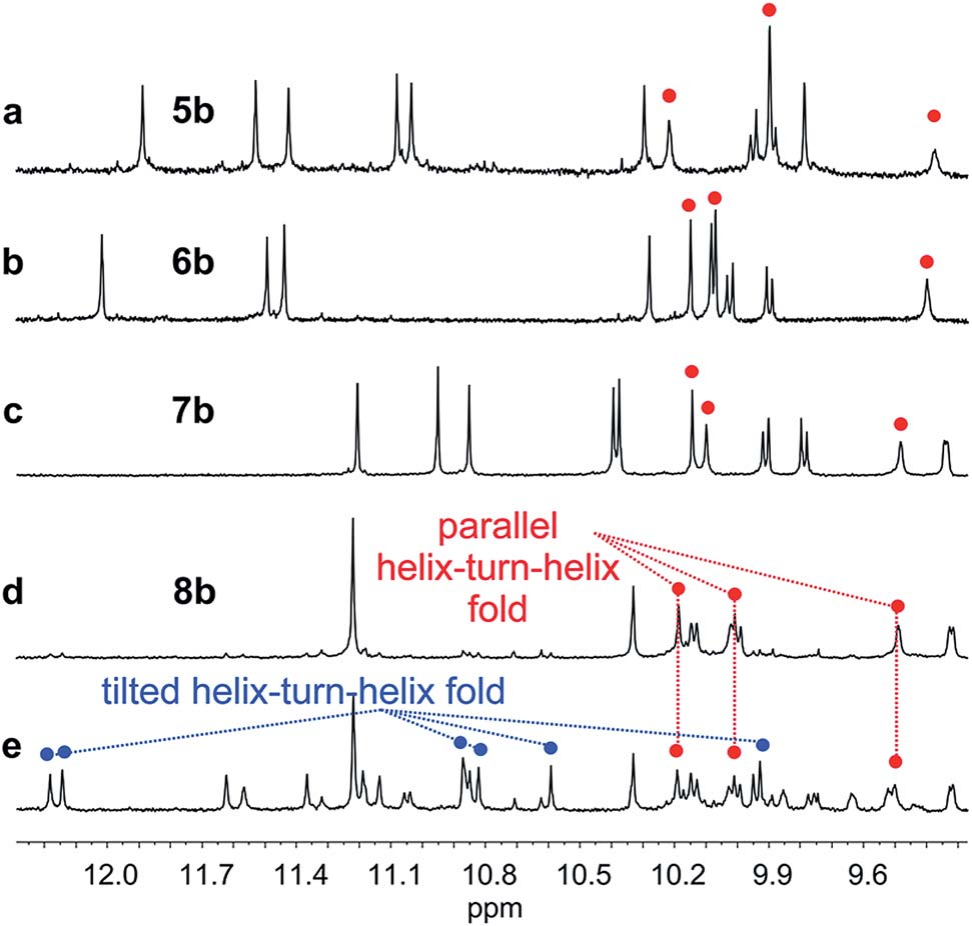
Extract of ^1^H NMR spectra (500 MHz, CDCl_3_) showing the NH and OH resonances of **5b** (a), **6b** (b), **7b** (c) and **8b** freshly dissolved crystals (d) and at equilibrium (e). The red and blue dots indicate signals corresponding to OH protons.

**Fig. 6:**
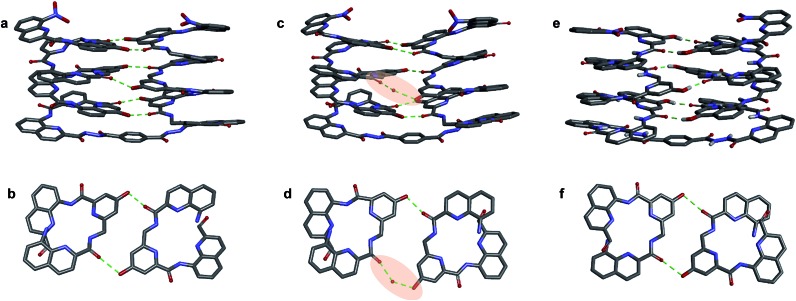
Crystal structures of compounds **7b** (a–d) and **5b**^32^ (e and f). Two symmetry independent molecules of **7b** are present in the asymmetric unit (a, b and c, d). They differ by the presence of an H-bonded water molecule (highlighted). In all representations, side chains of **Q**, **P** and **T**, included solvent molecules and most hydrogen atoms have been omitted for clarity.

We then explored the folding propensity of the corresponding deprotected sequences **5b–8b**. Compounds **5b–7b** all show one set of sharp NMR signals (Fig. 5a–c) consistent with well-defined helix–turn–helix motifs in which the handedness of the two helical segments are not independent from each other. A solid state structure of **7b** was obtained. The data quality for **7b** is low and conclusions drawn from the structure shall be treated with some care but the structure still represent a valuable piece of information regarding main chain shape and its nearest environment. The asymmetric unit of **7b** in the crystal contains two symmetry independent molecules (Fig. 6a–d). Both molecules adopted the expected *PP* or *MM* helix–turn–helix fold with inter-helix O···O distances in the range expected for hydrogen bonds between OH groups of **X** and **Y** units as a donors and amide carbonyls as acceptors (Table S1†).

One of the two independent molecule showed no noticeable differences with the crystal structure of **5b**^32^ (Fig. 6e and f), confirming the equivalent contribution to curvature of **P** and **Q** units. For the second independent molecule of **7b**, the structure is overall identical except for an electron density peak that could be interpreted as a water molecule inserted in the array of hydrogen bonds, bridging the OH of a **Y** unit and the carbonyl amide of the other helix (Fig. 6c and d). Such water-mediated hydrogen-bonded bridges are common in biopolymer structures. Here, it hints at possible mechanisms through which polar solvents disrupt the hydrogen-bonded helix–turn–helix fold. It may also be related to the non-optimal nature of hydrogen bonds within this fold: helices have been shown to assemble into other structures such as trimers or tilted dimers when they are not connected by the turn unit.^32^

Oligomer **8b** which contains the largest number of **P** units behaves differently. Its NMR spectrum at equilibrium in CDCl_3_ is also sharp but it consists of more than one set of signals (Fig. 5e), indicating the coexistence of different conformations in solution. Changing the solvent to toluene led to variations of the proportions between these species (Fig. S24 and 25†). Crystals of **8b** could be grown from toluene/hexane but they proved to be unsuitable for X-ray diffraction analysis. Nevertheless, an NMR spectrum of freshly dissolved crystals in CDCl_3_ showed one set of sharp signals (Fig. 5d) corresponding to the major species observed at equilibrium in CDCl_3_.^43^ The signals of other species progressively emerge over the course of hours (Fig. 5d, e and S22†). The ^1^H NMR spectrum of the major species of **8b** resembles those of **5b–7b** (Fig. 5a–d). Signals corresponding to OH protons hydrogen bonded at the helix–helix interface were all found in the same 9.5 to 10.2 ppm window (Fig. S10–S13†). Based on this, we tentatively assigned the major species in CDCl_3_ solutions of **8b** to a canonical helix–turn–helix structure similar to those of **5b–7b**. For the minor species present in equilibrated solutions of **8b**, OH proton signals were found in the 10.5–12.5 ppm window suggesting other folding modes with stronger hydrogen bonds. Such downfield shifted OH signals were previously observed in the trimers and tilted dimers that prevail in the absence of turn unit.^32^ The spectrum of **8b** does not change with concentration (data not shown), ruling out the coexistence of aggregated species. We thus assigned the other species to tilted helix–turn–helix folds. Indeed, energy minimized molecular models (Fig. S31 and S32†) show that the presence of four flexible **P** units close to the turn allows for considerable conformational freedom. Upon unfolding the central **PPTPP** segment of **8b** into an extended conformation, it can behave as a long turn between helices in a titled helix–turn–helix structure. In this conformation the two helix axes are not anymore parallel, yet all hydroxy groups are involved in hydrogen bonds.

The prevalence of *PP* or *MM* helix–turn–helix structures in **5b–8b** and the corresponding sharp sets of ^1^H signals much differ from the dynamic behaviour of their protected precursors. AB diastereotopic patterns for **5b–8b** indicate slow exchange of helix handedness on the NMR time scale, in contrast with *e.g.***7a** and **8a**. In addition, *PM* species present in solutions of the protected precursors are absent once the tertiary motifs form. In the case of **8a**, other species coexist but the very slow dynamics much contrasts with the fast dynamics of **4** and **8a**. Altogether, and referring to the double mutant cycle shown in Fig. 2, these results suggest that helix–turn–helix folds are stable even when the helical segments taken individually are very dynamic or, possibly, not fully helical. Such cooperativity is reminiscent to that observed in protein tertiary structure folding.

### Effects of polar solvent and heat

**Fig. 7:**
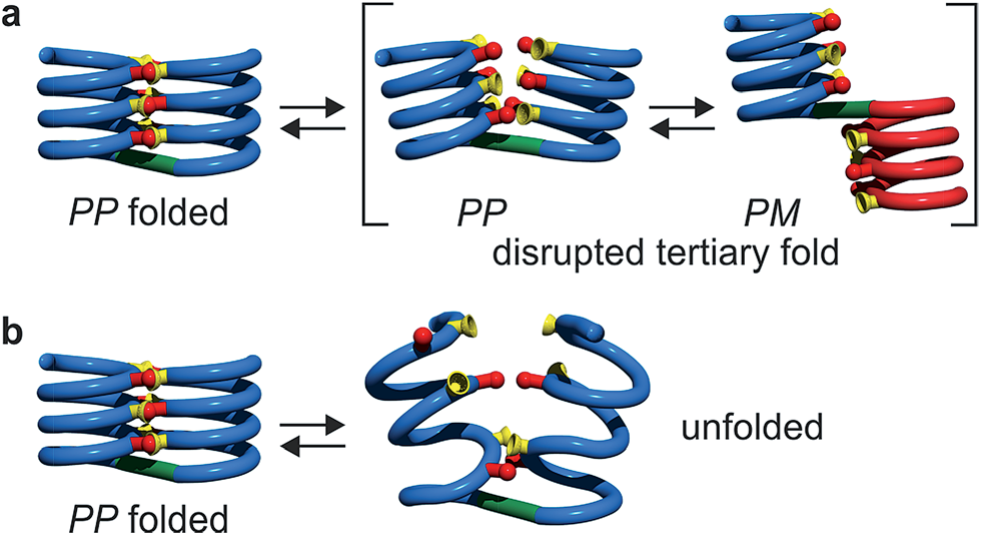
Tertiary structure disruption (a) or unfolding (b) occur upon adding a polar solvent, depending on whether the secondary helical modules are inherently stable (a) of have gained stability in the tertiary fold (b).

Tertiary folding was further evaluated through polar solvent addition as well as heating. Thus, the effect of the progressive addition of DMSO-d_6_, a solvent that competes for hydrogen bonding, was assessed by ^1^H NMR by monitoring chemical shift variations (Fig. S14–S23†). It should be noted that DMSO only has a weak effect on aromatic helix stability: **Q_n_** helices are very stable in all non-protic solvents and extremely stable in protic solvents.^34^ The prime effect of adding DMSO-d_6_ is to disrupt inter-helix hydrogen bonds. For compound **5b**, a sharp transition between two states state was observed suggesting an all-or-nothing phenomenon: the addition of up to 13% DMSO had little effect whereas the helix bundle went from folded to completely disrupted upon increasing DMSO content up to 28% DMSO. This can be understood if the two helical segments behave as rigid bodies that can establish all six hydrogen bonds between them, or none, *i.e.* that there is a strong chelate effect between the hydrogen bonds. The addition of DMSO then leads to hydrogen bond disruption but not to complete unfolding as the secondary modules remain intact even though their respective handedness become independent (Fig. 7a). In contrast, the transition observed for compounds **6b–8b** was more progressive and, for the two most flexible sequences, changes started with less than 5% DMSO. Furthermore, at high DMSO content, signals are broad and do not split into two sets of signals as for **5b**. It thus appears that enhancing helix flexibility increases sensitivity to polar solvent, and allows for partial unfolding to occur. This suggests a progressive unfolding of the tertiary motif and simultaneous destabilization of secondary structure (Fig. S18–S23†). As shown in Fig. 7, we thus relate the different behaviour of the oligomers to the rigidity of each helix backbone, that is, to the ratio and sequence of **P** and **Q** monomers. Although tertiary fold disruption undergoes a sharper transition for rigid helices because of a chelate effect, cooperativity between secondary and tertiary folds, *i.e.* the stabilization of secondary folds in the tertiary structure, becomes apparent only for the more flexible sequences.

Another possible consequence of flexible units was an enhancement in the folding entropy that would result in the possibility to denature, or “melt” the structures upon heating. We measured NMR spectra in CDCl_3_ up to 55 °C (Fig. S6–S9†) and, in the case of **7b**, in C_2_D_2_Cl_4_ up to 80 °C, unfortunately this temperature was not high enough to observe any denaturation. However, some variations of chemical shift values were detected in particular for OH protons: upon heating, signals shifted upfield suggesting an increase of averaged hydrogen bond distances.

We then performed some molecular dynamic (MD) studies to evaluate the effect of temperatures higher than can be reached experimentally. MD simulations were performed under stochastic dynamic conditions at different temperatures using the program MacroModel. Simulations were carried out for 1 ns using an energy minimized initial structure. Such a short time is insufficient for a correct equilibration of the system and MD simulation data collected at very high temperatures should be considered with caution. Yet some relevant information could be deduced from the results.

**Fig. 8:**
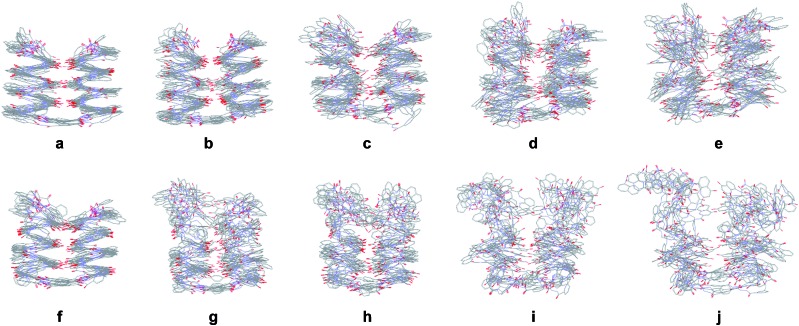
Stochastic dynamic simulations of **5b** in CHCl_3_ over 1 ns at 300 K (a), 400 K (b), 500 K (c), 600 K (d) and 700 K (e). Stochastic dynamic simulations of **8b** in CHCl_3_ over 1 ns at 300 K (f), 400 K (g), 500 K (h), 600 K (i) and 700 K (j). In each case, ten structures sampled every 100 ps are overlaid for each temperature. Side chains of **Q**, **P** and **T** have been omitted for clarity.

Single helical compounds **1** and **4** showed a marked difference of stability upon heating (Fig S30†): increasing the number of **P** units is highly destabilizing at high temperature, in agreement with solution data. We then modelled the behaviour of helix–turn–helix structures **5b** and **8b** (Fig. 8, see also ESI†). During the simulations, O···O hydrogen bond distances between the two helices were monitored and structures sampled every 10 ps. At a temperature of 300 K, the folded helix–turn–helix motif is highly conserved over time for both compounds (Fig. 8a and f). H bonds fluctuate but were present for most of the simulations, yet the central H bonds between **Y** units were found to be less stable (Fig. S28f and S29f†). This may be related to the inserted water molecule observed in the crystal structure of **7b** (Fig. 6c and d). Another feature of hydrogen bond fluctuation concerns the concerted behaviour of individual hydrogen bond pairs that occur at different levels of the helices. At moderate temperatures, it is frequently seen that one hydrogen bond of a given pair would break, or the other, but not both simultaneously (Fig. S28f, g, l and S29f, h†).

Upon increasing temperature by increments of 100 K, motions are evident and H bonds between the two helices break frequently (Fig S28 and S29†). Different behaviours were observed for the two compounds. For **5b**, the helical conformation of the backbone is mostly conserved, even at high temperature, when the tertiary fold is disrupted (hydrogen bonding distances > 5 Å), a behaviour comparable to that caused by the addition of DMSO. In addition, the conformation of each helical segment does not much differ from that of isolated helix **1** at the same temperature. For compound **8b**, partial helix unfolding start to be observed at 500 K (Fig. 8g), together with tertiary structure disruption. Furthermore, this disruption occurs by the free termini of the tertiary fold, and the helical structure near the turn unit is mostly conserved. This contrasts with the melting of single helical **4** that is more extensive at the same temperatures (Fig. S30f–j†). Altogether, the simulations confirm the conclusions drawn from the effect of adding DMSO (Fig. 7). When secondary structures are robust as for **5b**, helix–helix interactions can be disrupted without losing the secondary structures. For flexible sequences such as **8b**, the melting of tertiary and secondary folds occurs concomitantly. There is thus evidence of cooperative folding: the most flexible secondary structures are stabilized within the tertiary fold, but more subject to melting upon increasing temperature. Yet this melting occurs at high *in silico* temperatures; the temperatures that can be implemented experimentally were too low to cause melting.

## Conclusion

The rational design of abiotic tertiary folded structures as large, complex, and functionally efficient as proteins represents an exciting yet formidable challenge for chemistry. The possibility to elicit protein-like structures and properties in media completely different from water is intriguing and will certainly be enabling. First steps towards this goal are being made using aromatic oligoamide helix–turn–helix motifs stabilized by hydrogen bonds. From the start, it is important not to limit these designs to mere structures but to include dynamic aspects as well, for example how the folding of tertiary and secondary structures relate to one another and exchange with fully or partially unfolded states. We have shown here that completely abiotic structures show different types of unfolding behaviour depending on whether individual secondary modules are inherently stable or not. Both heating and polar solvents may disrupt helix–helix interactions. However, concomitant unfolding of the helices occurs only for more flexible helices that gain stability within the tertiary fold. Heating is predicted to be conducive of complete tertiary structure melting but only at temperatures above what can be reached experimentally. It might be inferred from this that the helix–turn–helix structures studied here may be tolerant towards even more flexible units than those introduced until now. For example, the **P** units may be replaced by 2-amino-ethoxy-acetic acid groups that have the same length and an ether oxygen atom instead of the pyridine endocyclic nitrogen atom. The findings reported here constitute a solid basis on which to further increase the size and complexity of these abiotic structures. Efforts towards more complex objects are in progress in our laboratory and will be reported in due course.

## Conflicts of interest

There are no conflicts to declare.

## Supplementary Material

Supplementary information

Crystal structure data
