# Supplementary material for: Interplay of secondary and tertiary folding in abiotic foldamers
Source: Chem Sci. 2019 Jun 10;10(29):6984–91. doi: 10.1039/c9sc01322a (PMC6676331; doi:10.1039/c9sc01322a)
Supplement: Supplementary file 1 [file SC-010-C9SC01322A-s001.pdf]

Electronic Supplementary Material (ESI) for Chemical Science.
This journal is © The Royal Society of Chemistry 2019

Supporting Information

Interplay of secondary and tertiary folding in abiotic foldamers

Daniela Mazzier, Soumen De, Barbara Wicher, Victor Maurizot and Ivan Huc*

Table of Contents **page**

1. Supplementary figures 2
2. Molecular modelling 15
   2.1 Molecular dinamic simulations 15
   2.2 Energy minimized models 19
3. Supplementary methods 20
   3.2 Nuclear magnetic resonance spectroscopy 20
   3.3 X-ray crystallography 20
4. Synthetic schemes 24
5. Experimental procedures 29
   5.1 General methods 29
   5.2 Synthesis of small molecules 30
   5.3 Solid phase synthesis general methods 39
   5.4 Synthesis of oligomers 39
6. References 48
7. ^{1}H and ^{13}C NMR spectra of new compounds 49
8. RP-HPLC profiles of oligomers 80

1. **Supplementary figures**

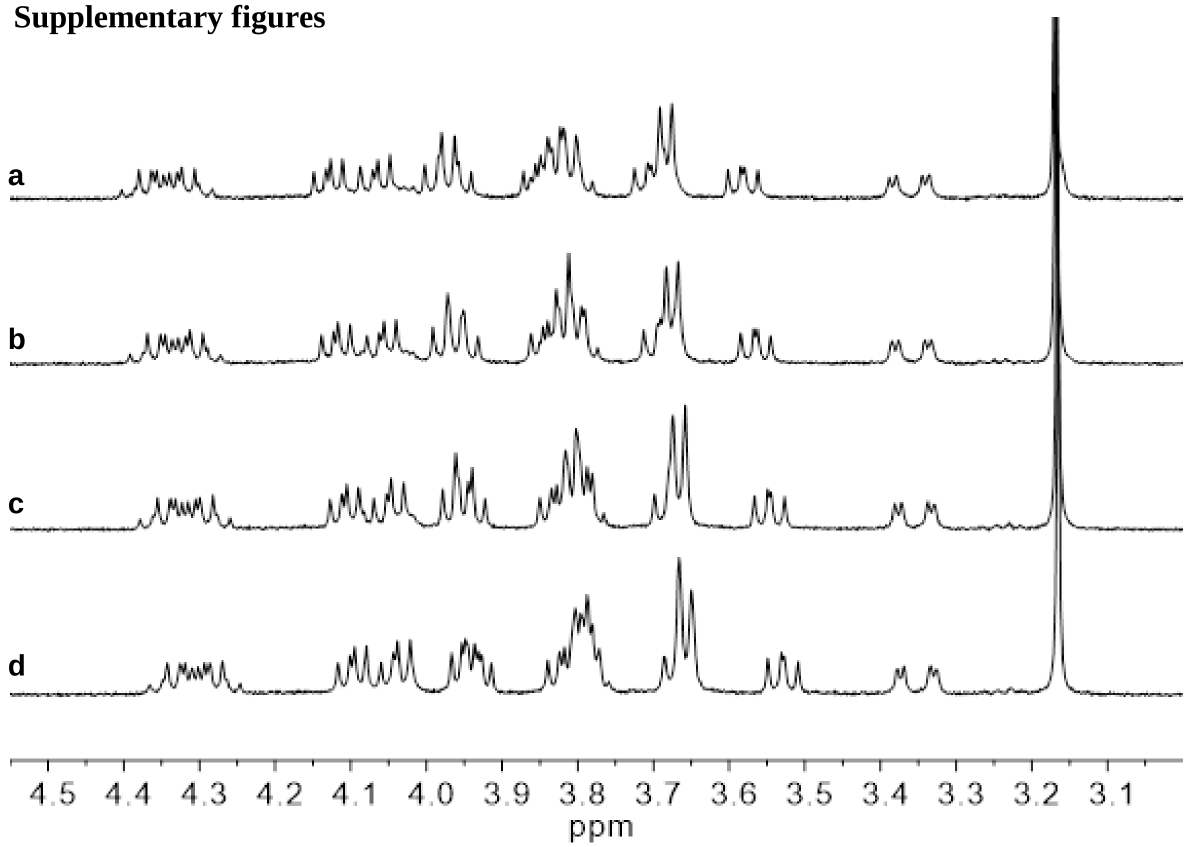

Figure S1 Extract of ^{1}H NMR spectra (400 MHz, $CDCl_3$) of **1** at 55 °C (a), at 45 °C (b), at 35 °C (c) and 25 °C (d).

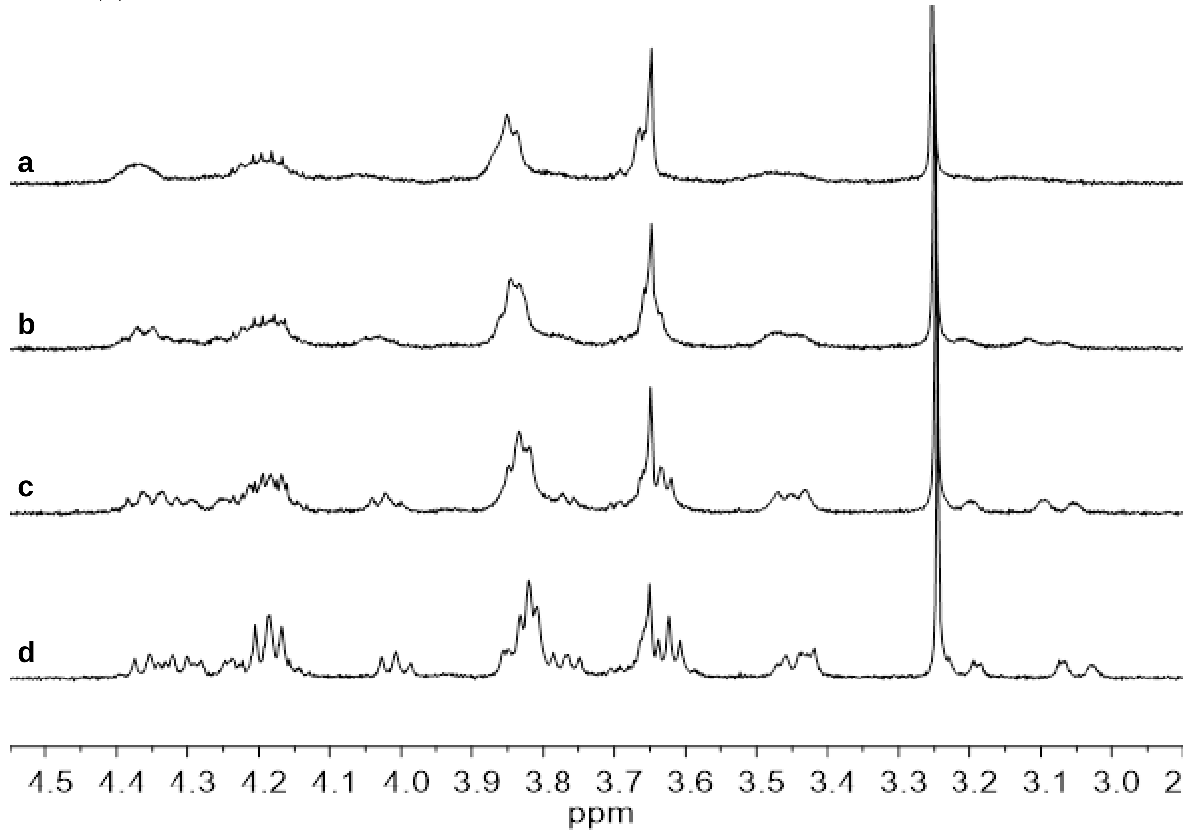

Figure S2 Extract of ^{1}H NMR spectra (400 MHz, $CDCl_3$) of **2** at 55 °C (a), at 45 °C (b), at 35 °C (c) and 25 °C (d).

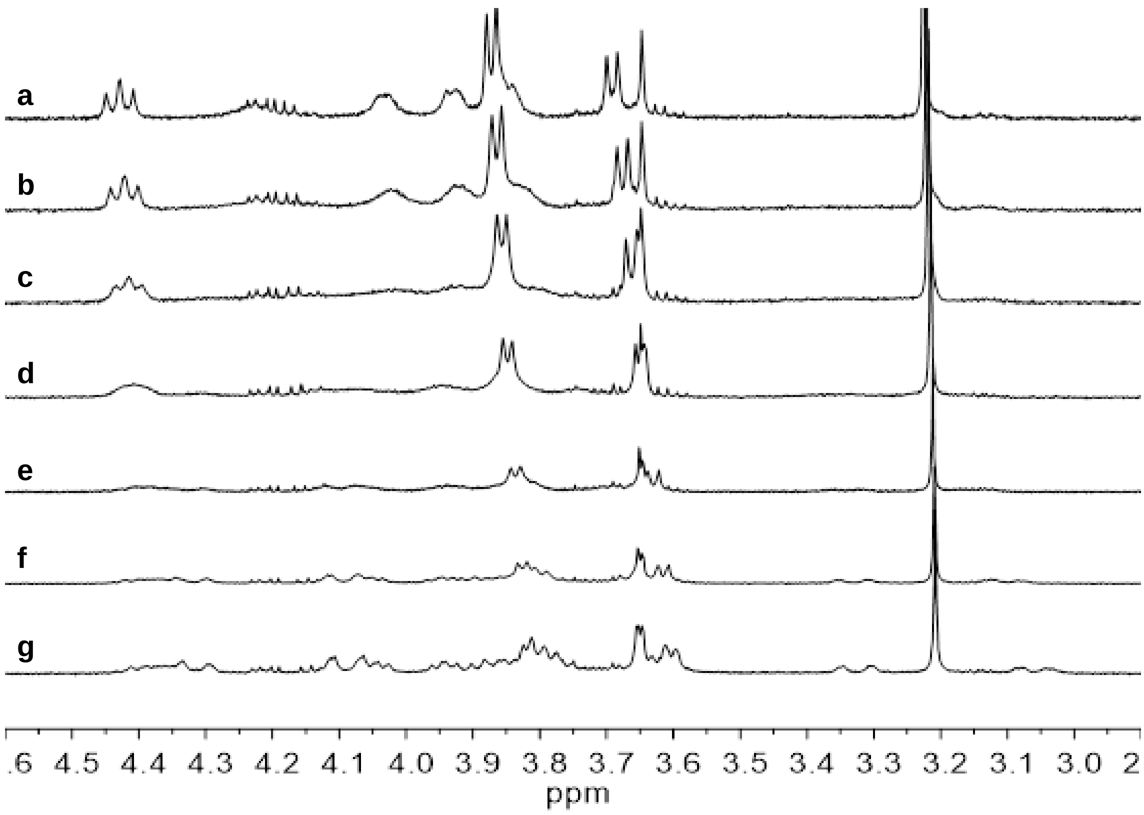

Figure S3 Extract of ^{1}H NMR spectra (400 MHz, $CDCl_3$) of **3** at 55 °C (a), at 45 °C (b), at 35 °C (c), 25 °C (d), 10 °C (e), 0 °C (f) and -10 °C (g).

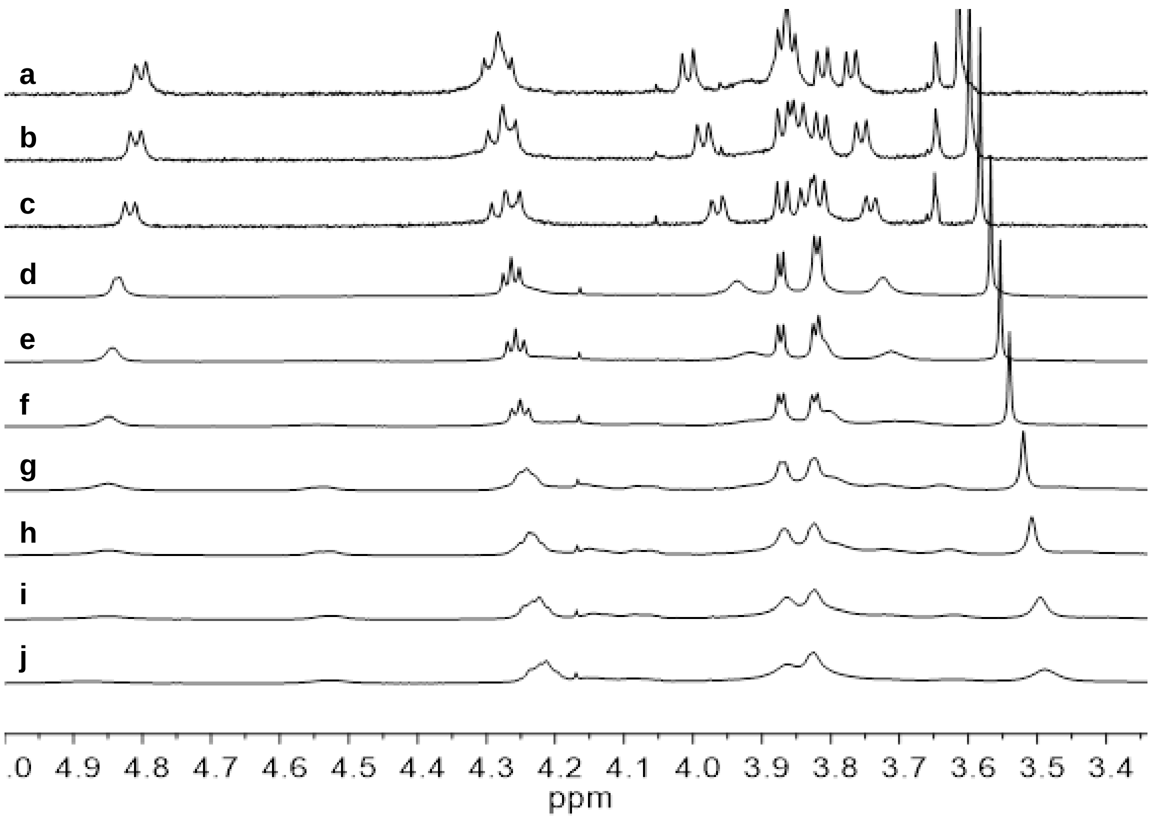

Figure S4 Extract of ^{1}H NMR spectra (400 MHz, $CDCl_3$) of **4** at 55 °C (a), at 45 °C (b) and at 35 °C (c). Extract of ^{1}H NMR spectra (700 MHz, $CDCl_3$) of **4** at 25 °C (d), 10 °C (e), 0 °C (f), -10 °C (g), -20 °C (h), -30 °C (i) and -40 °C (j).

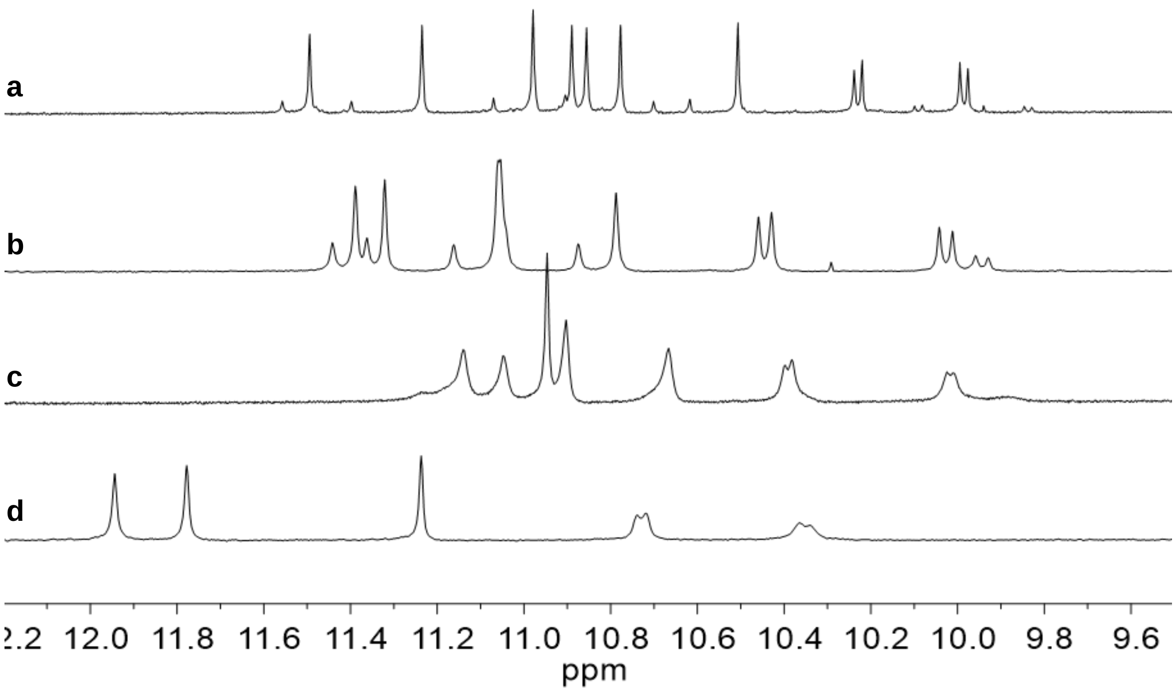

Figure S5 Extract of ^{1}H NMR spectra (400 MHz, $CDCl_3$) of **5a** (a), **6a** (b), **7a** (c) and **8a** (d).

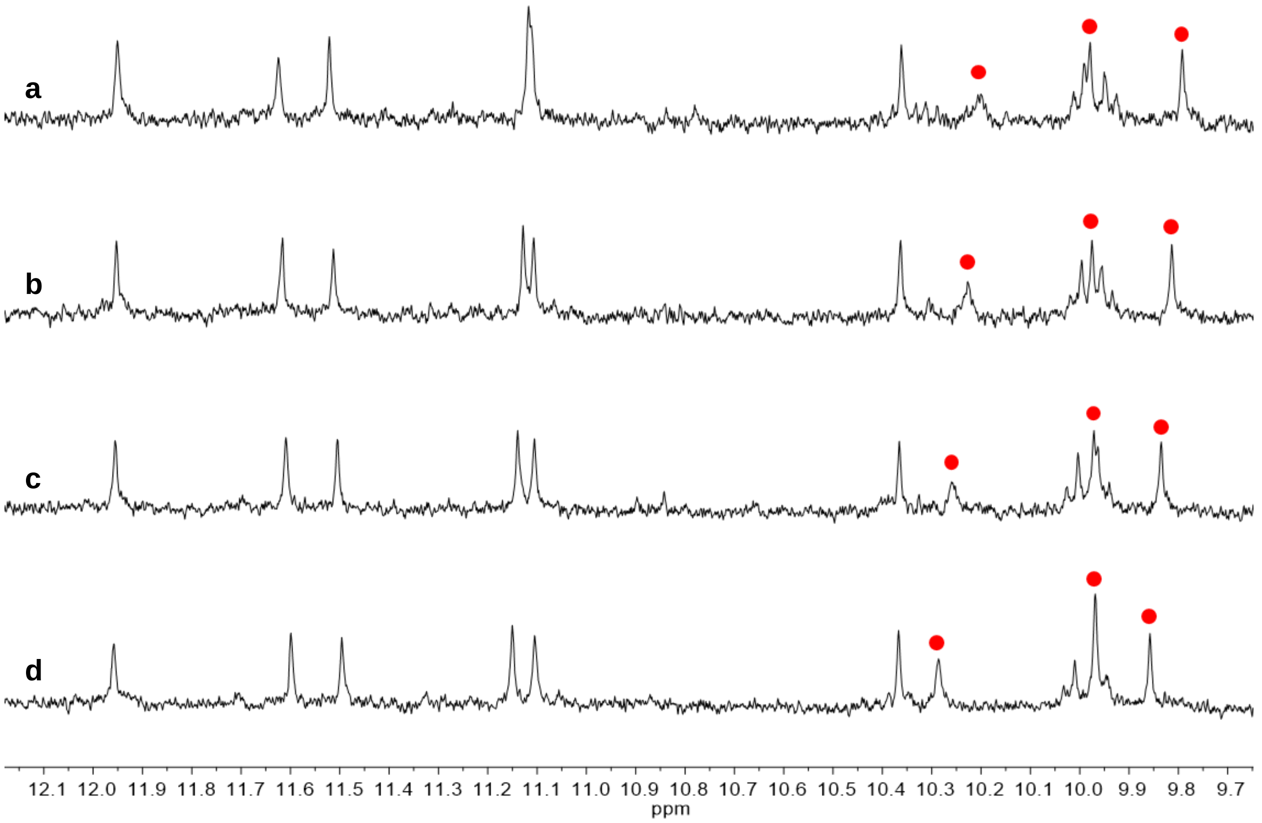

Figure S6 Extract of ^{1}H NMR spectra (400 MHz, $CDCl_3$) of **5b** at 55 °C (a), at 45 °C (b), at 35 °C (c) and 25 °C (d). Red dots indicate signals corresponding to OH protons.

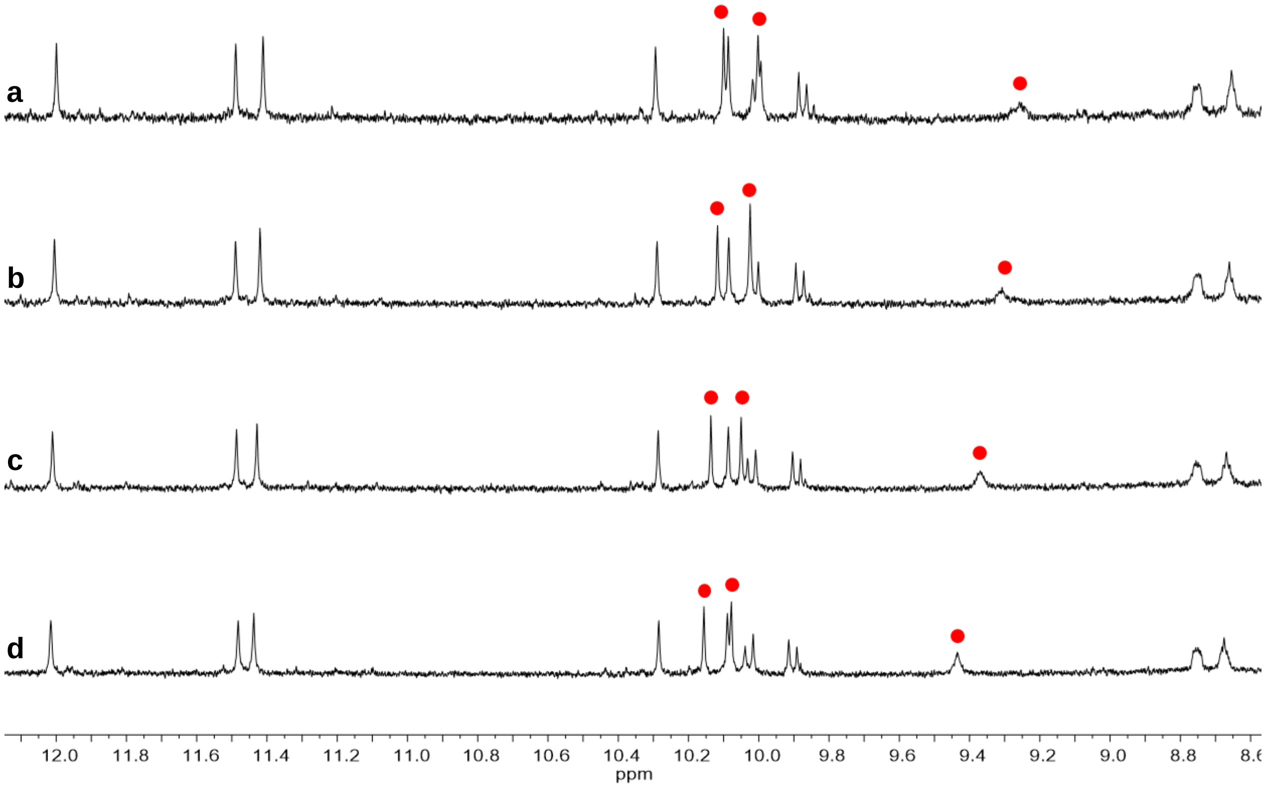

Figure S7 Extract of ^{1}H NMR spectra (400 MHz, $CDCl_3$) of **6b** at 55 °C (a), at 45 °C (b), at 35 °C (c) and 25 °C (d). Red dots indicate signals corresponding to OH protons.

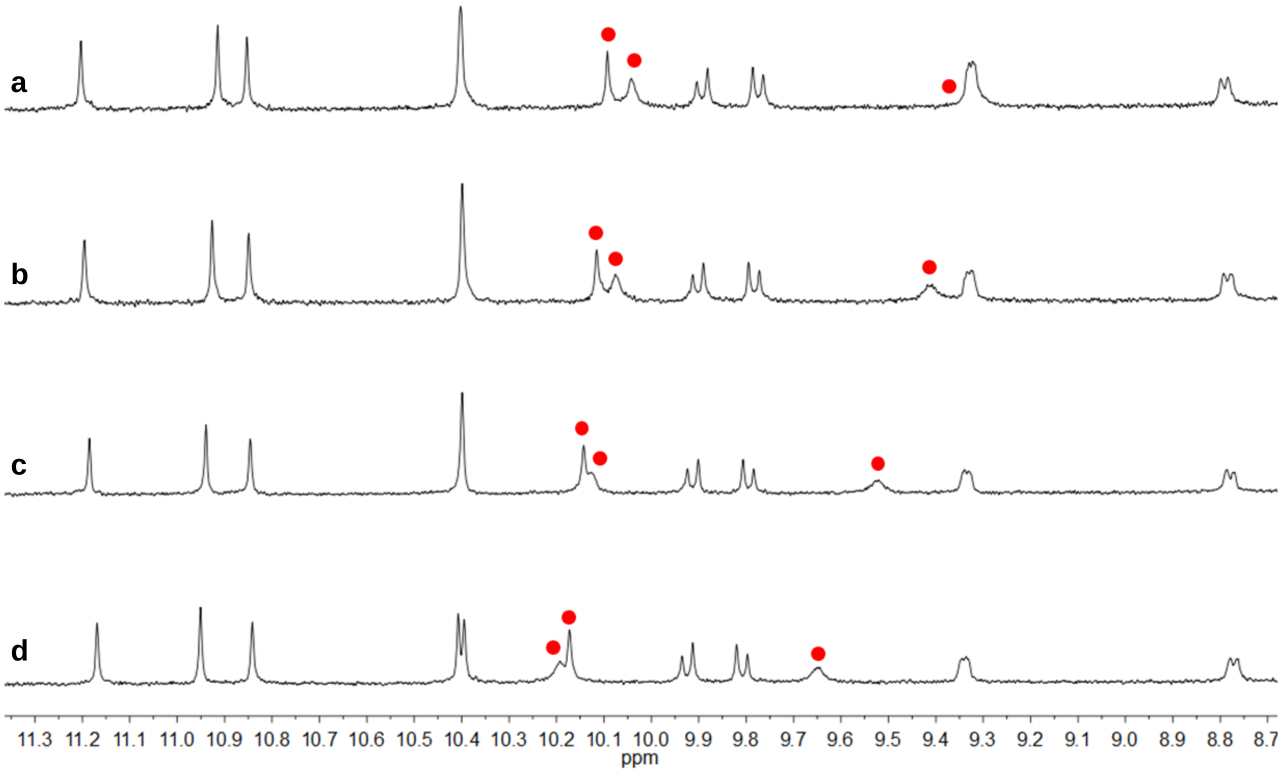

Figure S8 Extract of ^{1}H NMR spectra (400 MHz, $CDCl_3$) of **7b** at 55 °C (a), at 45 °C (b), at 35 °C (c) and 25 °C (d). Red dots indicate signals corresponding to OH protons.

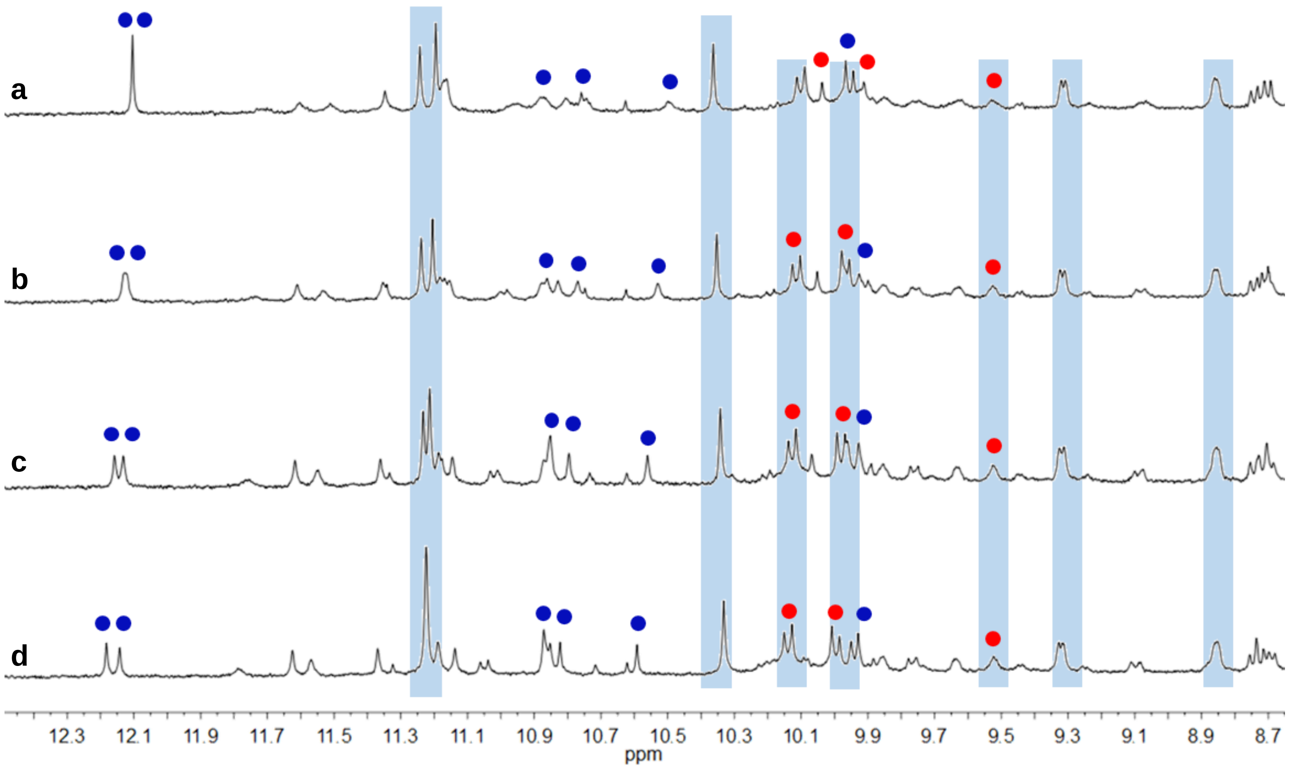

Figure S9 Extract of ^{1}H NMR spectra (400 MHz, $CDCl_3$) of **8b** at 55 °C (a), at 45 °C (b), at 35 °C (c) and 25 °C (d). Signals corresponding to the folded species are highlighted in blue. Red and blue dots indicate signals corresponding to OH protons.

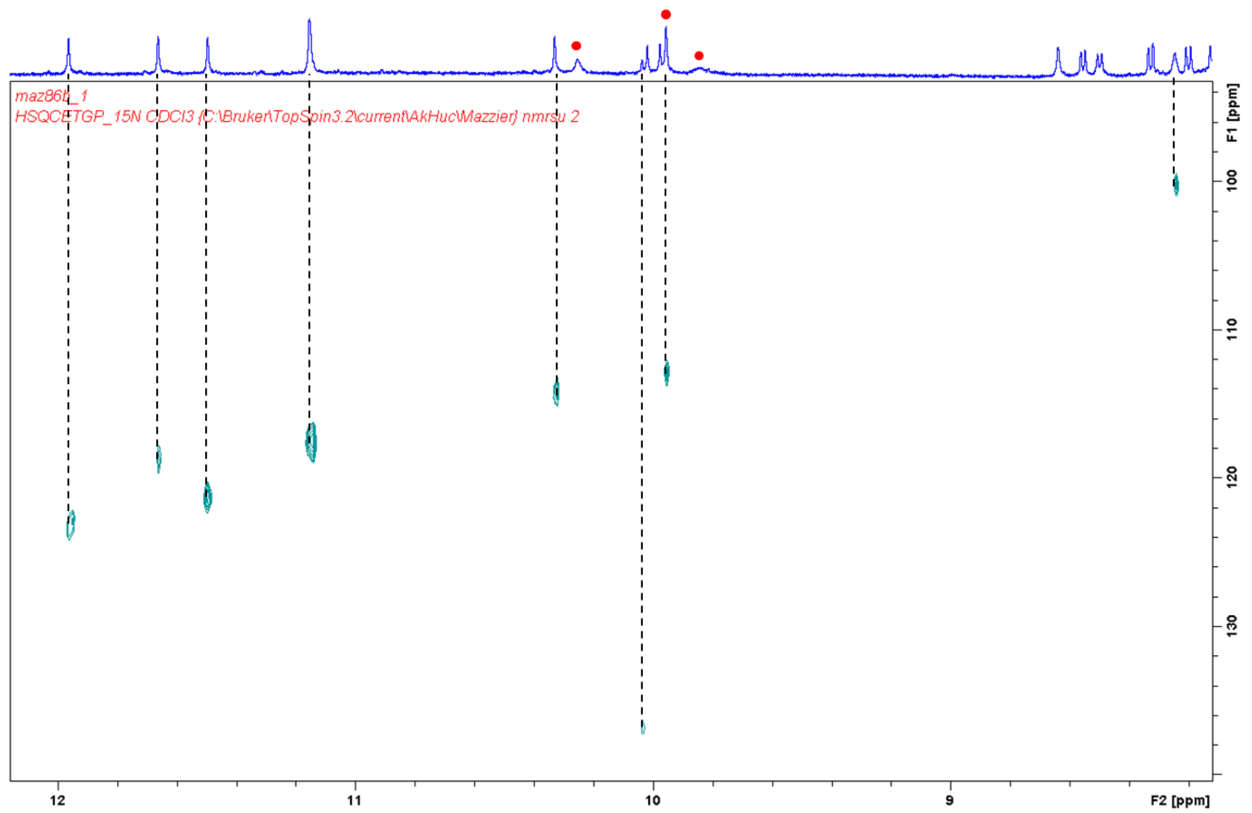

Figure S10 ^{15}N-^{1}H HSQC (500 MHz, $CDCl_3$) of **5b**. Only NH resonances correlate, red dots indicate the signals of OH protons.

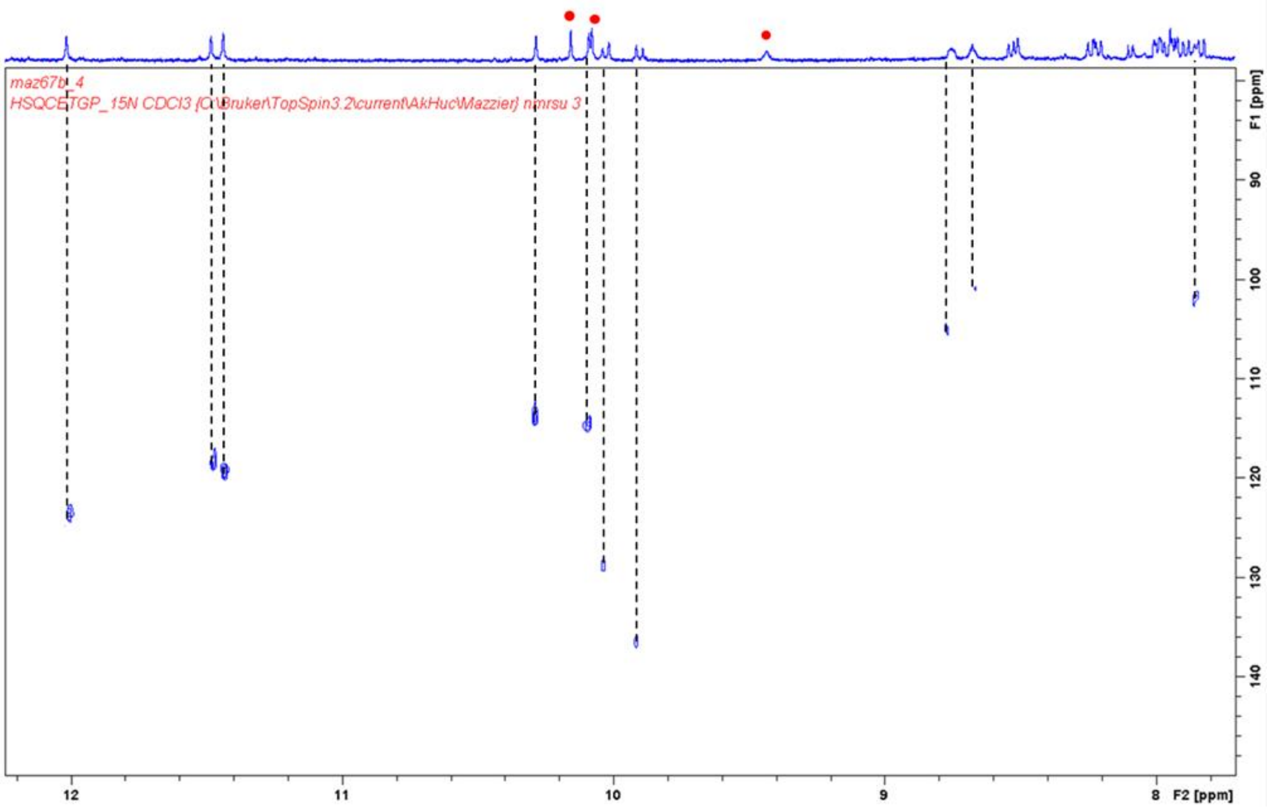

Figure S11 ^{15}N-^{1}H HSQC (500 MHz, $CDCl_3$) of **6b**. Only NH resonances correlate, dots indicate the signals of OH protons. Red dots and lines indicate signals corresponding to the folded species.

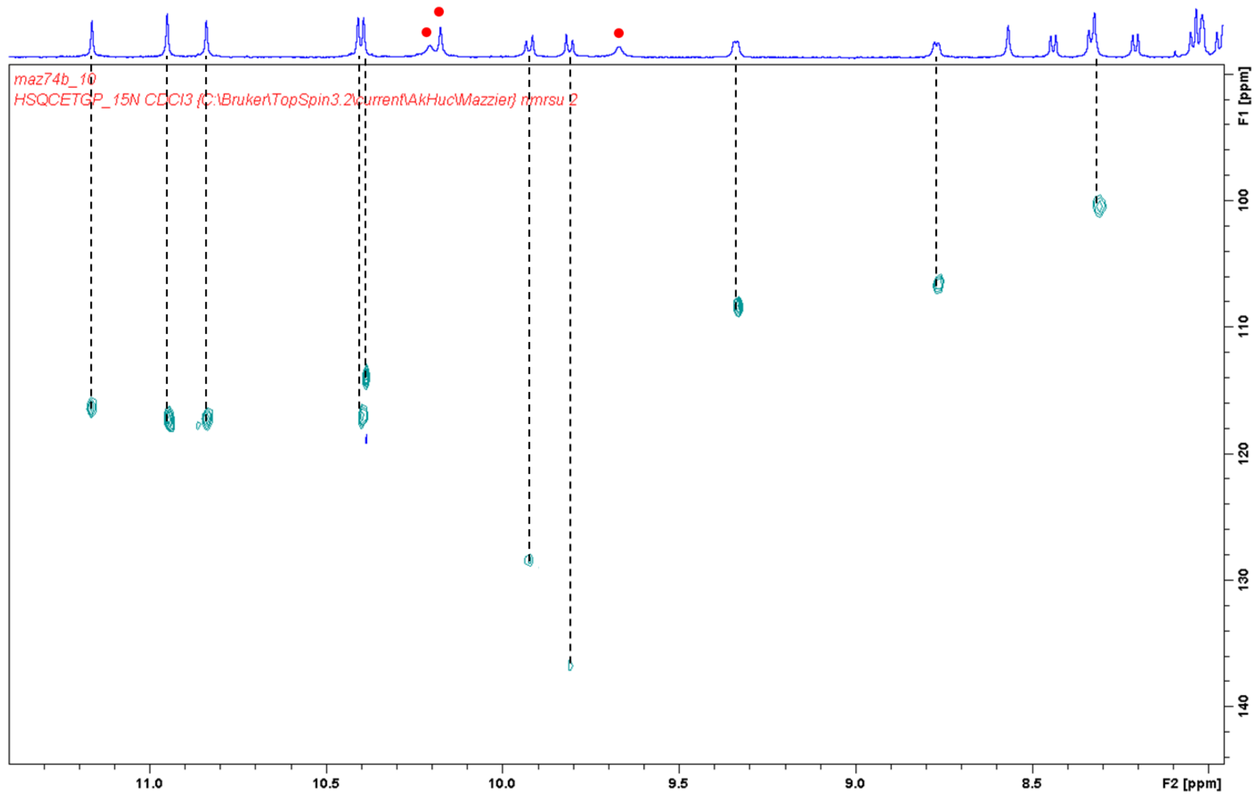

Figure S12 ^{15}N-^{1}H HSQC (500 MHz, $CDCl_3$) of **7b**. Only NH resonances correlate, red dots indicate the signals of OH protons.

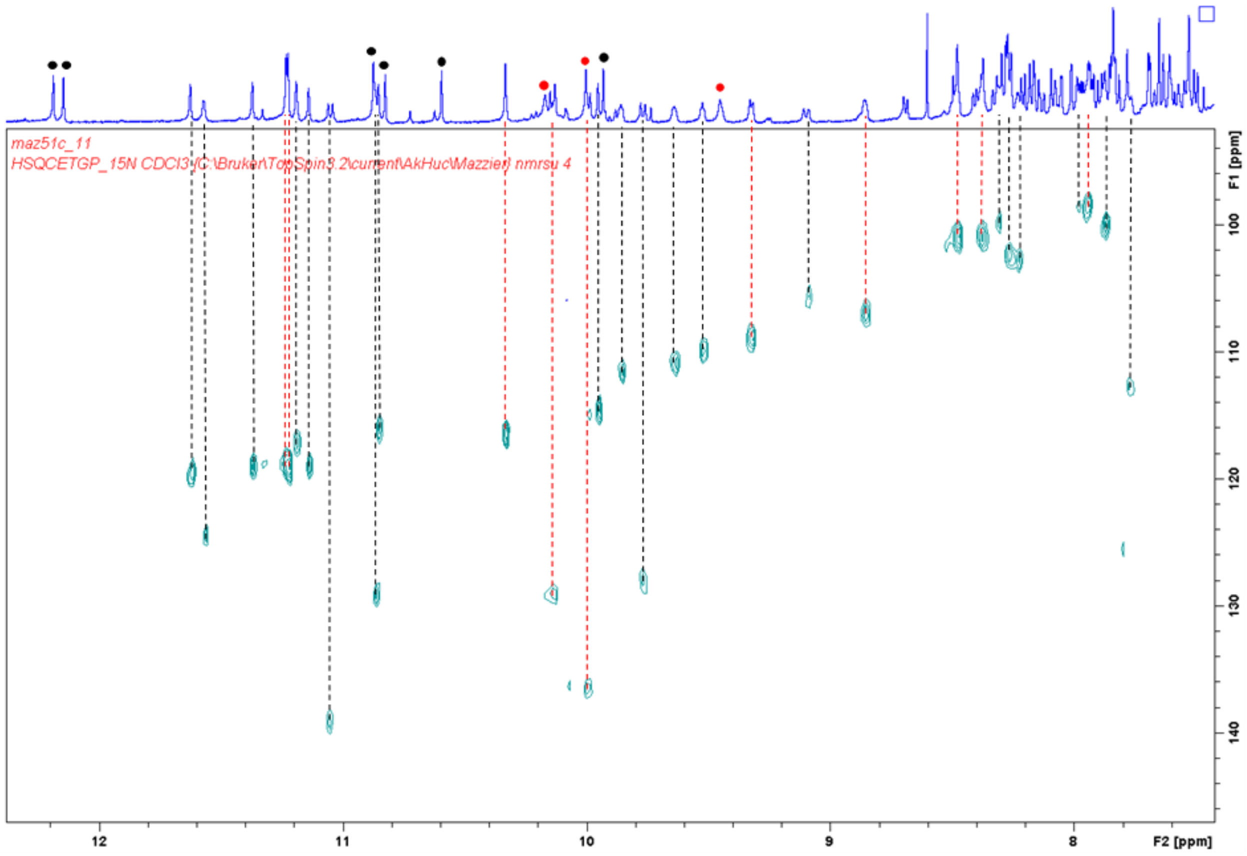

Figure S13 ^{15}N-^{1}H HSQC (500 MHz, $CDCl_3$) of **8b**. Only NH resonances correlate, dots indicate the signals of OH protons. Red dots and lines indicate signals corresponding to the folded species.

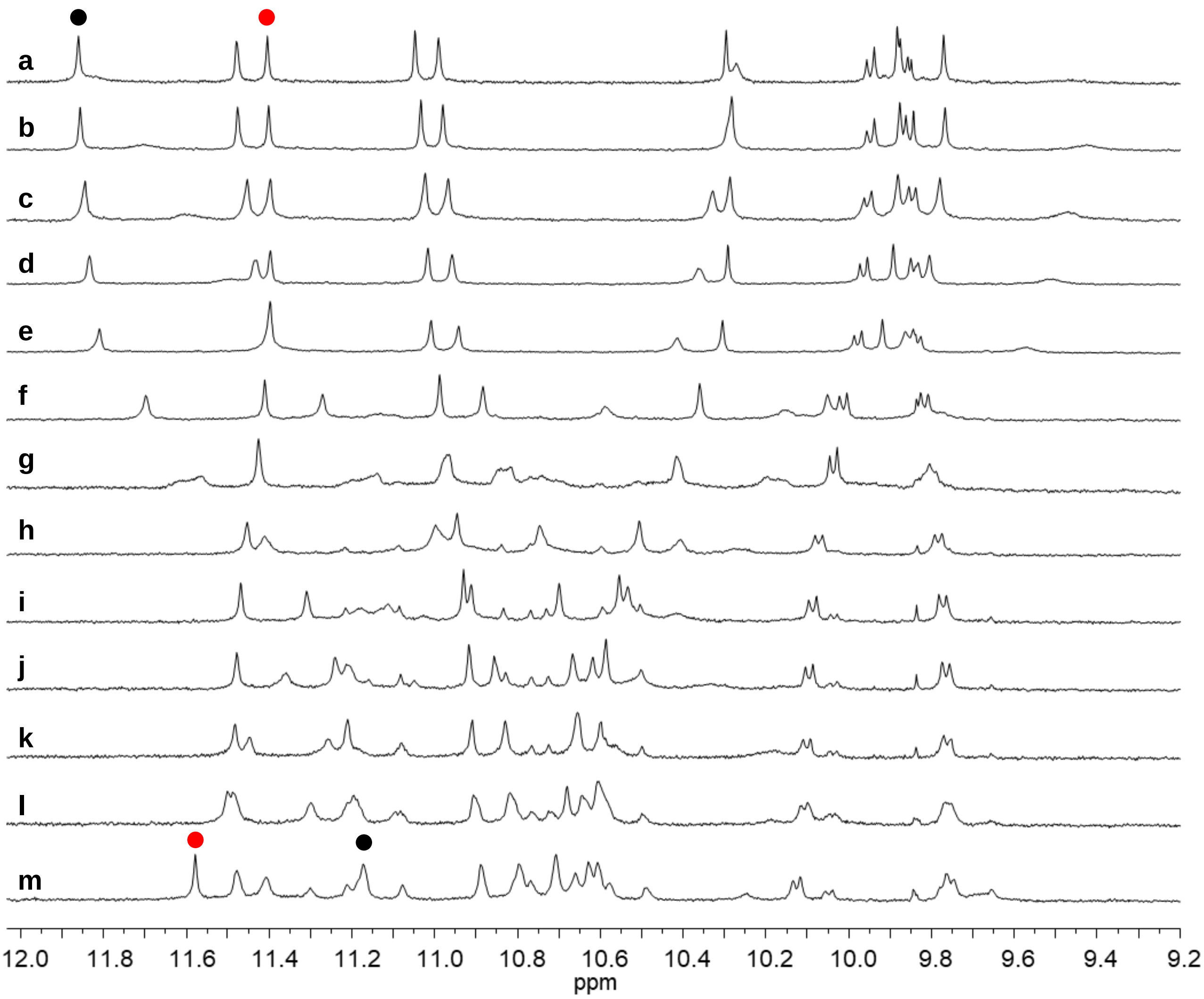

Figure S14 Part of the ^{1}H NMR spectra (500 MHz) showing amide resonances of **5b** in $CDCl_3$/DMSO-d_6. The volume percentages of DMSO-d_6 are 4 (a), 8 (b), 10 (c), 12 (d), 14 (e), 16 (f), 18 (g), 20 (h), 22 (i), 24 (j), 26 (k), 28 (l) and 30% (m), respectively. The chemical shift variations of two signals (marked with dots) are shown in Figure S16.

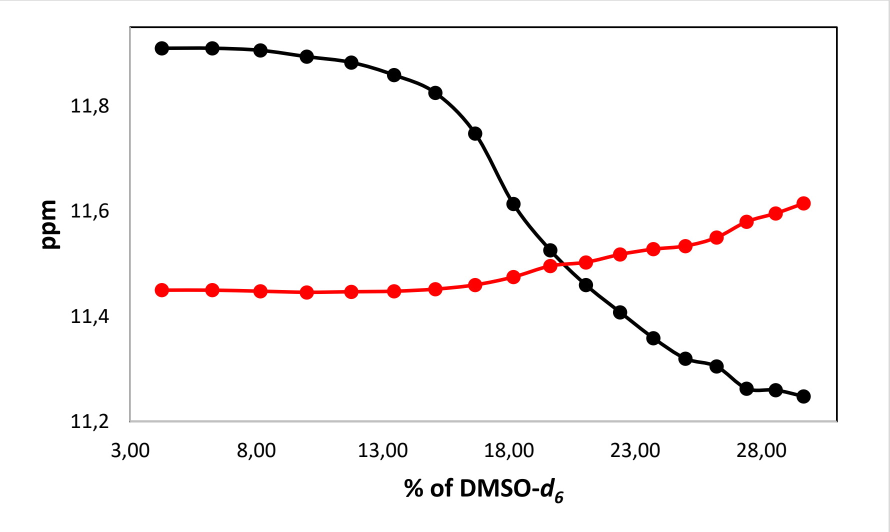

Figure S15 NMR chemical shift of amide NH protons of **5b** as a function of the volume percent of DMSO-d_6 in $CDCl_3$.

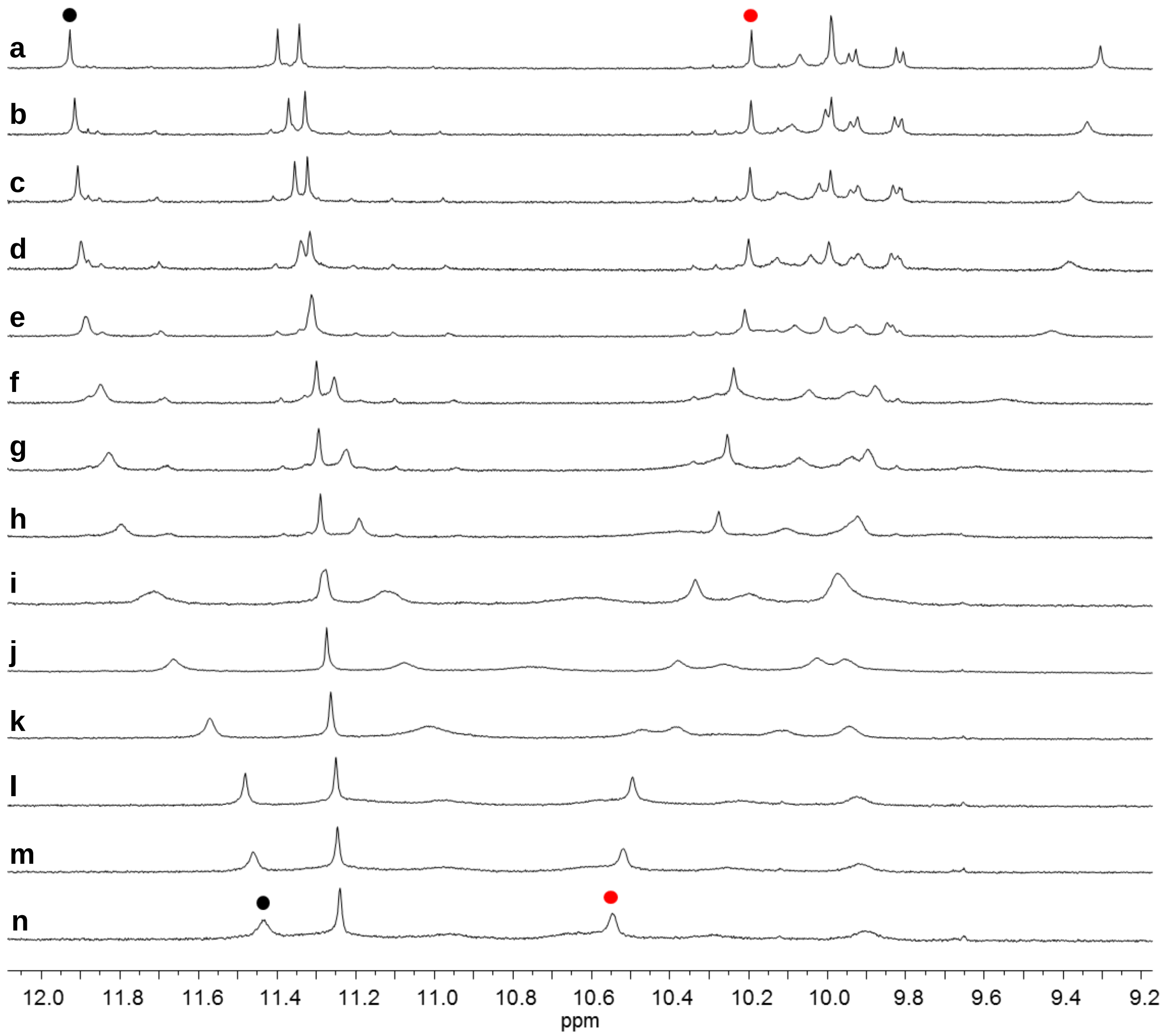

Figure S16 Part of the ^{1}H NMR spectra (500 MHz) showing amide resonances of **6b** in $CDCl_3$/DMSO-d_6. The volume percentages of DMSO-d_6 are 4 (a), 8 (b), 10 (c), 12 (d), 14 (e), 16 (f), 18 (g), 20 (h), 22 (i), 24 (j), 26 (k), 28 (l), 30 (m) and 32% (n) respectively. The chemical shift variations of two signals (marked with dots) are shown in Figure S16.

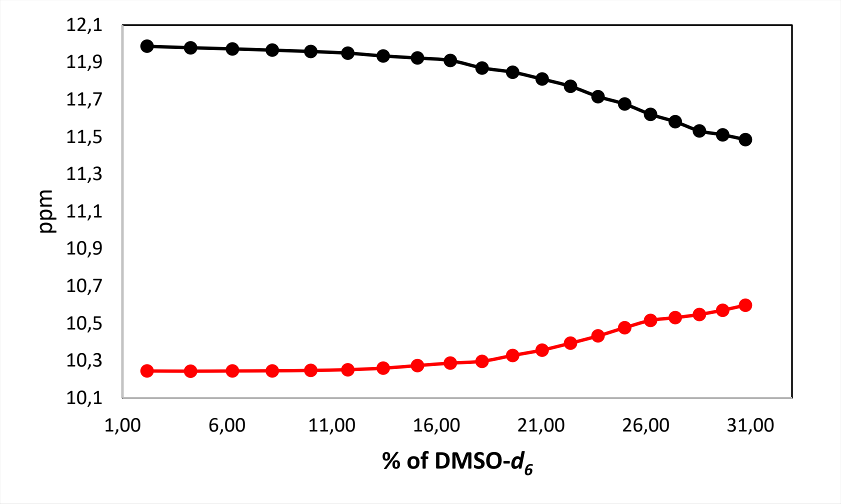

Figure S17 NMR chemical shift of two amide NH protons of **6b** as a function of the volume percent of DMSO-d_6 in $CDCl_3$.

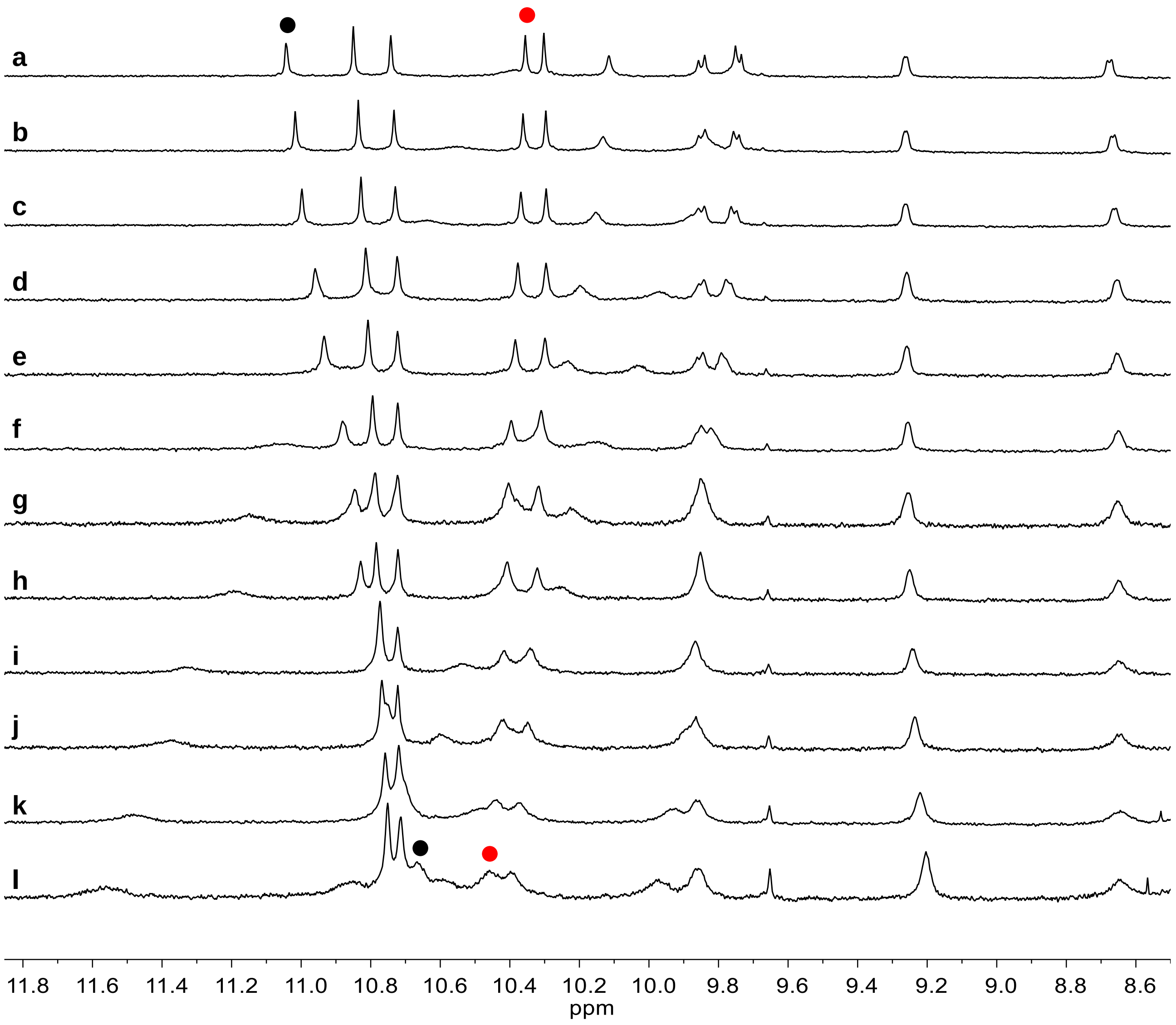

Figure S18 Part of the ^{1}H NMR spectra (500 MHz) showing amide resonances of **7b** in $CDCl_3$/DMSO-d_6. The volume percentages of DMSO-d_6 are 4 (a), 8 (b), 10 (c), 12 (d), 14 (e), 16 (f), 18 (g), 20 (h), 22 (i), 24 (j), 26 (k) and 28% (l), respectively. The chemical shift variations of two signals (marked with dots) are shown in Figure S20.

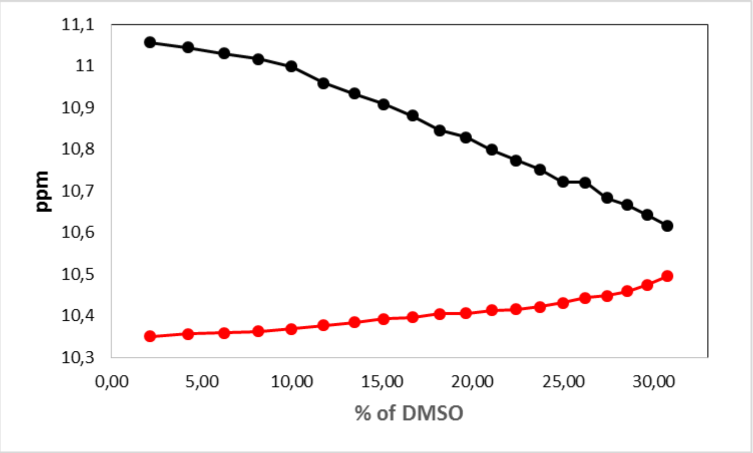

Figure S19 Variations of chemical shift of two amide NH protons of **7b** as a function of the volume percent of DMSO-d_6 in $CDCl_3$.

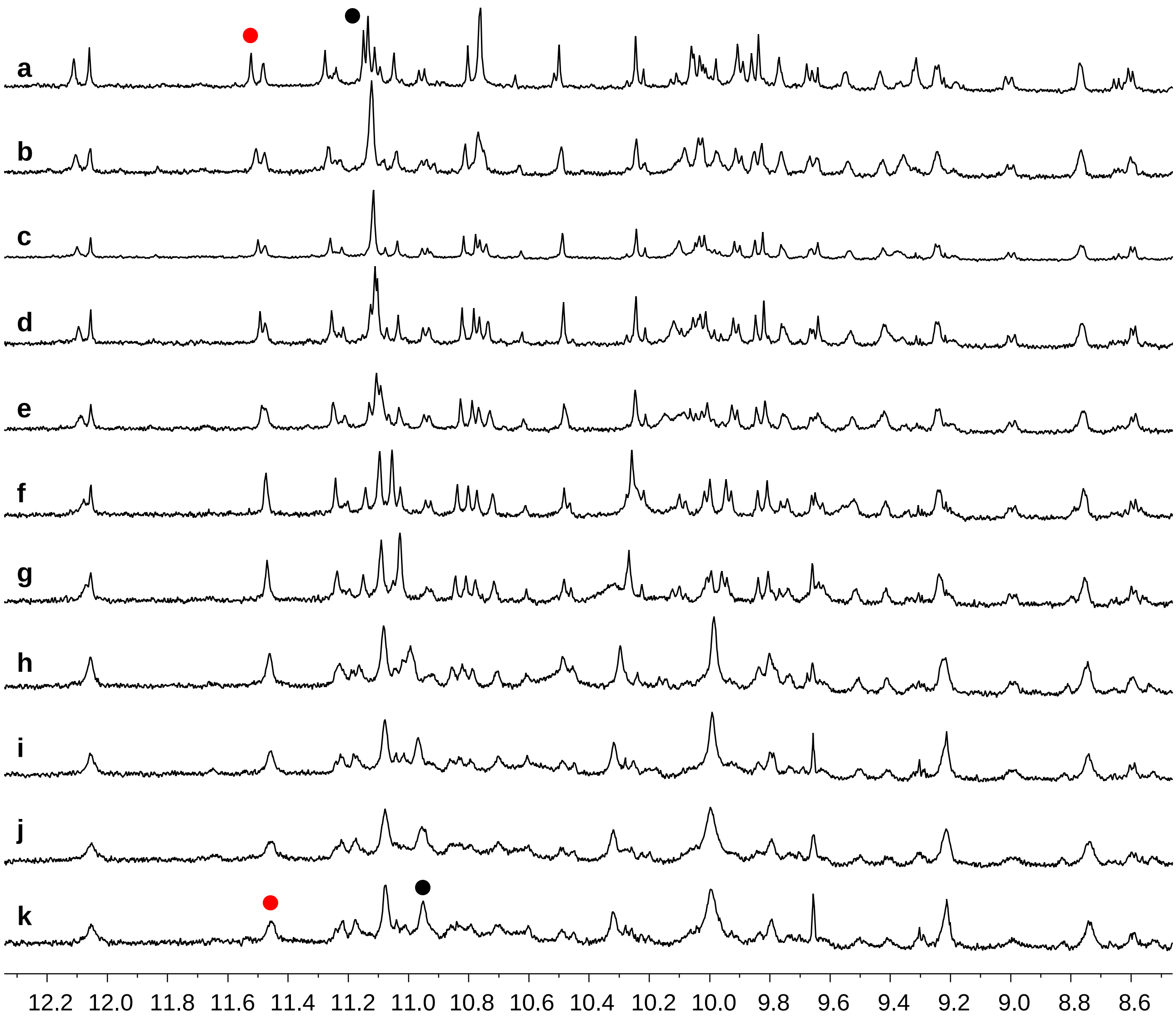

Figure S20 Part of the ^{1}H NMR spectra (500 MHz) showing amide resonances of **8b** in $CDCl_3$/DMSO-d_6. The volume percentages of DMSO-d_6 are 4 (a), 8 (b), 10 (c), 12 (d), 14 (e), 16 (f), 18 (g), 20 (h), 22 (i), 24 (j) and 26% (k), respectively. The chemical shift variations of two signals (marked with dots) are shown in Figure S22.

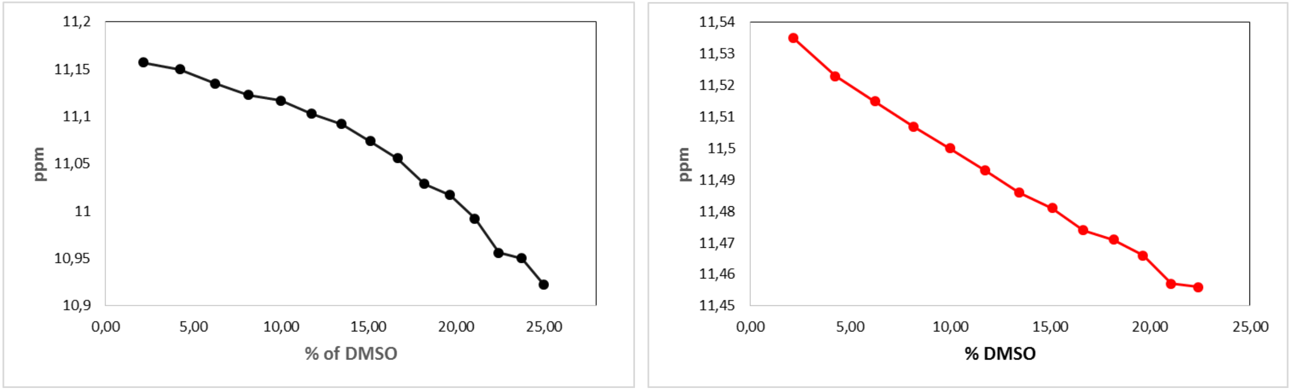

Figure S21 Variations of chemical shift of two amide NH protons of **8b** as a function of the volume percent of DMSO-d_6 in $CDCl_3$, (left major species, right minor species).

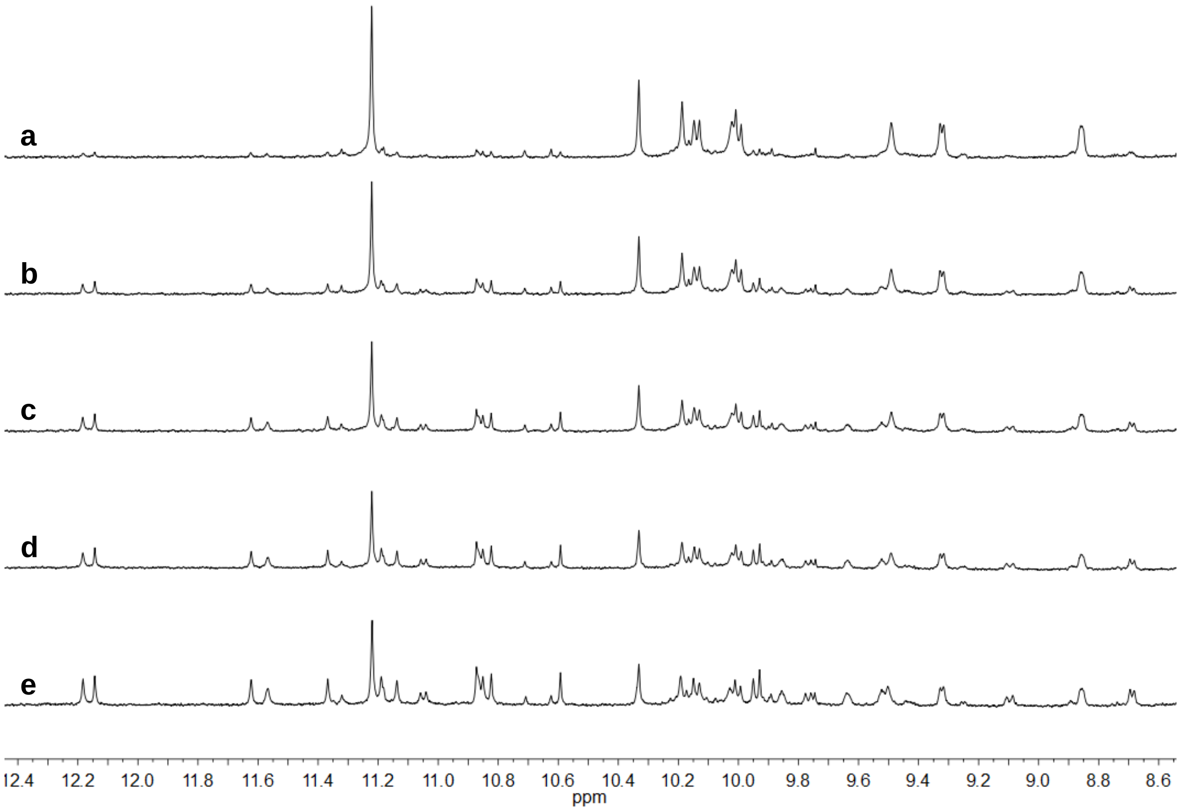

Figure S22 Evolution of the ^{1}H NMR spectrum (500 MHz) of freshly dissolved crystals of **8b** (grown from toluene/hexane) in $CDCl_3$ after 15 min (a), 2 h (b), 4 h (c), 8 h (d) and 24 h (e). This experiment suggests that the major species in solution is the same observed in solid state.

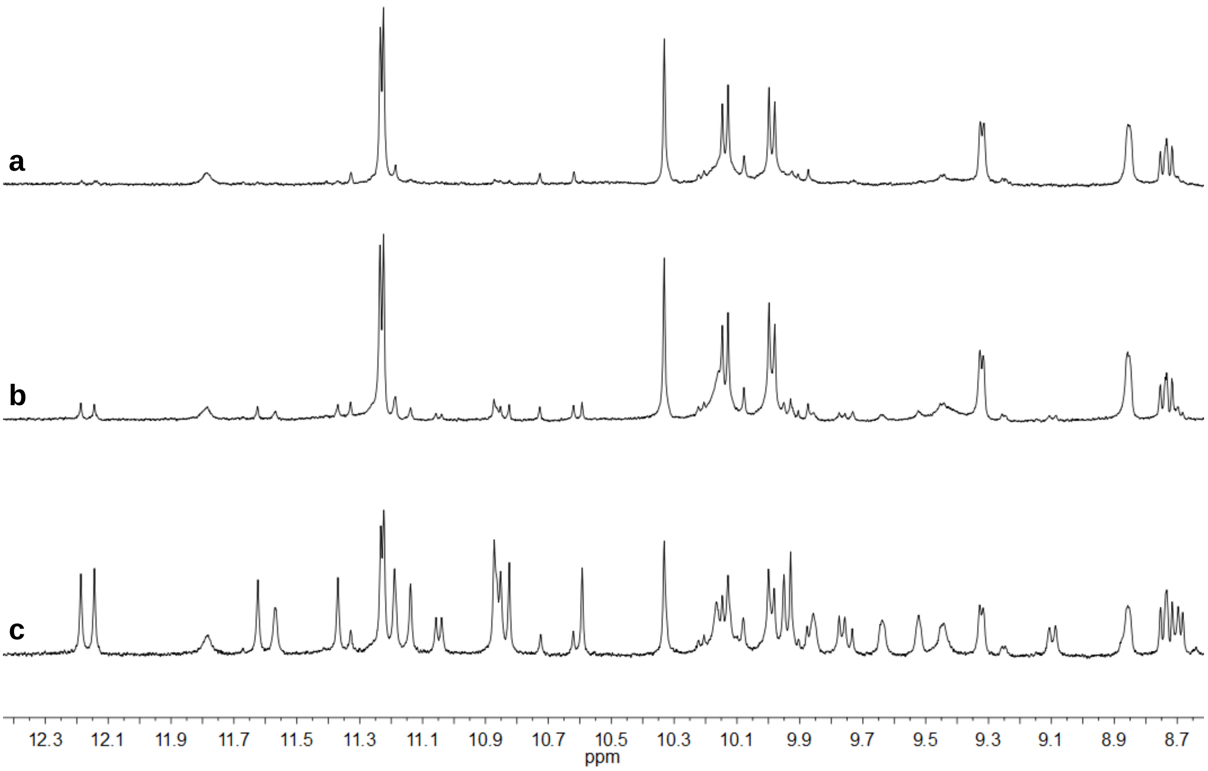

Figure S23 Evolution of the ^{1}H NMR spectrum (500 MHz) of a freshly dissolved freeze-dried sample of **8b** in $CDCl_3$ after 15 min (a), 1.5 h (b) and 24 h (c).

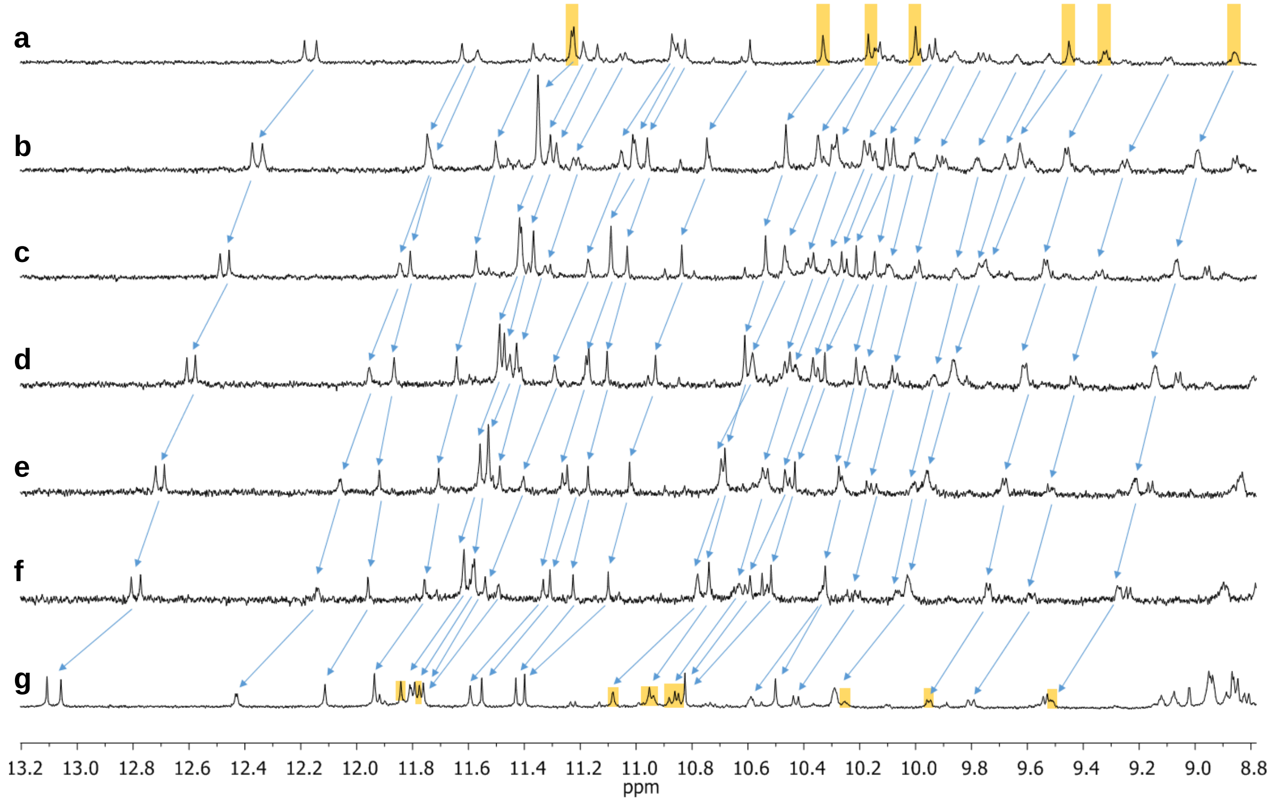

Figure S24 ^{1}H NMR spectra (500 MHz) of **8b** at equilibrium in $CDCl_3$ (a) and in toluene-d_8 (g), and in $CDCl_3$ with 10 % (b), 20% (c), 40% (d), 60% (e), 80% (f) of toluene-d_8. The signals corresponding to the folded species are highlighted in yellow.

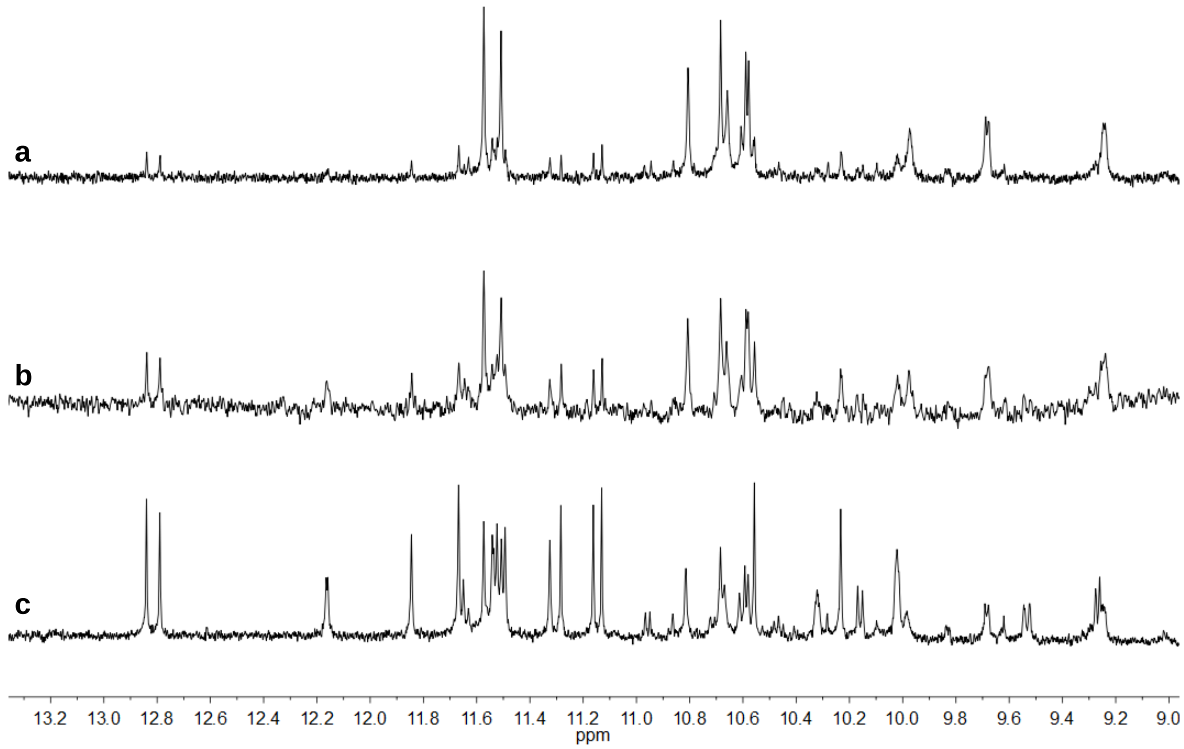

Figure S25 Evolution of the ^{1}H NMR spectrum (500 MHz) of freshly dissolved crystals of **8b** (grown from toluene/hexane) in toluene-d_8 after 15 min (a), 1 h (b) and 24 h (c). This experiment suggests that the major species in toluene-d_8 is not the same as in $CDCl_3$ and in solid state.

2. Molecular modelling

2.1 Molecular dynamic simulations

Molecular dynamic simulations were carried out using MacroModel version 11.1 (Schrödinger Inc.). Energy minimized structures, served as the starting point for the stochastic dynamic simulations, have been obtained using MMFFs force-field 1000 steps of Truncated Newton Conjugate Gradient (TNCG), no implicit solvent and the extended cutoff option. Stochastic dynamic simulations were obtained using MMFFs force field, $CHCl_3$ as solvent, extended cutoff and TNCG method.

The simulations were performed for 1 ns at different temperatures (300, 400, 500, 600 and 700 K), time step of 1.5 fs and 1 ps as equilibration time. Structures were sampled every 10 ps. H bond distances have been monitored during the simulation (H bonds highlighted in Fig. S26 and S27).

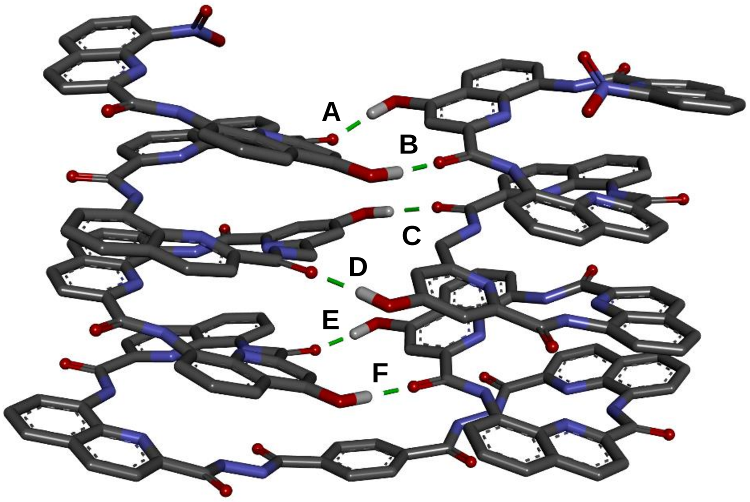

Figure S26 Energy minimized structure of **5b**. H bonds monitored during stochastic dynamics are highlighted.

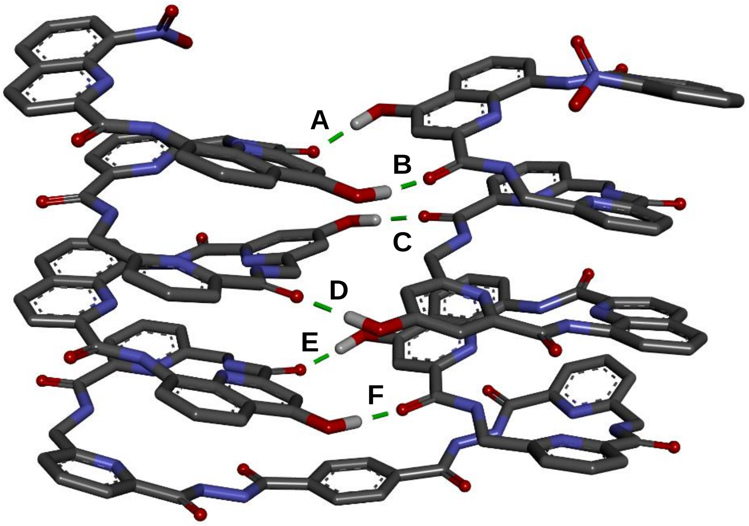

Figure S27 Energy minimized structure of **8b**. H bonds monitored during stochastic dynamics are highlighted.

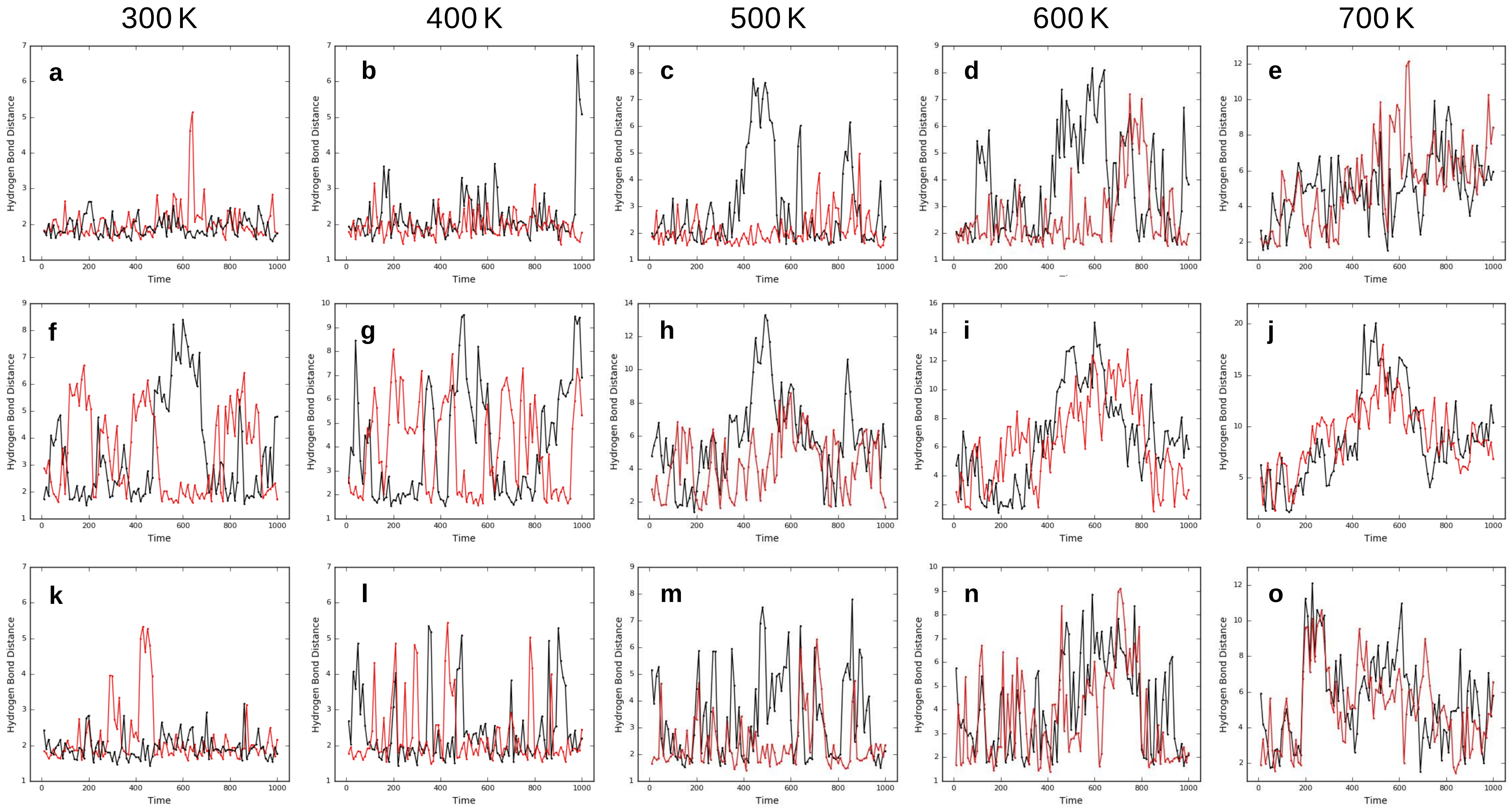

Figure S28 Time evolution of O…O hydrogen bond distances (Å) sampled every 10 ns during stochastic dynamic simulations for compound **5b**. H-bonds **A** (black) and **B** (red) at 300 K (**a**), 400 K (**b**), 500 K (**c**), 600 K (**d**) and 700 K (**e**). H-bonds **C** (red) and **D** (black) at 300 K (**f**), 400 K (**g**), 500 K (**h**), 600 K (**i**) and 700 K (**j**). H-bonds **E** (black) and **F** (red) at 300 K (**k**), 400 K (**l**), 500 K (**m**), 600 K (**n**) and 700 K (**o**). <u>Mind that the vertical scales are not all the same.</u>

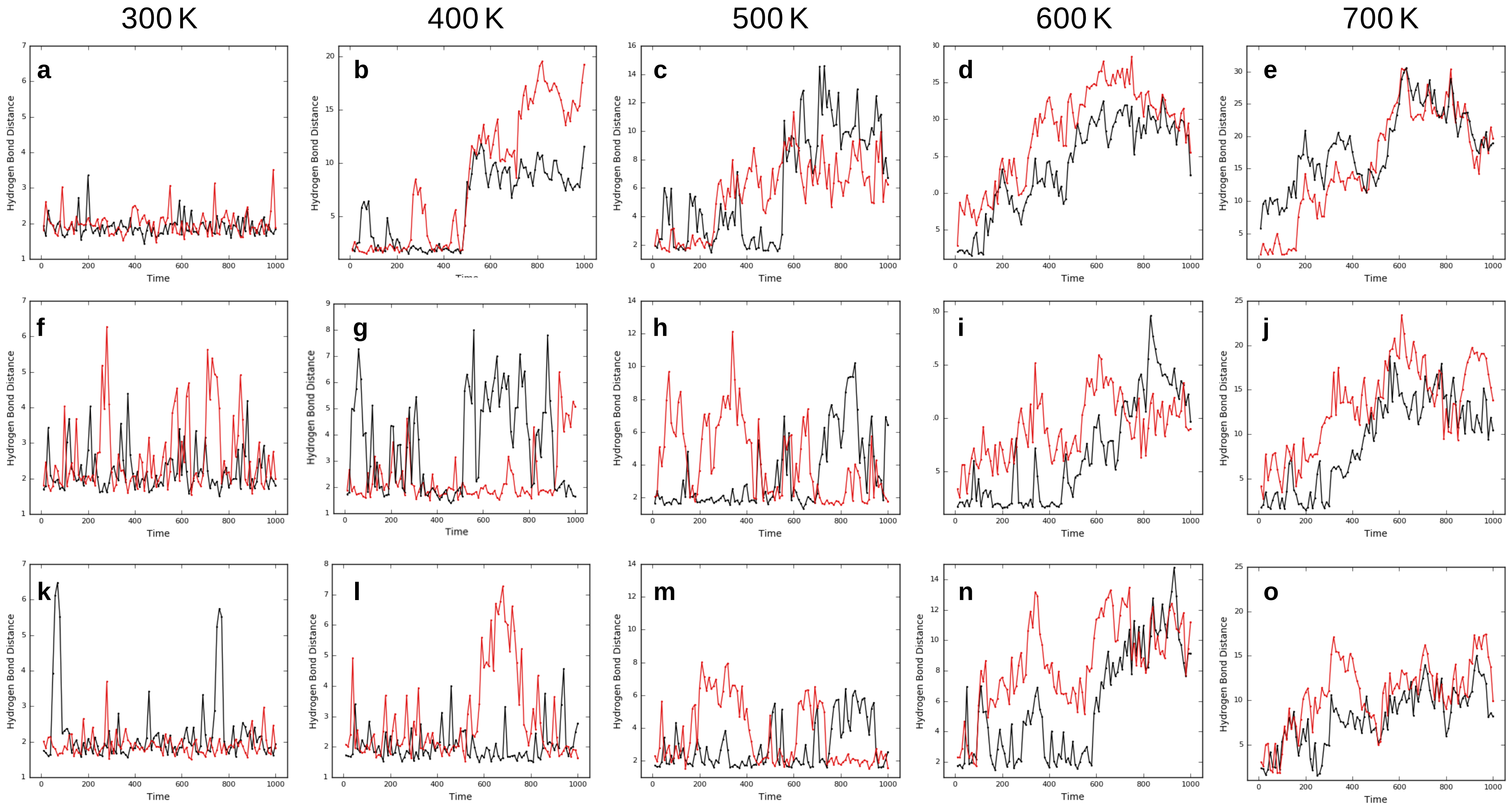

Figure S29 Time evolution of hydrogen bond distances sampled every 10 ns during stochastic dynamic simulations for compound **8b**. H-bonds **A** (black) and **B** (red) at 300 K (**a**), 400 K (**b**), 500 K (**c**), 600 K (**d**) and 700 K (**e**). H-bonds **C** (red) and **D** (black) at 300 K (**f**), 400 K (**g**), 500 K (**h**), 600 K (**i**) and 700 K (**j**). H-bonds **E** (black) and **F** (red) at 300 K (**k**), 400 K (**l**), 500 K (**m**), 600 K (**n**) and 700 K (**o**). <u>Mind that the vertical scales are not all the same.</u>

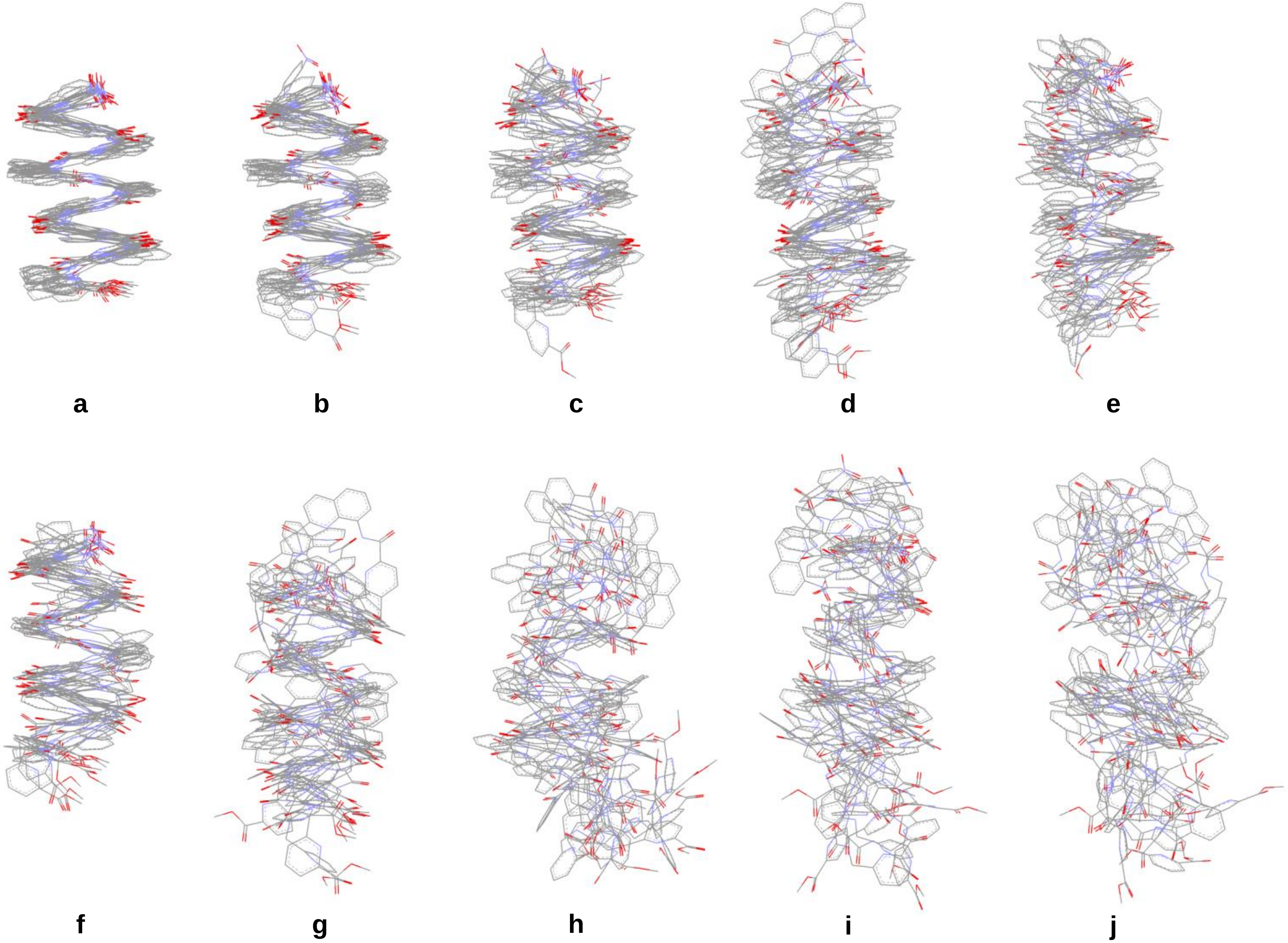

Figure S30 Stochastic dynamic simulations of **1** in $CHCl_3$ over 1 ns at 300 K (**a**), 400 K (**b**), 500 K (**c**), 600 K (**d**) and 700 K (**e**). Stochastic dynamic simulations of **4** in $CHCl_3$ over 1 ns at 300 K (**f**), 400 K (**g**), 500 K (**h**), 600 K (**i**) and 700 K (**j**). In each case, ten structures sampled every 100 ps are overlaid for each temperature. Side chains of **Q**, **P**, **X** and **Y** have been omitted for clarity.

2.2 Energy minimized models

Energy minimized structures, carried out using MacroModel version 11.1 (Schrödinger Inc.); have been obtained using MMFFs force-field 1000 steps of Truncated Newton Conjugate Gradient (TNCG), no implicit solvent and the extended cutoff option.

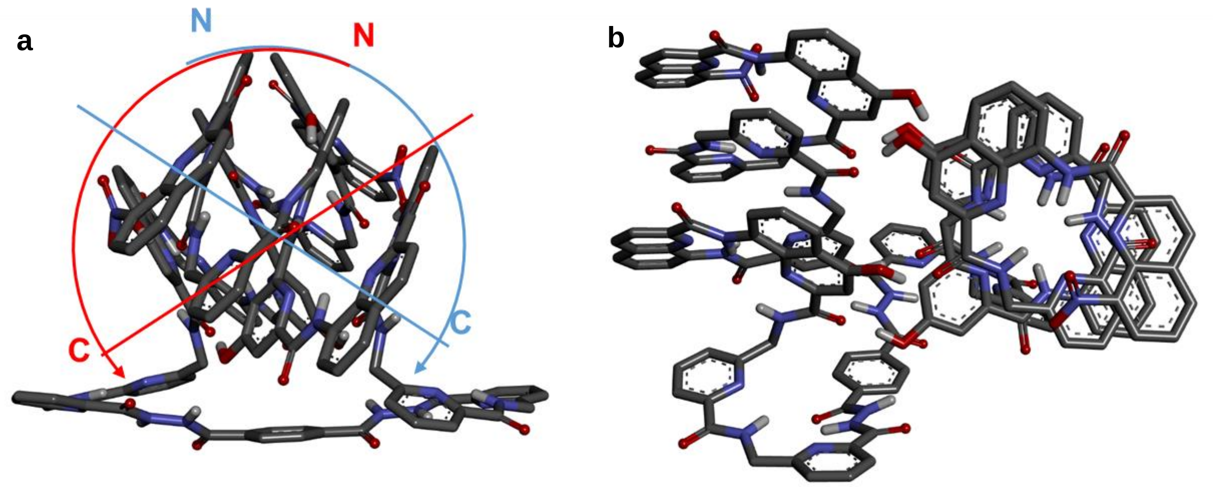

Figure S31 Different views (a,b) of an energy minimized model of compound **8b** folded in a tilted helix-turn-helix motif. The model corresponds to a right-handed 120° tilt with respect to the parallel helix-turn-helix motif. Side chains have been omitted for clarity.

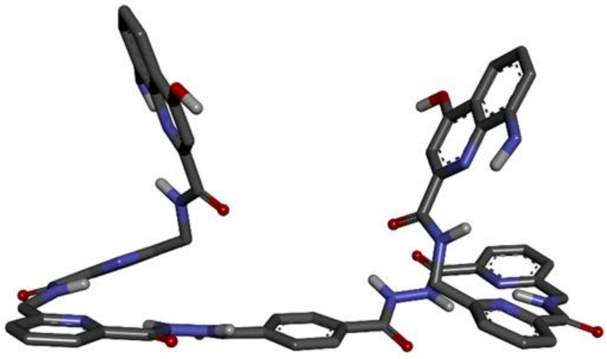

Figure S32 Particular of the model of **8b** showing **XPP-T-PPX** conformation. Side chains have been omitted for clarity.

3. Supplementary methods

3.1 Nuclear magnetic resonance spectroscopy

NMR spectra were recorded on different NMR spectrometers: (I) an Avance II NMR spectrometer (Bruker BioSpin) with a vertical 7.05 T narrow-bore/ultrashield magnet operating at 300 MHz for ^{1}H observation and 75 MHz for ^{13}C observation by means of a 5-mm direct BBO H/X probe with Z gradient capabilities; (II) an Avance III NMR spectrometer (Bruker BioSpin) with a vertical 16.45 T narrow-bore/ultrashield magnet operating at 700 MHz for ^{1}H observation by means of a 5-mm TXI ^{1}H/^{13}C/^{15}N probe with Z gradient capabilities. (III) an Avance III HD NMR spectrometer 400 MHz (Bruker BioSpin); (IV) an Avance III HD NMR spectrometer 500 MHz (Bruker BioSpin) with CryoProbe™ Prodigy.

Chemical shifts are described in part per million (ppm, δ) relative to the ^{1}H residual signal of the deuterated solvent used. ^{1}H NMR splitting patterns with observed first-order coupling are entitled as singlet (s), doublet (d), triplet (t), quartet (q) or broad singlet (bs). Coupling constants (J) are reported in hertz.

3.2 X-ray crystallography

X-ray diffraction measurements for **4**, **7b** and **8a** were carried out on a Rigaku FRX rotating anode (2.9 kW) diffractometer at the IECB x-ray facility (UMS 3033 – UMS001). CuKα radiation monochromated with high flux Osmic Varimax mirrors was used for data collections. The x-ray source is equipped with a Dectris Pilatus 200K detector and partial chi goniometer. All crystals were kept at 100(2) K during data collection. Data were processed with CrysAlis PRO[1] software. Structures were solved with the ShelXT[2] structure solution program using Intrinsic Phasing. Using Olex2[3] all structures were refined with the ShelXL[2] refinement package using Least Squares minimization. Crystals of **7b** were decompose easily in ambient atmosphere. Data collection was carried out despite diffraction images suggesting partial decomposition of the crystal. This resulted in low resolution and weak data quality. Thus, not all positions for C atoms of side chains were determined and only few solvent molecules were introduced into refinement. In the structure, 33% of unit cell volume (8784 Å^3) is not occupied as estimated with Mercury[4] using a probe radius of 1.2 Å. Only the main chain aromatic amide backbones and, in the case of **4** some side chains and solvent molecules, were refined with anisotropic displacement parameters. For the structure of **7b**, no H atoms were localized. For the structure of **8a**, H atoms were determined only for backbone positions. For the structure of **4**, all hydrogen atoms were determined except some side chains positions. All H atoms were

refined in the riding-model approximation, with Uiso(H)=1.2Ueq(CH, CH2, NH) and Uiso(H)=1.5Ueq(CH_3).

DFIX, AFIX, SIMU, FLAT, EADP, RIGU and DELU instructions were employed to model geometry of the molecules and temperature parameters.

Refinement of large foldamer crystal structures faces problems usually observed in macromolecular crystallography, i.e. large volume fractions of disordered solvent molecules, weak diffraction intensity, incompleteness of the data, moderate or low resolution. Thus, it is not suprising that a number of A-level and B-level alerts were detected using IUCR's checkcif algorithm. These alerts are inherent to the data and refinement procedures and do not reflect errors. Rather, they illustrate the limited practicality of the checkcif tool for medium size molecule crystallography. They are listed below and have been divided into two groups.

Group 1 alerts illustrate weak quality of the data and refinement statistics if compared to that expected for small molecule structures from highly diffracting crystals:

THETM01_ALERT_3_A The value of sine(theta_max)/wavelength is less than 0.550

THETM01_ALERT_3_B The value of sine(theta_max)/wavelength is less than 0.575

PLAT934_ALERT_3_A Number of (Iobs-Icalc)/SigmaW > 10 Outliers

PLAT023_ALERT_3_A Resolution (too) Low [sin(theta)/Lambda < 0.6].

PLAT082_ALERT_2_A High R1 Value

PLAT084_ALERT_3_A High wR2 Value (i.e. > 0.25)

PLAT084_ALERT_3_B High wR2 Value (i.e. > 0.25)

PLAT220_ALERT_2_B Non-Solvent Resd 1 C Ueq(max)/Ueq(min) Range 8.6 Ratio

PLAT241_ALERT_2_B High 'MainMol' Ueq as Compared to Neighbors

PLAT242_ALERT_2_B Low 'MainMol' Ueq as Compared to Neighbors of

PLAT340_ALERT_3_B Low Bond Precision on C-C Bonds .... 0.0112 Ang.

Group 2 alerts are connected to decisions made during refinement and explained below:

PLAT201_ALERT_2_A Isotropic non-H Atoms in Main Residue(s)

PLAT202_ALERT_3_A Isotropic non-H Atoms in Anion/Solvent

As mentioned above not all atoms were refined with ADP

PLAT043_ALERT_1_A Calculated and Reported Mol. Weight Differ by .. 1137.39 Check

PLAT044_ALERT_1_A Calculated and Reported Density Dx Differ by .. 0.1422 Check

PLAT043_ALERT_1_B Calculated and Reported Mol. Weight Differ by

Not all atoms of foldamer molecules were determined but they were used in SFAC calculation

PLAT602_ALERT_2_A VERY LARGE Solvent Accessible VOID(S) in Structure!

It was not possible to determined severely disordered solvent molecules

PLAT430_ALERT_2_A Short Inter D...A Contact

PLAT430_ALERT_2_B Short Inter D...A Contact

These contacts are connected with dummy O atoms introduced into refinement or with O-H groups for which no H-atoms were detected

PLAT306_ALERT_2_B Isolated Oxygen Atom (H-atoms Missing?)

Dummy O atoms were introduced into refinement

PLAT315_ALERT_2_B Singly Bonded Carbon Detected (H-atoms Missing)

Not all H atoms were introduced into refinement

PLAT049_ALERT_1_B Calculated Density Less Than 1.0 gcm-3

Alert concerns structure **7b** and it is explained above

Table S1 O···O distances in crystal structure of **7b**. Atom numbers are those of the cif file.

$O_{(H)}\cdots O_{(amide)}$	**distance (Å)**	$O_{(H)}\cdots O_{(amide)}$	**distance (Å)**
O7D···O16C	2.77(3)	O7H···O16G	2.47(4)
O14D O9C	2.68(3)	O14H···O9G	2.69(3)
O16D···O6C	2.87(3)	O16H···O6G	2.68(3)
O5D···O19C	2.75(2)	O5H···O1W	2.56(4)
		O1W···O19G	2.47(4)
O2D···O21C	2.63(3)	O2H···O21G	2.28(3)
O19D···O4C	2.63(3)	O19H···O4G	2.94(5)

**Identification code**	**4**	**7b**	**8a**
**Chemical formula**	$2(C_{121}H_{148}N_{18}O_{21}Si)\cdot C_6H_{14}\cdot 4.78(CHCl_3)\cdot 4(O)$*	$2(C_{234}H_{242}N_{40}O_{44})\cdot CHCl_3\cdot 5(O)$*	$C_{256}H_{314}N_{40}O_{44}Si_2\cdot 13(CH_3OH)\cdot 2.98(H_2O)$
**Formula weight**	5157.71	8836.69	4947.07
**Temperature**	100(2)	100(2)	100(2)
**Wavelength**	1.54178 Å	1.54178 Å	1.54178 Å
**Crystal system**	Triclinic	Triclinic	Monoclinic
**Space group**	*P*-1	*P*-1	$P2_1/n$
**Unit cell dimensions**	a=17.7180 (6), α=98.564 (2)<br>b=18.1791 (5), β=99.814 (2)<br>c=22.9828 (6), γ=102.609 (3)	a=21.4823 (11), α=84.990 (3)<br>b=26.9299 (9), β=82.990 (3)<br>c=48.1304 (14), γ=74.494 (4)	a=16.1473 (3), α=90<br>b=36.4163 (7), β=106.983 (2)<br>c=26.2800 (5), γ=90
**Volume**	6983.8 (4)	26586.5 (19)	14779.4 (5)
Z	1	2	2
**Density (calculated)**	1.226	1.104	1.112
**Absorption coefficient**	1.98	0.78	0.71
**Absorption correction**	Multi-scan	Multi-scan	Multi-scan
**Crystal size**	0.30 × 0.07 × 0.03	0.10 × 0.05 × 0.02	0.20 × 0.07 × 0.05
**Index ranges**	*h* = -21→21<br>*k* = -20→22<br>*l* = -28→28	*h* = -19→19<br>*k* = -24→24<br>*l* = -43→43	*h* = −18→17<br>*k* = −39→41<br>*l* = −29→29
**Completeness to theta = 67.68º**	98.3	98.5	98.8
**Reflections collected**	79828	41234	89253
**Reflections observed [*I* > 2σ(*I*)]**	17895	19491	15000
R_{int}	0.062	0.062	0.033
**Data/parameters/restrains**	26994/1439/47	41234/3593/3876	22382/1247/113
**Goodness-of-fit on F^2**	2.13	2.59	2.71
**Final R indices [*I* > 2σ(*I*)]**	R1 = 0.1523, wR2 = 0.3751	R1 = 0.3030, wR2 = 0.6461	R1 = 0.2411, wR2 = 0.6025
**R indices (all data)**	R1 = 0.1799, wR2 = 0.3988	R1 = 0.3716, wR2 = 0.6841	R1 = 0.2658, wR2 = 0.6246
**Lasgest diff. peak and hole**	1.41, -0.94	1.18, -0.69	1.32, -0.82
**CCDC #**	1901969	1901970	1901971

*Unrecognized electron density was introduced to the refinement as dummy oxygen atoms

4. Synthetic schemes

Scheme S1 Synthesis of **16**

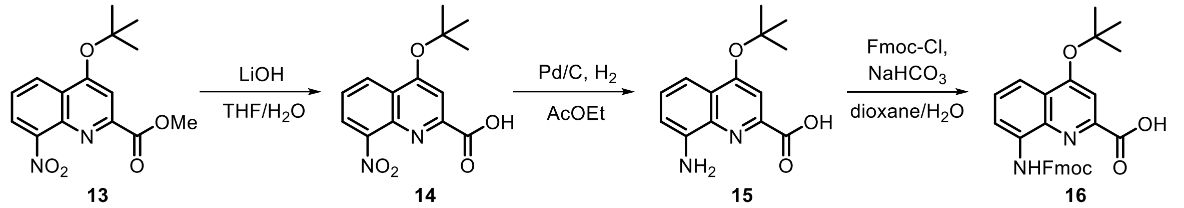

Scheme S2 Synthesis of **24**

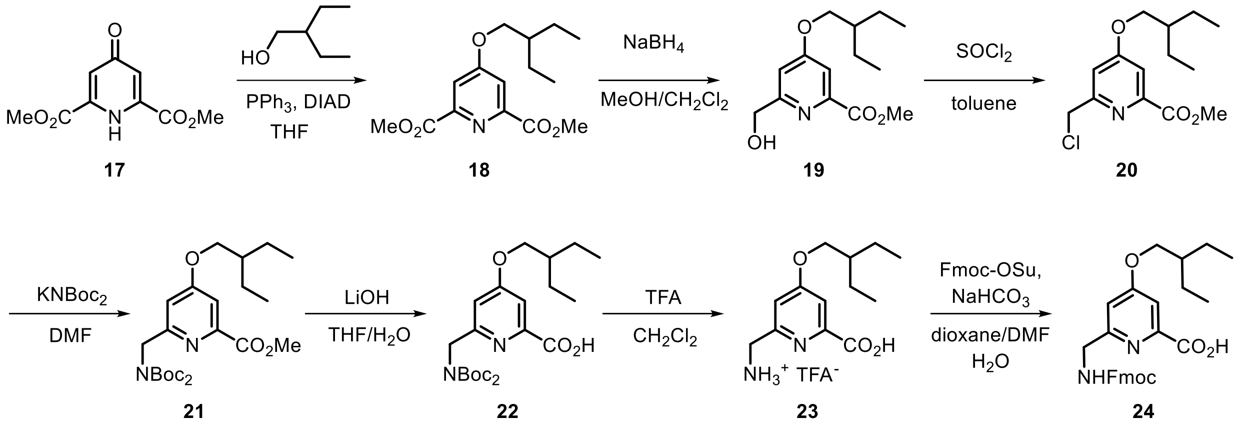

Scheme S3 Synthesis of **31**

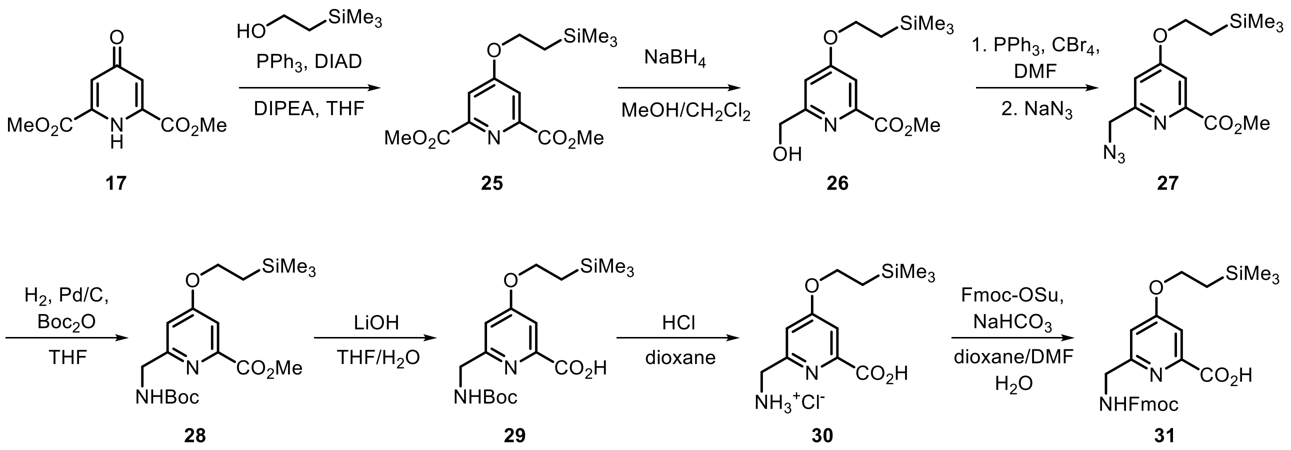

Scheme S4 Synthesis of **34**

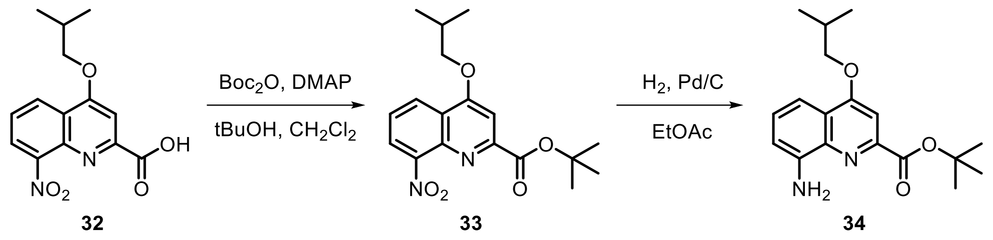

Scheme S5 Synthesis of **36**

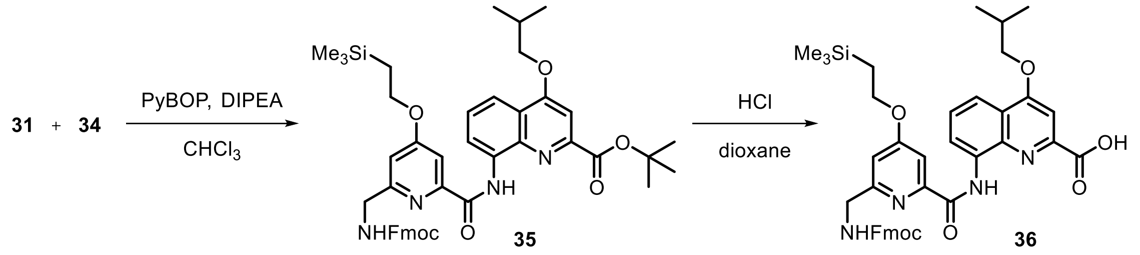

Scheme S6 Synthesis of **38**

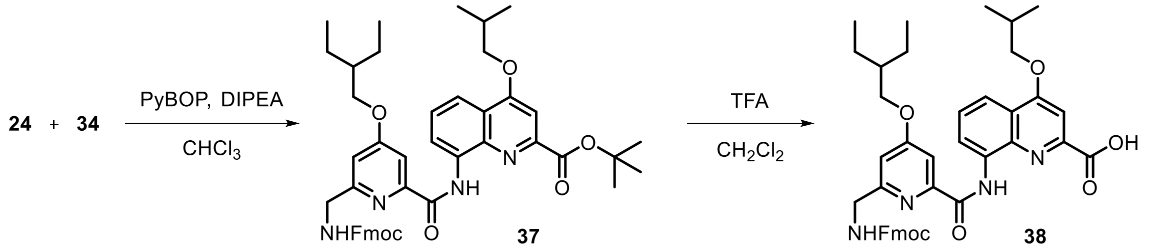

Scheme S7 Synthesis of **1**

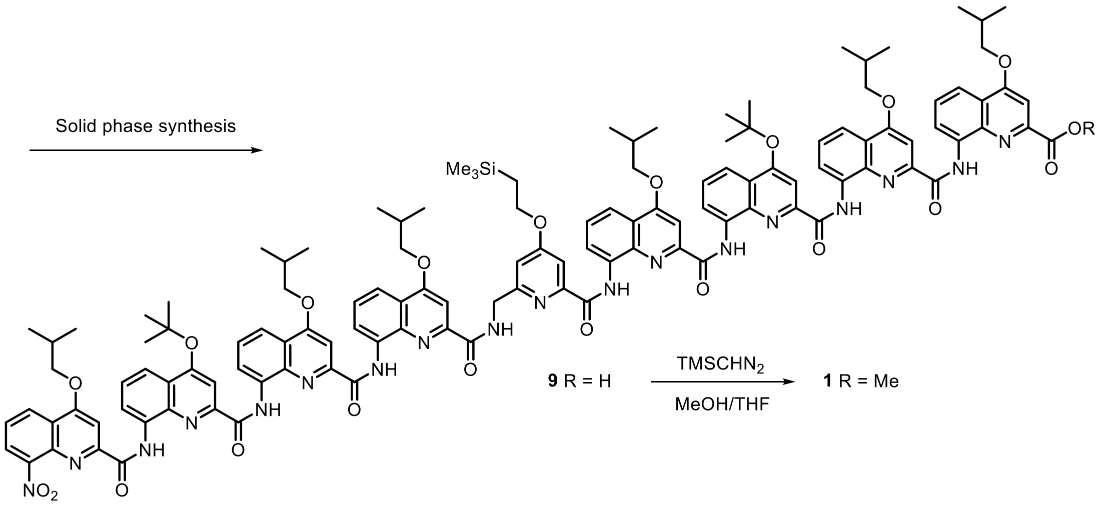

Scheme S8 Synthesis of **2**

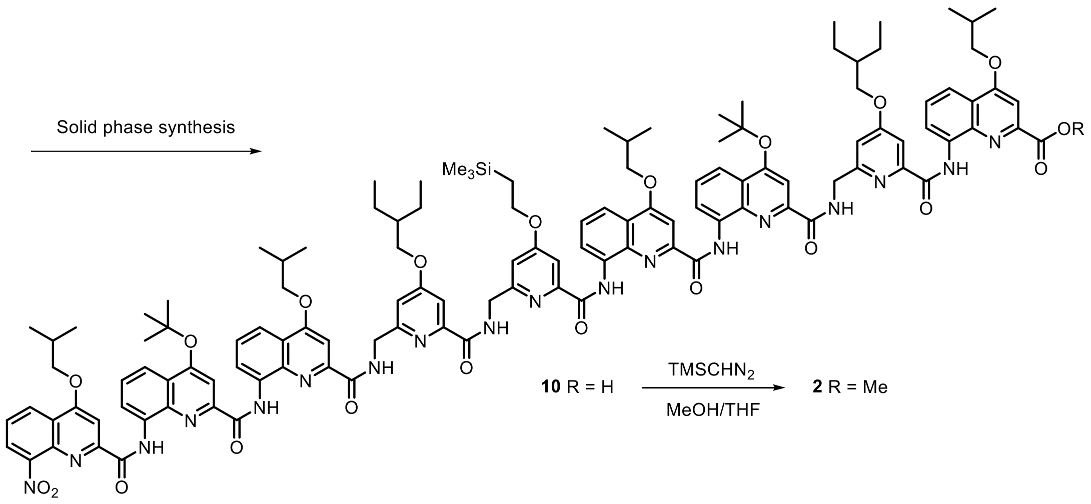

Scheme S9 Synthesis of **3**

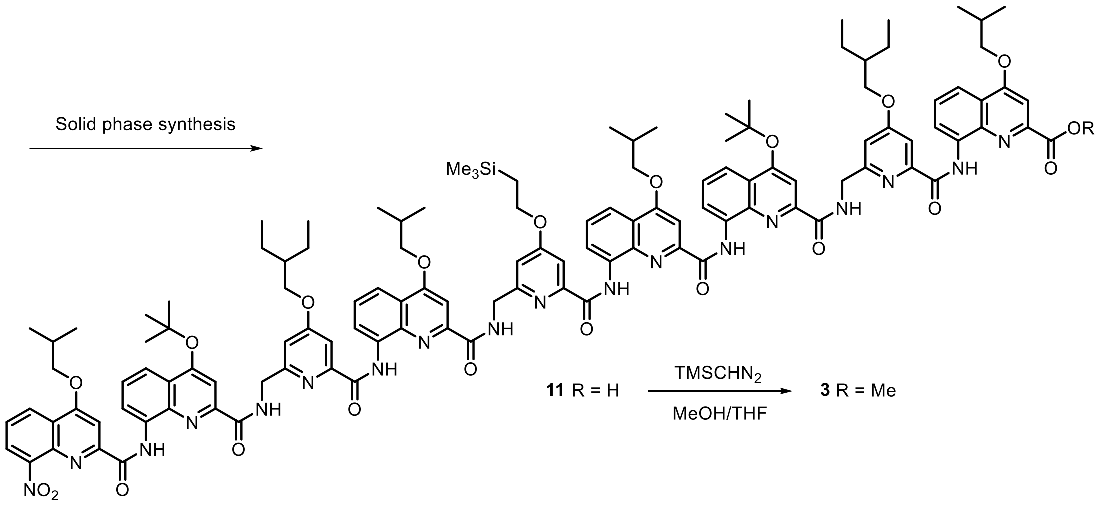

Scheme S10 Synthesis of **4**

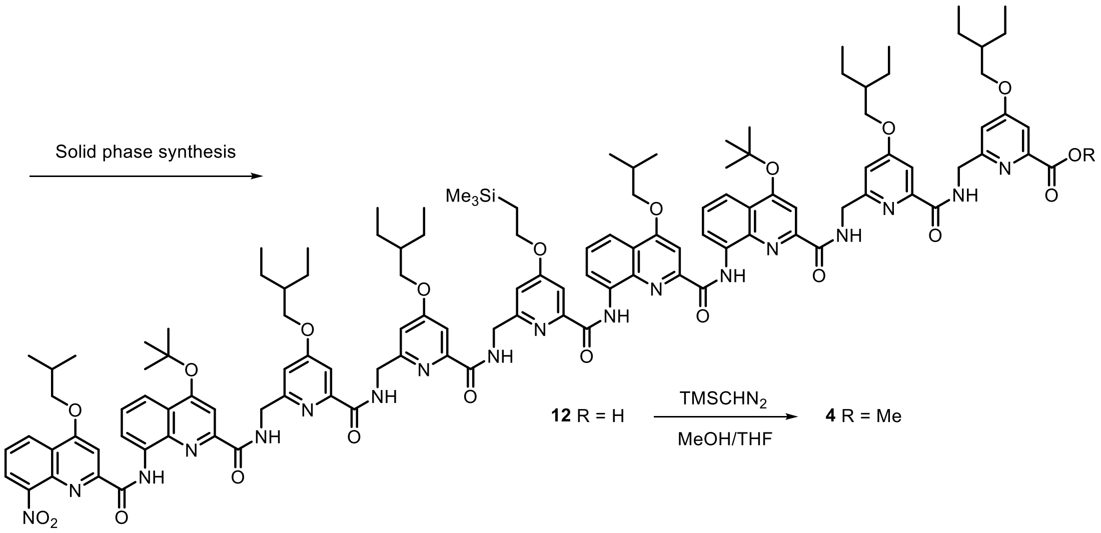

Scheme S11 Synthesis of **5b**

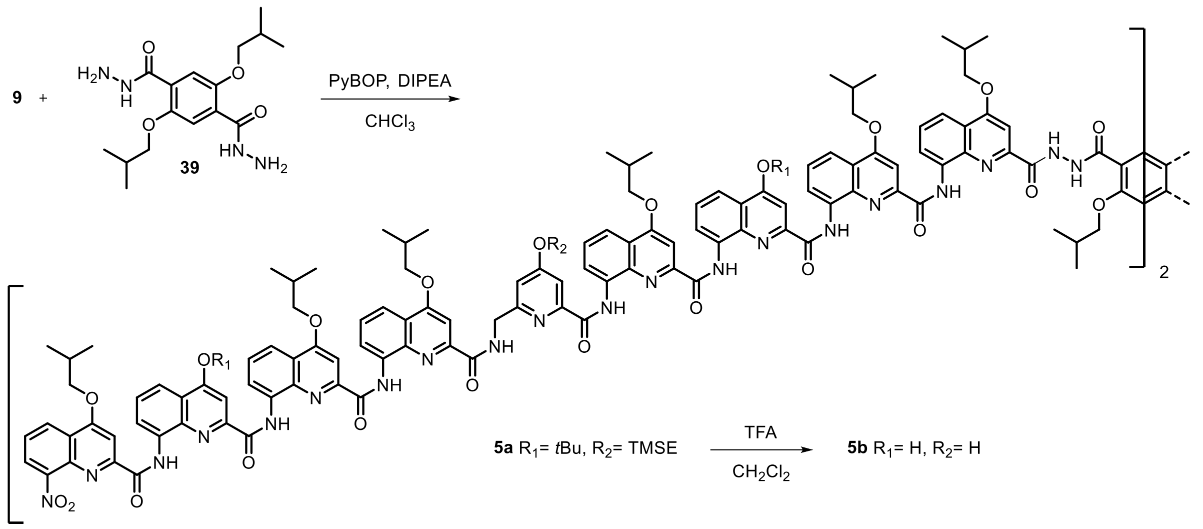

Scheme S12 Synthesis of **6b**

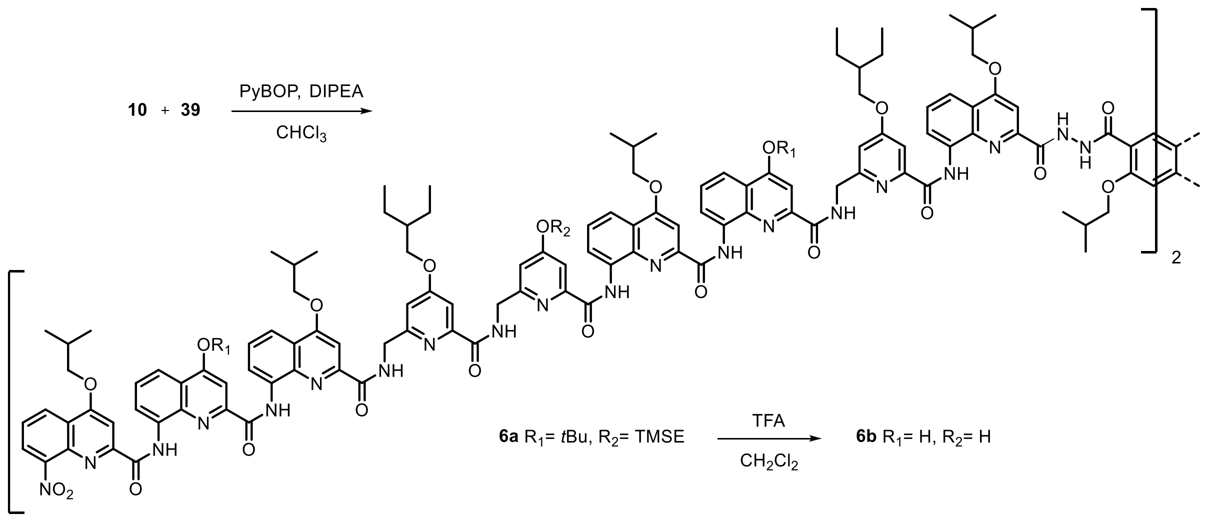

Scheme S13 Synthesis of **7b**

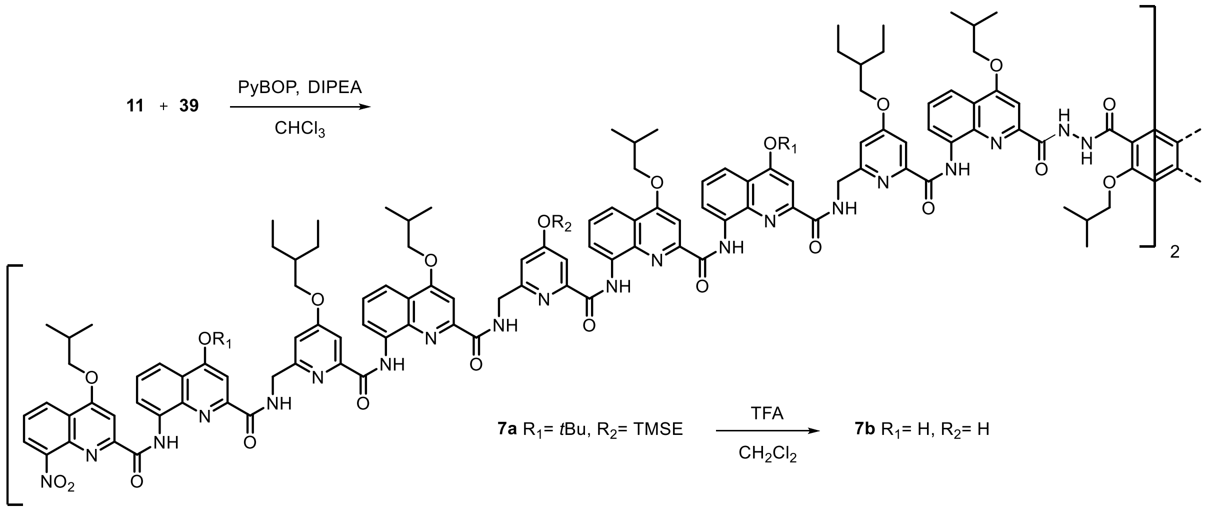

Scheme S14 Synthesis of **8b**

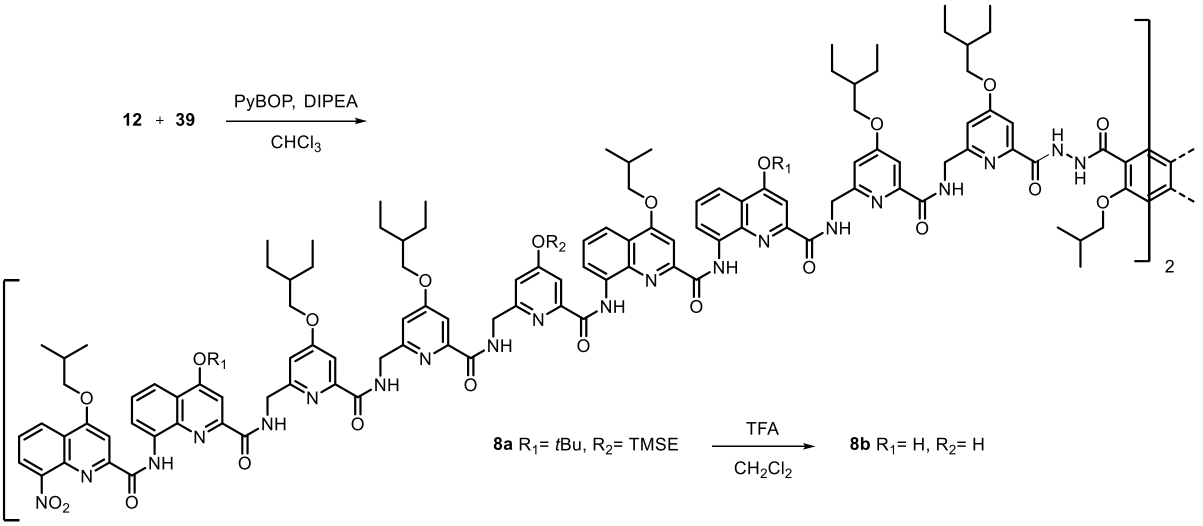

5. Experimental procedures

5.1 General methods

Commercial reagents were purchased from Sigma-Aldrich, Alfa-Aesar or TCI and were used without further purification unless otherwise specified. SASRIN resin (100-200 mesh, loading 0.7-1.0 mmol/g) was purchased from Bachem. Tetrahydrofuran (THF), dichloromethane (DCM) and toluene were dried over alumina columns (MBRAUN SPS-800 solvent purification system); chloroform and diisopropylethylamine (DIPEA) were distilled over calcium hydride (CaH_2) prior to use. Reactions were monitored by thin layer chromatography (TLC) on Merck silica gel 60-F254 plates and observed under UV light. Column chromatography purifications were carried out on Merck GEDURAN Si60 (40-63 µm). Preparative recycling GPC (gel permeation chromatography) was carried out on JAIGEL 20*600 mm columns (Japan Analytical Industry) in chloroform containing 1% ethanol and 0.5% triethylamine as mobile phase, with a flow rate of 7.5 mL/min. Monitoring by UV detection was carried out at 254 nm, 280 nm, 300 nm and 360 nm.

Analytical RP-HPLC analyses were performed on an Ultimate 3000 HPLC System (ThermoFisher Scientific) using a Nucleodur C_8 Gravity column (4 x 100 mm, 5 µm, Macherey-Nagel). The mobile phase was composed of H_2O (solvent A) and CH_3CN (solvent B). Semipreparative purifications of oligomers were performed on an Ultimate 3000 HPLC System (ThermoFisher Scientific) using a Nucleodur C_8 Gravity column (10 x 125 mm, 5 µm, Macherey-Nagel).

Solid phase synthesis (SPS) was performed manually under microwave irradiation on a CEM Discover (Liberty Bio) microwave oven using open reaction vessel and an internal fiber optic probe for temperature control.

High-resolution electrospray mass spectra were recorded on a Thermo Exactive orbitrap instrument.

5.2 Synthesis of small molecules

4-(*tert*-butoxy)-8-nitroquinoline-2-carboxylic acid (**14**) Methyl 4-(*tert*-butoxy)-8-nitroquinoline-2-carboxylate (**13**)[5] (1.4 g, 4.6 mmol, 1 equiv.) was dissolved in THF/water 5:1 (120 mL) and $LiOH·H_2O$ (290 mg, 6.9 mmol, 1.5 equiv.) was added. The solution was stirred at r.t. for 1 h (complete conversion from TLC). The solution was diluted with water and 5% aqueous citric acid was added until pH 5-6. The compound was extracted with CH_2Cl_2 (3v). The organic layer was washed with water, dried over $MgSO_4$ and filtered. After evaporation, the compound was recovered as a yellow solid (1.27 g, 95% yield).

^{1}H NMR (300 MHz, $CDCl_3$) δ 8.50 (dd, *J* = 8.5, 1.4 Hz, 1H), 8.19 (dt, *J* = 10.9, 5.5 Hz, 1H), 7.87 (s, 1H), 7.68 (dd, *J* = 8.5, 7.6 Hz, 1H), 1.74 (s, 9H). ^{13}C NMR (126 MHz, $CDCl_3$) δ 163.88, 162.28, 148.82, 147.32, 138.82, 128.00, 126.31, 126.14, 125.78, 103.89, 83.82, 28.56. MS calcd for $C_{14}H_{13}N_2O_5$ $[M-H]^-$ 289.0830, found (HR-ESI) 289.0832.

8-amino-4-(*tert*-butoxy)quinoline-2-carboxylic acid (**15**) 4-(*tert*-butoxy)-8-nitroquinoline-2-carboxylic acid (**14**) (1.1 g, 3.8 mmol) was dissolved in EtOAc (250 mL) under N_2 atmosphere. Then Pd/C catalyst (120 mg, 10% by mass) was added and N_2 exchanged with H_2. The reaction mixture was stirred overnight at r.t. under H_2 atmosphere. The resulting mixture was filtered over celite and evaporated under reduced pressure to yield the compound as an orange solid (0.97 g, 98% yield).

^{1}H NMR (400 MHz, DMSO-d_6) δ 12.77 (s, 1H), 7.57 (s, 1H), 7.37 – 7.29 (m, 1H), 7.21 (dd, *J* = 8.3, 1.3 Hz, 1H), 6.85 (dd, *J* = 7.6, 1.3 Hz, 1H), 6.48 (s, 2H), 1.59 (s, 9H). ^{13}C NMR (126 MHz, DMSO-d_6) δ 165.85, 159.95, 146.51, 143.72, 136.86, 129.24, 125.16, 109.40, 107.32, 104.50, 81.09, 28.21. MS calcd for $C_{14}H_{17}N_2O_3$ $[M+H]^+$ 261.1234, found (HR-ESI) 261.1233.

8-((((9H-fluoren-9-yl)methoxy)carbonyl)amino)-4-(*tert*-butoxy)quinoline-2-carboxylic acid (**16**) 8-amino-4-(*tert*-butoxy)quinoline-2-carboxylic acid (**15**) (970 mg, 3.7 mmol, 1 equiv.) was dissolved in dioxane (100 mL) and a 10% *w/v* $NaHCO_3$ solution (30 mL) was added. The resulting slurry was cooled to 0 °C and a solution of Fmoc-Cl (1.06 g, 4.1 mmol, 1.1 equiv) in 50 mL dioxane was added dropwise over 1 h. The reaction mixture was stirred at r. t. overnight. The resulting mixture was diluted with water and pH was brought to 5-6 by dropwise addition of 5% citric acid solution. The compound was extracted with CH_2Cl_2 (3v) and the organic layer was washed with water. Then dried over $MgSO_4$, filtered and concentrated. The crude product was purified by chromatography increasing solvent polarity

from CH_2Cl_2 to CH_2Cl_2/EtOAc 1:1. After recrystallization from CH_2Cl_2/cyclohexane, the compound was obtained as a light yellow solid (1 g, 56%).

^{1}H NMR (500 MHz, DMSO-d_6) δ 13.52 (s, 1H), 10.42 (s, 1H), 8.33 (s br, 1H), 7.93 (d, *J* = 7.5 Hz, 2H), 7.80-7.77 (m, 3H), 7.71 (s, 1H), 7.63 – 7.55 (m, 1H), 7.44 (t, *J* = 7.4 Hz, 2H), 7.36 (t, *J* = 7.4 Hz, 2H), 4.61 (d, *J* = 6.4 Hz, 1H), 4.45 (t, *J* = 6.7 Hz, 1H), 1.63 (s, 9H). ^{13}C NMR (75 MHz, DMSO-d_6) δ 166.24, 160.88, 153.94, 146.47, 144.01, 141.16, 138.37, 135.89, 128.68, 128.23, 127.63, 125.50, 124.61, 120.59, 116.84, 115.90, 105.44, 82.49, 66.67, 46.95, 28.42. MS calcd for $C_{29}H_{27}N_2O_5$ [M+H]$^+$ 483.1914, found (HR-ESI) 483.1914.

Dimethyl 4-(2-ethylbutoxy)pyridine-2,6-dicarboxylate (**18**) Dimethyl 4-hydroxy-2,6-pyridinedicarboxylate (**17**)[6] (2 g, 9.5 mmol, 1.0 equiv.) and triphenylphosphine (PPh_3) (2.73 g, 10.4 mmol, 1.1 equiv.) were dissolved in dry THF (100 mL) under N_2. Then 2-ethyl-1-butanol (1.28 mL, 10.4 mmol, 1.1 equiv.) was added and the reaction mixture was cooled to 0 °C. Diisopropylazodicarboxylate (DIAD) (2 mL, 10.4 mmol, 1.1 equiv.) was slowly added dropwise. The reaction mixture was stirred at r.t. overnight. The solvent was evaporated, the residue was triturated in hexane and filtered. The filtrated was concentrated under vacuum and the residue purified by flash chromatography (1:1 cyclohexane/Et_2O). The product was obtained as a white solid (2.66 g, 95% yield).

^{1}H NMR (300 MHz, $CDCl_3$) δ 7.81 (s, 2H), 4.03 (d, *J* = 5.7 Hz), 4.01 (s, 6H), 1.73 (m, 1H), 1.49 (m, 4H), 0.94 (t, *J* = 7.5 Hz, 6H). ^{13}C NMR (75 MHz, $CDCl_3$) δ 167.47, 165.41, 149.87, 114.70, 71.31, 53.36, 40.74, 23.38, 11.21. MS calcd for $C_{15}H_{22}NO_5$ [M+H]$^+$ 296.1492, found (HR-ESI) 296.1485.

Methyl 6-hydroxymethyl-4-(2-ethylbutoxy)-2-pyridine carboxylate (**19**) Dimethyl 4-(2-ethylbutoxy)pyridine-2,6-dicarboxylate (**18**) (2.5 g, 8.5 mmol, 1 equiv.) was dissolved in 1:2 CH_2Cl_2/MeOH solution (75 mL). After cooling to 0 °C, $NaBH_4$ (640 mg, 16.9 mmol, 2 equiv.) was added under a N_2 atmosphere. The reaction mixture was stirred at 0°C for 30 min then at r. t. for 1 h (from TLC complete conversion). The reaction mixture was neutralized with 5% citric acid solution and extracted with CH_2Cl_2 (3v). The organic phase was washed with water and dried with $MgSO_4$, filtered, and evaporated. The crude product was purified by chromatography using CH_2Cl_2/MeOH 98:2 to provide a white solid (1.85 g, 82 % yield).

^{1}H NMR (300 MHz, $CDCl_3$) δ 7.56 (d, *J* = 2.4 Hz, 1H), 7.01 (d, *J* = 2.3 Hz, 1H), 4.79 (d, *J* = 4.9 Hz, 2H), 3.98 (s, 3H), 3.97 (d, *J* = 5.7 Hz, 2H), 3.25 (t, *J* = 5.4 Hz, 1H), 1.76-1.65 (m, 1H), 1.54-1.39 (m, 4H), 0.93 (t, *J* = 7.5 Hz, 6H). ^{13}C NMR (75 MHz, CDCl3) δ 167.06, 165.81,

162.10, 148.63, 111.25, 109.74, 70.74, 64.88, 53.05, 40.72, 23.36, 11.19. MS calcd for $C_{14}H_{22}NO_4$ $[M+H]^+$ 268.1543, found (HR-ESI) 268.1539.

Methyl 6-chloromethyl-4-(2-ethylbutoxy)-2-pyridine carboxylate (**20**) To a solution of **19** (2 g, 7.5 mmol, 1 equiv.) in dry toluene (20 mL) under N_2 atmosphere $SOCl_2$ (2.45 mL, 33.8 mmol, 4.5 equiv.) was added. The reaction mixture was stirred at r.t. for 3 h. The solvents were removed under reduced pressure to yield the product as an oil (2.14 g, quant. yield).
^{1}H NMR (300 MHz, $CDCl_3$) δ 7.61 (d, *J* = 2.4 Hz, 1H), 7.22 (d, *J* = 2.4 Hz, 1H), 4.74 (s, 2H), 4.00 (s, 3H), 3.99 (d, *J* = 5.2 Hz, 2H), 1.77-1.63 (m, 1H), 1.55-1.42 (m, 4H), 0.94 (t, *J* = 7.5 Hz, 6H). ^{13}C NMR (75 MHz, $CDCl_3$) δ 167.51, 165.44, 158.69, 148.95, 112.48, 111.52, 71.01, 53.30, 46.31, 40.77, 23.39, 11.22. MS calcd for $C_{14}H_{21}ClNO_3$ $[M+H]^+$ 286.1204, found (HR-ESI) 286.1211.

Compound 21 To a suspension of **20** (2.14 g, 7.5 mmol, 1 equiv.) in dry DMF (20 mL) potassium ditertbutylimino dicarbonate[7] (2.85 g, 11.2 mmol, 1.5 equiv.) was added under a N_2 atmosphere. The flask was then heated at 60 °C and stirred for 48 h. DMF was removed and the residue dissolved in CH_2Cl_2. Undissolved salts were filtered off and the solvent was evaporated. The crude product was purified by chromatography (cyclohexane/Et_2O 7:3) to provide the product (3.14 g, 90% yield).
^{1}H NMR (300 MHz, $CDCl_3$) δ 7.54 (d, *J* = 2.3 Hz, 1H), 6.82 (d, *J* = 2.3 Hz, 1H), 4.97 (s, 2H), 3.98 (s, 3H), 3.92 (d, *J* = 5.7 Hz, 2H), 1.75 – 1.62 (m, 1H), 1.48-1.42 (m, 4H), 1.44 (s, *J* = 3.8 Hz, 18H), 0.92 (t, *J* = 7.5 Hz, 6H). ^{13}C NMR (75 MHz, $CDCl_3$) δ 167.12, 166.06, 160.76, 152.38, 149.19, 110.29, 109.14, 83.04, 70.64, 53.09, 51.57, 40.66, 28.09, 23.37, 11.18. MS calcd for $C_{24}H_{39}N_2O_7$ $[M+H]^+$ 467.2752, found (HR-ESI) 467.2760.

Compound 22 Compound **21** (1.8 g, 3.9 mmol, 1 equiv.) was dissolved in a mixture THF/water 2:1 (mL). LiOH·H_2O (243 mg, 5.8 mmol, 1.5 equiv.) was added and the solution was stirred at r.t. for 30 min. The solution was diluted with water and neutralized using 5% citric acid solution, then extracted with CH_2Cl_2 (3v). The organic phase was washed with water, dried over $MgSO_4$, filtered and evaporated to provide the product as a white solid (1.66 g, 95% yield).
^{1}H NMR (300 MHz, $CDCl_3$) δ 7.61 (d, *J* = 2.3 Hz, 1H), 6.92 (d, *J* = 2.3 Hz, 1H), 4.89 (s, 2H), 3.97 (d, *J* = 5.7 Hz, 2H), 1.74-1.64 (m, 1H), 1.47 (s, 18H), 1.41-1.51 (m, 4H), 0.92 (t, *J* = 7.5 Hz, 7H). ^{13}C NMR (75 MHz, $CDCl_3$) δ 168.25, 164.20, 158.59, 152.80, 147.34, 111.42, 108.21, 83.32, 71.25, 50.12, 40.65, 28.13, 23.34, 11.15. MS calcd for $C_{23}H_{37}N_2O_7$ $[M+H]^+$ 453.2595, found (HR-ESI) 453.2602.

Compound 23 Compound **22** (2.3 g, 5.1 mmol) was treated with a mixture of TFA 50% in CH_2Cl_2 at r.t. until TLC showed complete deprotection. The solvent was removed under reduced pressure to provide the product as TFA salt (quant. yield).
^{1}H NMR (300 MHz, MeOD-d_4) δ 7.67 (d, J = 2.3 Hz, 1H), 7.23 (t, J = 8.0 Hz, 1H), 4.29 (s, 2H), 4.08 (d, J = 5.6 Hz, 2H), 1.86-1.76 (m, 1H), 1.59 – 1.45 (m, 4H), 0.97 (t, J = 7.5 Hz, 6H). ^{13}C NMR (75 MHz, MeOD-d_4) δ 169.07, 167.52, 155.68, 150.25, 112.82, 112.61, 72.24, 44.00, 42.05, 24.31, 11.39. MS calcd for $C_{13}H_{20}N_2O_3$ $[M+H]^+$ 253.1547, found (HR-ESI) 253.1552.

Compound 24 Compound **23** (from deprotection step, 5.1 mmol, 1 equiv.) was dissolved in a mixture of dioxane/DMF 2:1 (60 mL) and a 10% *w/v* $NaHCO_3$ solution (50 mL) was added. The resulting slurry was cooled to 0 °C and a solution of Fmoc-OSu (1.9 g, 5.6 mmol, 1.1 equiv) in 15 mL dioxane was added dropwise over 1 h. The reaction mixture was stirred at r.t. overnight. The resulting mixture was diluted with water and pH was brought to 2-3 by dropwise addition of citric acid solution 5%. The precipitate was collected by filtration and washed with water. The compound was obtained as a white solid (1.33 g, 55% yield).
^{1}H NMR (300 MHz, MeOD-d_4) δ 7.79 (d, J = 7.5 Hz, 2H), 7.67 (d, J = 7.3 Hz, 2H), 7.47 (d, J = 2.3 Hz, 1H), 7.38 (m, 2H), 7.31 (m, 2H), 6.89 (d, J = 2.1 Hz, 1H), 4.43 – 4.32 (m, 4H), 4.23 (t, J = 7.0 Hz, 1H), 3.99 (d, J = 5.6 Hz, 2H), 1.73-1.61 (m, 1H), 1.54 – 1.39 (m, 4H), 0.93 (t, J = 7.4 Hz, 6H). ^{13}C NMR (75 MHz, MeOD-d_4) δ 172.74, 168.51, 160.36, 159.15, 157.79, 145.29, 142.60, 128.77, 128.16, 126.19, 120.91, 109.94, 109.79, 71.33, 67.94, 46.90, 42.07, 24.36, 11.42. MS calcd for $C_{28}H_{31}N_2O_5$ $[M+H]^+$ 475.2227, found (HR-ESI) 475.2223.

Dimethyl 4-(2-(trimethylsilyl)ethoxy)pyridine-2,6-dicarboxylate (25) Dimethyl chelidamate (**17**)[6] (3 g, 14.2 mmol, 1 equiv.) and PPh_3 (11.18 g, 42.6 mmol, 3 equiv.) were dissolved in dry THF (80 mL) under N_2. DIPEA (7.4 mL, 42.6 mmol, 3 equiv.) was added and then DIAD (8.39 mL, 42.6 mmol, 3 equiv.) was slowly added dropwise. The mixture was stirred for 1 h at r.t. and the formation of a white precipitate was observed. TMSE (6.1 mL, 42.6 mmol, 3 equiv.) was added, the precipitated disappeared and the reaction was stirred overnight at 35 °C under N_2. The solvent was removed, the residue dissolved in Et_2O and washed with water (3v). Then dried over Na_2SO_4, filtered and concentrated. The residue was triturated in Et_2O/cyclohexane 1:1 and the white solid removed by filtration. The same procedure was repeated twice, then the filtrated was dried under vacuum. The crude was purified by chromatography using Et_2O/cyclohexane 1:1. The compound was obtained as an oil (2.5 g, 56% yield).

^1H NMR (300 MHz, $CDCl_3$) δ 7.77 (s, 2H), 4.29 – 4.20 (m, 2H), 4.01 (s, 6H), 1.23 – 1.14 (m, 2H), 0.10 (s, 9H). ^{13}C NMR (75 MHz, $CDCl_3$) δ 167.08, 165.41, 149.81, 114.69, 67.16, 53.35, 17.46, -1.24. MS calcd for $C_{14}H_{22}NO_5Si$ $[M+H]^+$ 312.1262, found (HR-ESI) 312.1263.

Compound 26 Compound **25** (4.3 g, 13.8 mmol, 1 equiv.) was dissolved in CH_2Cl_2/MeOH 1:1 (80 mL). After cooling to 0 °C, $NaBH_4$ (1.05 g, 27.6 mmol, 2 equiv.) was added. The solution stirred for 30 min at 0 °C and for 1h ½ at r.t. The solution was acidified until pH 4-5 with a solution of citric acid 5%. Then the compound was extracted with CH_2Cl_2 (3v) and the organic layer washed with water, dried over $MgSO_4$, filtered and concentrated. The crude was purified by chromatography using Et_2O/cyclohexane 9:1 yielding the product as a white solid (3.3 g, 84 % yield).
^1H NMR (300 MHz, $CDCl_3$) δ 7.57 (d, J = 2.4 Hz, 1H), 7.01 (d, J = 2.4 Hz, 1H), 4.82 (d, J = 5.5 Hz, 2H), 4.27 – 4.17 (m, 2H), 4.02 (s, 3H), 3.29 (t, J = 5.5 Hz, 1H), 1.25 – 1.12 (m, 2H), 0.13 (s, 9H). ^{13}C NMR (75 MHz, $CDCl_3$) δ 166.68, 165.84, 162.03, 148.68, 111.21, 109.79, 66.55, 64.88, 53.06, 17.49, -1.22. MS calcd for $C_{13}H_{22}NO_4Si$ $[M+H]^+$ 284.1313, found (HR-ESI) 284.1313.

Compound 27 Compound **26** (2.25 g, 7.97 mmol, 1 equiv.) and PPh_3 (3.13 g, 11.9 mmol, 1.5 equiv.) were dissolved in dry DMF (60 mL) under N_2. Then CBr_4 (4.23 g, 12.7 mmol, 1.6 equiv.) and the solution was stirred at r.t. for 2 h. NaN_3 (1.55 g, 23.9 mmol, 3 equiv.) was added and the mixture was stirred overnight. The solution was diluted with Et_2O and washed with water, then dried over Na_2SO_4, filtered and concentrated. The residue was triturated in Et_2O/cyclohexane 1:1 and the solid was removed by filtration. The filtrate was concentrated and purified by chromatography using Et_2O/cyclohexane 1:1. The compound was obtained as a yellow oil (2.15 g, 87% yield).
^1H NMR (300 MHz, $CDCl_3$) δ 7.57 (d, J = 2.4 Hz, 1H), 7.04 (d, J = 2.3 Hz, 1H), 4.58 (s, 2H), 4.30 – 4.12 (m, 2H), 4.00 (s, 3H), 1.23 – 1.09 (m, 2H), 0.10 (s, 9H). ^{13}C NMR (75 MHz, $CDCl_3$) δ 166.94, 165.74, 157.95, 149.48, 111.45, 111.16, 66.68, 55.64, 53.25, 17.48, -1.23. MS calcd for $C_{13}H_{21}N_4O_3Si$ $[M+H]^+$ 309.1377, found (HR-ESI) 309.1378.

Compound 28 Compound **27** (5 g, 16.2 mmol, 1 equiv.) was dissolved in dry THF (300 mL) and N_2 was purged in the solution. Then Boc_2O (10.62 g, 48.7 mmol, 3) and Pd/C catalyst (500 mg) were added. The reaction was then stirred overnight under N_2 atmosphere. The solution was filtered through a celite pad and washed with EtOAc. The filtrate was concentrated and

purified by chromatography using Et_2O/cyclohexane 6:4. The product was obtained as a white solid (4.8 g, 77% yield).

^{1}H NMR (300 MHz, $CDCl_3$) δ 7.52 (d, *J* = 2.4 Hz, 1H), 6.96 (d, *J* = 2.3 Hz, 1H), 5.37 (s br, 1H), 4.45 (d, *J* = 5.9 Hz, 2H), 4.23 – 4.11 (m, 2H), 3.99 (s, 3H), 1.46 (s, 9H), 1.19 – 1.11 (m, 2H), 0.09 (s, 9H). ^{13}C NMR (75 MHz, $CDCl_3$) δ 166.67, 165.94, 160.37, 156.12, 149.11, 111.13, 66.50, 53.13, 46.21, 28.53, 17.52, -1.22. MS calcd for $C_{18}H_{31}N_2O_5Si$ $[M+H]^+$ 383.1997, found (HR-ESI) 383.1999.

Compound 29 Compound **28** (4.7 g, 12.28 mmol, 1 equiv.) was dissolved in THF/H_2O 5:1 (120 mL), then LiOH·H_2O (773 mg, 18.43 mmol, 1.5 equiv.) was added. The mixture was stirred at r.t. for 30 min (complete conversion from TLC). The reaction mixture was diluted with water and citric acid solution was added until acid pH. The compound was extracted with CH_2Cl_2 (3v), dried over $MgSO_4$, filtered and concentrated. The compound was obtained as a white solid (4.2 g, 93% yield).

^{1}H NMR (300 MHz, MeOD-d_4) δ 7.52 (d, *J* = 2.4 Hz, 1H), 6.99 (d, *J* = 2.4 Hz, 1H), 4.36 (s, *J* = 8.7 Hz, 2H), 4.33 – 4.25 (m, 2H), 1.46 (s, 9H), 1.23 – 1.15 (m, 2H), 0.11 (s, 9H). ^{13}C NMR (75 MHz, MeOD-d_4) δ 169.64, 169.41, 160.28, 158.60, 154.36, 110.55, 80.69, 68.05, 45.50, 28.73, 18.12, -1.36. MS calcd for $C_{17}H_{29}N_2O_5Si$ $[M+H]^+$ 369.1840, found (HR-ESI) 369.1842.

Compound 30 Compound **29** (4.1 g, 11.13 mmol) was dissolved in dioxane (30 mL) and HCl 4 M in dioxane was added (30 mL). The mixture was stirred at r.t. until complete conversion. Then the solvent was removed under vacuum yielding quantitatively the compound that was used directly in the next step.

^{1}H NMR (300 MHz, MeOD-d_4) δ 7.66 (d, *J* = 2.3 Hz, 1H), 7.28 (d, *J* = 2.3 Hz, 1H), 4.36-4.31 (m, 2H), 4.33 (s, 2H), 1.24 – 1.16 (m, 2H), 0.12 (s, 9H). ^{13}C NMR (75 MHz, MeOD-d_4) δ 171.02, 164.94, 154.20, 148.06, 113.95, 113.92, 69.81, 42.71, 18.15, -1.39. MS calcd for $C_{12}H_{19}N_2O_3Si$ $[M-H]^-$ 267.1170, found (HR-ESI) 267.1171.

Compound 31 Compound **30** (from previous step, 11.13 mmol, 1 equiv.) was dissolved in dioxane/DMF 1:1 (200 mL) and $NaHCO_{3(aq)}$ 15% (100 mL) was added. The suspension was cooled to 0 °C and a solution of Fmoc-OSu (4.12 g, 12.24 mmol, 1.1 equiv.) in dioxane (25 mL) was added dropwise. The reaction mixture was stirred overnight at r.t. The mixture was diluted with water and citric acid solution 5% was added to neutralize the pH. The precipitated obtained was recovered by filtration and then triturated in Et_2O. The compound was recovered as a white solid (3 g, 55% yield).

^{1}H NMR (500 MHz, DMSO-d_6) δ 8.13 (t, *J* = 6.1 Hz, 1H), 7.88 (d, *J* = 7.5 Hz, 2H), 7.69 (d, *J* = 7.4 Hz, 2H), 7.40 (m, 3H), 7.30 (t, *J* = 7.4 Hz, 2H), 6.74 (s, 1H), 4.31 (d, *J* = 7.0 Hz, 2H), 4.27 (d, *J* = 6.0 Hz, 2H), 4.22 (t, *J* = 6.8 Hz, 1H), 4.14 (t, *J* = 7.8 Hz, 2H), 1.08 (t, *J* = 7.8 Hz, 2H), 0.05 (s, 9H). ^{13}C NMR (75 MHz, DMSO-d_6) δ 168.32, 165.97, 159.41, 158.89, 157.00, 144.07, 141.00, 128.00, 127.45, 125.44, 120.43, 108.25, 108.18, 66.00, 65.65, 46.96, 45.90, 17.06, -1.05. MS calcd for $C_{27}H_{31}N_2O_5Si$ $[M+H]^+$ 491.1997, found (HR-ESI) 491.2005.

***Tert*-butyl 4-isobutoxy-8-nitroquinoline-2-carboxylate** (**33**) 4-isobutoxy-8-nitroquinoline-2-carboxylic acid (**32**)[8] (2 g, 6.89 mmol, 1 equiv.) was dissolved in a solution of dry CH_2Cl_2/*t*BuOH 1:1 (25 mL) under N_2 atmosphere. Then Boc_2O (3 g, 13.8 mmol, 2 equiv.) and DMAP (84 mg, 0.69 mmol, 0.1 equiv.) were added to the solution. The reaction mixture was stirred at r.t. for 48 h. The solvents were removed under vacuum and the residue dissolved in CH_2Cl_2. The organic phase was washed with $NaHCO_{3(sat)}$ solution, dried over $MgSO_4$, filtered and concentrated to dryness. The crude was purified by chromatography using CH_2Cl_2 as eluent. The product was obtained as a light yellow solid (2.1 g, 88% yield).
^{1}H NMR (400 MHz, $CDCl_3$) δ 8.46 (dd, *J* = 8.4, 1.3 Hz, 1H), 8.09 (dd, *J* = 7.5, 1.3 Hz, 1H), 7.62 (dd, *J* = 8.3, 7.6 Hz, 1H), 7.59 (s, 1H), 4.07 (d, *J* = 6.5 Hz, 2H), 2.35-2.25 (m, 1H), 1.67 (s, 9H), 1.14 (d, *J* = 6.7 Hz, 6H). ^{13}C NMR (75 MHz, $CDCl_3$) δ 164.20, 162.76, 153.04, 148.62, 140.21, 126.41, 125.73, 125.04, 123.33, 102.00, 83.12, 75.58, 28.15, 21.93, 19.33. MS calcd for $C_{18}H_{23}N_2O_5$ $[M+H]^+$ 347.16015, found (HR-ESI) 347.15948.

***Tert*-butyl 4-isobutoxy-8-aminoquinoline-2-carboxylate** (**34**) *Tert*-butyl 4-isobutoxy-8-nitroquinoline-2-carboxylate (**33**) (500 mg, 1.44 mmol) was dissolved in EtOAc (30 mL) under N_2 atmosphere. Pd/C catalyst (50 mg, 10% by mass) was added and N_2 exchanged with H_2. The reaction mixture was stirred overnight at r.t. under H_2 atmosphere. The resulting mixture was filtered over celite and solvent was evaporated under reduced pressure to yield the title compound as a bright yellow powder (0.45 g, 98% yield).
^{1}H NMR (300 MHz, $CDCl_3$) δ 7.51 (dd, *J* = 8.3, 1.3 Hz, 1H), 7.43 (s, 1H), 7.35 (dd, *J* = 8.2, 7.6 Hz, 1H), 6.92 (dd, *J* = 7.5, 1.3 Hz, 1H), 5.08 (s br, 2H), 4.00 (d, *J* = 6.4 Hz, 2H), 2.31-2.22 (m, 1H), 1.67 (s, 9H), 1.13 (d, *J* = 6.7 Hz, 6H). ^{13}C NMR (75 MHz, $CDCl_3$) δ 165.05, 162.64, 147.51, 144.96, 138.53, 128.40, 122.95, 110.81, 109.75, 100.69, 82.16, 74.87, 28.36, 28.30, 19.42. MS calcd for $C_{18}H_{25}N_2O_3$ $[M+H]^+$ 317.18597, found (HR-ESI) 317.18568.

Compound 35 Compound **31** (800 mg. 1.63 mmol, 1.1 equiv.), *tert*-butyl 4-isobutoxy-8-aminoquinoline-2-carboxylate (**34**) (455 mg, 1.44 mmol, 1 equiv.) and PyBOP (1.696 g, 3.26

mmol, 2.2 equiv.) were dissolved in dry $CHCl_3$ (15 mL) under N_2. DIPEA (1.1 mL, 6.34 mmol, 4.4 equiv.) was added and the solution stirred at r.t. for 2 days. The reaction mixture was diluted with $CHCl_3$, washed with citric acid solution 5% and $NaHCO_3$ solution 5%. The organic phase was dried over $MgSO_4$, filtered and concentrated. The crude was purified by chromatography using CH_2Cl_2. The compound was obtained as a pale yellow solid (870 mg, 76% yield).

^{1}H NMR (300 MHz, $CDCl_3$) δ 13.01 (s, 1H), 8.93 (d, *J* = 7.4 Hz, 1H), 7.96 (dd, *J* = 7.5, 1.0 Hz, 1H), 7.70 (d, *J* = 2.1 Hz, 1H), 7.66 (d, *J* = 8.1 Hz, 1H), 7.54 (d, *J* = 7.6 Hz, 2H), 7.50-7.45 (m, 1H), 7.45-7.43 (m, 1H), 7.43 (s, 1H), 7.18 (t, *J* = 7.5 Hz, 2H), 6.97 (d, *J* = 1.7 Hz, 1H), 6.84 (t, *J* = 7.4 Hz, 2H), 4.59 (d, *J* = 6.6 Hz, 2H), 4.31–4.17 (m, 4H), 4.14 – 4.06 (m, 1H), 4.05 (d, *J* = 6.3 Hz, 2H), 2.38–2.25 (m, 1H), 1.67 (s, 9H), 1.25–1.17 (m, 2H), 1.17 (d, *J* = 6.7 Hz, 6H), 0.10 (s, 9H). ^{13}C NMR (75 MHz, $CDCl_3$) δ 167.28, 163.63, 163.15, 162.58, 158.97, 157.46, 151.94, 147.98, 144.08, 141.20, 139.86, 135.35, 128.57, 127.50, 126.80, 125.24, 122.20, 119.75, 117.13, 115.81, 111.66, 106.86, 101.27, 82.97, 75.02, 66.74, 66.51, 47.32, 46.15, 28.42, 28.31, 19.43, 17.65, -1.19. MS calcd for $C_{45}H_{53}N_4O_7Si$ $[M+H]^+$ 789.3678, found (HR-ESI) 789.3671.

Compound 36 Compound **35** (500 mg, 0.63 mmol) was dissolved in a solution 4M of HCl in dioxane (18 mL). The mixture was stirred at r.t. for 5 h. The solvent was removed under vacuum. The residue was recrystalized from CH_2Cl_2/cyclohexane. The solid compound was collected by filtration (430 mg, 93% yield).

^{1}H NMR (500 MHz, DMSO-d_6) δ 13.66 (s br, 1H), 12.63 (s, 1H), 8.85 (d, *J* = 7.6 Hz, 1H), 8.15 (s br, 1H), 7.91 (dd, *J* = 8.4, 1.1 Hz, 1H), 7.86 (d, *J* = 7.5 Hz, 2H), 7.74 – 7.69 (m, 1H), 7.67 (d, *J* = 7.5 Hz, 2H), 7.64 (s, 1H), 7.59 (d, *J* = 2.2 Hz, 1H), 7.37 (t, *J* = 7.5 Hz, 2H), 7.21 (t, *J* = 7.3 Hz, 2H), 7.05 (d, *J* = 2.1 Hz, 1H), 4.47 (d, *J* = 6.2 Hz, 2H), 4.32-4.26 (m, 4H), 4.20 (t, *J* = 7.1 Hz, 1H), 4.15 (d, *J* = 6.4 Hz, 2H), 2.27-2.18 (m, 1H), 1.16 – 1.12 (m, 2H), 1.10 (d, *J* = 6.7 Hz, 6H), 0.07 (s, 9H). ^{13}C NMR (75 MHz, DMSO-d_6) δ 168.14, 166.78, 161.81, 161.63, 160.52, 156.83, 150.80, 143.79, 140.67, 138.76, 134.55, 129.07, 127.65, 127.01, 125.34, 121.00, 120.10, 115.69, 115.50, 110.12, 106.41, 102.09, 74.44, 66.25, 65.94, 46.65, 45.56, 27.76, 19.05, 16.93, -1.24. MS calcd for $C_{41}H_{45}N_4O_7Si$ $[M+H]^+$ 733.3052, found (HR-ESI) 733.3058.

Compound 37 Compound **24** (775 mg. 1.63 mmol, 1.1 equiv.), *tert*-butyl 4-isobutoxy-8-aminoquinoline-2-carboxylate (**34**) (470 mg, 1.18 mmol, 1 equiv.) and PyBOP (1.696 g, 3.26 mmol, 2.2 equiv.) were dissolved in dry $CHCl_3$ (15 mL) under N_2. DIPEA (1.17 mL, 6.52 mmol, 4 equiv.) was added and the solution stirred at r.t. for 2 days. The reaction mixture was

diluted with CH_2Cl_2, washed with citric acid solution 5% and $NaHCO_3$ 5% solution. The organic phase was dried over $MgSO_4$, filtered and concentrated. The crude was purified by chromatography using CH_2Cl_2. The compound was obtained as a yellow solid (700 mg, 61% yield).

^{1}H NMR (300 MHz, $CDCl_3$) δ 12.99 (s, 1H), 8.93 (d, *J* = 7.3 Hz, 1H), 7.96 (d, *J* = 7.9 Hz, 1H), 7.73 (d, *J* = 2.2 Hz, 1H), 7.67 (t, *J* = 8.0 Hz, 1H), 7.54 (d, *J* = 7.5 Hz, 2H), 7.48 – 7.41 (m, 3H), 7.43 (s, 1H), 7.18 (t, *J* = 7.4 Hz, 2H), 7.01 (s, 1H), 6.85 (t, *J* = 7.4 Hz, 2H), 4.58 (d, *J* = 6.6 Hz, 2H), 4.28 (d, *J* = 7.2 Hz, 2H), 4.10-4.05 (m, 1H), 4.05 (d, *J* = 6.3 Hz, 2H), 4.01 (d, *J* = 5.7 Hz, 2H), 2.38-2.25 (m, 1H), 1.76 – 1.68 (m, 1H), 1.68 (s, 9H), 1.54-1.41 (m, 4H), 1.17 (d, *J* = 6.7 Hz, 6H), 0.94 (t, *J* = 7.4 Hz, 6H). ^{13}C NMR (75 MHz, $CDCl_3$) δ 167.63, 163.65, 163.16, 162.56, 158.95, 157.48, 151.95, 147.97, 144.07, 141.19, 139.84, 135.34, 128.59, 127.51, 126.80, 125.24, 122.19, 119.76, 117.15, 115.81, 111.70, 106.84, 101.26, 82.99, 75.02, 70.76, 66.74, 47.31, 46.16, 40.80, 28.42, 28.31, 23.40, 19.44, 11.24. MS calcd for $C_{46}H_{53}N_4O_7$ $[M+H]^+$ 773.3909, found (HR-ESI) 773.3902.

Compound 38 Compound **37** (650 mg, 0.84 mmol) were treated with TFA/CH_2Cl_2 1:1 (8 mL) at r.t. for 24 h. The solvent was removed and the residue triturated in CH_2Cl_2. The solid compound was collected by filtration (550 mg, 90% yield).

^{1}H NMR (300 MHz, DMSO-d_6) δ 12.56 (s, 1H), 8.86 (dd, *J* = 7.7, 1.1 Hz, 1H), 8.04 (t, *J* = 6.4 Hz, 1H), 7.92 (dd, *J* = 8.3, 0.9 Hz, 1H), 7.86 (d, *J* = 7.5 Hz, 2H), 7.75 (d, *J* = 7.9 Hz, 1H), 7.69 (d, *J* = 8.1 Hz, 2H), 7.63 (s br, 2H), 7.38 (t, *J* = 7.3 Hz, 2H), 7.24 (t, *J* = 7.1 Hz, 2H), 7.05 (d, *J* = 2.0 Hz, 1H), 4.47 (d, *J* = 6.1 Hz, 2H), 4.34 (d, *J* = 7.1 Hz, 2H), 4.22 (t, *J* = 7.0 Hz, 1H), 4.15 (d, *J* = 6.3 Hz, 2H), 4.07 (d, *J* = 5.8 Hz, 2H), 2.28 – 2.14 (m, 1H), 1.71-1.61 (m, 1H), 1.51 – 1.35 (m, 4H), 1.09 (d, *J* = 6.7 Hz, 6H), 0.89 (t, *J* = 7.4 Hz, 6H). ^{13}C NMR (126 MHz, DMSO-d_6) δ 167.22, 166.12, 162.41, 161.53, 160.50, 156.54, 150.66, 148.47, 143.77, 140.72, 138.46, 134.53, 128.27, 127.61, 126.96, 125.12, 121.47, 120.13, 116.28, 115.52, 109.91, 106.33, 101.67, 74.71, 70.26, 65.73, 46.67, 45.44, 27.67, 22.69, 18.95, 10.90. MS calcd for $C_{42}H_{45}N_4O_7$ $[M+H]^+$ 717.3283, found (HR-ESI) 717.3276.

5.3 Solid phase synthesis general methods

The oligomers **9-12** were synthesized using SPS on SASRIN resin. Chlorination of the resin and insertion of the first monomer were performed as described below. Cleavage of the foldamers from the resin was performed following conditions for SASRIN resin (CH_2Cl_2/HFIP 4:1 for 5 h at r.t.).

Quinoline monomers (**16**, **32** and Fmoc-Q^{iBu}-OH[9]) were activated *via* formation of acid chloride and coupled following standard SPS procedures previously reported.[9]

Dimeric blocks **36** and **38** were first prepared in solution and then coupled on solid support following standard activation and coupling conditions.

For the coupling of **24** on an aliphatic amine, previously reported protocol for coupling with HOBt/HBTU was used.[10]

Chlorination of SASRIN resin[11]

SASRIN resin resin (200 mg, max 0.2 mmol) was swollen in 2 mL dry CH_2Cl_2 for 1 h under N_2. PPh_3 (273 mg, 1.04 mmol, 5.2 equiv.) and CCl_4 (100 µL, 1.04 mmol, 5.2 equiv.) were then added in that order and the resin stirred at r.t. under N_2 for 24 h. The resin was filtered, and washed with anhydrous CH_2Cl_2 and then dried and desiccated under vacuum.

Loading of first unit

SASRIN chloride resin (200 mg, max 0.2 mmol) was first swollen in 2 mL of dry DMF for 1 h under N_2. Fmoc-monomer (0.3 mmol, 1.5 equiv.) and CsI (0.3 mmol, 1.5 equiv.) were then added, followed by DIPEA (0.3 mmol, 1.5 equiv.). The reaction mixture was stirred at r.t. under N_2 for 24 h. The resin was then washed with DMF and CH_2Cl_2. Then loading was measured using UV spectroscopic method.

5.4 Synthesis of oligomers

NO_2-Q<u>X</u>QQ<u>Y</u>Q<u>X</u>QQ-OH (**9**) Compound **9** was synthesized using the SPS procedures previously described. After cleavage from the resin, the crude product was purified by semi-preparative RP-HPLC. After lyophilization, the product was recovered as a yellow solid.

^{1}H NMR (400 MHz, $CDCl_3$) δ 11.37 (s, 1H), 11.33 (s, 1H), 11.03 (s, 1H), 10.85 (s, 2H), 10.76 (s, 1H), 10.50 (s, 1H), 8.32 (dd, *J* = 8.5, 0.9 Hz 1H), 8.29 (dd, *J* = 8.2, 1.3 Hz, 1H), 8.20 (s, 1H), 8.20-8.18 (m, 2H), 8.07 (d, *J* = 7.2 Hz, 1H), 7.99 (dd, *J* = 8.2, 1.0 Hz, 1H), 7.92 – 7.84 (m, 2H), 7.81 (dd, *J* = 8.3, 0.9 Hz, 1H), 7.77 (dd, *J* = 3.4, 0.9 Hz, 1H), 7.75 (dd, *J* = 3.5, 0.9 Hz, 1H), 7.73 (s, 1H), 7.68 (dd, *J* = 8.3, 0.9 Hz, 1H), 7.65 (s, 1H), 7.52 (d, *J* = 7.3 Hz, 1H),

7.48 (d, *J* = 7.0 Hz, 1H), 7.40-7.35 (m, 1H), 7.38-7.22 (m, 5H), 7.15 – 7.09 (m, 1H), 7.09 (s, 1H), 7.08-6.98 (m, 3H), 6.91-6.87 (m, 1H), 6.69 (d, *J* = 1.9 Hz, 1H), 6.63 (s, 1H), 6.56 (d, *J* = 2.0 Hz, 1H), 6.49 (s, 1H), 6.43 (s, 1H), 5.96 (s, 1H), 5.80 (s, 1H), 4.36-4.24 (m, 2H), 4.09 (dd, *J* = 8.6, 6.2 Hz, 1H), 4.02 (dd, *J* = 8.8, 6.7 Hz, 1H), 3.86-3.90 (m, 2H), 3.86 – 3.75 (m, 4H), 3.72 (d, *J* = 6.3 Hz, 2H), 3.69 – 3.65 (m, 2H), 3.52 (dd, *J* = 8.7, 7.2 Hz, 1H), 3.38 (dd, *J* = 17.4, 3.4 Hz, 1H), 2.49 – 2.42 (m, 2H), 2.37 – 2.20 (m, 4H), 1.79 (s, 9H), 1.72 (s, 9H), 1.46 – 1.41 (m, 2H), 1.33 – 1.08 (m, 36H), 0.32 (s, 9H). MS calcd for $C_{124}H_{132}N_{18}O_{21}Si$ $[M+2H]^{2+}$ 1118.4786, found (HR-ESI) 1118.4761.

NO_2-QXQPYQXPQ-OH (**10**) Compound **10** was synthesized using the SPS procedures previously described. After cleavage from the resin, the crude product was purified by semi-preparative RP-HPLC. After lyophilization, the product was recovered as a yellow solid.
^{1}H NMR (300 MHz, $CDCl_3$) δ 11.51 (s, 1H), 11.41 (s, 1H), 11.11 (s, 1H), 10.96 (s, 1H), 10.86 (s, 1H), 8.66 (s br, 1H), 8.41 (dd, *J* = 7.7, 0.9 Hz, 1H), 8.40 – 8.36 (m, 1H), 8.31 (dd, *J* = 7.6, 0.9 Hz, 1H), 8.27 (dd, *J* = 8.3, 1.4 Hz, 1H), 8.12 (dd, *J* = 7.5, 0.9 Hz, 1H), 7.87 (dd, *J* = 8.2, 1.2 Hz, 2H), 7.85 (s, 1H), 7.83 (s br, 1H), 7.77 (dd, *J* = 8.3, 1.3 Hz, 1H), 7.72 (s br, 1H), 7.71 (dd, *J* = 7.3, 1 Hz, 1H), 7.60 (s, 1H), 7.59 (s, 1H), 7.58 (s, 1H), 7.50 (dt, *J* = 10.1, 8.1 Hz, 2H), 7.32 (d, *J* = 1.3 Hz, 1H), 7.25 (d, *J* = 2.2 Hz, 1H), 7.16 – 7.10 (m, 2H), 7.06-7.02 (m, 1H), 7.01 (s, 1H), 6.93– 6.88 (m, 1H), 6.69 (d, *J* = 2.1 Hz, 1H), 6.65 (d, *J* = 2.1 Hz, 1H), 6.60 (d, *J* = 2.1 Hz, 1H), 6.59 (s, 1H), 6.53 (d, *J* = 2.1 Hz, 1H), 6.20 (d, *J* = 2.1 Hz, 1H), 5.67 (s, 1H), 4.44-4.35 (m, 3H), 4.24-4.17 (m, 3H), 4.07 – 4.01 (m, 1H), 3.89-3.80 (m, 4H), 3.77– 3.74 (m, 5H), 3.53 – 3.43 (m, 2H), 3.30 (dd, *J* = 18.2, 4.0 Hz, 1H), 3.10 (d, *J* = 16.1 Hz, 1H), 2.55 – 2.47 (m, 2H), 2.42 – 2.15 (m, 4H), 1.78 (s, 9H), 1.73 (s, 9H), 1.71 – 1.40 (m, 10H), 1.38 – 1.22 (m, 12H), 1.21 – 1.07 (m, 12H), 1.07 – 0.96 (m, 6H), 0.94 – 0.80 (m, 6H), 0.32 (s, 9H). MS calcd for $C_{122}H_{139}N_{18}O_{21}Si$ $[M+H]^+$ 2220.0126, found (HR-ESI) 2220.0162.

NO_2-QXPQYQXPQ-OH (**11**) Compound **11** was synthesized using the SPS procedures previously described. After cleavage from the resin, the crude product was purified by semi-preparative RP-HPLC. After lyophilization, the product was recovered as a yellow solid.
^{1}H NMR (300 MHz, $CDCl_3$) δ 11.20 (s, 1H), 11.06 (s, 1H), 10.99 (s, 1H), 10.95 (s, 1H), 10.82 (s, 1H), 8.85 (t, *J* = 4.0 Hz, 1H), 8.62 (t, *J* = 3.9 Hz, 1H), 8.33 (dd, *J* = 8.2, 1.3 Hz, 1H), 8.32 (s br, 1H), 8.26 (dd, *J* = 7.6, 0.9 Hz, 1H), 8.17 (dd, *J* = 7.0, 0.8 Hz, 1H), 7.93 (dd, *J* = 8.3, 1.1 Hz, 1H), 7.82 (dd, *J* = 8.4, 1.0 Hz, 1H), 7.78 (dd, *J* = 7.5, 0.8 Hz, 1H), 7.77-7.68 (m, 3H), 7.68 (s, 1H), 7.64 (dd, *J* = 8.0, 0.9 Hz, 1H), 7.60 (s, 1H), 7.52 (dd, *J* = 7.5, 1.3 Hz, 1H), 7.44 – 7.35 (m, 1H), 7.24-7.14 (m, 4H), 7.11 – 6.98 (m, 2H), 7.08 (s, 1H), 6.68 (s, 1H), 6.68-6.65 (m, 1H),

6.65 (s, 1H), 6.61 (d, *J* = 1.9 Hz, 1H), 6.37 (s, 1H), 6.34 (s, 1H), 6.28 (d, *J* = 2.0 Hz, 1H), 4.86-3.82 (broad signals), 3.79 (d, *J* = 5.6 Hz, 2H), 3.76 (d, *J* = 6.5 Hz, 1H), 3.50-3.25 (broad signal), 2.47 – 2.28 (m, 4H), 2.26-2.17 (m, 2H), 1.75 (s, 9H), 1.66 (s, 9H), 1.58 – 1.51 (m, 4H), 1.51 – 1.44 (m, 4H), 1.41 – 1.34 (m, 2H), 1.27 (d, *J* = 6.7 Hz, 6H), 1.24 (d, *J* = 6.7 Hz, 6H), 1.18 (d, *J* = 6.7 Hz, 6H), 1.11 (d, *J* = 6.7 Hz, 6H), 1.04 (t, *J* = 7.4 Hz, 6H), 0.96 (t, *J* = 7.4 Hz, 6H), 0.24 (s, 9H). MS calcd for $C_{122}H_{139}N_{18}O_{21}Si$ $[M+H]^+$ 2220.0126, found (HR-ESI) 2220.01733.

NO_2-QXPPYQXPP-OH (**12**) Compound **12** was synthesized using the SPS procedures previously described. After cleavage from the resin, the crude product was purified by semi-preparative RP-HPLC. After lyophilization, the product was recovered as a yellow solid.
^1H NMR (300 MHz, $CDCl_3$) δ 11.92 (s, 1H), 11.74 (s, 1H), 11.22 (s, 1H), 9.32 (s br, 1H), 9.08 (s br, 1H), 8.86 (s br, 1H), 8.61 (d, *J* = 7.3 Hz, 1H), 8.48 (d, *J* = 8.0 Hz, 1H), 8.37 (d, *J* = 7.4 Hz, 1H), 8.12 (s br, 1H), 8.00 (d, *J* = 7.3 Hz, 1H), 7.87 (s, 1H), 7.77 (s, 1H), 7.75 (s, 1H), 7.69 (s, 1H), 7.63-7.46 (m, 4H), 7.08 (s, 1H), 7.05 (s, 1H), 7.02 (s, 1H), 6.99 (s, 1H), 6.81 (s, 1H), 6.77 (d, *J* = 1.6 Hz, 1H), 6.67 (s, 1H), 6.62 (d, *J* = 1.0 Hz, 1H), 4.84 (d, *J* = 2.9 Hz, 2H), 4.31 – 4.21 (m, 4H), 3.89 (s br, 4H), 3.83 (d, *J* = 5.1 Hz, 2H), 3.78 (d, *J* = 4.9 Hz, 2H), 3.72 (d, *J* = 5.4 Hz, 2H), 2.38-2.12 (m, 4H), 2.08-1.98 (m, 2H), 1.72 (s, 9H), 1.65 (s, 9H), 1.50-1.38 (m, 12H), 1.35 – 1.27 (m, 6H), 1.12 (d, *J* = 6.5 Hz, 6H), 0.98 – 0.76 (m, 30H), 0.07 (s, 9H). MS calcd for $C_{120}H_{147}N_{18}O_{21}Si$ $[M+H]^+$ 2204.0752, found (HR-ESI) 2204.0798.

NO_2-QXQQYQXQQ-OMe (**1**) Compound **9** (6 mg, 2.68 μmol, 1 equiv.) was dissolved in a mixture of dry THF/MeOH 3:2 (1.25 mL) under N_2. $TMSCHN_2$ (soluz. 2M in hexane, 3 μL, 5.36 μmol, 2 equiv.) was added dropwise and the solution stirred at r.t. for 2h. Few drops of acetic acid were added and the solution stirred for 5 min at r.t. Then the solution was diluted with CH_2Cl_2, washed with $NaHCO_3$, dried $MgSO_4$, filtered and concentrated. The crude product was purified by semi-prep RP-HPLC (4 mg, 66% yield).
^1H NMR (400 MHz, $CDCl_3$) δ 11.56 (s, 1H), 11.33 (s, 1H), 11.31 (s, 1H), 11.03 (s, 1H), 10.86 (s, 1H), 10.82 (s, 1H), 10.52 (s, 1H), 8.31 (dd, *J* = 6.6, 1.2 Hz, 1H), 8.30 (dd, *J* = 8.2, 1.3 Hz, 1H), 8.20 (dd, *J* = 7.5, 1.1 Hz, 1H), 8.15 (t, *J* = 3.3 Hz, 1H), 8.00 (dd, *J* = 8.3, 1.2 Hz, 1H), 7.96 (dd, *J* = 7.5, 1.0 Hz, 1H), 7.90 (dd, *J* = 5.1, 1.2 Hz, 1H), 7.88 (dd, *J* = 5.9, 1.2 Hz, 1H), 7.86 (dd, *J* = 8.4, 1.2 Hz, 1H), 7.82 (dd, *J* = 8.3, 1.2 Hz, 1H), 7.72 (s, 2H), 7.71-7.68 (m, 1H), 7.67 (dd, *J* = 6.3, 1.2 Hz, 1H), 7.57 (s, 1H), 7.53 (dd, *J* = 7.4, 1.2 Hz, 1H), 7.44 (dd, *J* = 7.5, 1.2 Hz, 1H), 7.36 (t, *J* = 8.0 Hz, 1H), 7.33 – 7.24 (m, 4H), 7.15 – 7.09 (m, 1H), 7.09 (s, 1H), 7.09 – 7.06 (m, 1H), 7.05 – 6.97 (m, 2H), 6.93 – 6.88 (m, 1H), 6.66 (d, *J* = 2.2 Hz, 1H), 6.58 (s, 1H and dd, d, *J* = 2.2 Hz, 1H), 6.47 (s, 1H), 6.40 (s, 1H), 5.94 (s, 1H), 5.81 (s, 1H), 4.37-4.24 (m,

2H), 4.12 – 4.01 (m, 2H), 3.98-3.90 (m, 2H), 3.85-3.76 (m, 4H), 3.70-3.63 (m, 2H), 3.53 (dd, *J* = 8.8, 7.2 Hz, 1H), 3.35 (dd, *J* = 17.0, 3.3 Hz, 1H), 3.16 (s, 3H), 2.49 – 2.41 (m, 1H), 2.40 – 2.14 (m, 5H), 1.78 (s, 9H), 1.72 (s, 9H), 1.48 – 1.43 (m, 6H), 1.33 – 1.08 (m, 32H), 0.32 (s, 9H). MS calcd for $C_{125}H_{134}N_{18}O_{21}Si$ $[M+2H]^{2+}$ 1125.4865, found (HR-ESI) 1125.4852.

NO_2-QXQPYQXPQ-OMe (**2**) Compound **10** (10 mg, 4.5 µmol, 1 equiv.) was dissolved in a mixture of dry THF/MeOH 3:2 (1.25 mL) under N_2. $TMSCHN_2$ (soluz. 2M in hexane, 5 µL, 9 µmol, 2 equiv.) was added dropwise and the solution stirred at r.t. for 2h. Few drops of acetic acid were added and the solution stirred for 5 min at r.t. Then the solution was diluted with CH_2Cl_2, washed with $NaHCO_3$, dried $MgSO_4$, filtered and concentrated. The crude product was purified by semi-preparative RP-HPLC (6 mg, 60% yield).
^{1}H NMR (500 MHz, $CDCl_3$) δ 11.49 (s, 1H), 11.37 (s, 1H), 11.24 (s, 1H), 11.05 (s, 1H), 10.78 (s, 1H), 8.91 (t, *J* = 3.7 Hz, 1H), 8.27 (t, *J* = 3.5 Hz, 1H), 8.25 (dd, *J* = 7.4, 1.2 Hz, 1H), 8.24 (dd, *J* = 7.4, 1.2 Hz, 1H), 8.22 (dd, *J* = 7.3, 1.4 Hz, 1H), 8.08 (dd, *J* = 7.5, 1.3 Hz, 1H), 7.83 (dd, *J* = 8.3, 1.2 Hz, 1H), 7.80 (dd, *J* = 8.3, 1.3 Hz, 1H), 7.74 (dd, *J* = 8.3, 1.3 Hz, 1H), 7.68 (dd, *J* = 7.5, 1.4 Hz, 1H), 7.66 (dd, *J* = 6.7, 1.4 Hz, 1H), 7.63 (t, *J* = 3.6 Hz, 1H), 7.56 (s, 1H), 7.54 (s, 1H), 7.50 (dd, *J* = 7.4, 1.1 Hz, 1H), 7.47 – 7.44 (m, 1H), 7.44-7.40 (m, 2H), 7.43 (s, 1H), 7.32 (t, *J* = 7.6 Hz, 1H), 7.27 (dd, *J* = 7.4, 1.4 Hz,1H), 7.20 (d, *J* = 2.3 Hz, 1H), 7.11 – 7.05 (m, 2H), 6.98 (s, 1H), 7.01 – 6.96 (m, 1H), 6.93 (dd, *J* = 7.6, 1.2 Hz, 1H), 6.87 – 6.83 (m, 1H), 6.59 (d, *J* = 2.3 Hz, 1H), 6.57 (d, *J* = 2.3 Hz, 1H), 6.55 (d, *J* = 2.2 Hz, 1H), 6.47 (d, *J* = 2.3 Hz, 1H), 6.38 (s, 1H), 6.13 (d, *J* = 2.4 Hz, 1H), 5.62 (s, 1H), 4.39-4.29 (m, 2H), 4.26 (dd, *J* = 16.7, 3.9 Hz, 1H), 4.22 – 4.14 (m, 3H), 4.00 (t, *J* = 7.9 Hz, 1H), 3.86-3.80 (m, 4H), 3.77 (dd, *J* = 8.0, 6.4 Hz, 1H), 3.62 (dd, *J* = 8.6, 6.4 Hz, 2H), 3.48 – 3.41 (m, 3H), 3.25 (s, 3H), 3.21 (dd, *J* = 17.7, 3.6 Hz, 1H), 3.05 (dd, *J* = 16.4, 3.1 Hz, 1H), 2.53-2.45 (m, 1H), 2.38-2.25 (m, 2H), 2.20-2.12 (m, 1H), 1.81-1.76 (m, 2H), 1.72-1.68 (m, 1H), 1.75 (s, 9H), 1.70 (s, 9H), 1.65-1.50 (m, 8H), 1.41 (dd, *J* = 9.0, 7.7 Hz, 2H), 1.34-1.22 (m, 12H), 1.17-1.12 (m, 6H), 1.11 – 1.05 (m, 12H), 1.05-1.00 (m, 6H), 0.28 (s, 9H). MS calcd for $C_{123}H_{142}N_{18}O_{21}Si$ $[M+2H]^{2+}$ 1117.5178, found (HR-ESI) 1117.5165.

NO_2-QXPQYQXPQ-OMe (**3**) Compound **11** (6 mg, 2.7 µmol, 1 equiv.) was dissolved in a mixture of dry THF/MeOH 3:2 (1.25 mL) under N_2. $TMSCHN_2$ (soluz. 2M in hexane, 3 µL, $5.4 \cdot 10^{-3}$ mmol, 2 equiv.) was added dropwise and the solution stirred at r.t. for 2h. Few drops of acetic acid were added and the solution stirred for 5 min at r.t. Then the solution was diluted with CH_2Cl_2, washed with $NaHCO_3$, dried $MgSO_4$, filtered and concentrated. The crude product was purified by semi-preparative RP-HPLC (3 mg, 50% yield).

^{1}H NMR (500 MHz, $CDCl_3$) δ 11.32 (s, 1H), 11.09 (s, 1H), 10.94 (s, 1H), 10.92 (s, 1H), 10.70 (s, 1H), 8.96 (t, *J* = 3.6 Hz, 1H), 8.86 (t, *J* = 4.0 Hz, 1H), 8.33 (dd, *J* = 8.2, 1.4 Hz, 1H), 8.27 (t, *J* = 3.6 Hz, 1H), 8.11 (d, *J* = 7.4 Hz, 1H), 8.05 (dd, *J* = 7.5, 1.0 Hz, 1H), 7.92 (dd, *J* = 8.2, 1.2 Hz, 1H), 7.85 (dd, *J* = 8.2, 1.1 Hz, 1H), 7.71 (dd, *J* = 8.3, 1.2 Hz, 1H), 7.70 (dd, *J* = 8.3, 1.2 Hz, 1H), 7.68 (dd, *J* = 8.3, 1.1 Hz, 1H), 7.65 (s, 1H), 7.64 (d, *J* = 2.1 Hz, 1H), 7.60 (d, *J* = 7.4 Hz, 1H), 7.51 (dd, *J* = 7.4, 1.3 Hz, 1H), 7.49 (s, 1H), 7.32 – 7.27 (m, 1H), 7.24 – 7.20 (m, 1H), 7.20 – 7.16 (m, 1H), 7.17 – 7.13 (m, 2H), 7.08 – 7.04 (m, 2H), 7.05 (s, 1H), 7.01 – 6.97 (m, 1H), 6.72 (d, *J* = 2.0 Hz, 1H), 6.64 (d, *J* = 2.0 Hz, 1H), 6.60 (d, *J* = 2.2 Hz, 1H), 6.58 (d, *J* = 1.9 Hz, 1H), 6.43 (s, 1H), 6.29 (s, 1H), 6.26 (s, 1H), 6.22 (d, *J* = 1.4 Hz, 1H), 4.40 (s br, 2H), 4.30 (broad signal), 4.17 – 3.91 (broad signals), 3.85 (d, *J* = 5.7 Hz, 6H), 3.78 (broad signal), 3.65 (d, *J* = 6.3 Hz, 2H), 3.33 (broad signal), 3.22 (s, 1H), 2.46 – 2.38 (m, 1H), 2.37-2.29 (m, 2H), 2.20 – 2.14 (m, 1H), 1.81-1.76 (m, 1H), 1.76 (s, 9H), 1.73 – 1.67 (m, 1H), 1.66 (s, 9H), 1.61 – 1.50 (m, 8H), 1.28 (t, *J* = 7 Hz, 6H), 1.23 (d, *J* = 6.6 Hz, 6H), 1.18 (d, *J* = 6.6 Hz, 6H), 1.10 (d, *J* = 6.8 Hz, 6H), 1.07 (t, *J* = 7.5 Hz, 6H), 1.02 (t, *J* = 7.5 Hz, 6H), 0.25 (s, 9H). MS calcd for $C_{123}H_{142}N_{18}O_{21}Si$ $[M+2H]^{2+}$ 1117.5178, found (HR-ESI) 1117.5178.

NO_2-Q<u>X</u>PP<u>Y</u>Q<u>X</u>PP-OMe (**4**) Compound **12** (8 mg, 2.7 μmol, 1 equiv.) was dissolved in a mixture of dry THF/MeOH 3:2 (1.25 mL) under N_2. $TMSCHN_2$ (soluz. 2M in hexane, 3 μL, 5.4μmol, 2 equiv.) was added dropwise and the solution stirred at r.t. for 2h. Few drops of acetic acid were added and the solution stirred for 5 min at r.t. Then the solution was diluted with CH_2Cl_2, washed with $NaHCO_3$, dried $MgSO_4$, filtered and concentrated. The crude product was purified by semi-prep RP-HPLC (5 mg, 62% yield).

^{1}H NMR (700 MHz, $CDCl_3$) δ 11.82 (s, 1H), 11.73 (s, 1H), 11.27 (s, 1H), 9.20 – 9.14 (m, 1H), 9.06 (t, *J* = 5.9 Hz, 1H), 9.02 (t, *J* = 5.8 Hz, 1H), 8.72 – 8.63 (m, 2H), 8.38 (dd, *J* = 6.4, 1.0 Hz, 1H), 8.37 (dd, *J* = 8.4, 1.4 Hz, 1H), 8.36 (d, *J* = 1.4 Hz, 1H), 8.11 (t, *J* = 4.5 Hz, 1H), 8.01 (d, *J* = 7.5, 1.4 Hz, 1H), 7.87 (s, 1H), 7.76 (dd, *J* = 9.5, 1.1 Hz, 1H), 7.75 (s, 1H), 7.74 (s, 1H), 7.67 (dd, *J* = 8.2, 0.8 Hz, 1H), 7.64 (s, 1H), 7.64 (s, 1H), 7.59 – 7.52 (m, 2H), 7.48 (dd, *J* = 8.3, 7.6 Hz, 1H), 7.24 – 7.18 (m, 2H), 7.17 (d, *J* = 2.1 Hz, 1H), 7.12-7.06 (m, 1H), 7.05 – 7.00 (m, 2H), 6.97 (d, *J* = 2.3 Hz, 1H), 6.90 (d, *J* = 2.1 Hz, 1H), 6.80 (d, *J* = 2.3 Hz, 1H), 6.79 (d, *J* = 2.3 Hz, 1H), 6.76 (d, *J* = 2.3 Hz, 1H), 6.71 (d, *J* = 2.4 Hz, 1H), 6.58 (d, *J* = 2.2 Hz, 1H), 4.82 (d, *J* = 5.9 Hz, 2H), 4.31-4.21 (m, 6H), 3.95 (d, *J* = 6.2 Hz, 2H), 3.87 (d, J = 5.6 Hz, 4H), 3.85-3.80 (m, 6H), 3.73 (d, *J* = 5.3 Hz, 3H), 3.57 (s, 3H), 2.33-2.18 (m, 2H), 2.15-1.97 (m, 2H), 1.75 (s, 9H), 1.69-1.61 (m, 2H), 1.65 (s, 9H), 1.51 – 1.35 (m, 8H), 1.25 (t, *J* = 8.2 Hz, 2H), 1.12 (d,

J = 6.7 Hz, 6H), 0.98 – 0.85 (m, 30H), 0.14 (s, 9H). MS calcd for $C_{121}H_{150}N_{18}O_{21}Si$ $[M+2H]^{2+}$ 1109.5491, found (HR-ESI) 1109.5482.

NO_2-QXQQYQXQQ-T-QQXQYQQXQ-NO_2 (**5a**) Compound **9** (12 mg, 5.36 μmol, 1 equiv.), 2,6-diisobutoxyterephthalohydrazide (**39**)[5] (0.91 mg, 2.27 μmol, 0.5 equiv.) and PyBOP (8.4 mg, 16 μmol, 3 equiv.) were dissolved in dry $CHCl_3$ under N_2. Then DIPEA (6 μL, 32 μmol, 6 equiv.) was added and the solution stirred at r.t. for 48 h. The solvent was removed and the crude purified by GPC. The product was recovered as a yellow solid (6.4 mg, 50% yield).

^{1}H NMR (500 MHz, $CDCl_3$) δ 11.49 (s, 2H), 11.24 (s, 2H), 10.98 (s, 2H), 10.89 (s, 2H), 10.86 (s, 2H), 10.78 (s, 2H), 10.51 (s, 2H), 10.23 (d, J = 9.3 Hz, 2H), 9.99 (d, J = 9.3 Hz, 2H), 8.27 (dd, J = 7.9, 1.4 Hz, 2H), 8.23 (dd, J = 7.2, 0.9 Hz, 2H), 8.10 (dd, J = 7.4, 0.9 Hz, 2H), 8.09 – 8.08 (d, J = 3.7 Hz, 2H), 7.98 (dd, J = 8.0, 1.2 Hz, 2H), 7.95 (dd, J = 7.4, 0.9 Hz, 2H), 7.89 (dd, J = 8.0, 1.2 Hz, 2H), 7.86 (dd, J = 7.0, 0.9 Hz, 2H), 7.78 (dd, J = 8.1, 1.2 Hz, 2H), 7.75 (dd, J = 8.1, 1.1 Hz, 2H), 7.70 (dd, J = 8.1, 1.1 Hz, 2H), 7.61 (dd, J = 8.2, 1.2 Hz, 2H), 7.60 (s, 2H), 7.48 (t, J = 7.5 Hz, 4H), 7.30 (t, J = 7.5 Hz, 2H), 7.25-7.19 (m, 4H), 7.10 (s, 2H), 7.09-7.06 (m, 4H), 7.06 (d, J = 1.3 Hz, 2H), 7.04 – 6.98 (m, 4H), 6.90 (t, J = 7.7 Hz, 2H), 6.87 (s, 2H), 6.72 (s, 2H), 6.60 (s, 2H), 6.47 (d, J = 1.7 Hz, 2H), 6.43 (d, J = 1.9 Hz, 2H), 6.41 (s, 2H), 5.88 (s, 2H), 5.79 (s, 2H), 4.10 – 4.00 (m, 8H), 3.95-3.89 (m, 4H), 3.90 – 3.84 (m, 2H), 3.83 – 3.74 (m, 8H), 3.68-3.58 (m, 6H), 3.50 (t, J = 7.5 Hz, 2H), 3.21 (dd, J = 16.4, 3.4 Hz, 2H), 2.50-2.42 (m, 2H), 2.39 – 2.14 (m, 12H), 1.81 (d, J = 11.1 Hz, 4H), 1.61 (s, 18H), 1.46 (s, 18H), 1.31 (d, J = 6.7 Hz, 12H), 1.25 – 1.11 (m, 48H), 1.08 (d, J = 6.8 Hz, 12H), 1.00 (d, J = 6.7 Hz, 12H), 0.10 (s, 18H), (mixture of two diastereomers PM and PP/MM and their ratio is 8:2, only the major peaks are reported). MS calcd for $C_{264}H_{284}N_{40}O_{44}Si_2$ $[M+2H]^{2+}$ 2387.0371, found (HR-ESI) 2387.0427.

NO_2-QXQPYQXPQ-T-QPXQYPQXQ-NO_2 (**6a**) Compound **10** (11 mg, 5.18 μmol, 1 equiv.), **39**[5] (0.87 mg, 2.59 μmol, 0.5 equiv.) and PyBOP (8.1 mg, 15.5 μmol, 3 equiv.) were dissolved in dry $CHCl_3$ under N_2. Then DIPEA (6 μL, 31 μmol, 6 equiv.) was added and the solution stirred at r.t. for 48 h. The solvent was removed and the crude purified by GPC. The product was recovered as a yellow solid (5 mg, 40% yield).

^{1}H NMR (300 MHz, $CDCl_3$) δ 11.39 (s, 2H), 11.32 (s, 2H), 11.06 (s, 2H), 11.05 (s, 2H), 10.79 (s, 2H), 10.44 (d, J = 9.1 Hz, 2H), 10.03 (d, J = 9.2 Hz, 2H), 8.77 (t, J = 5.0 Hz, 2H), 8.31 (dd, J = 7.5, 0.8 Hz, 2H), 8.28-8.23 (m, 4H), 8.22 (dd, J = 8.3, 1.4 Hz, 2H), 8.17 (dd, J = 7.5, 0.8 Hz, 2H), 8.10 (d, J = 7.8 Hz, 2H), 8.06 (dd, J = 7.5, 0.8 Hz, 2H), 7.82 (dd, J = 8.3, 1.0 Hz, 2H),

7.78 (dd, J = 8.3, 1.0 Hz, 2H), 7.75-7.70 (m, 2H), 7.68 (dd, J = 8.2, 1.3 Hz, 2H), 7.64-7.51 (m, 4H), 7.55 (s, 2H), 7.45 (s, 2H), 7.43-7.32 (m, 4H), 7.24 (dd, J = 8.2, 1.3 Hz, 2H), 7.15 (s, 2H), 7.14-7.03 (m, 4H), 7.02-6.91 (m, 4H), 6.88 – 6.81 (m, 2H), 6.63-6.59 (m, 2H), 6.58 (s, 2H), 6.57 (s, 2H), 6.48 (s, 2H), 6.47-6.40 (m, 2H), 6.29 (d, J = 1.8 Hz, 2H), 6.15 (d, J = 1.6 Hz, 1H), 6.09 (d, J = 2.1 Hz, 2H), 5.63 (s, 1H), 5.62 (s, 2H), 4.59 – 4.27 (m, 4H), 4.27 – 4.06 (m, 12H), 4.06-3.93 (m, 4H), 3.92 – 3.74 (m, 10H), 3.74 – 3.61 (m, 4H), 3.57 (dd, J = 16.3, 2.3 Hz, 2H), 3.50-3.34 (m, 4H), 3.10 (dd, J = 16.3, 4.0 Hz, 2H), 2.99 (dd, J = 16.4, 2.3 Hz, 2H), 2.51 (dt, J = 13.3, 6.7 Hz, 4H), 2.41 – 2.10 (m, 12H), 1.74 (d, J = 8.3 Hz, 12H), 1.61 (s, 18H), 1.55 (s, 18H), 1.38 – 1.19 (m, 36H), 1.19 – 1.00 (m, 32H), 0.99 – 0.88 (m, 12H), 0.08 (s, 18H), (mixture of two diastereomers PM and PP/MM and their ratio is 7:3, only the major peaks are reported). MS calcd for $C_{260}H_{300}N_{40}O_{44}Si_2$ $[M+2H]^{2+}$ 2371.0997, found (HR-ESI) 2371.1036.

NO_2-Q<u>X</u>PQ<u>Y</u>Q<u>X</u>PQ-T-QP<u>X</u>Q<u>Y</u>QP<u>X</u>Q-NO_2 (7a) Compound **10** (13 mg, 5.8 μmol, 1 equiv.), **39**[5] (0.93 mg, 2.7 μmol, 0.5 equiv.) and PyBOP (9 mg, 17.5 μmol, 3 equiv.) were dissolved in dry $CHCl_3$ under N_2. Then DIPEA (6 μL, 35 μmol, 6 equiv.) was added and the solution stirred at r.t. for 48 h. The solvent was removed and the crude purified by GPC. The product was recovered as a yellow solid (6 mg, 42% yield).

^{1}H NMR (500 MHz, $CDCl_3$) δ 11.14 (s br, 2H), 11.05 (s br, 2H), 10.95 (s, 2H), 10.90 (s, 2H), 10.67 (s br, 2H), 10.39 (d, J = 8.6 Hz, 2H), 10.02 (d, J = 8.6 Hz, 2H), 8.79 (s, 2H), 8.31 (d, J = 8.1 Hz, 2H), 8.21-8.09 (m, 2H), 8.15 (m, 4H), 7.91 (d, J = 8.1 Hz, 2H), 7.83 (d, J = 8.0 Hz, 2H), 7.75 – 7.67 (m, 4H), 7.57 (s br, 2H), 7.48 (m, 2H), 7.30 (t, J = 7.8 Hz, 2H), 7.19-7.11 (m, 4H), 7.07-6.96 (m, 4H), 6.74 (s, 2H), 6.70 (s, 2H), 6.57 (s, 2H), 6.41 (s, 2H), 6.25 (s, 2H), 6.22 (s, 2H), 4.51 (d, J = 14.8 Hz, 2H), 4.37 (broad signal, 2H), 4.20 (s br, 4H), 4.09-3.99 (m, 4H), 3.98-3.78 (m, 12H), 3.74 (s br, 4H), 3.26 (d, J = 16.1 Hz, 2H), 3.14-3.07 (m, 2H), 2.44-2.27 (m, 4H), 2.27-2.17 (m, 4H), 2.07-2.00 (m, 4H), 1.83-1.71 (m, 4H), 1.80-1.58 (m, 16H), 1.42 (s, 18H), 1.33 (s, 18H), 1.30-1.20 (m, 24H), 1.10-1.19 (m, 24H), 1.08-0.98 (m, 24H), 0.91-0.85 (m, 4H), 0.07 (s, 18H). MS calcd for $C_{260}H_{300}N_{40}O_{44}Si_2$ $[M+2H]^{2+}$ 2371.0997, found (HR-ESI) 2371.1022.

NO_2-Q<u>X</u>PP<u>Y</u>Q<u>X</u>PP-T-PP<u>X</u>Q<u>Y</u>PP<u>X</u>Q-NO_2 (8a) Compound **12** (10 mg, 4.53 μmol, 1 equiv.), **39**[5] (0.76 mg, 2.27 μmol, 0.5 equiv.) and PyBOP (7 mg, 13.6 μmol, 3 equiv.) were dissolved in dry $CHCl_3$ under N_2. Then DIPEA (5 μL, 27 μmol, 6 equiv.) was added and the solution stirred at r.t. for 48 h. The solvent was removed and the crude purified by GPC. The product was recovered as a yellow solid (7 mg, 66% yield).

^1H NMR (300 MHz, $CDCl_3$) δ 11.94 (s, 2H), 11.78 (s, 2H), 11.24 (s, 2H), 10.73 (d, *J* = 5.5 Hz, 2H), 10.35 (d, *J* = 6.6 Hz, 2H), 9.31 (t, *J* = 5.0 Hz, 2H), 9.07 (t, *J* = 6.0 Hz, 2H), 8.99 (t, *J* = 5.0 Hz, 1H), 8.65 (d, *J* = 7.6 Hz, 2H), 8.60 (t, *J* = 5.9 Hz, 2H), 8.51 (d, *J* = 7.3 Hz, 2H), 8.35 (dd, *J* = 8.4, 1.3 Hz, 2H), 8.08 (m, 2H), 8.00 (dd, *J* = 7.5, 1.2 Hz, 2H), 7.85 (s, 2H), 7.79-7.74 (m, 2H), 7.73 (s, 2H), 7.71 (s, 1H), 7.60 (m, 2H), 7.60 (s, 2H), 7.52 (d, *J* = 8.1 Hz, 2H), 7.49 – 7.43 (m, 2H), 7.26 (m, overlap with solvent signal, 2H), 7.09-7.05 (m, 4H), 7.05-7.01 (m, 4H), 6.99 (d, *J* = 2.3 Hz, 2H), 6.87 (d, *J* = 1.8 Hz, 2H), 6.77 (d, *J* = 2.2 Hz, 2H), 6.62 (d, *J* = 2.0 Hz, 2H), 6.57 (d, *J* = 2.1 Hz, 2H), 4.87 (d, *J* = 5.4 Hz, 4H), 4.31 (s br, 4H), 4.26 – 4.18 (m, 8H), 3.98 – 3.90 (m, 8H), 3.88 (d, *J* = 5.6 Hz, 4H), 3.85 – 3.79 (m, 12H), 3.72 (d, *J* = 5.4 Hz, 8H), 2.38 – 2.18 (m, 8H), 2.06 – 1.96 (m, 6H), 1.69 (s, 18H), 1.63 (s, 18H), 1.33 (m, 16H), 1.23 – 1.16 (m, 16H), 1.12 (d, *J* = 6.8 Hz, 12H), 1.09 (d, *J* = 6.7 Hz, 12H), 0.95 – 0.87 (m, 48H), 0.84 (t, *J* = 7.4 Hz, 12H), 0.10 (s, 18H). MS calcd for $C_{256}H_{316}N_{40}O_{44}Si_2$ $[M+2H]^{2+}$ 2355.1623, found (HR-ESI) 2355.1619.

NO_2-QXQQYQXQQ-T-QQXQYQQXQ-NO_2 (5b)

Compound **5a** (3 mg, 0.6 μmol) was treated with a 50% solution of TFA in CH_2Cl_2 (2 mL) at r.t. for 4 h. Then the solvent was removed under vacuum obtaining the product as a yellow solid (quantitative yield). Analytical data in agreement with previously reported data for this compound.[5]

NO_2-QXQPYQXPQ-T-QPXQYPQXQ-NO_2 (6b)

Compound **6a** (3 mg, 0.6 μmol) was treated with a 50% solution of TFA in CH_2Cl_2 (2 mL) at r.t. for 2 h. Then the solvent was removed under vacuum obtaining the product as a yellow solid (quantitative yield).

^1H NMR (500 MHz, $CDCl_3$) δ 12.02 (s, 2H), 11.49 (s, 2H), 11.44 (s, 2H), 10.28 (s, 2H), 10.15 (s, 2H), 10.08 (s, 4H), 10.03 (d, *J* = 9.0 Hz, 2H), 9.90 (d, *J* = 9.0 Hz, 2H), 9.40 (s, 2H), 8.75 (s br, 2H), 8.68 (m, 2H), 8.54 (d, *J* = 7.6 Hz, 2H), 8.51 (s, 2H), 8.24 (dd, *J* = 8.2, 1.4 Hz, 2H), 8.21 (dd, *J* = 7.3, 1.1 Hz, 2H), 8.00 (d, *J* = 7.9 Hz, 2H), 7.99 (d, *J* = 7.5 Hz, 2H), 7.96 (d, *J* = 8.3 Hz, 2H), 7.94 – 7.92 (m, 2H), 7.89 (dd, *J* = 7.9, 1.0 Hz, 2H), 7.83 (d, *J* = 8.2 Hz, 1H), 7.61 (s, 2H), 7.60 (d, *J* = 1.7 Hz, 2H), 7.54 (t, *J* = 7.7 Hz, 2H), 7.46 (s, 2H), 7.44-7.40 (m, 4H), 7.26-7.24 (overlap with solvent peak), 7.22-7.19 (m, 4H), 7.10 (s, 2H), 7.08 – 7.05 (m, 4H), 6.85 (d, *J* = 2.1 Hz, 2H), 6.71 (d, *J* = 1.6 Hz, 2H), 6.36 (s, 2H), 6.07 (d, *J* = 2.2 Hz, 2H), 5.15 (s, 2H), 4.68 (s, 2H), 4.50 (d, *J* = 15.2 Hz, 2H), 4.18 – 4.02 (m, 12H), 4.00 – 3.94 (m, 4H), 3.87-3.81 (m, 8H), 3.78 (d, *J* = 14.4 Hz, 2H), 3.69 (d, *J* = 15.1 Hz, 2H), 3.60 (t, *J* = 6.5 Hz, 2H), 3.53 (t, *J* = 7.9 Hz, 2H), 3.37 (d, *J* = 15.7 Hz, 2H), 3.16 (t, *J* = 6.8 Hz, 2H), 2.49 – 2.43 (m, 2H), 2.40

– 2.29 (m, 4H), 2.27-2.20 (m, 2H), 2.07 – 1.99 (m, 2H), 1.93 – 1.85 (m, 4H), 1.79 – 1.70 (m, 8H), 1.68 – 1.60 (m, overlap with solvent peak), 1.39 (d, *J* = 6.7 Hz, 6H), 1.30 (d, *J* = 6.7 Hz, 6H), 1.28 – 1.17 (m, 36H), 1.17 – 1.10 (m, 24H), 1.00 (d, *J* = 6.6 Hz, 6H), 0.83 (d, *J* = 6.7 Hz, 6H). MS calcd for $C_{234}H_{244}N_{40}O_{44}$ $[M+2H]^{2+}$ 2158.9037, found (HR-ESI) 2158.9046.

NO_2-QXPQYQXPQ-T-QPXQYQPXQ-NO_2 (7b)

Compound **7b** (3 mg, 0.6 μmol) was treated with a 50% solution of TFA in CH_2Cl_2 (2 mL) at r.t. for 6 h. Then the solvent was removed under vacuum. The pure compound was obtained after GPC purification as a yellow solid (1 mg, 38% yield).

^{1}H NMR (500 MHz, $CDCl_3$) δ 11.21 (s, 2H), 10.95 (s, 2H), 10.85 (s, 2H), 10.40 (s, 2H), 10.38 (s, 2H), 10.14 (s, 2H), 10.10 (s, 2H), 9.91 (d, *J* = 9.0 Hz, 2H), 9.79 (d, *J* = 9.0 Hz, 2H), 9.48 (s, 2H), 9.38 – 9.31 (m, 2H), 8.77 (d, *J* = 6.2 Hz, 2H), 8.61 (s, 2H), 8.47 (d, *J* = 7.3 Hz, 2H), 8.33 (dd, *J* = 8.1, 0.9 Hz, 2H), 8.26 (t, *J* = 5.0 Hz, 2H), 8.23 (d, *J* = 7.2 Hz, 2H), 8.07-8.02 (m, 8H), 7.97 (d, *J* = 7.9 Hz, 2H), 7.95 – 7.89 (m, 6H), 7.82 (d, *J* = 7.3 Hz, 2H), 7.78 (d, *J* = 7.9 Hz, 2H), 7.50-7.45 (m, 8H), 7.42 (s, 2H), 7.37 – 7.28 (m, 8H), 7.24 – 7.18 (m, 4H), 7.14-7.12 (m, 4H), 7.10 (s, 2H), 7.09 (d, *J* = 1.7 Hz, 2H), 7.08 (s, 2H), 7.05 (s, 2H), 7.03 (s, 2H), 6.99 (d, *J* = 2.3 Hz, 2H), 6.98 (s, 2H), 6.60 (d, *J* = 2.0 Hz, 2H), 6.11 (s, 2H), 5.91 (s, 2H), 5.58 (s, 2H), 5.46 (d, *J* = 2.2 Hz, 2H), 5.07 (s, 2H), 4.55-4.52 (m, 2H), 4.38 (dd, *J* =12, 1.0 Hz, 1H), 4.32 (d, *J* = 6.5 Hz, 1H), 4.29 (d, *J* = 6.6 Hz, 1H), 4.26 – 4.19 (m, 6H), 4.18-4.11 (m, 6H), 4.08 (s, 2H), 4.06-3.97 (m, 8H), 3.86 (t, *J* = 6.4 Hz, 2H), 3.83-3.79 (m, 2H), 3.77 –3.60 (m, 6H), 3.52 – 3.48 (m, 4H), 2.55-2.48 (s, 4H), 2.46 – 2.38 (m, 8H), 2.35 (t, *J* = 7.5 Hz, 6H), 2.29-2.24 (m, 12H), 1.96-1.90 (m, 6H), 1.83 – 1.77 (m, 6H), 1.68-1.46 (m, 24H), 1.37-1.09 (m, 48H). MS calcd for $C_{234}H_{244}N_{40}O_{44}$ $[M+2H]^{2+}$ 2158.9037, found (HR-ESI) 2158.9044.

NO_2-QXPPYQXPP-T-PPXQYPPXQ-NO_2 (8b)

Compound **8b** (6 mg, 1.1 μmol) was treated with a 50% solution of TFA in CH_2Cl_2 (2 mL) at r.t. for 2 h. Then the solvent was removed under vacuum, the residue suspended in CH_3CN/H_2O and freeze-dried. The product was obtained as a yellow powder (quantitative yield).

^{1}H NMR (500 MHz, $CDCl_3$) δ 11.22 (s, 4H), 10.33 (s, 2H), 10.19 (s, 2H), 10.14 (d, *J* = 8.9 Hz, 2H), 10.04 – 9.97 (m, 4H), 9.49 (s, 2H), 9.32 (d, *J* = 6.1 Hz, 2H), 8.86 (s, 2H), 8.41 – 8.35 (m, 4H), 8.29 (d, *J* = 9.0 Hz, 2H), 8.21 (d, *J* = 7.2 Hz, 2H), 8.17 (d, *J* = 7.4 Hz, 2H), 8.01 (d, *J* = 2.1 Hz, 2H), 7.97 – 7.87 (m, 8H), 7.84 (d, *J* = 7.9 Hz, 2H), 7.65 (s, 2H), 7.53 (s, 2H), 7.50 (d, , *J* = 7.3, 1.5 Hz, 2H), 7.47 (s, 2H), 7.40 (d, *J* = 7.2 Hz, 2H), 7.32 (s, 2H), 7.29-7.26 (m, overlap with solvent peak), 7.20 – 7.16 (m, 6H), 7.10 (d, *J* = 2.3 Hz, 2H), 7.05 (s, 2H), 7.00 (t, *J* = 7.7 Hz, 2H), 6.88-6.84 (m, 4H), 6.66 (s, 2H), 6.63 (s, 2H), 6.49 (s, 2H), 5.97 (d, *J* = 2.2 Hz, 2H),

5.24 (d, J = 1.8 Hz, 2H), 4.45 (d, J = 14.7 Hz, 2H), 4.29 – 3.92 (m, 20H), 3.76 – 3.64 (m, 10H), 3.62-3.56 (m, 8H), 3.53 – 3.42 (m, 4H), 3.34 (d, J = 15.8 Hz, 2H), 2.54-2.48 (m, 2H), 2.40-2.33 (m, 4H), 2.15-2.07 (m, 4H), 1.90 – 1.80 (m, 12H), 1.79 – 1.70 (m, 12H), 1.70-1.58 (m, overlap with water signal), 1.35 (d, J = 6.7 Hz, 6H), 1.32 (d, J = 6.7 Hz, 6H), 1.27 – 1.17 (m, 24H), 1.17 – 1.10 (m, 24H), 1.06 – 0.98 (m, 12H), 0.89 (t, J = 6.5 Hz, 12H). Only the peaks corresponding to the folded species are reported. It is too difficult to analyze the spectrum as the compound presents in solution as a mixture of conformations. MS calcd for $C_{230}H_{260}N_{40}O_{44}$ $[M+2H]^{2+}$ 2142.9663, found (HR-ESI) 2142.9691.

6. References

1. Rigaku Oxford Diffraction (2015). CrysAlis PRO. Rigaku Oxford Diffraction, Yarnton, England.
2. G. Sheldrick, *Acta Crystallogr.*, 2015, **71**, 3-8.
3. O. V. Dolomanov, L. J. Bourhis, R. J. Gildea, J. A. K. Howard and H. Puschmann, *J. Appl. Cryst.*, 2009, **42**, 339-341.
4. C. F. Macrae, P. R. Edgington, P. McCabe, E. Pidcock, G. P. Shields, R. Taylor, M. Towler and J. van de Streek, *J. Appl. Cryst.*, 2006, **39**, 453-457.
5. S. De, B. Chi, T. Granier, T. Qi, V. Maurizot and I. Huc, *Nat. Chem.*, 2018, **10**, 51-57.
6. L. Pellegatti, J. Zhang, B. Drahos, S. Villette, F. Suzenet, G. Guillaumet, S. Petoud and E. Toth, *Chem. Commun.*, 2008, 6591-6593.
7. L. Grehn and U. Ragnarsson, *Synthesis*, 1987, **1987**, 275-276.
8. T. Qi, T. Deschrijver and I. Huc, *Nat. Protoc.*, 2013, **8**, 693.
9. B. Baptiste, C. Douat-Casassus, K. Laxmi-Reddy, F. Godde and I. Huc, *J. Org. Chem.*, 2010, **75**, 7175-7185.
10. X. Hu, S. J. Dawson, Y. Nagaoka, A. Tanatani and I. Huc, *J. Org. Chem.*, 2016, **81**, 1137-1150.
11. M. Mergler, R. Nyfeler and J. Gosteli, *Tetrahedron Lett.*, 1989, **30**, 6741-6744.

7. ^{1}H and ^{13}C NMR spectra of new compounds

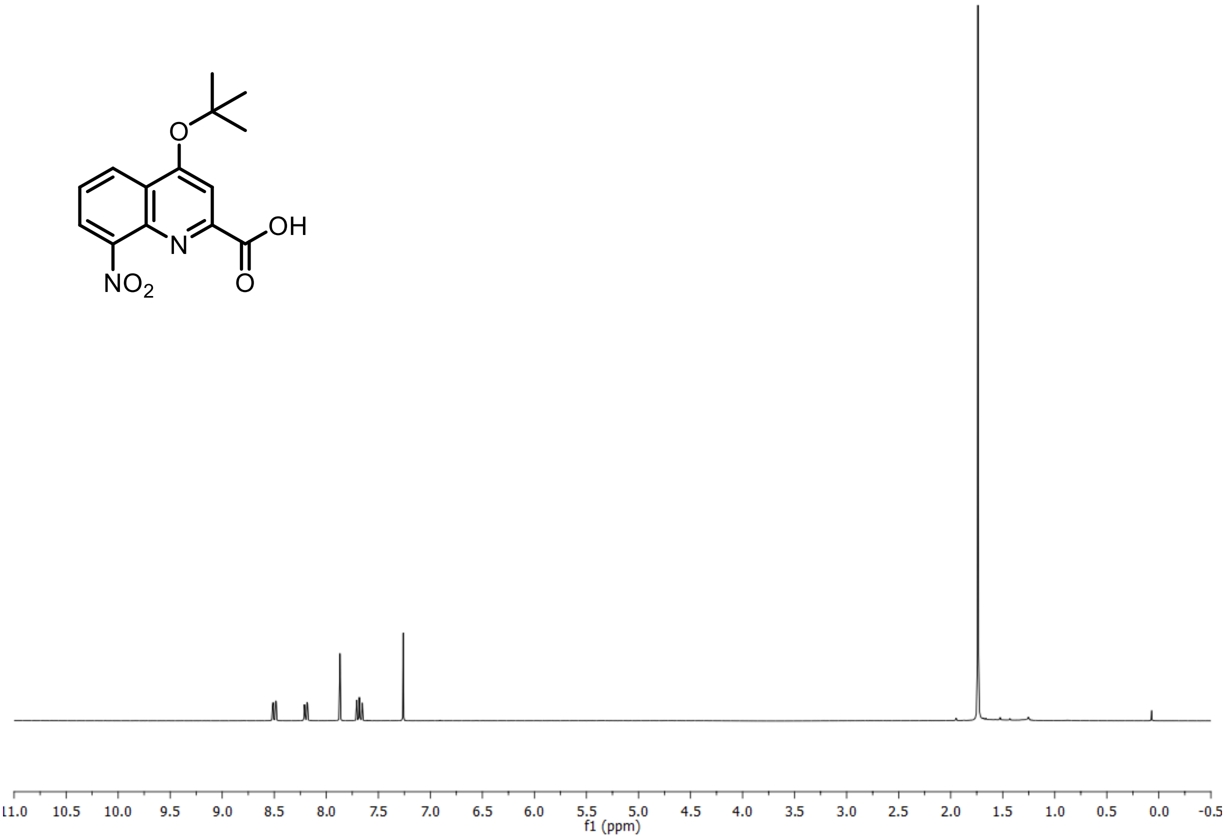

Figure S33 ^{1}H NMR spectrum (300 MHz, $CDCl_3$) of **14**.

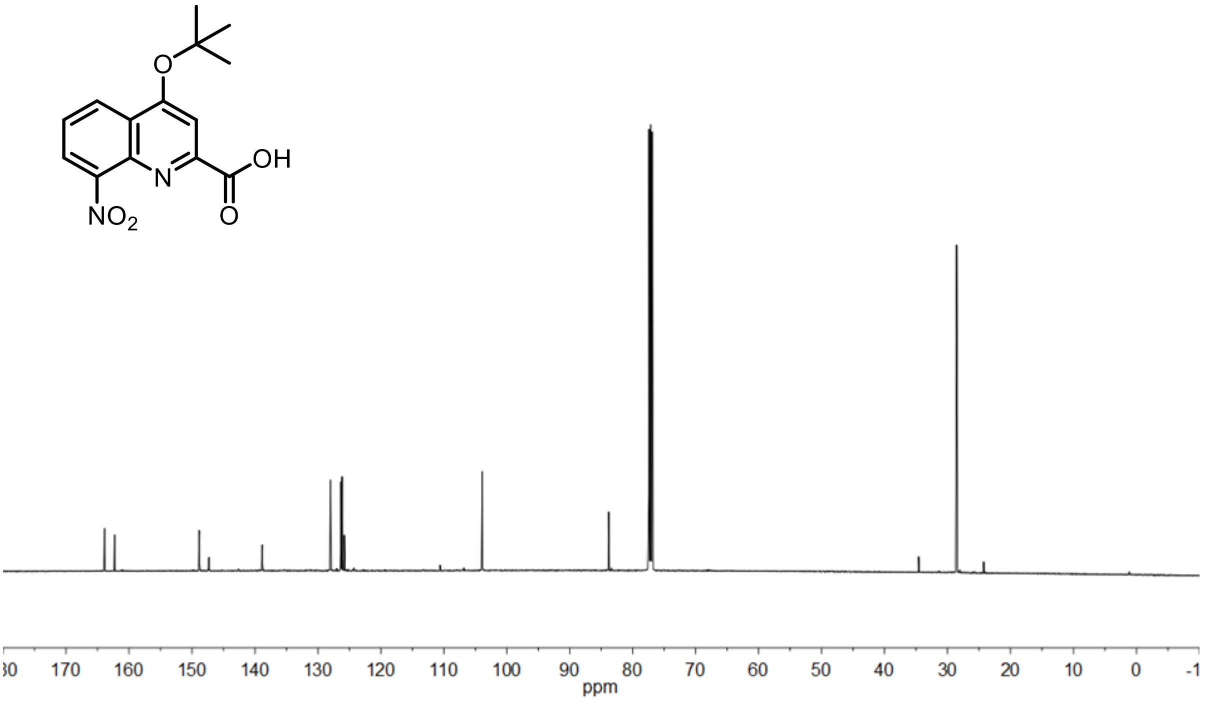

Figure S34 ^{13}C NMR spectrum (75 MHz, $CDCl_3$) of **14**.

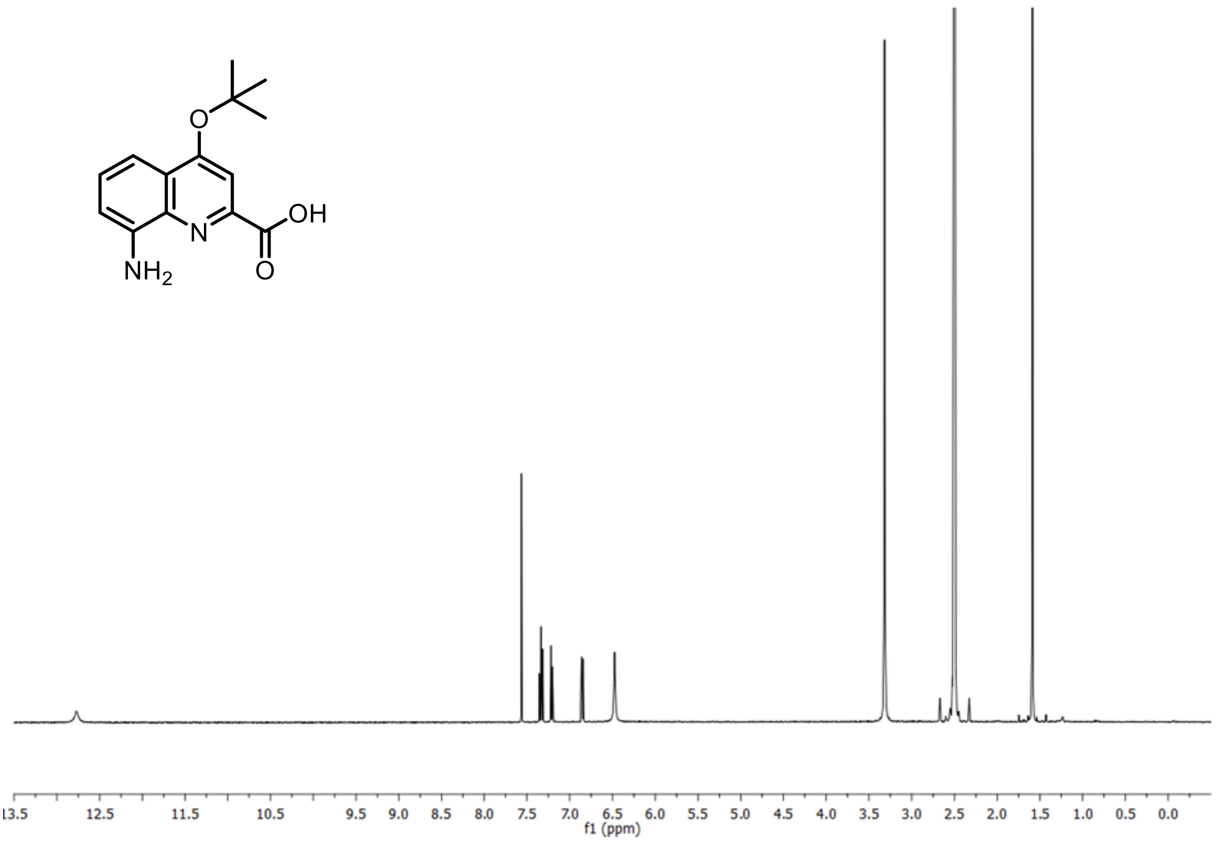

Figure S35 ^{1}H NMR spectrum (400 MHz, DMSO-d_6) of **15**.

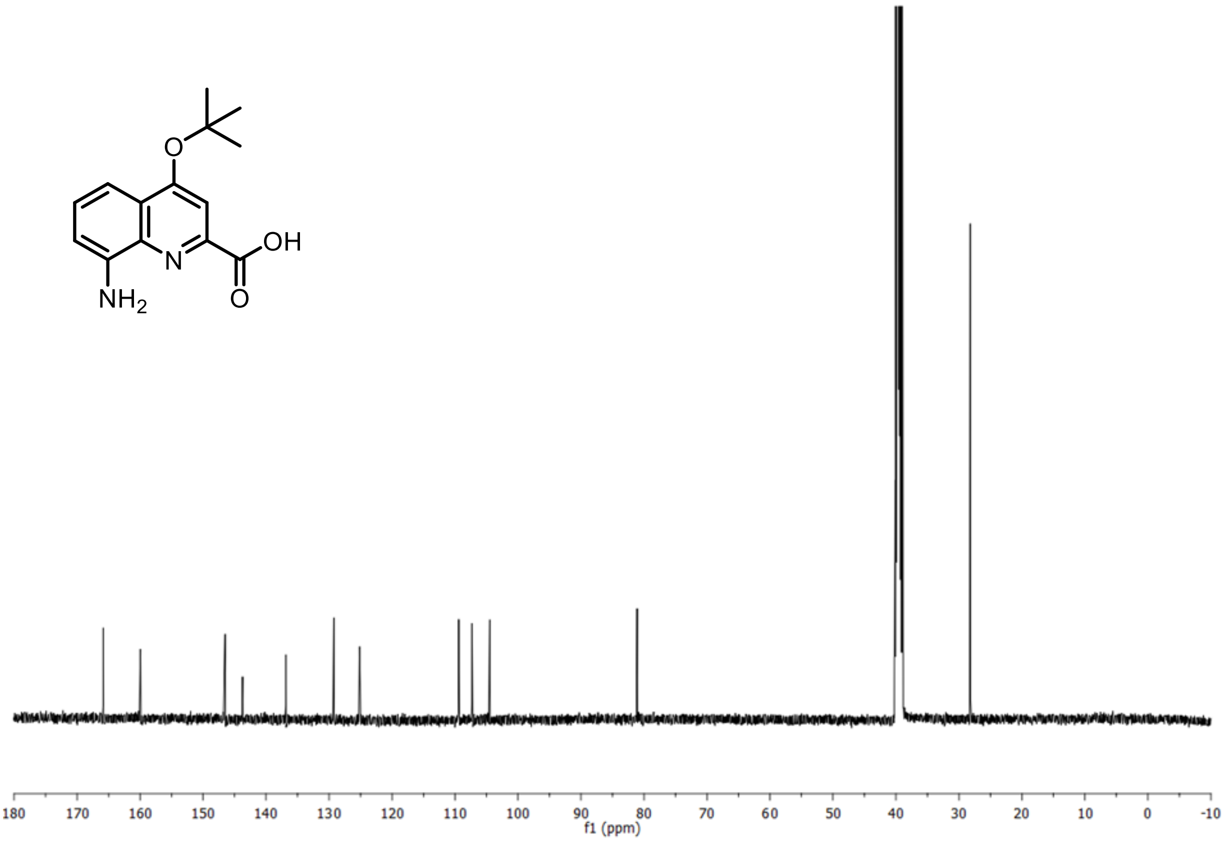

Figure S36 ^{13}C NMR spectrum (156 MHz, DMSO-d_6) of **15**.

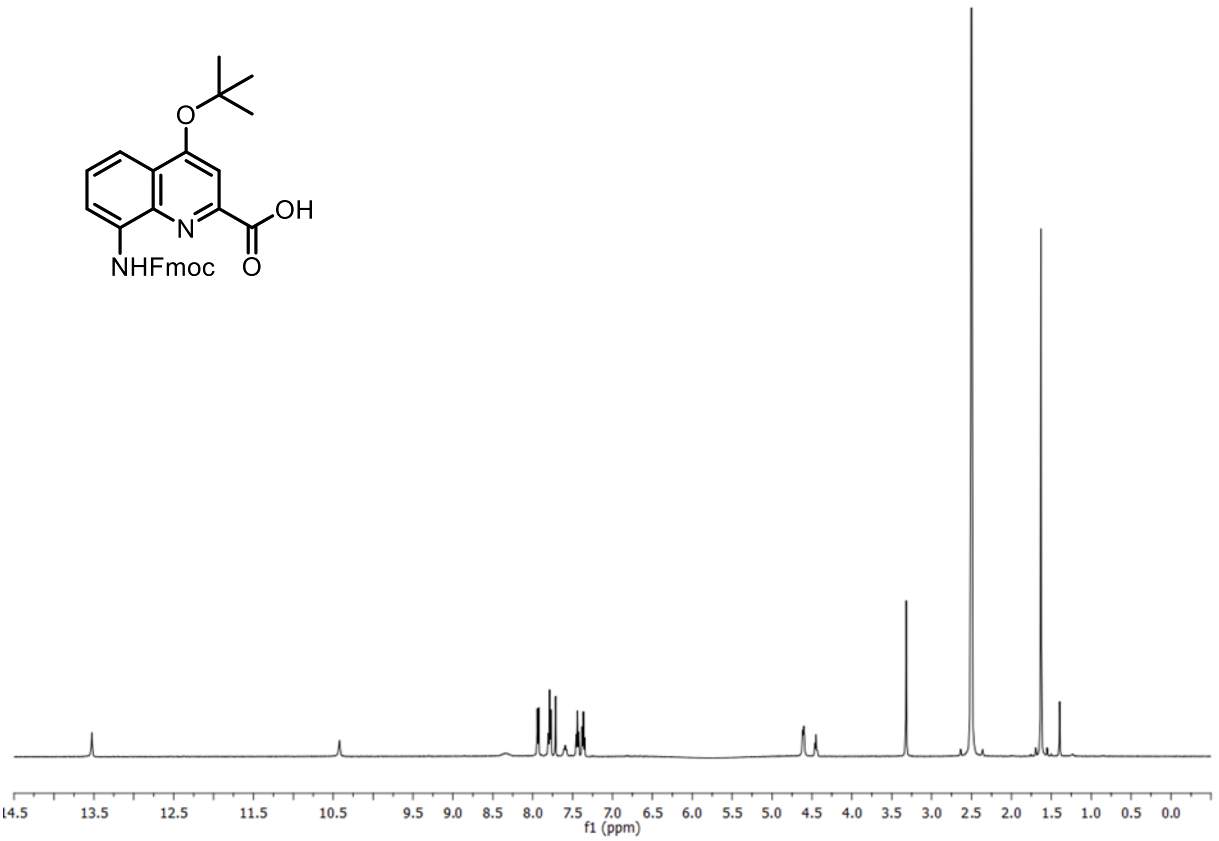

Figure S37 ^{1}H NMR spectrum (300 MHz, DMSO-d_6) of **16**.

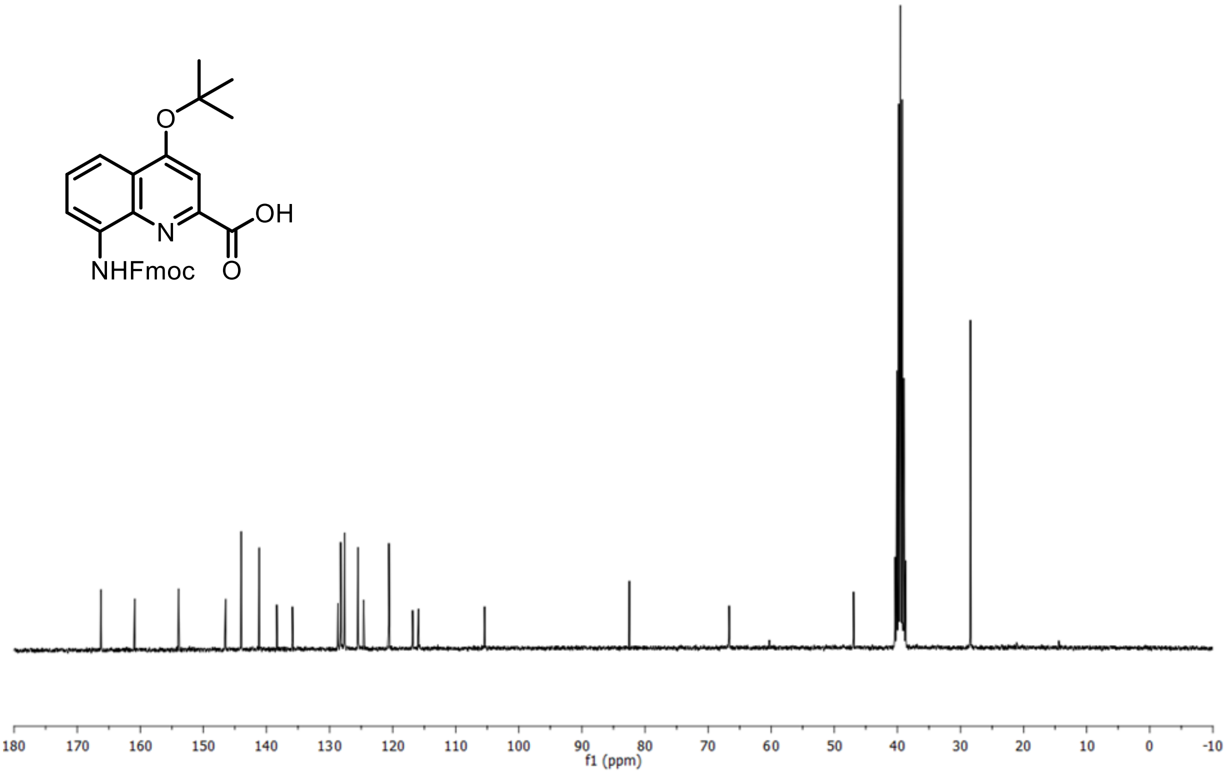

Figure S38 ^{13}C NMR spectrum (75 MHz, DMSO-d_6) of **16**.

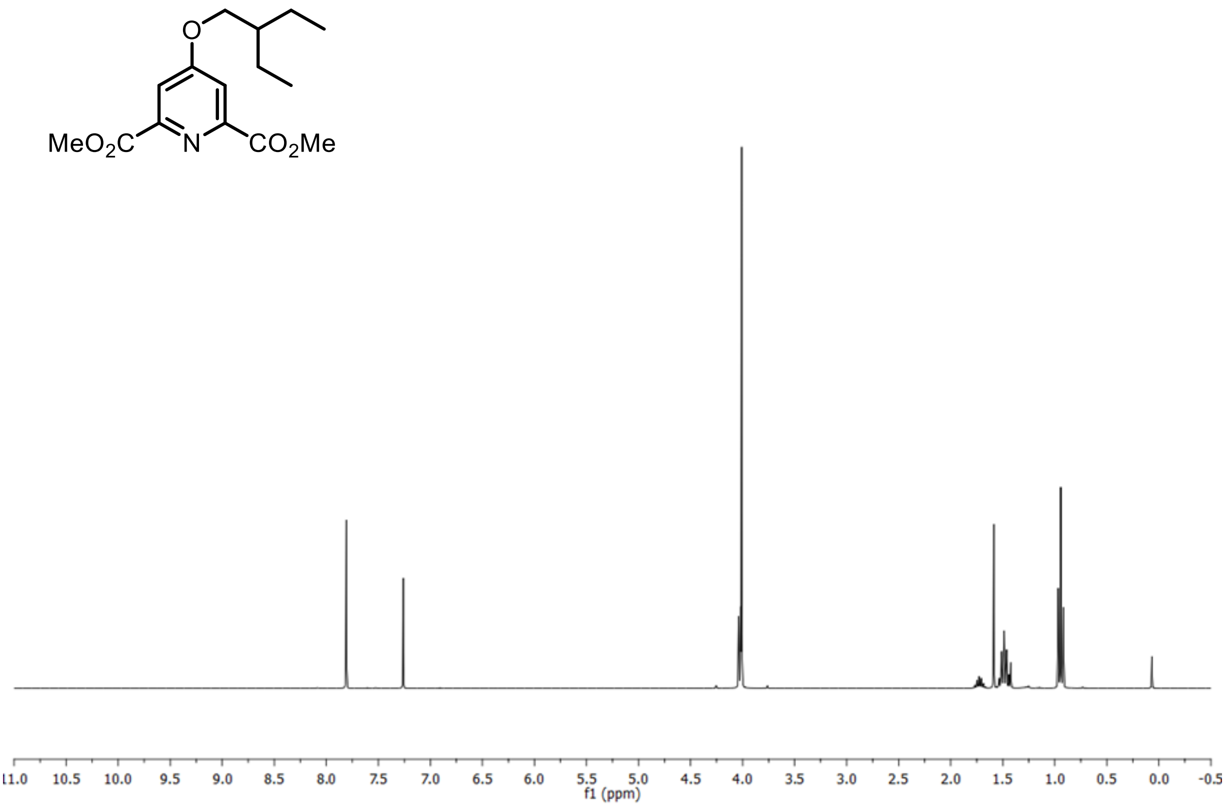

Figure S39 ^{1}H NMR spectrum (300 MHz, $CDCl_3$) of **18**.

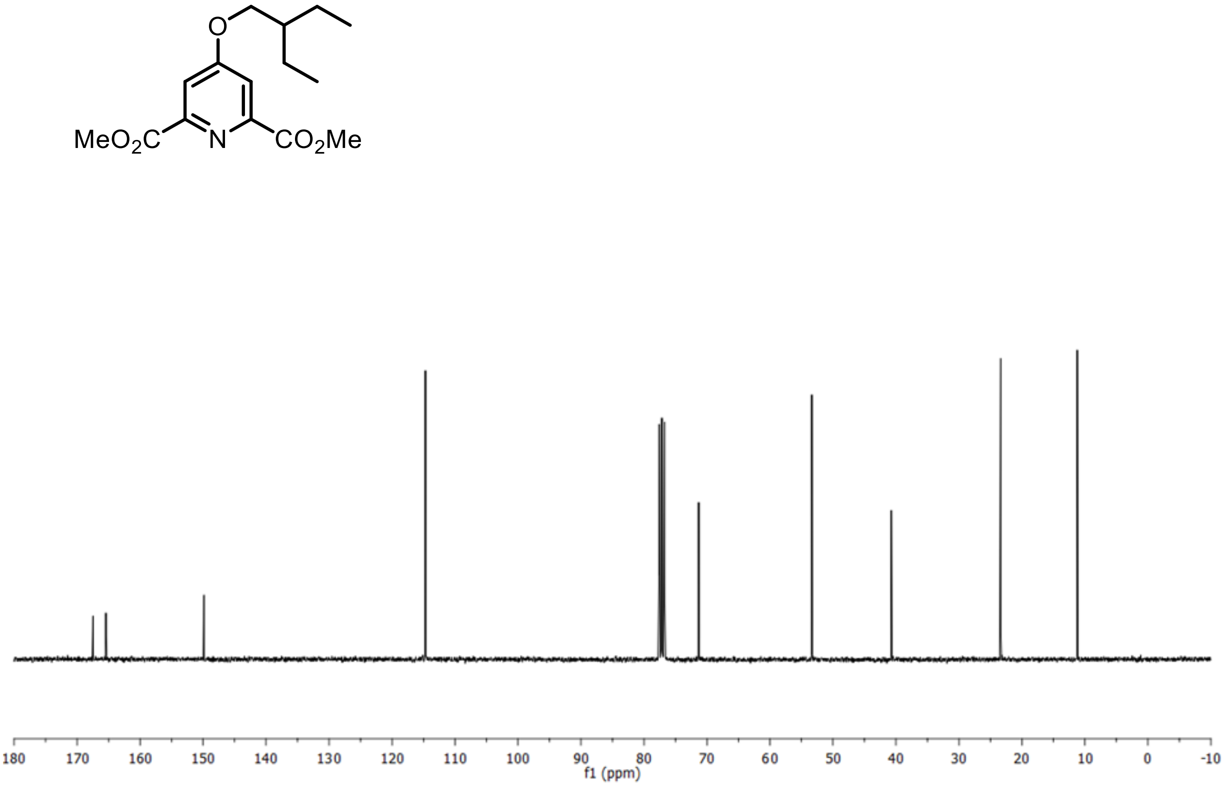

Figure S40 ^{13}C NMR spectrum (75 MHz, $CDCl_3$) of **18**.

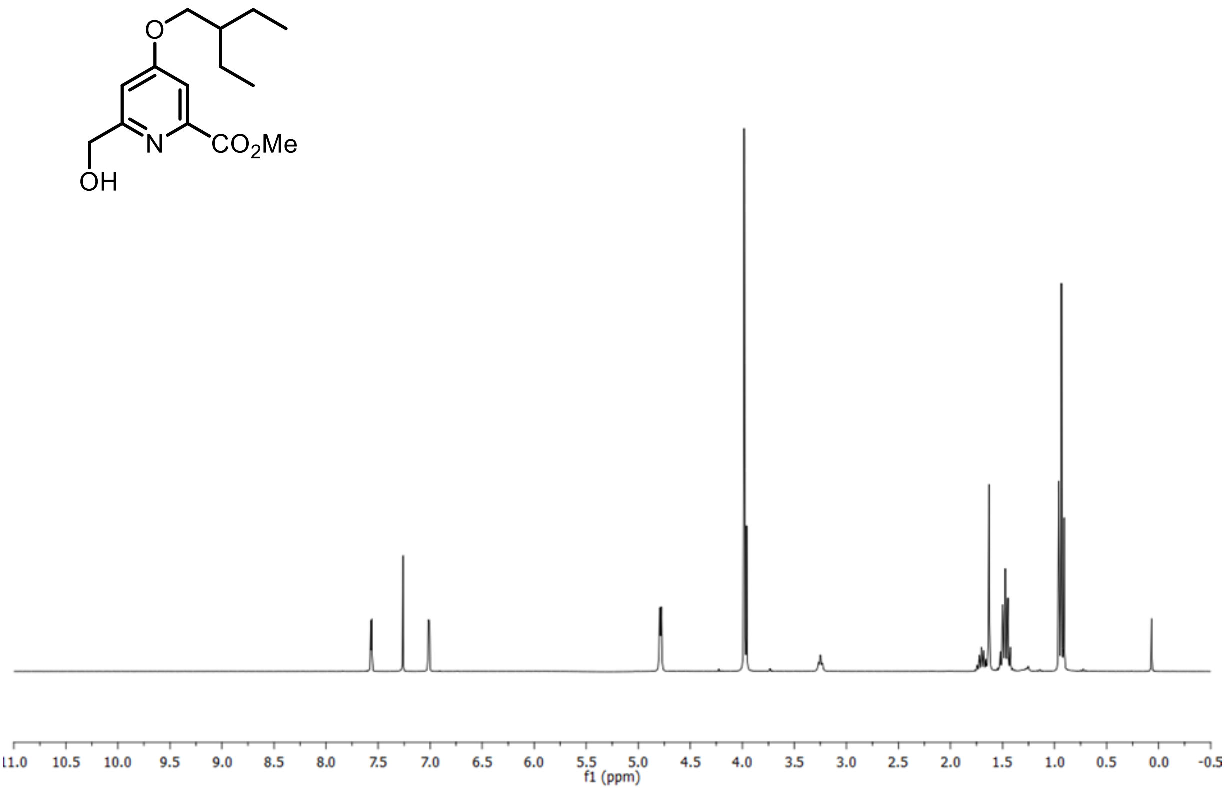

Figure S41 ^{1}H NMR spectrum (300 MHz, $CDCl_3$) of **19**.

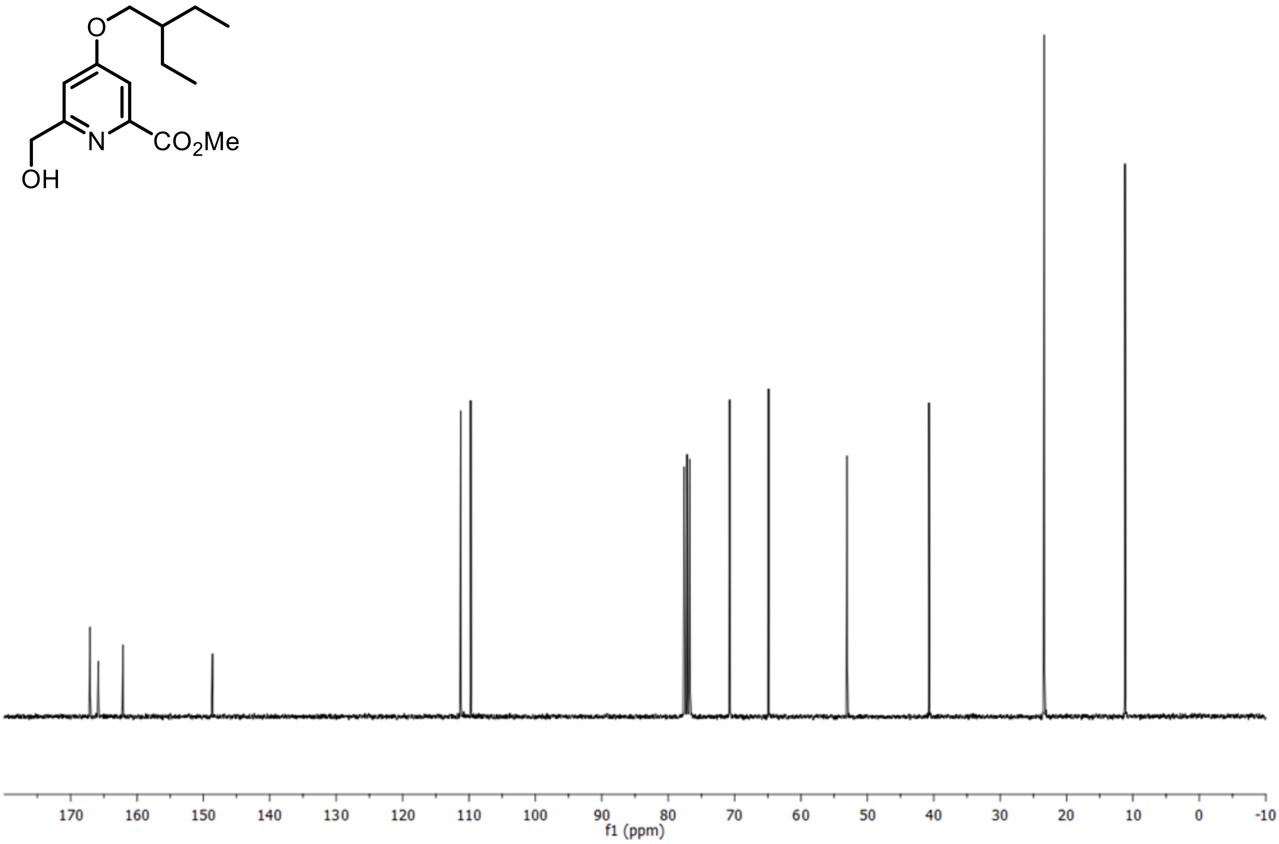

Figure S42 ^{13}C NMR spectrum (75 MHz, $CDCl_3$) of **19**.

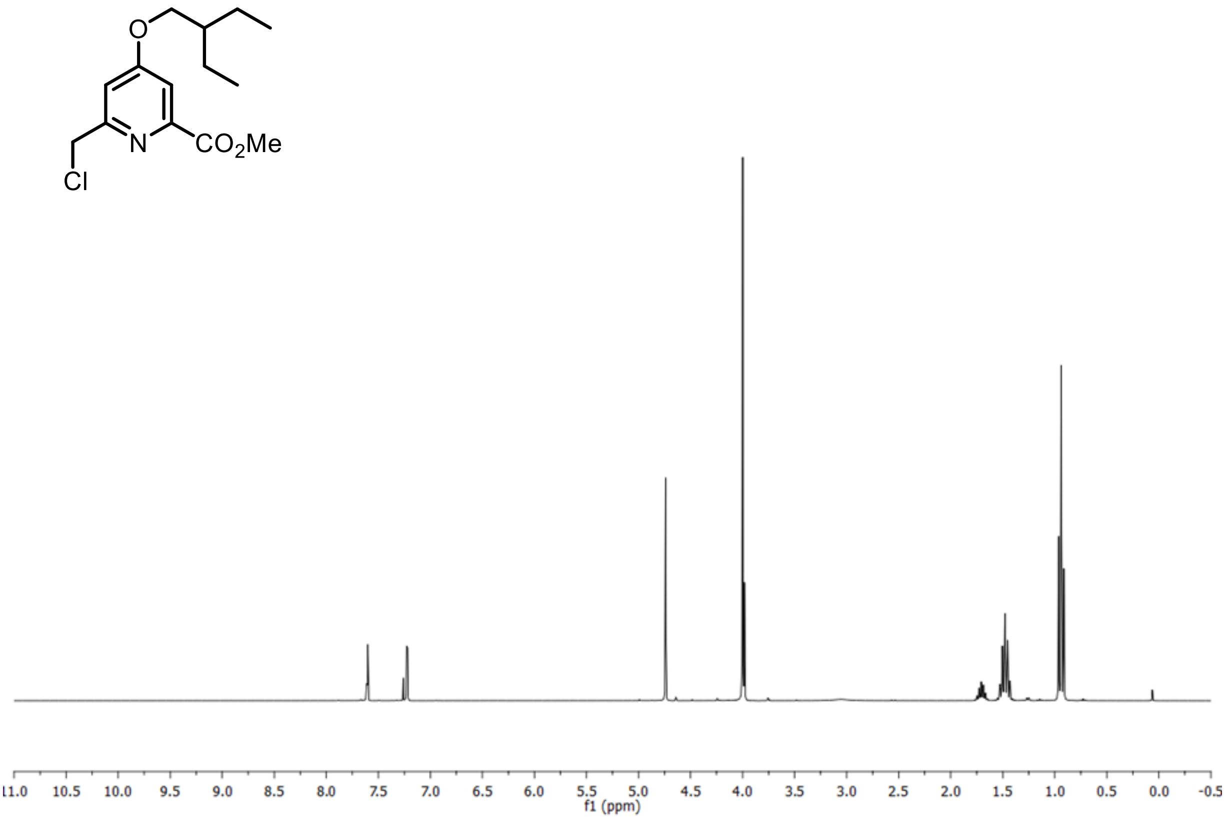

Figure S43 ^{1}H NMR spectrum (300 MHz, $CDCl_3$) of **20**.

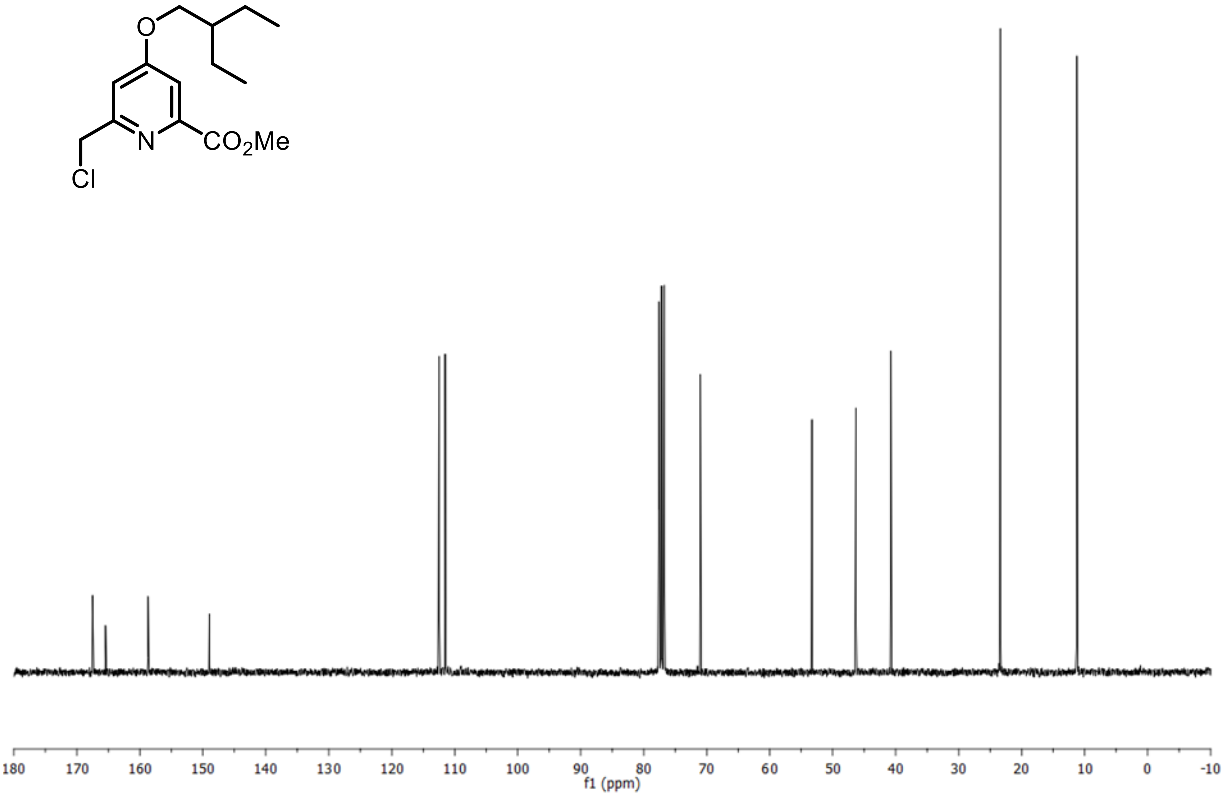

Figure S44 ^{13}C NMR spectrum (75 MHz, $CDCl_3$) of **20**.

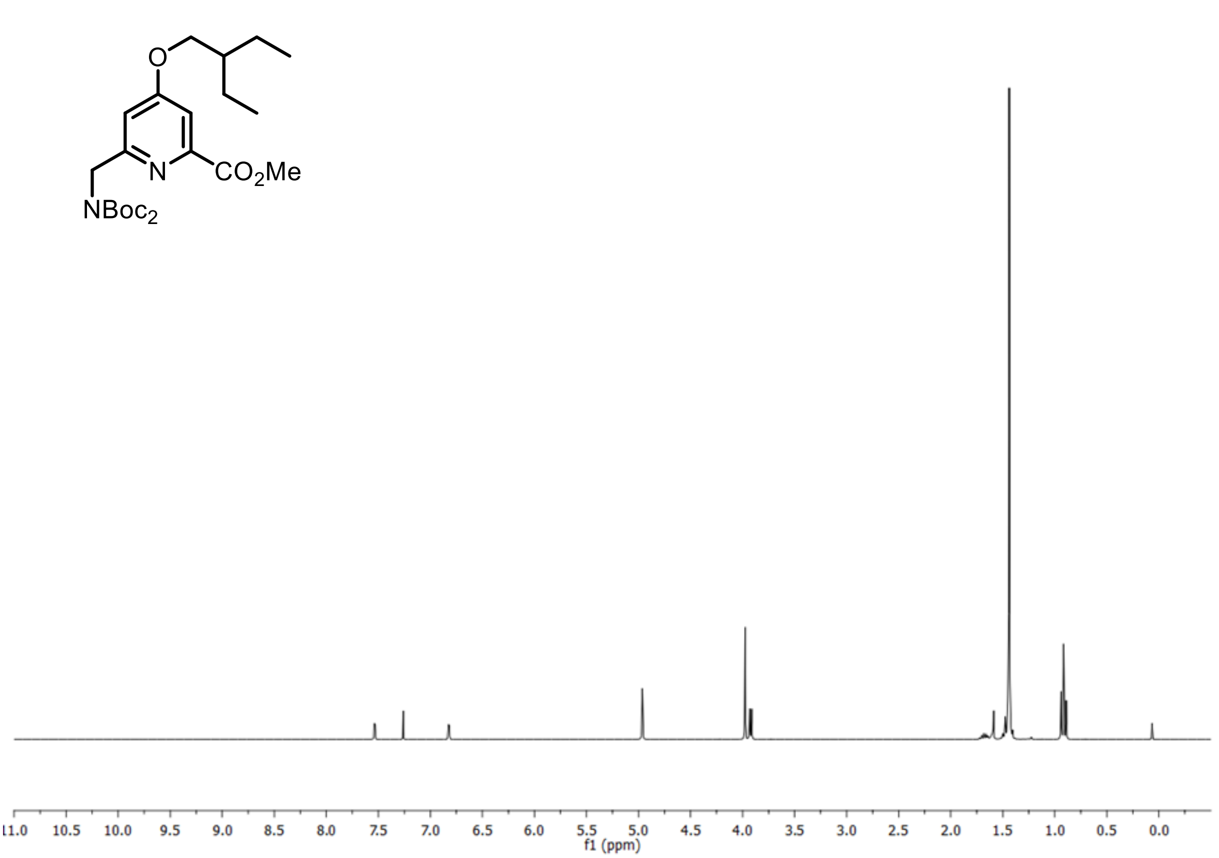

Figure S45 ^{1}H NMR spectrum (300 MHz, $CDCl_3$) of **21**.

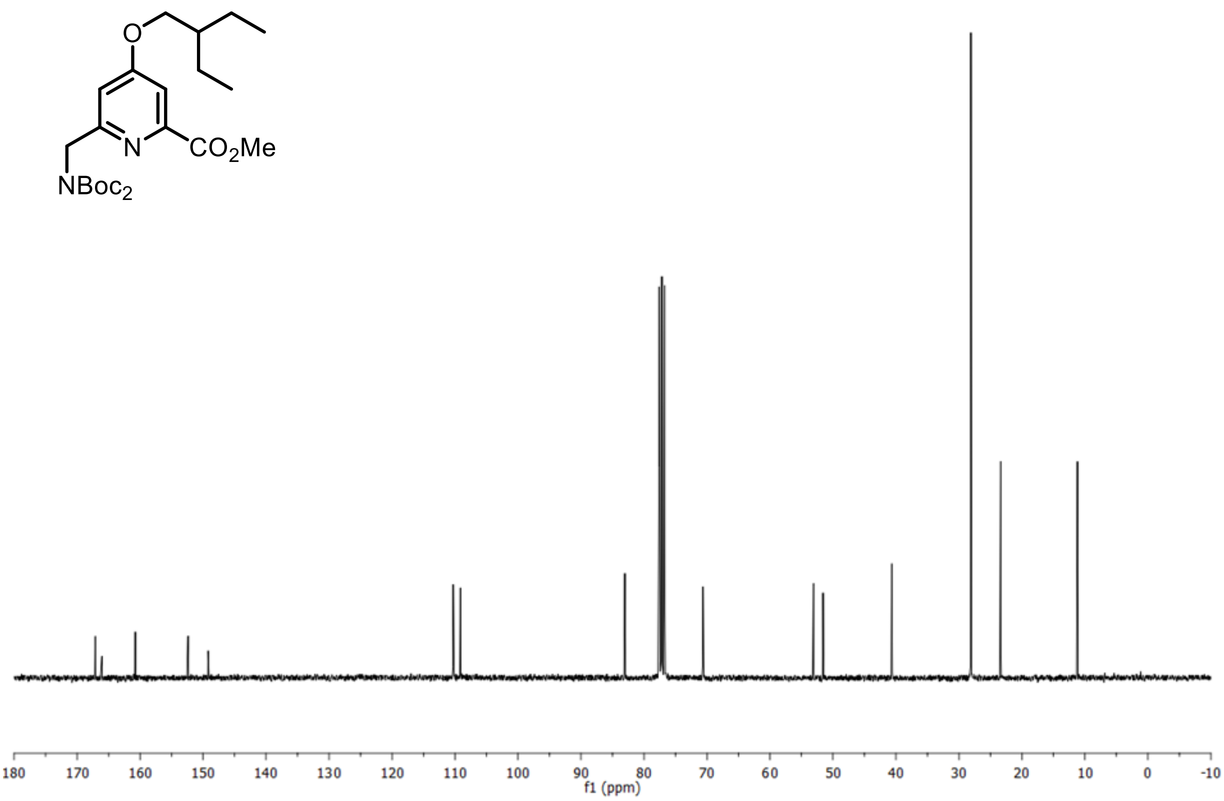

Figure S46 ^{13}C NMR spectrum (75 MHz, $CDCl_3$) of **21**.

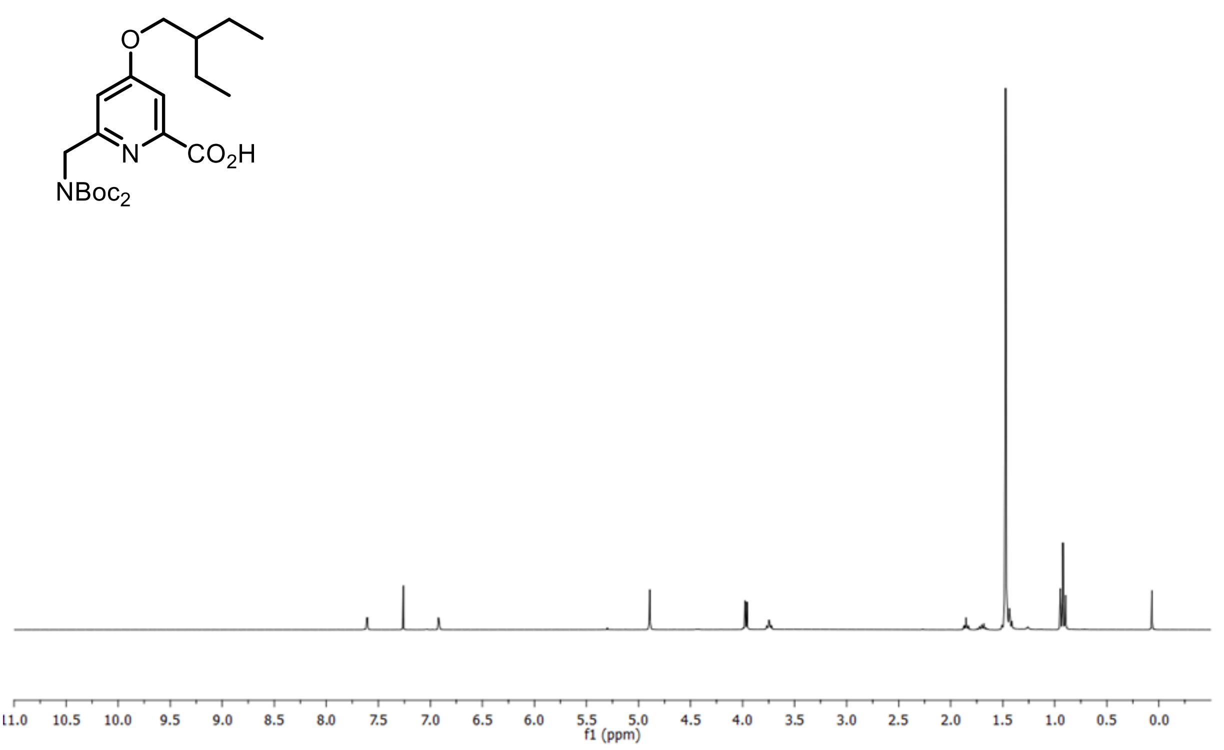

Figure S47 ^{1}H NMR spectrum (300 MHz, $CDCl_3$) of **22**.

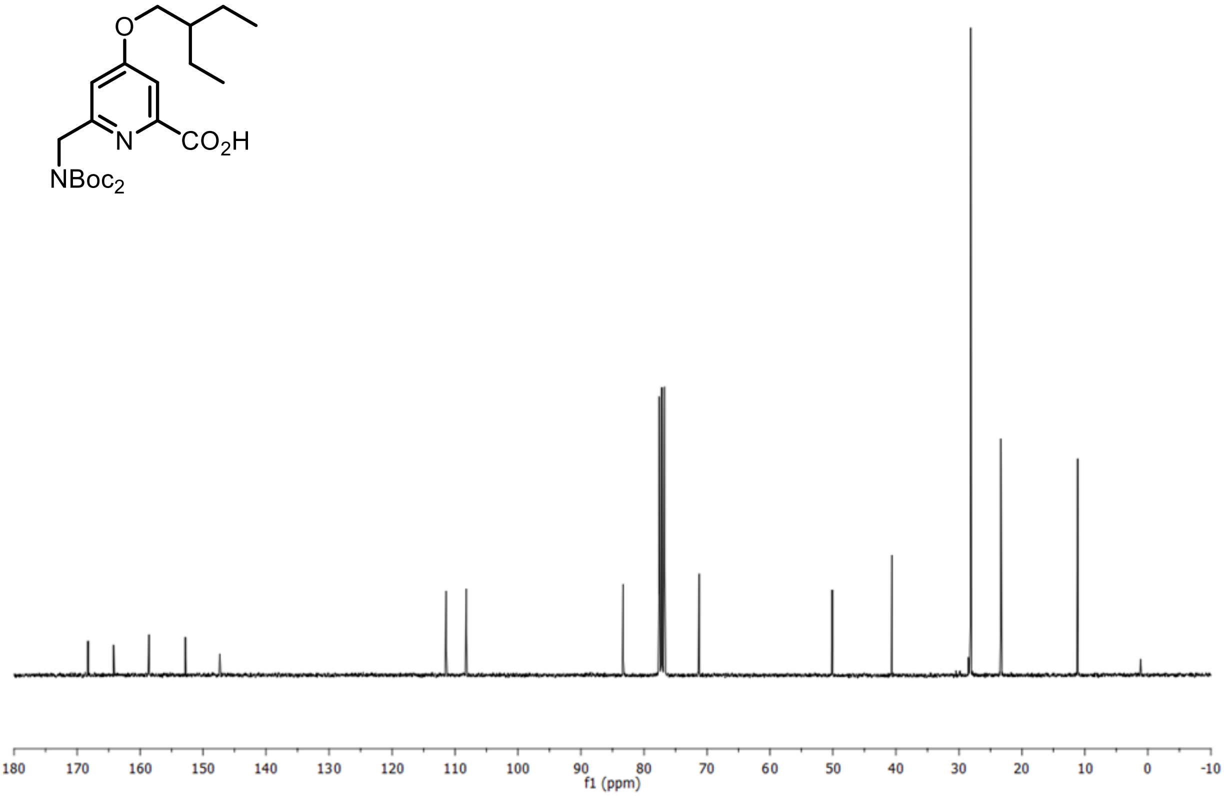

Figure S48 ^{13}C NMR spectrum (75 MHz, $CDCl_3$) of **22**.

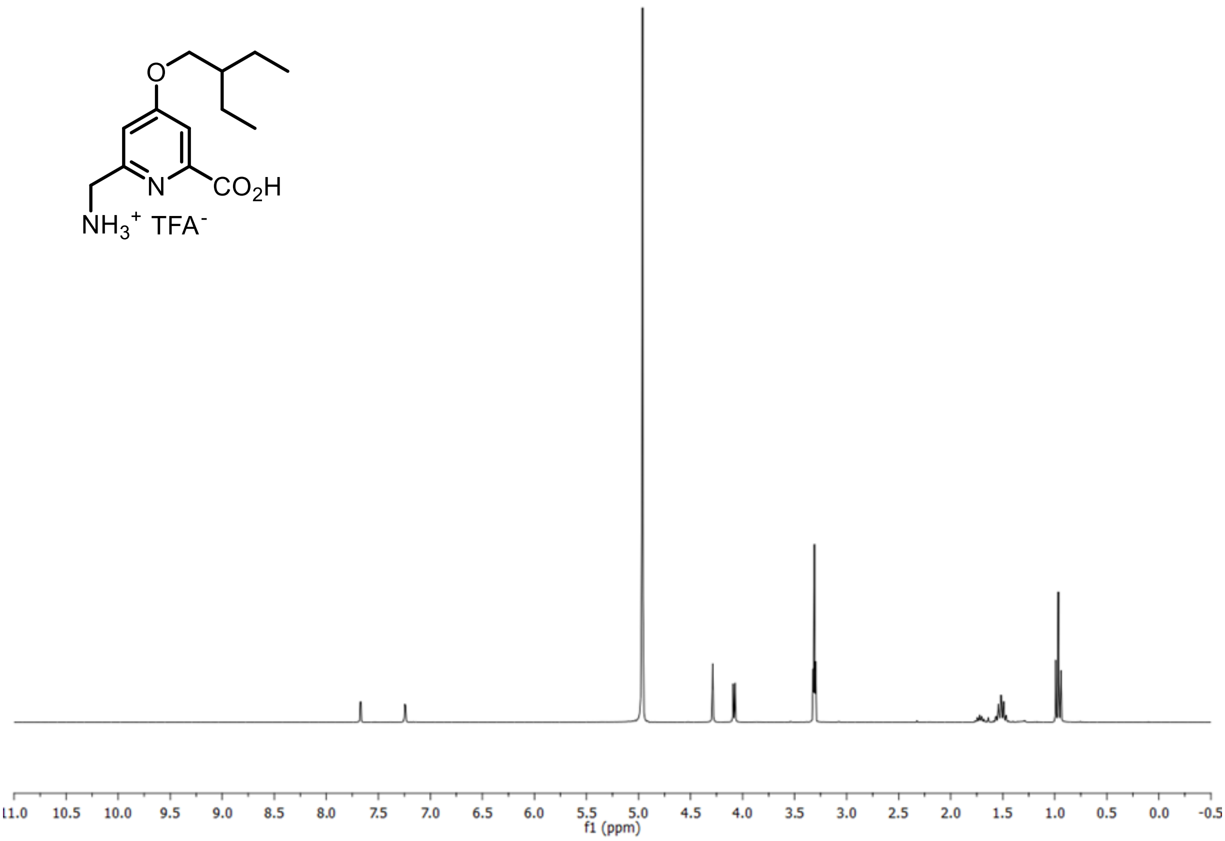

Figure S49 ^{1}H NMR spectrum (300 MHz, MeOD-d_4) of **23**.

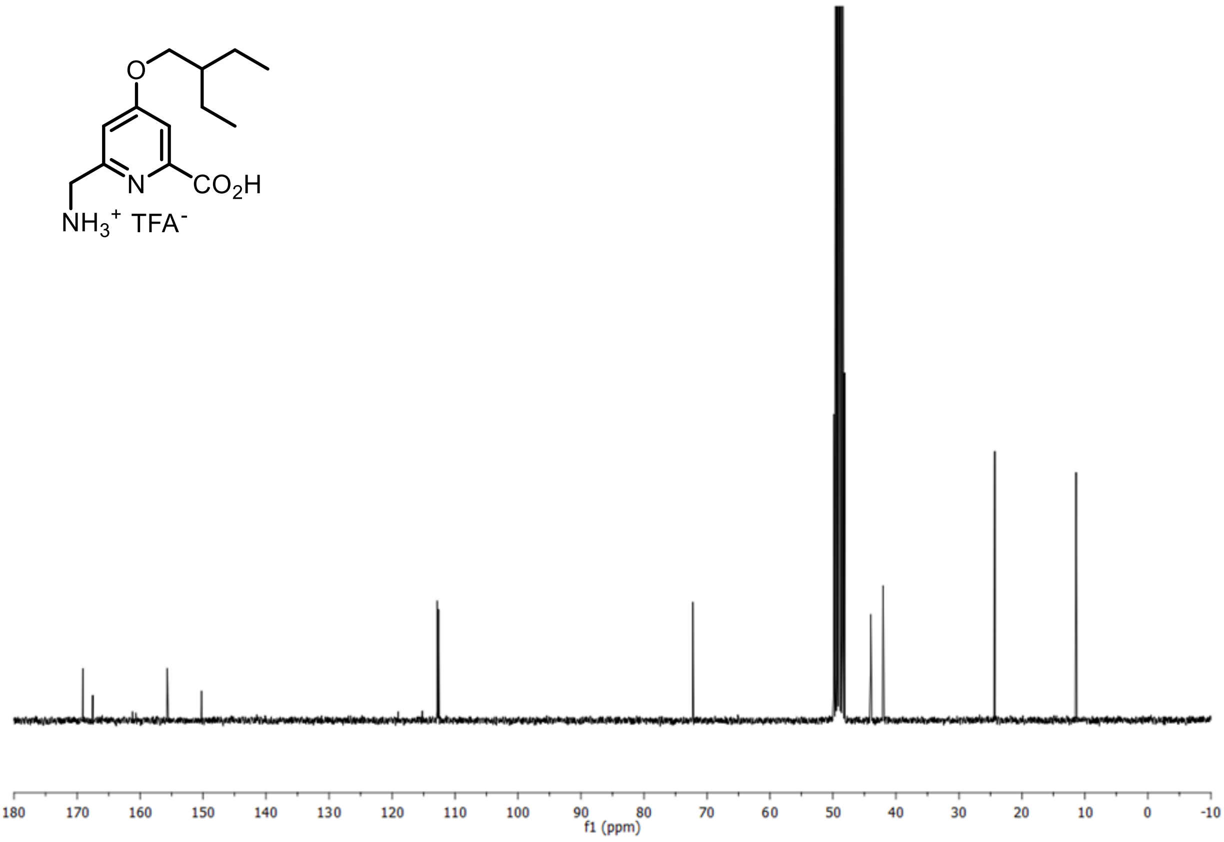

Figure S50 ^{13}C NMR spectrum (75 MHz, MeOD-d_4) of **23**.

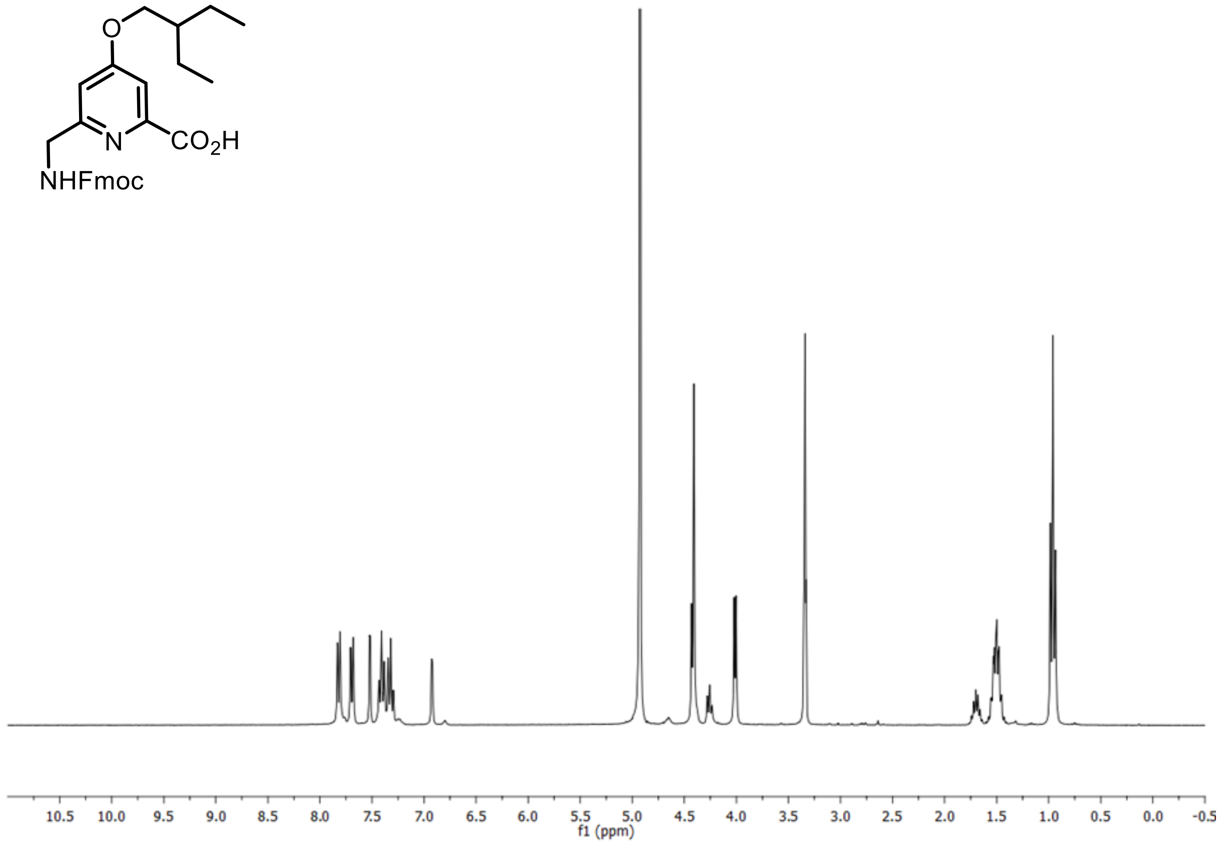

Figure S51 ^{1}H NMR spectrum (300 MHz, MeOD-d_4) of **24**.

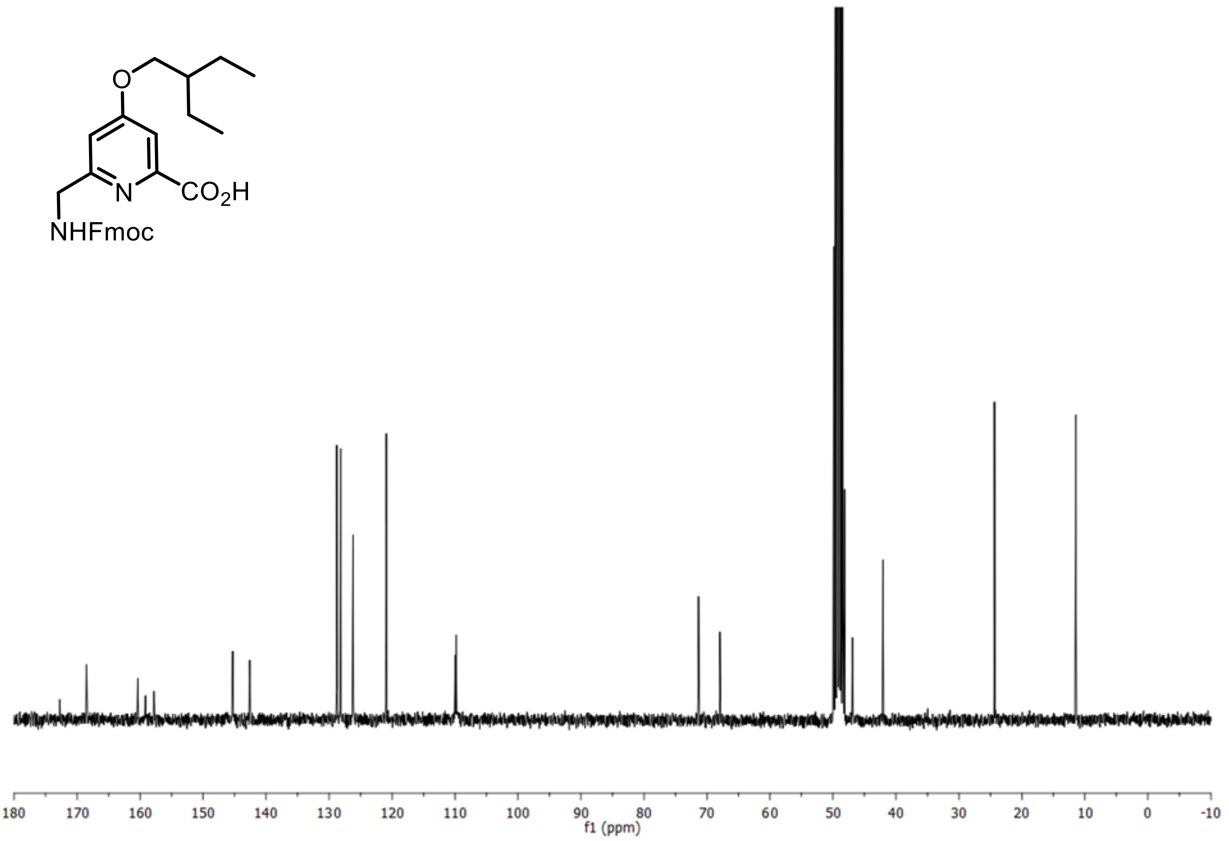

Figure S52 ^{13}C NMR spectrum (75 MHz, MeOD-d_4) of **24**.

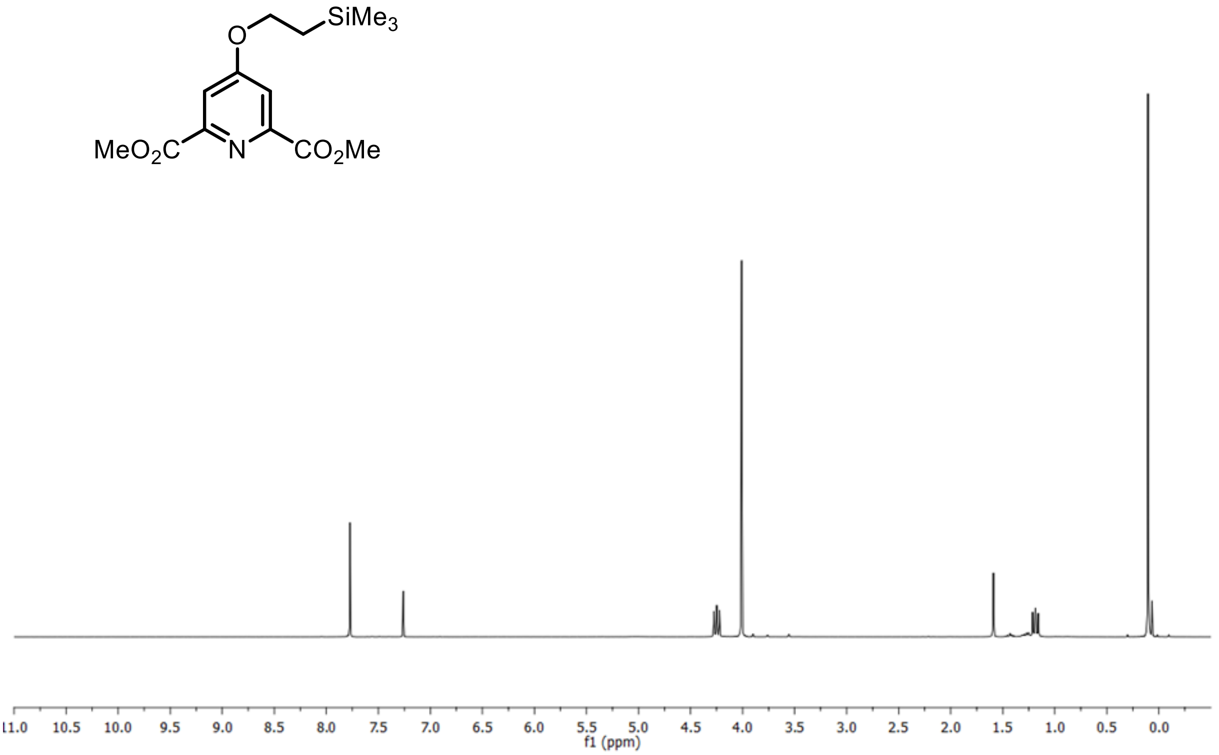

Figure S53 ^{1}H NMR spectrum (300 MHz, $CDCl_3$) of **25**.

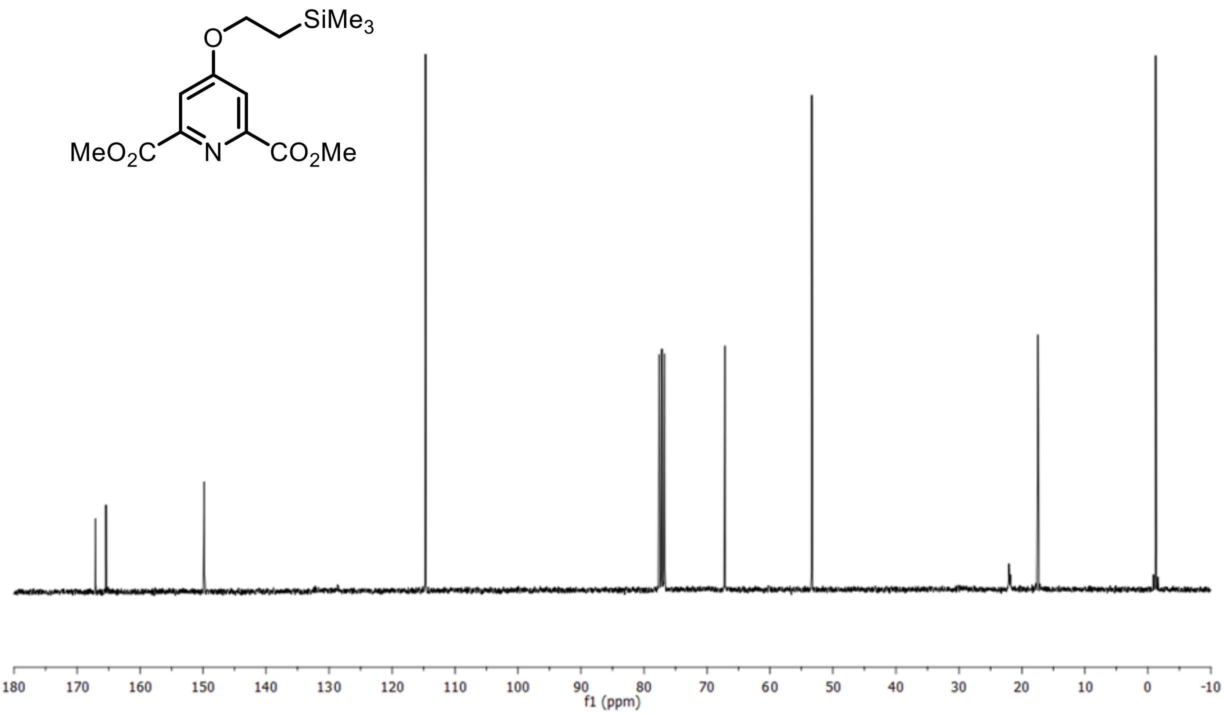

Figure S54 ^{13}C NMR spectrum (75 MHz, $CDCl_3$) of **25**.

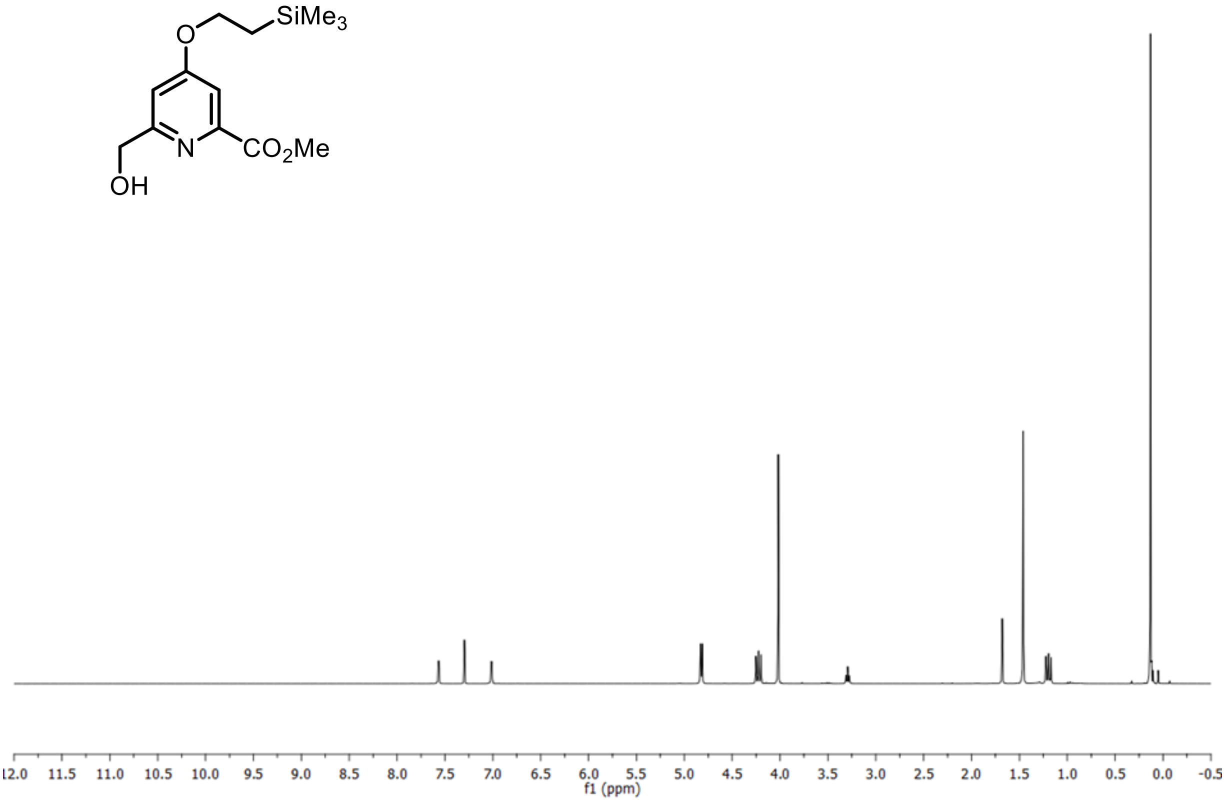

Figure S55 ^{1}H NMR spectrum (300 MHz, $CDCl_3$) of **26**.

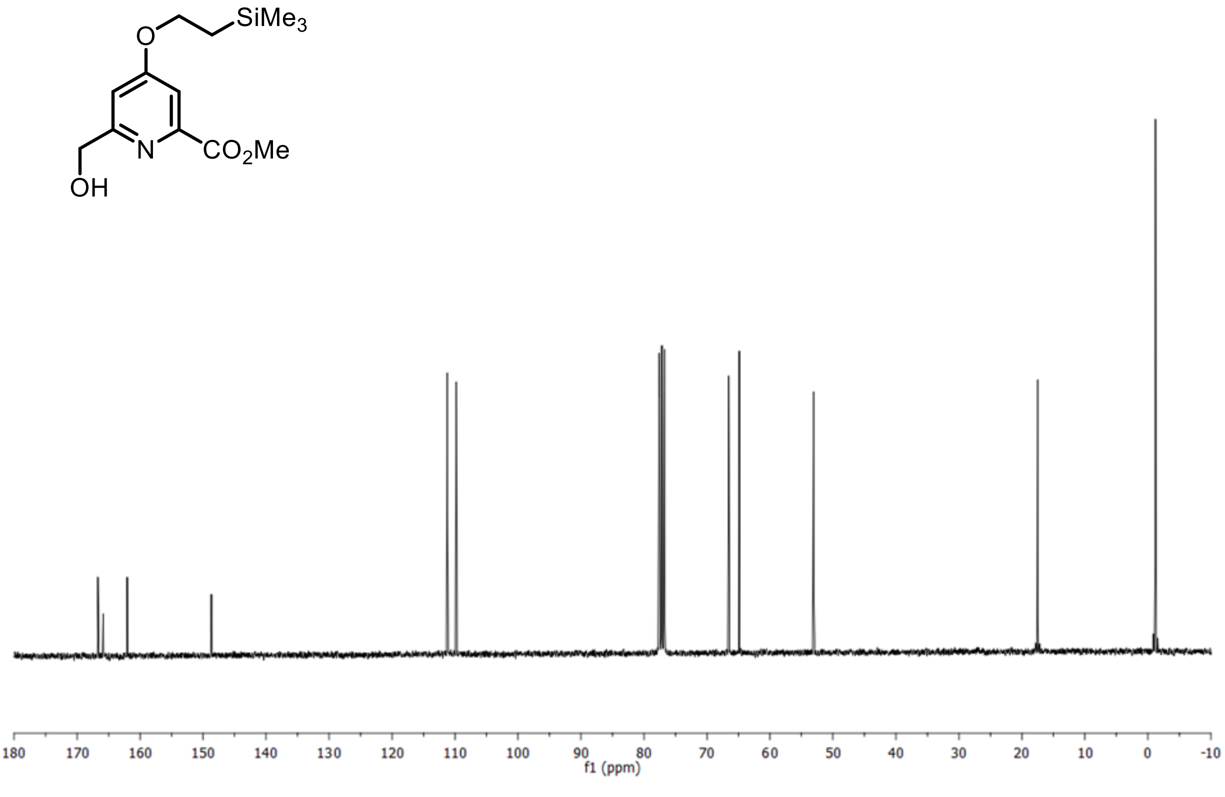

Figure S56 ^{13}C NMR spectrum (75 MHz, $CDCl_3$) of **26**.

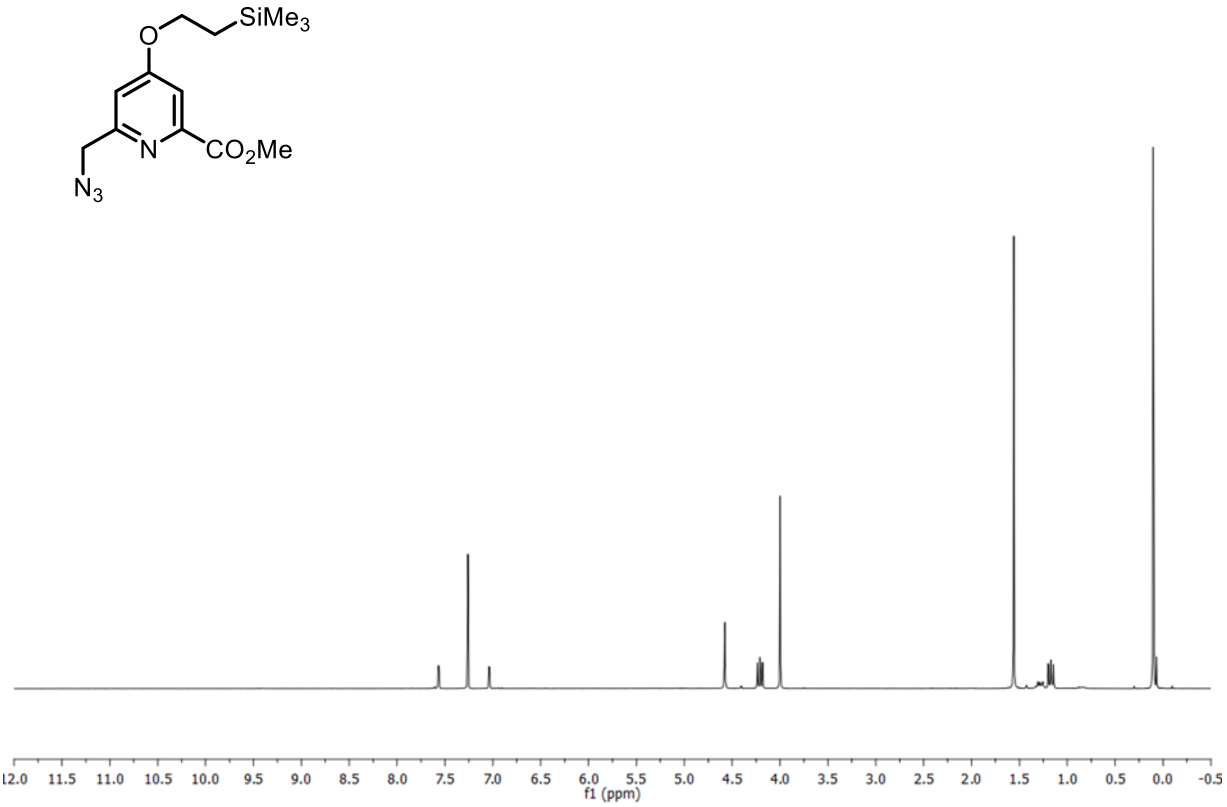

Figure S57 ^{1}H NMR spectrum (300 MHz, $CDCl_3$) of **27**.

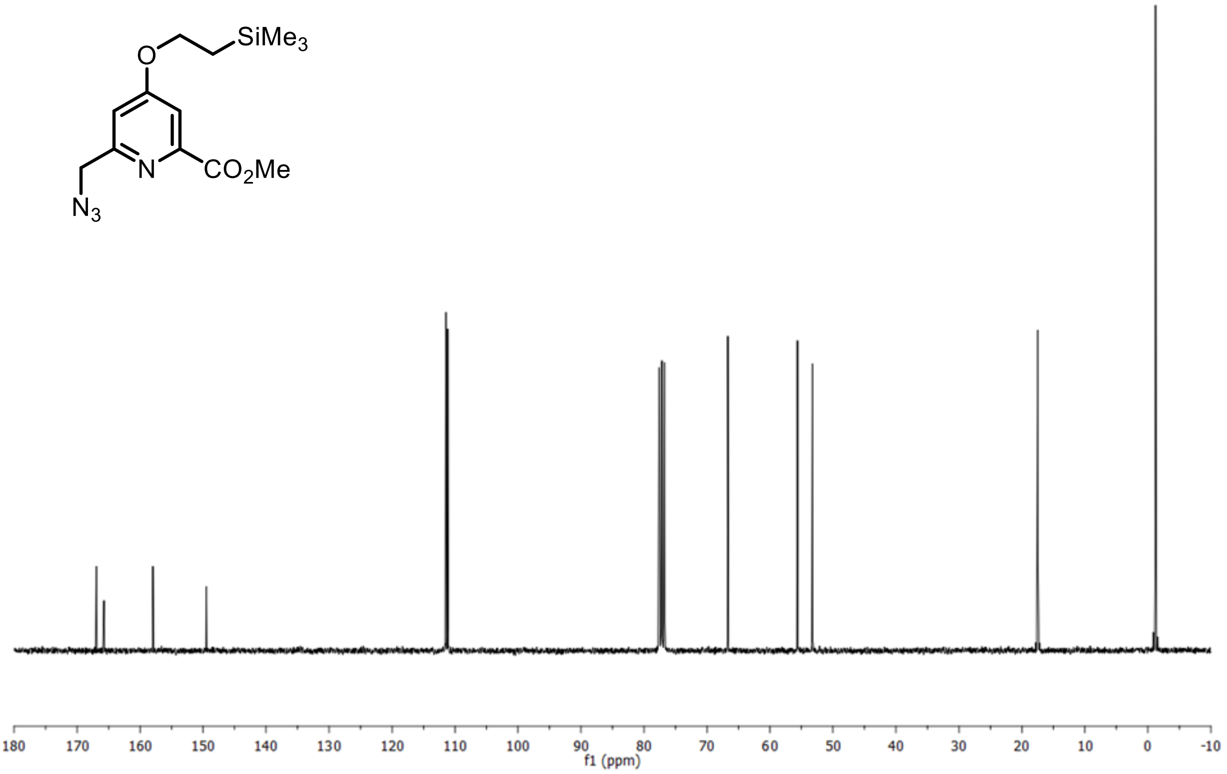

Figure S58 ^{13}C NMR spectrum (75 MHz, $CDCl_3$) of **27**.

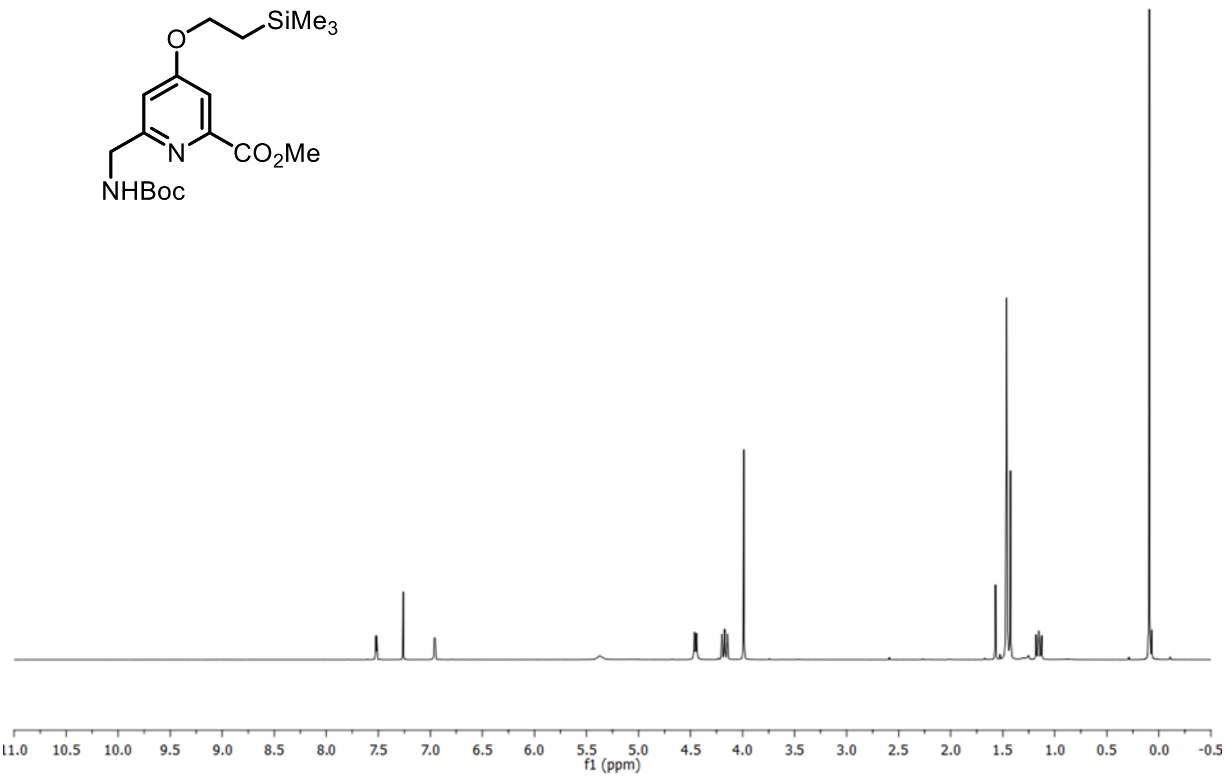

Figure S59 ^{1}H NMR spectrum (300 MHz, $CDCl_3$) of **28**.

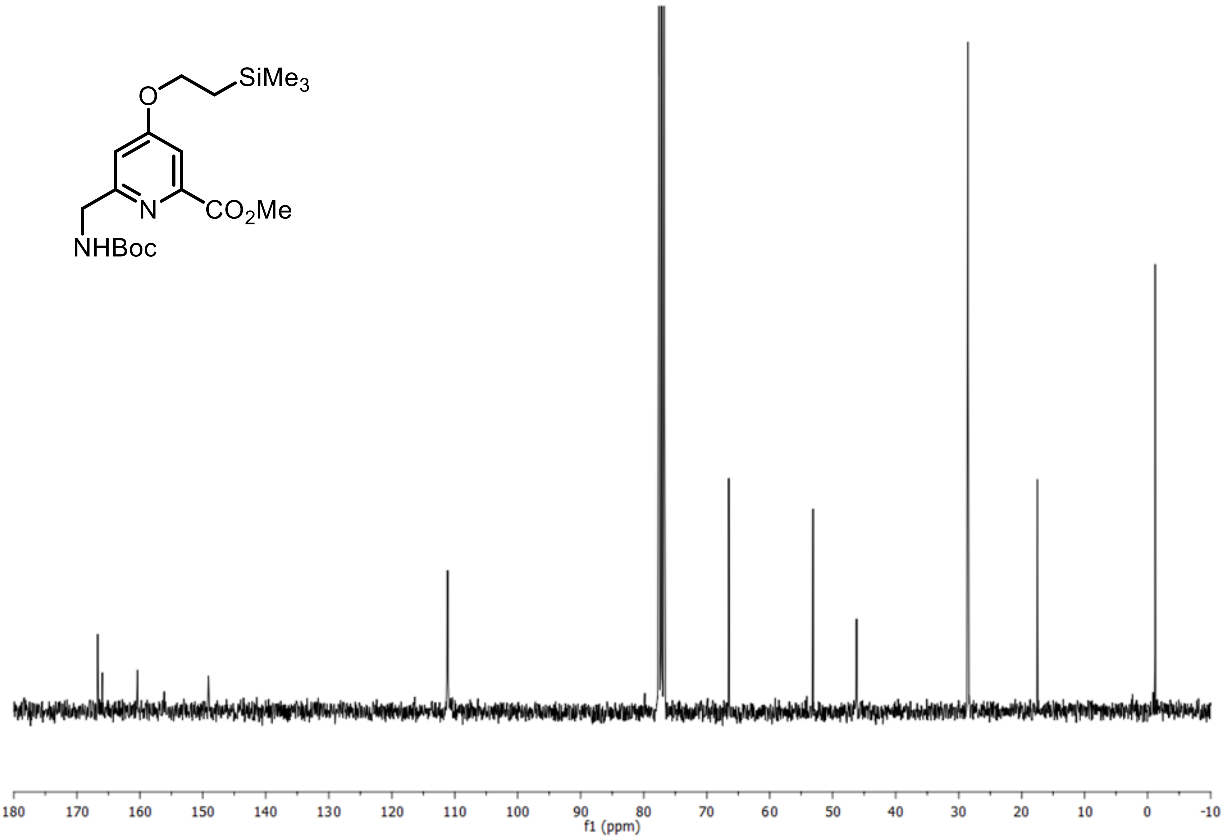

Figure S60 ^{13}C NMR spectrum (75 MHz, $CDCl_3$) of **28**.

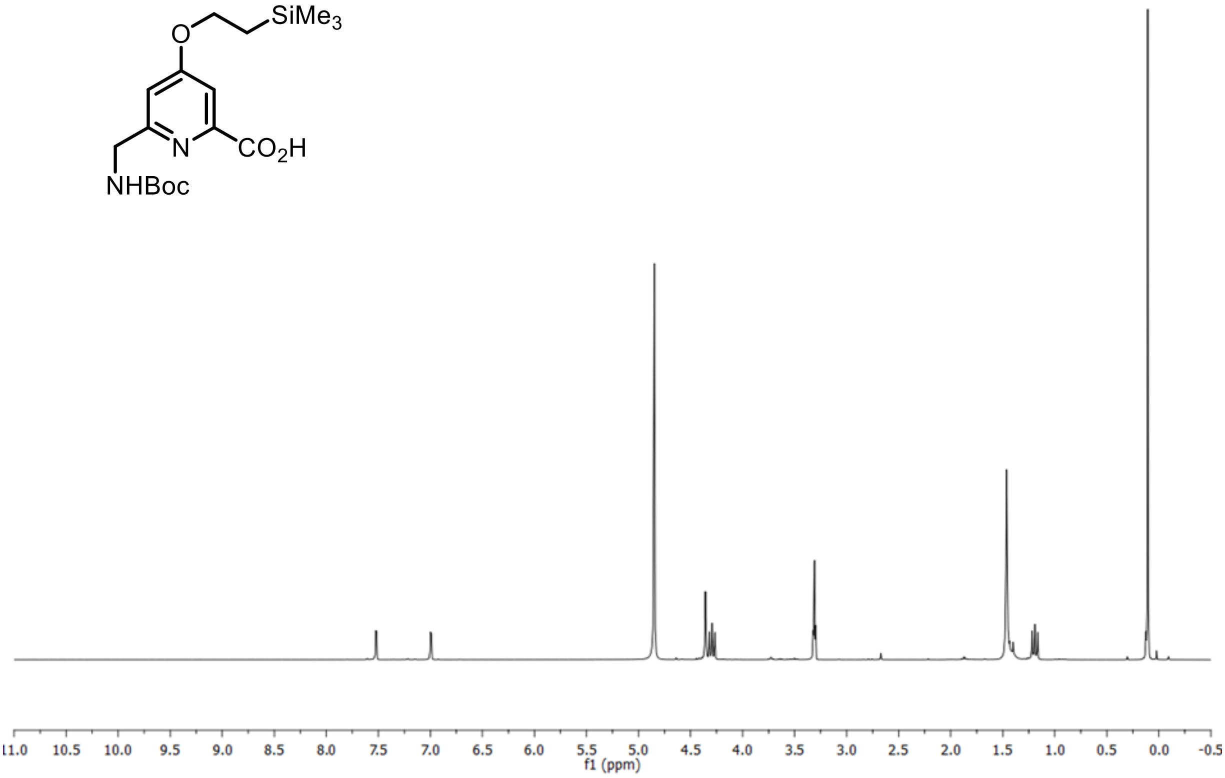

Figure S61 ^{1}H NMR spectrum (300 MHz, MeOD-d_4) of **29**.

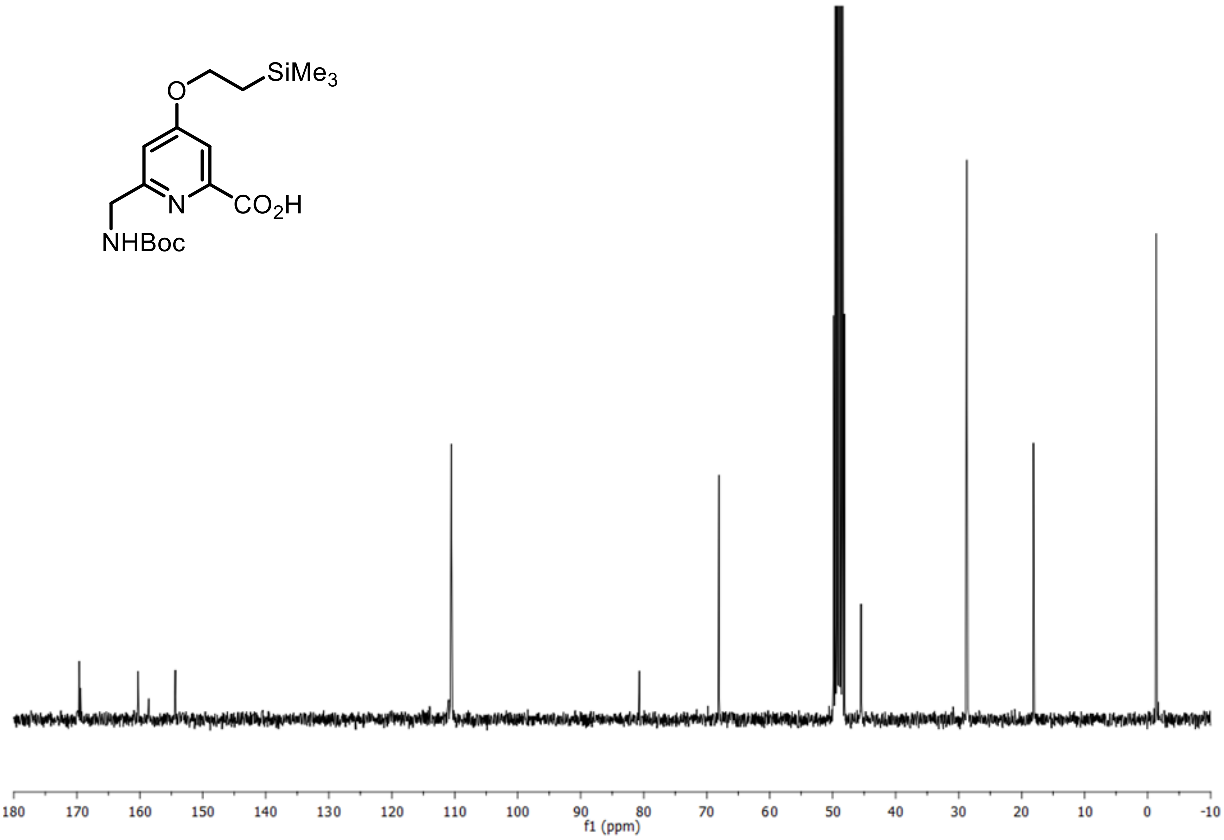

Figure S62 ^{13}C NMR spectrum (75 MHz, MeOD-d_4) of **29**.

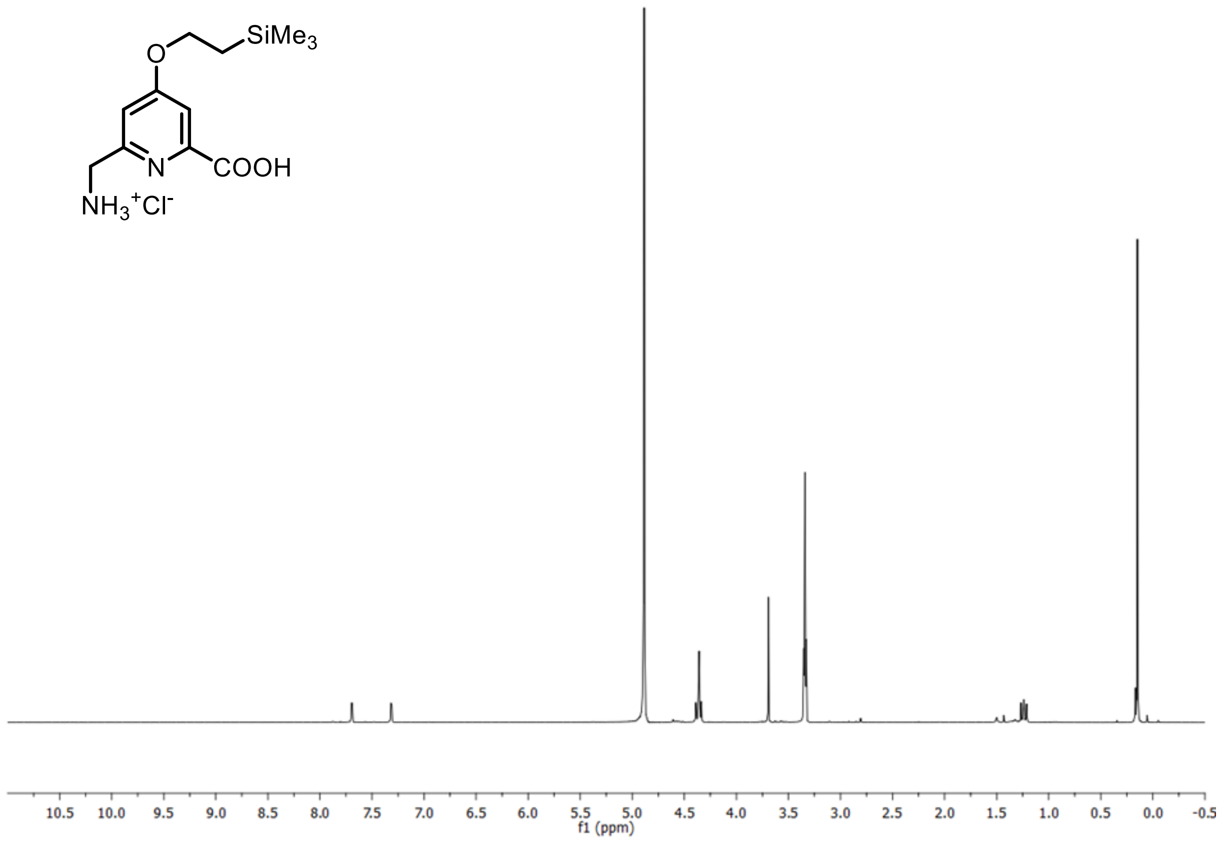

Figure S63 ^{1}H NMR spectrum (300 MHz, MeOD-d_4) of **30**.

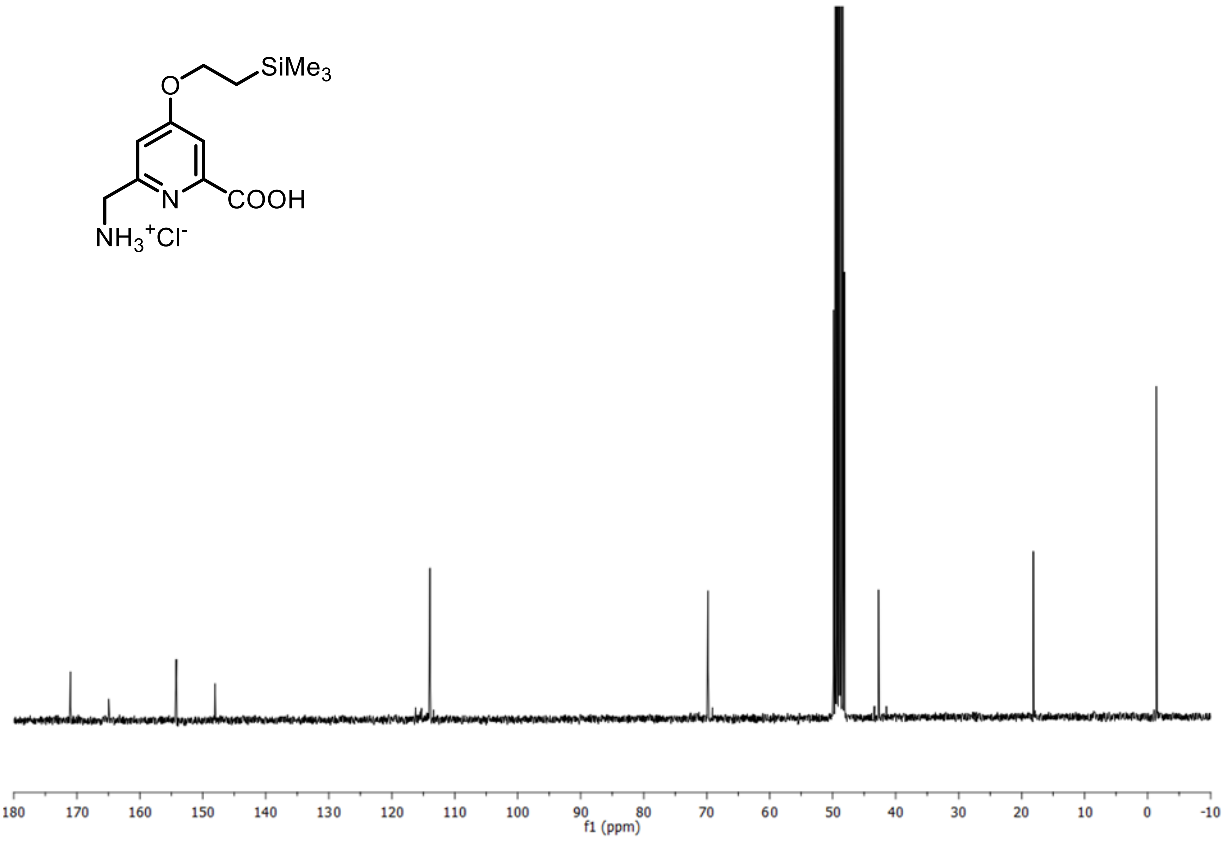

Figure 64 ^{13}C NMR spectrum (75 MHz, MeOD-d_4) of **30**.

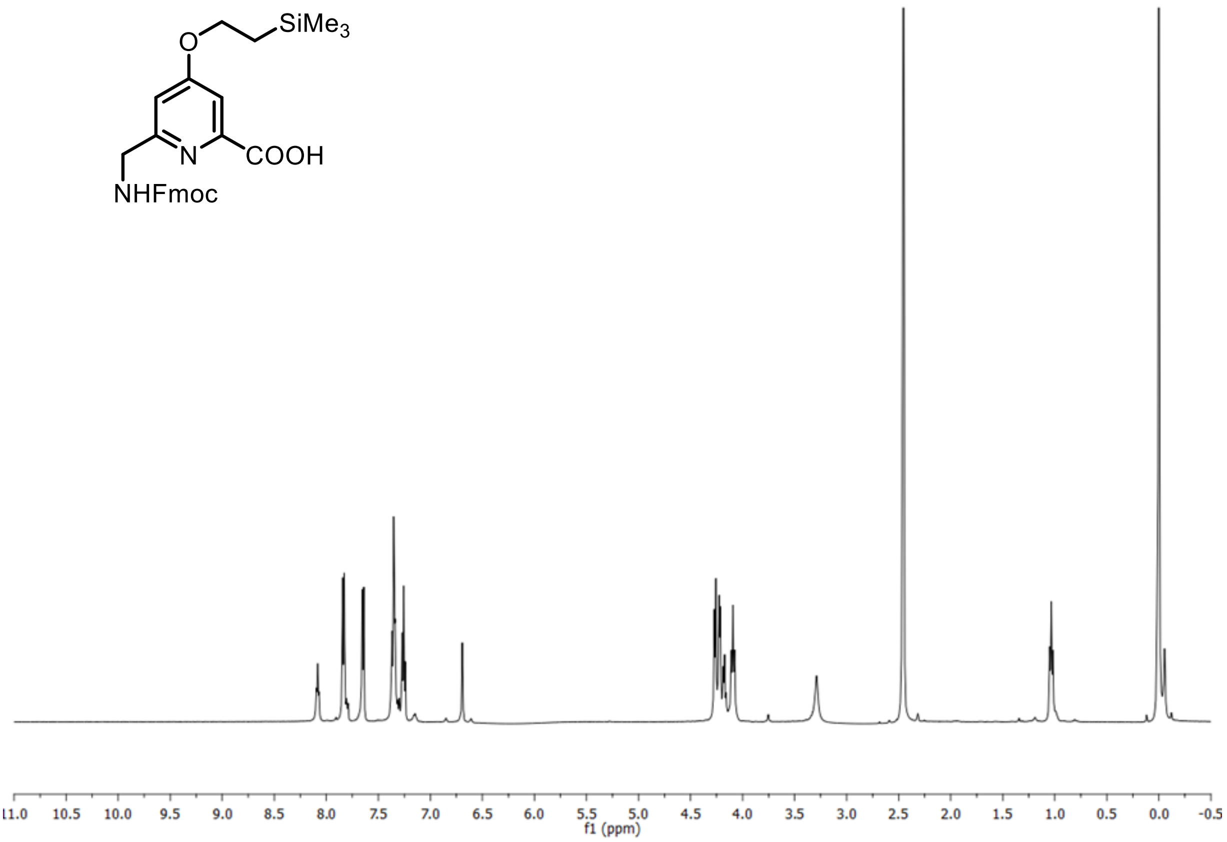

Figure S65 ^{1}H NMR spectrum (500 MHz, DMSO-d_6) of **31**.

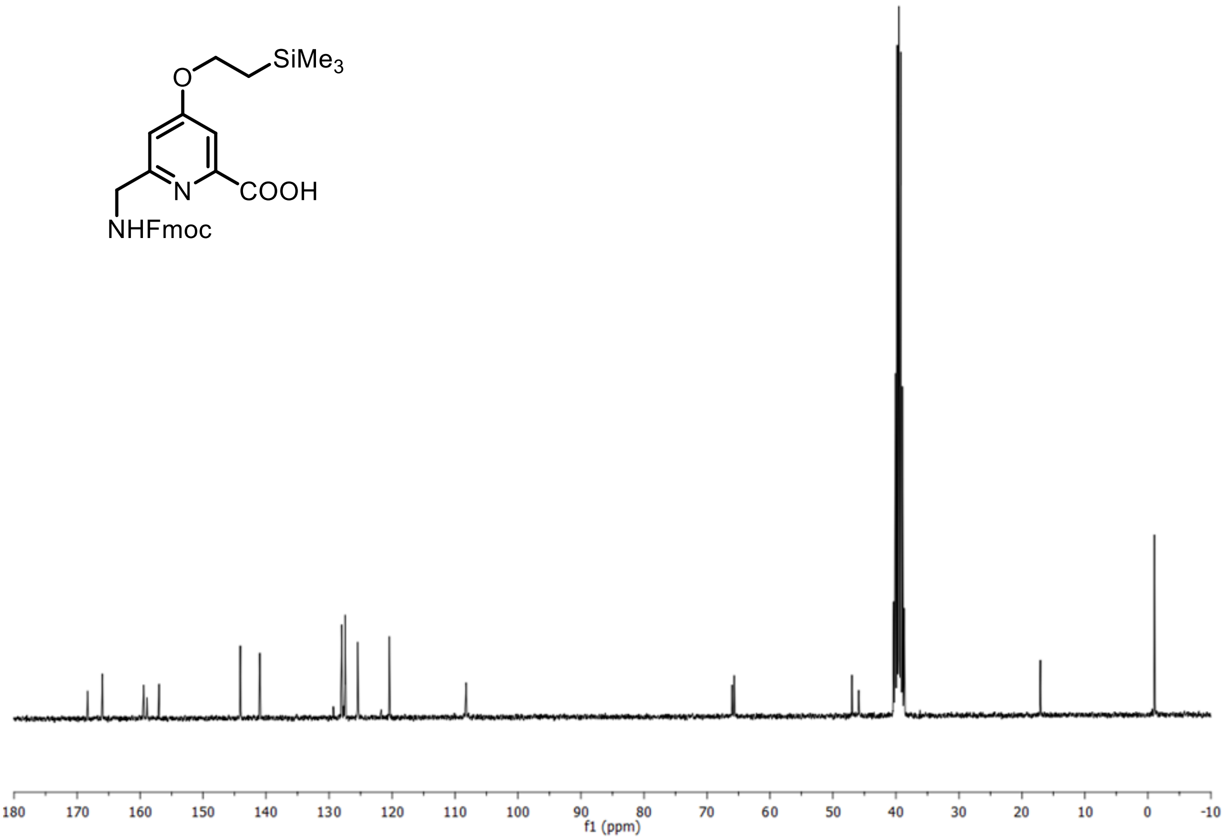

Figure S66 ^{13}C NMR spectrum (75 MHz, DMSO-d_4) of **31**.

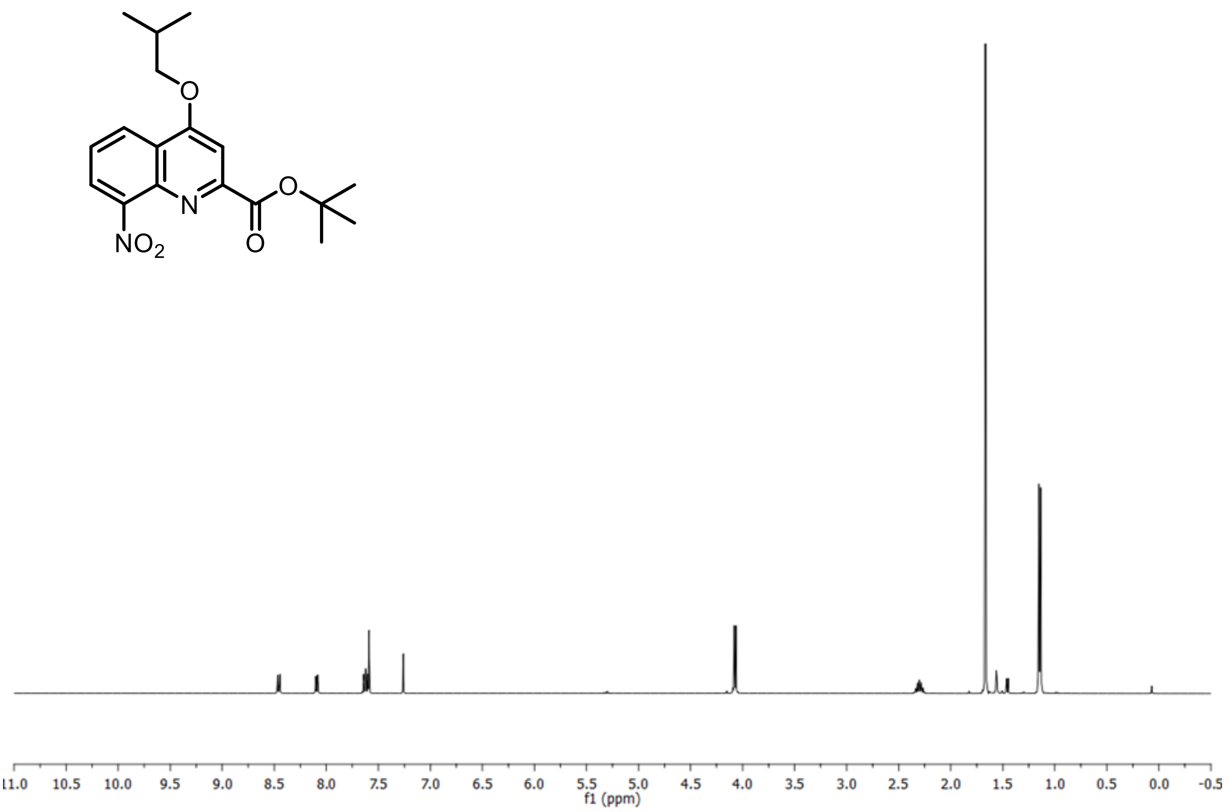

Figure S67 ^{1}H NMR spectrum (300 MHz, $CDCl_3$) of **33**.

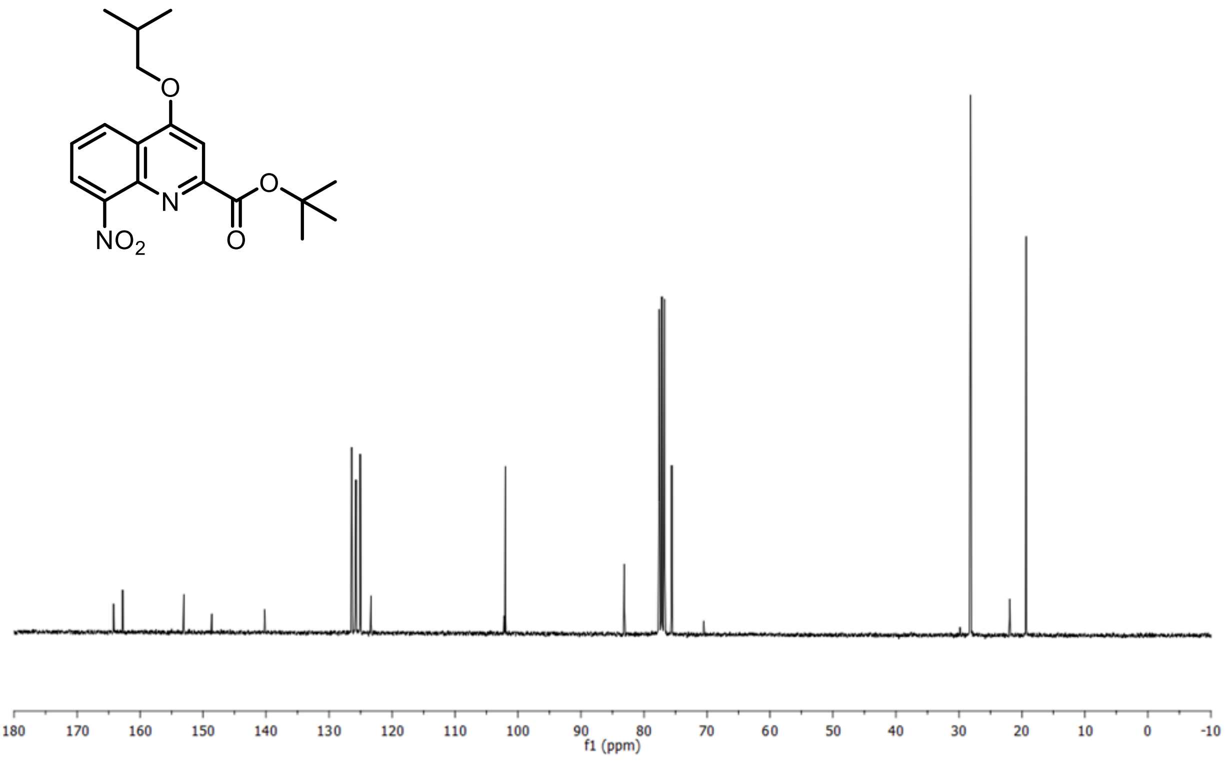

Figure S68 ^{13}C NMR spectrum (75 MHz, $CDCl_3$) of **33**.

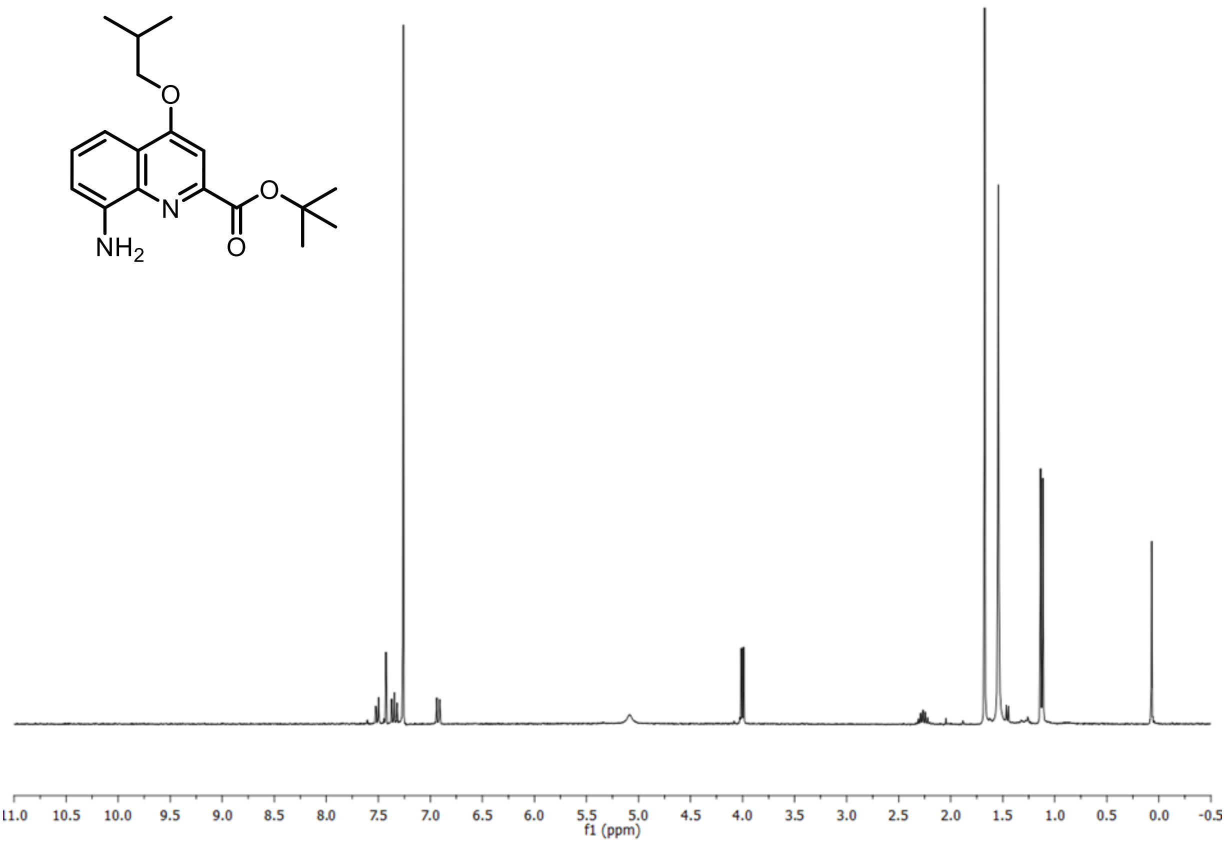

Figure S69 ^{1}H NMR spectrum (300 MHz, $CDCl_3$) of **34**.

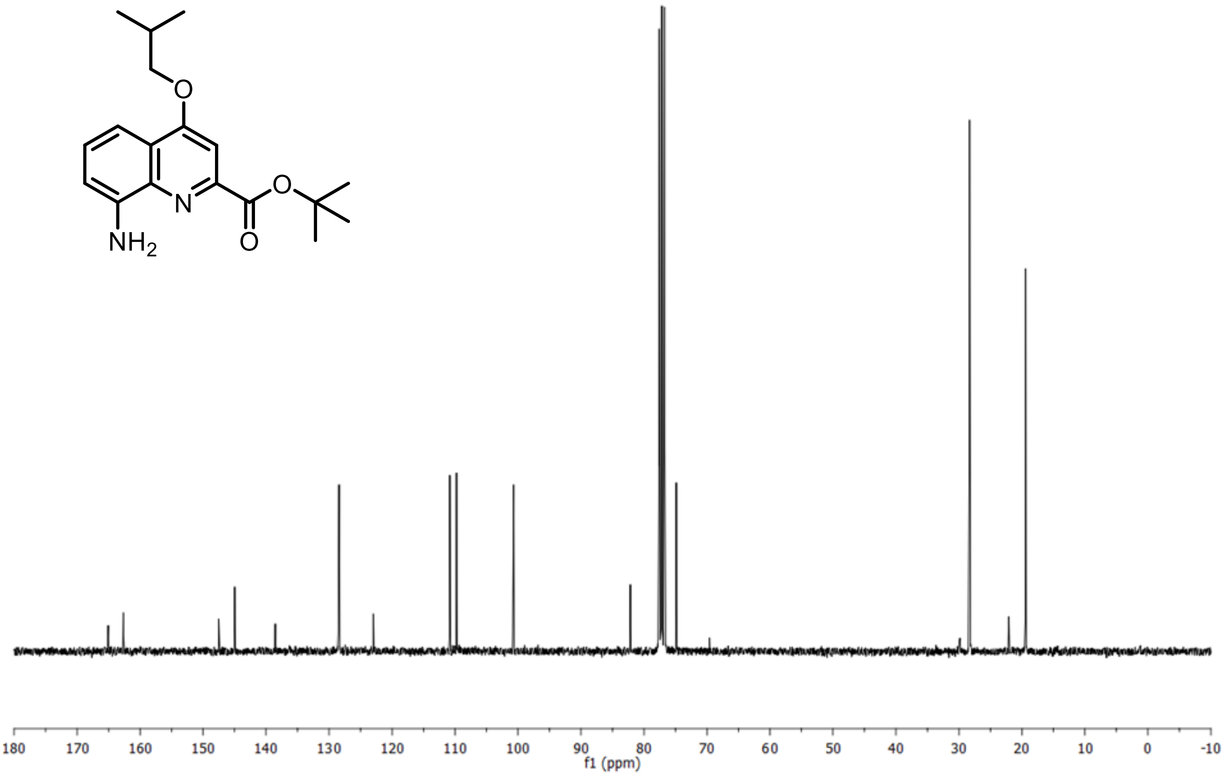

Figure S70 ^{13}C NMR spectrum (75 MHz, $CDCl_3$) of **34**.

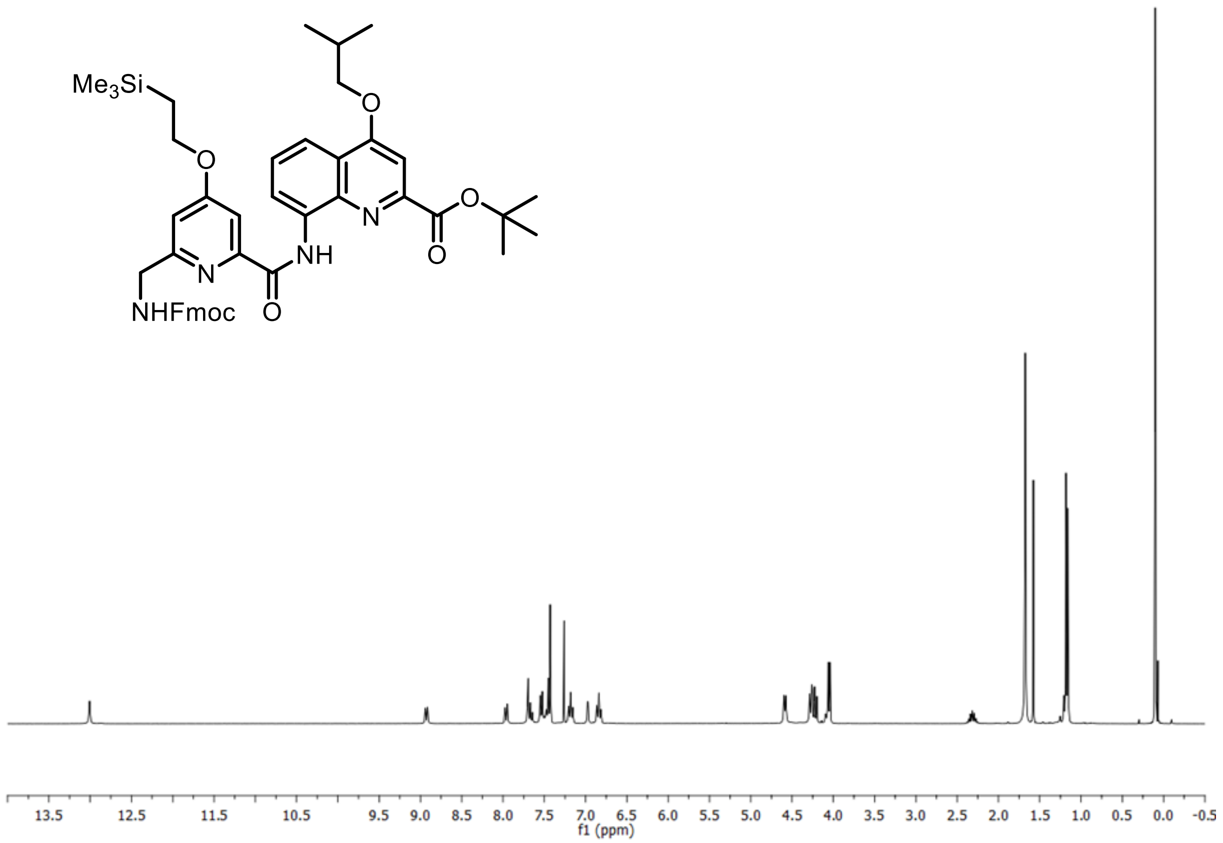

Figure S71 ^{1}H NMR spectrum (300 MHz, $CDCl_3$) of **35**.

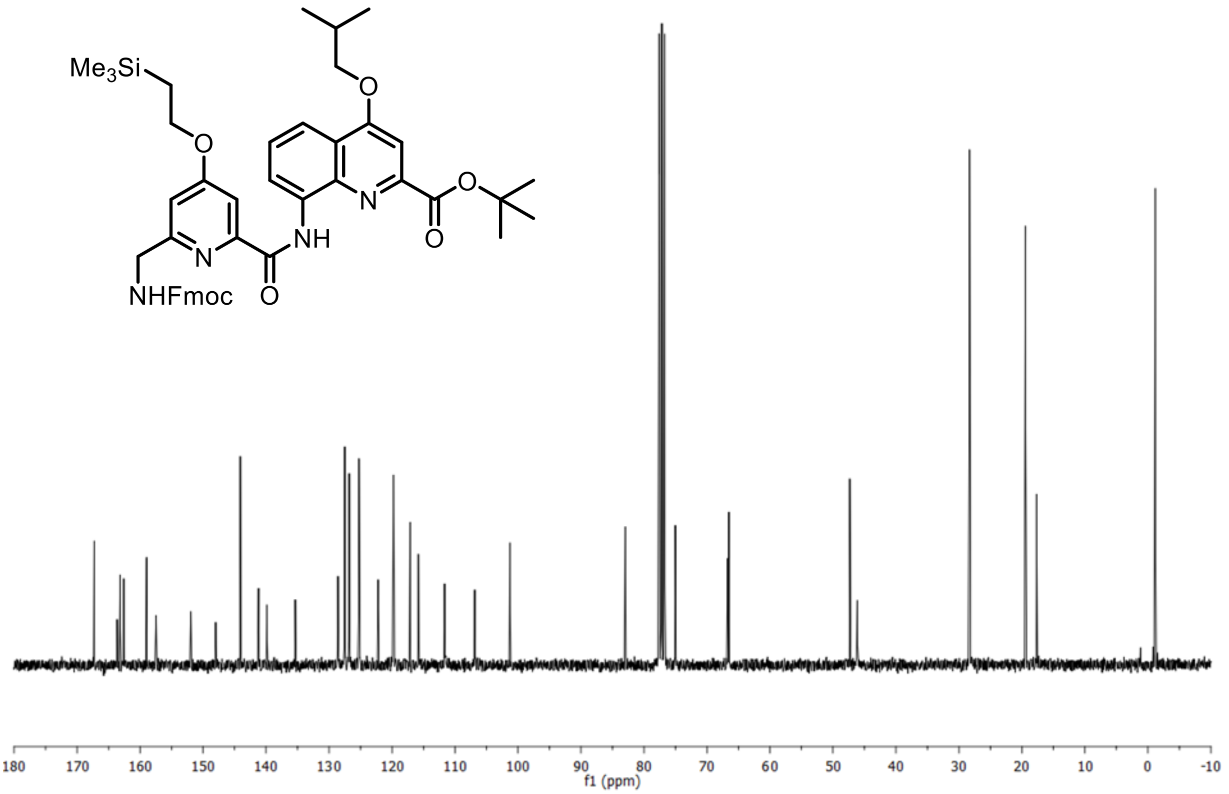

Figure S72 ^{13}C NMR spectrum (75 MHz, $CDCl_3$) of **35**.

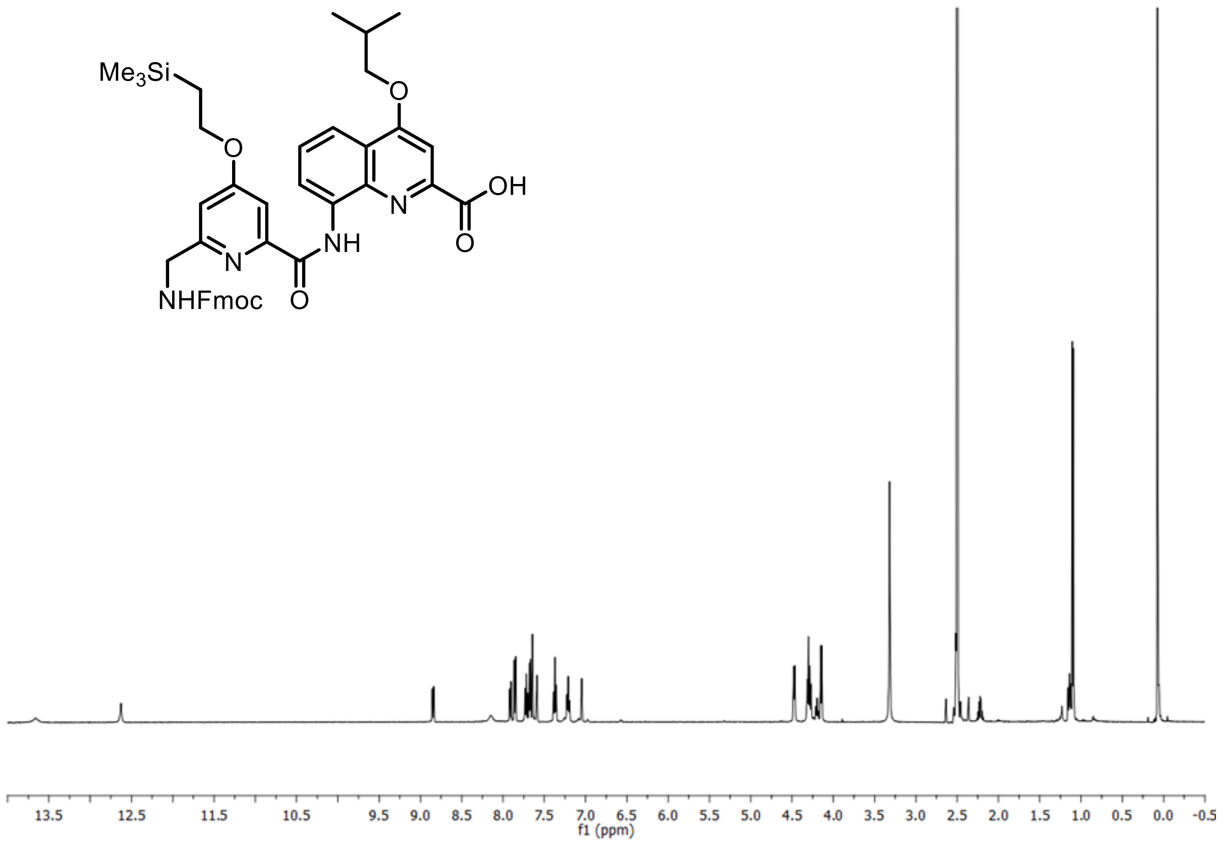

Figure S73 ^{1}H NMR spectrum (500 MHz, DMSO-d_6) of **36**.

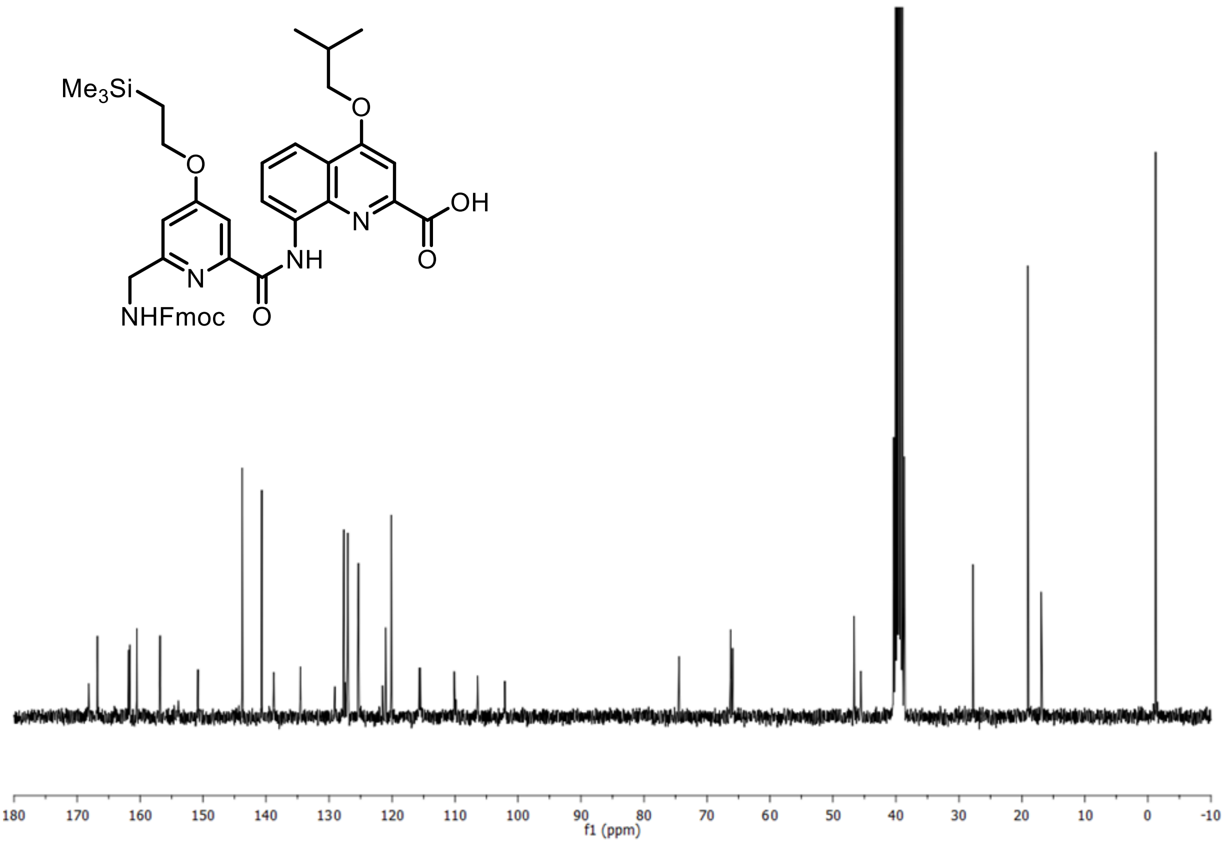

Figure S74 ^{13}C NMR spectrum (75 MHz, DMSO-d_6) of **36**.

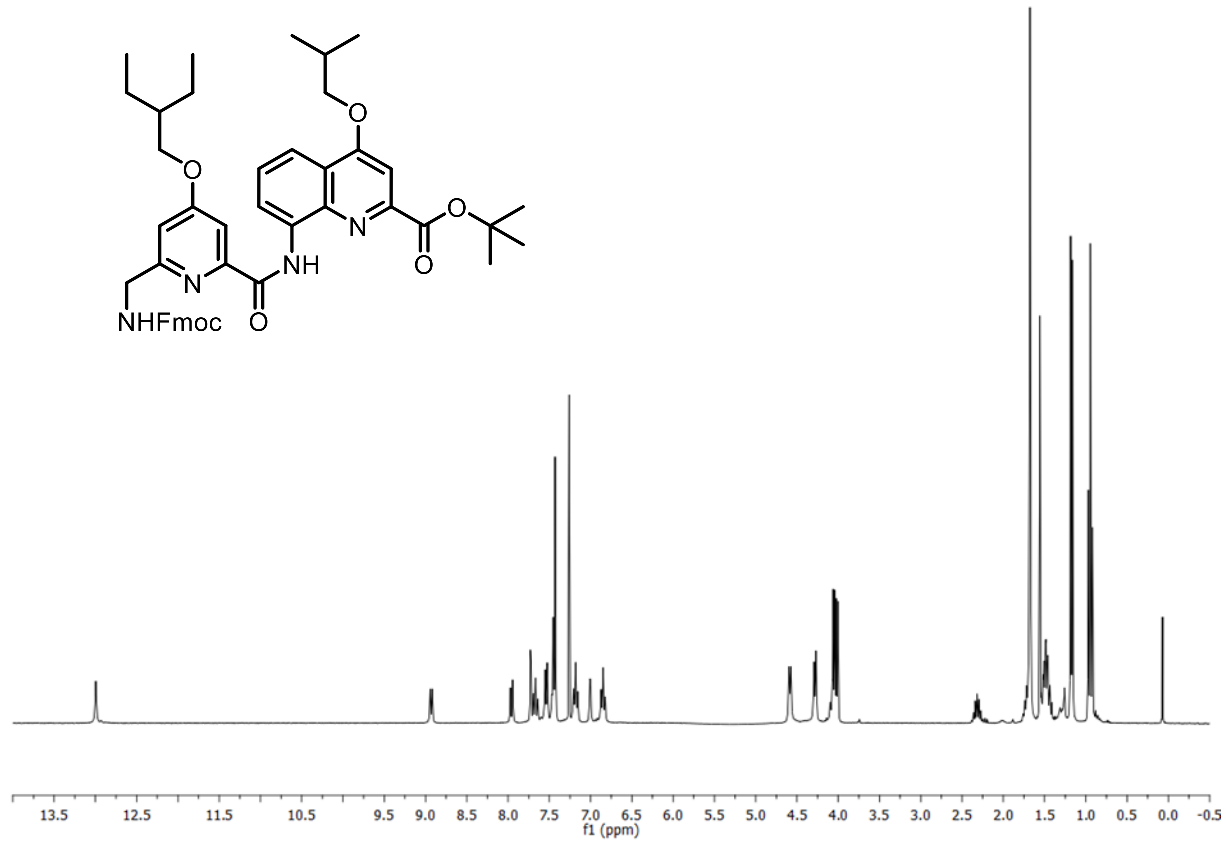

Figure S75 ^{1}H NMR spectrum (300 MHz, $CDCl_3$) of **37**.

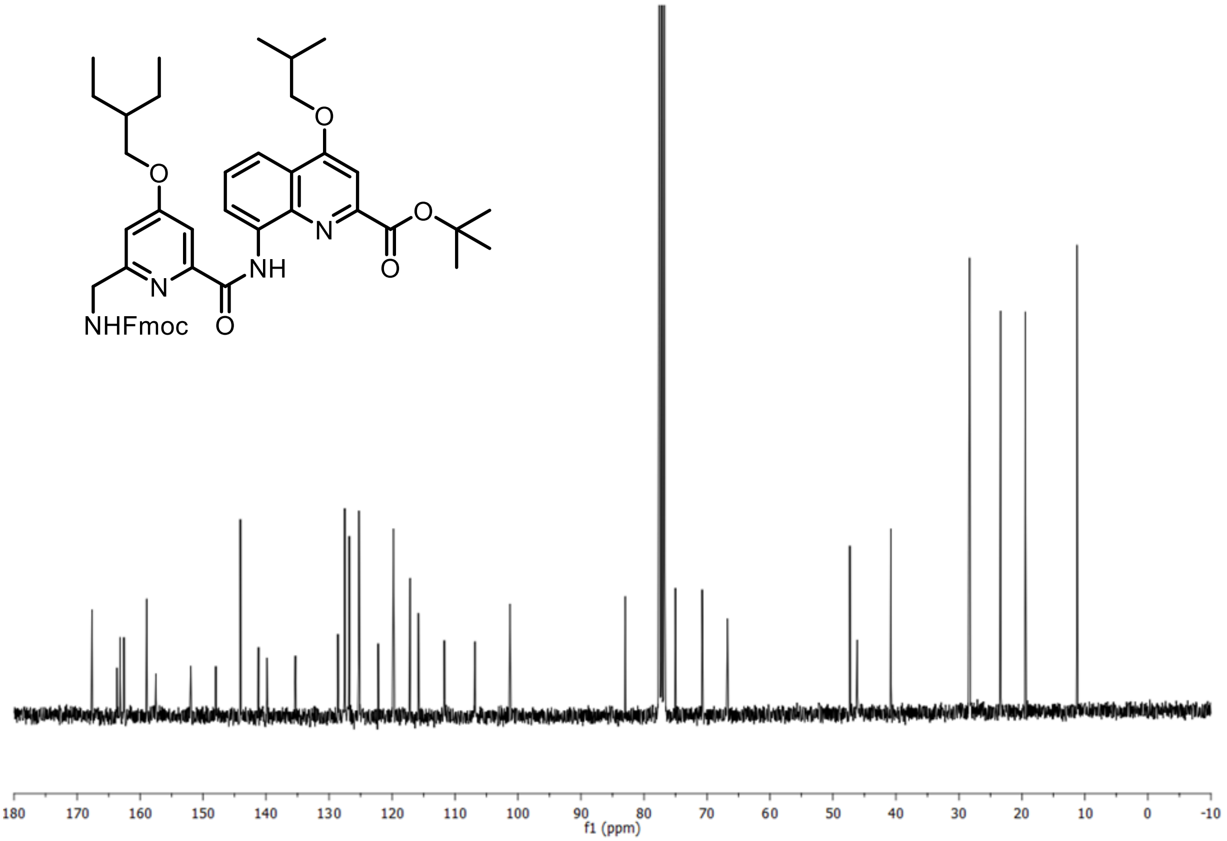

Figure S76 ^{13}C NMR spectrum (75 MHz, $CDCl_3$) of **37**.

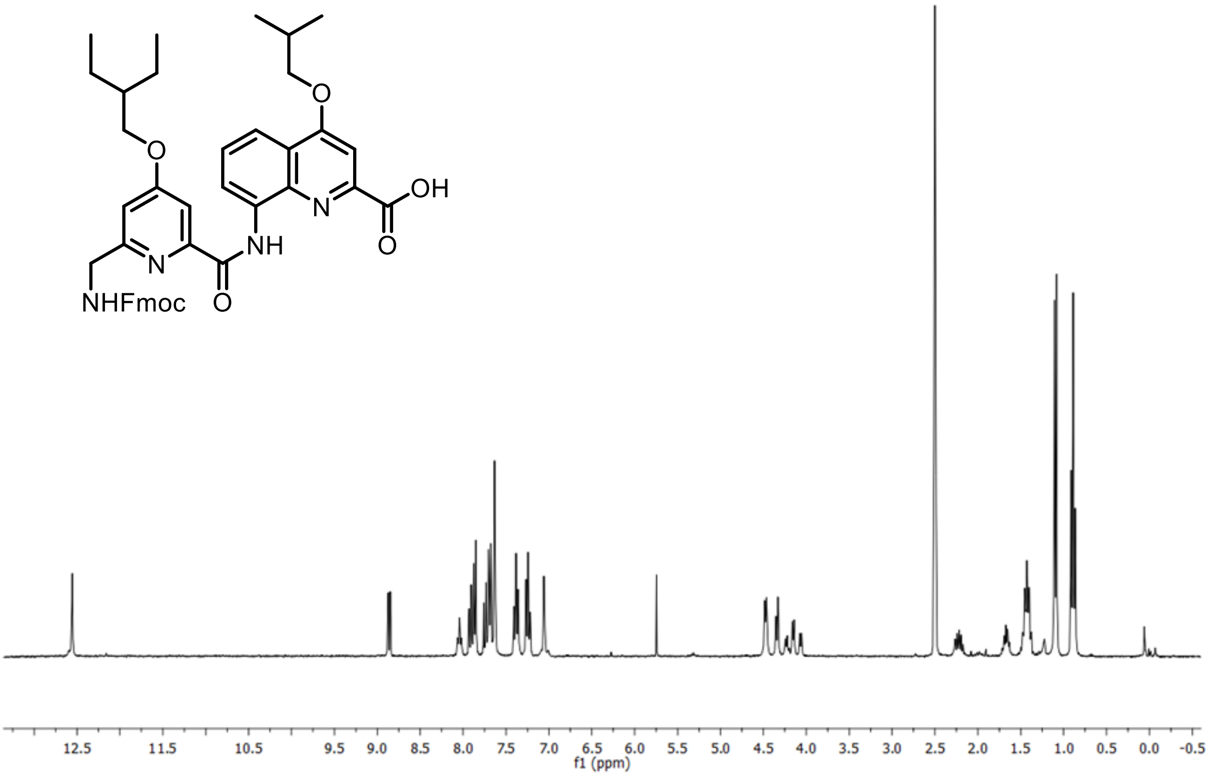

Figure S77 ^{1}H NMR spectrum (300 MHz, DMSO-d_6) of **38**.

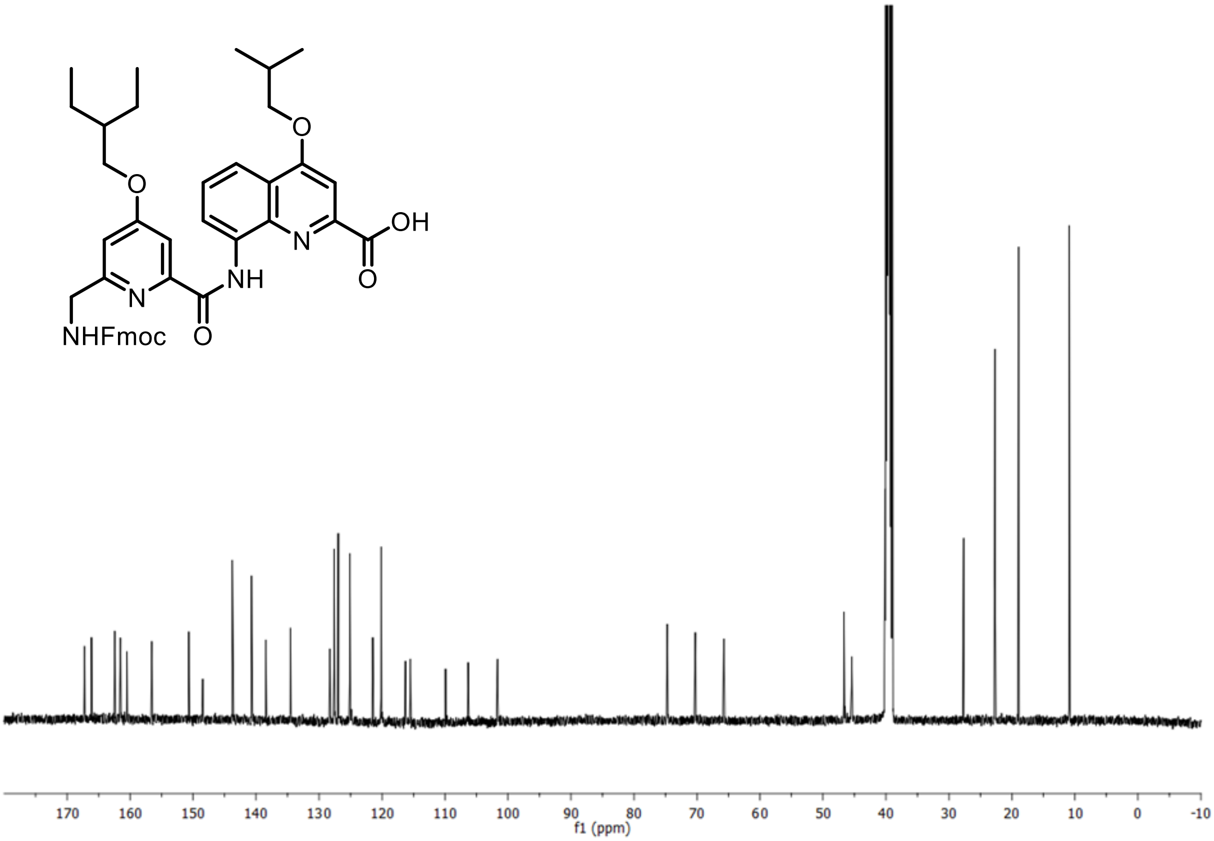

Figure S78 ^{13}C NMR spectrum (75 MHz, DMSO-d_6) of **38**.

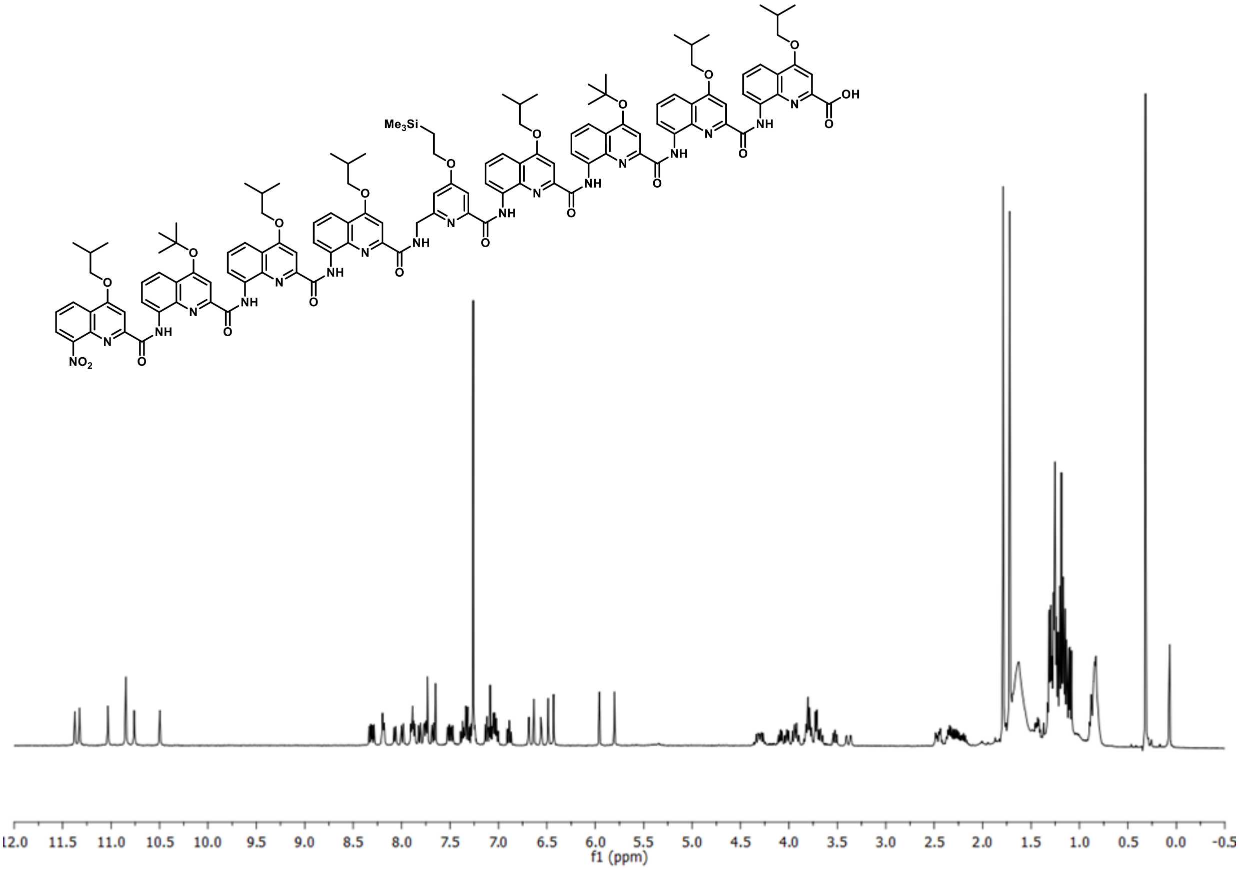

Figure S79 ^{1}H NMR spectrum (400 MHz, $CDCl_3$) of **9**.

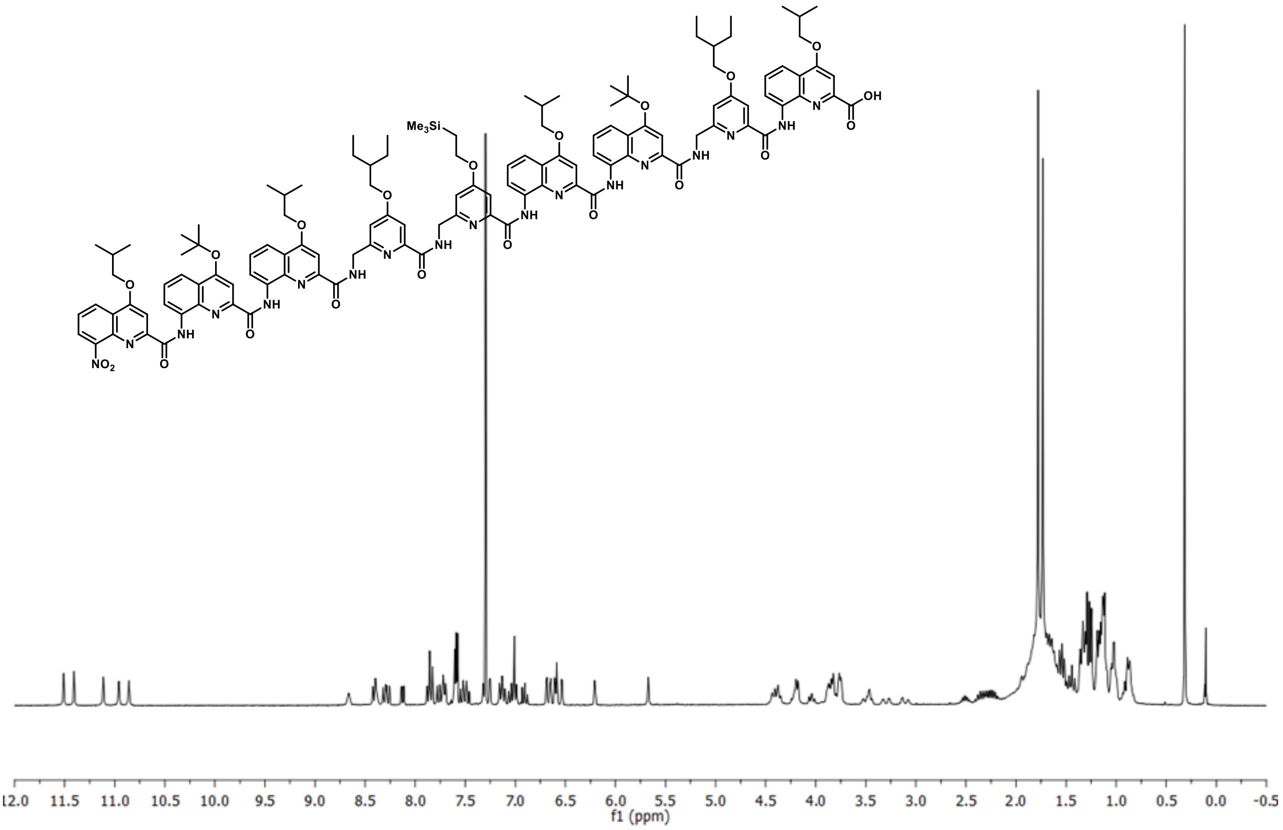

Figure S80 ^{1}H NMR spectrum (300 MHz, $CDCl_3$) of **10**.

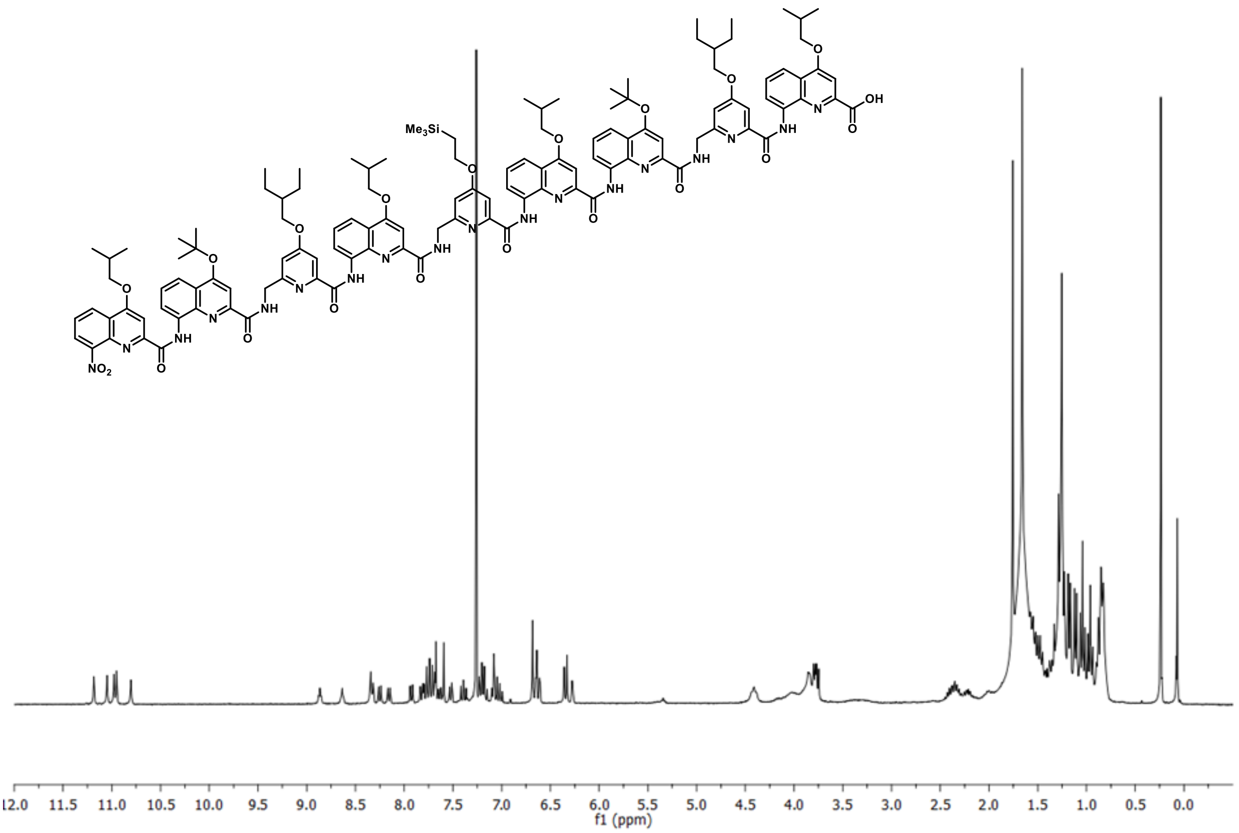

Figure S81 ^{1}H NMR spectrum (300 MHz, $CDCl_3$) of **11**.

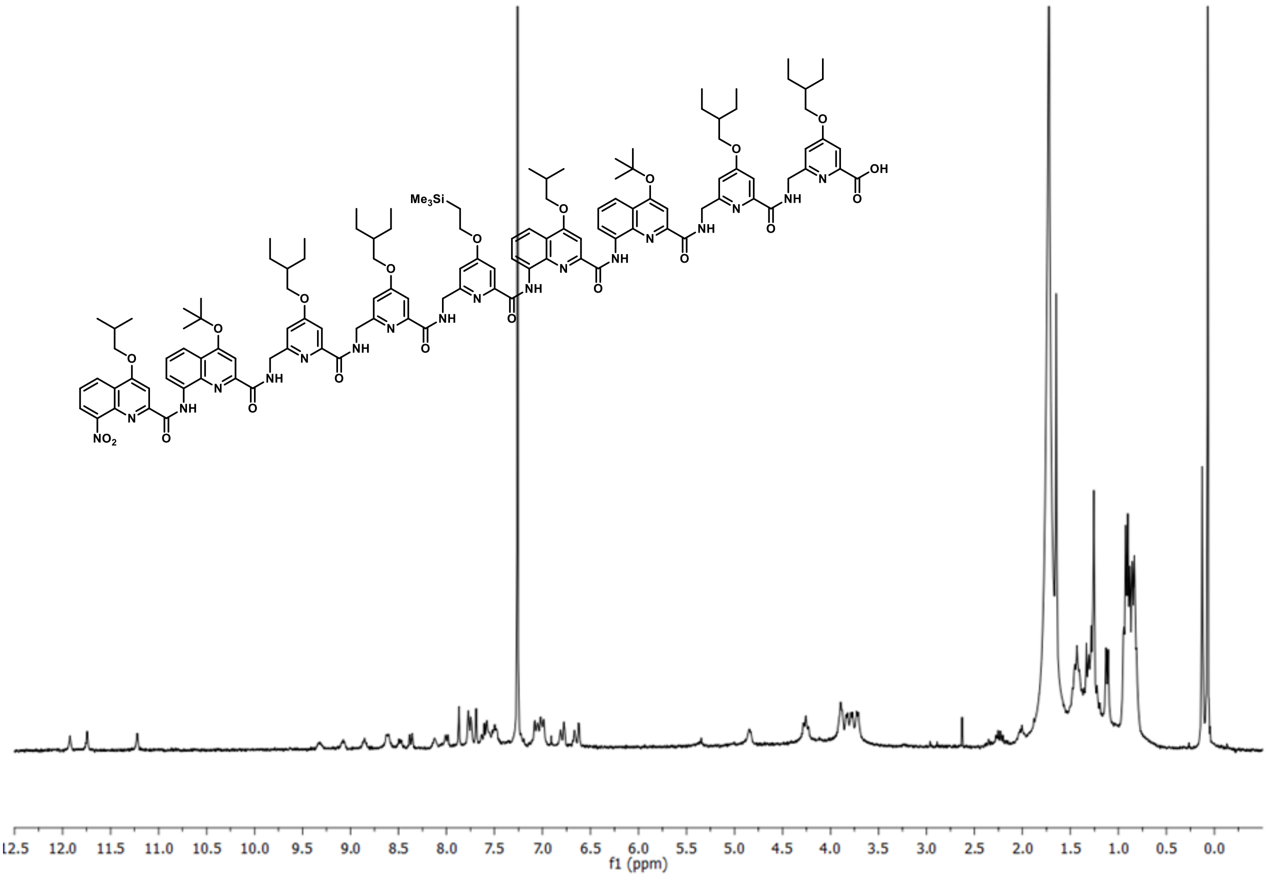

Figure S82 ^{1}H NMR spectrum (300 MHz, $CDCl_3$) of **12**.

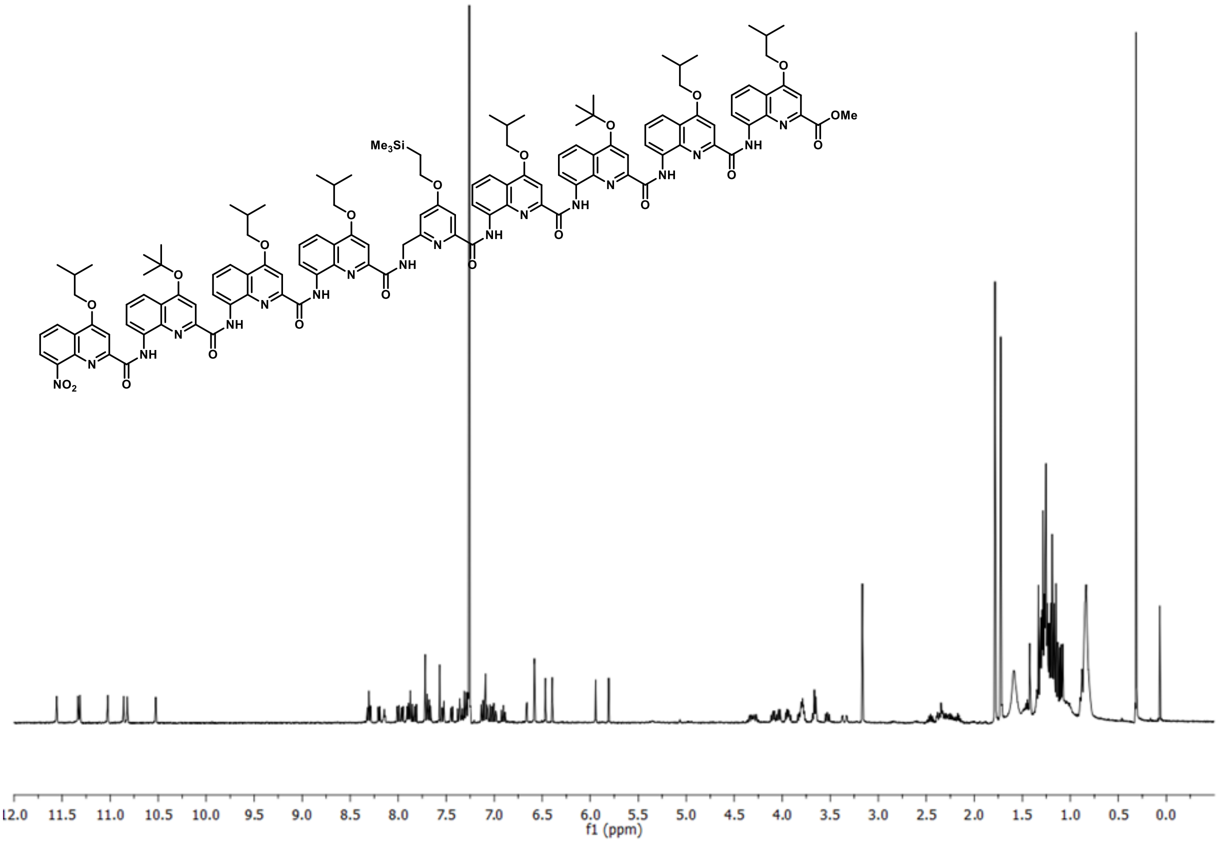

Figure S83 ^{1}H NMR spectrum (400 MHz, $CDCl_3$) of **1**.

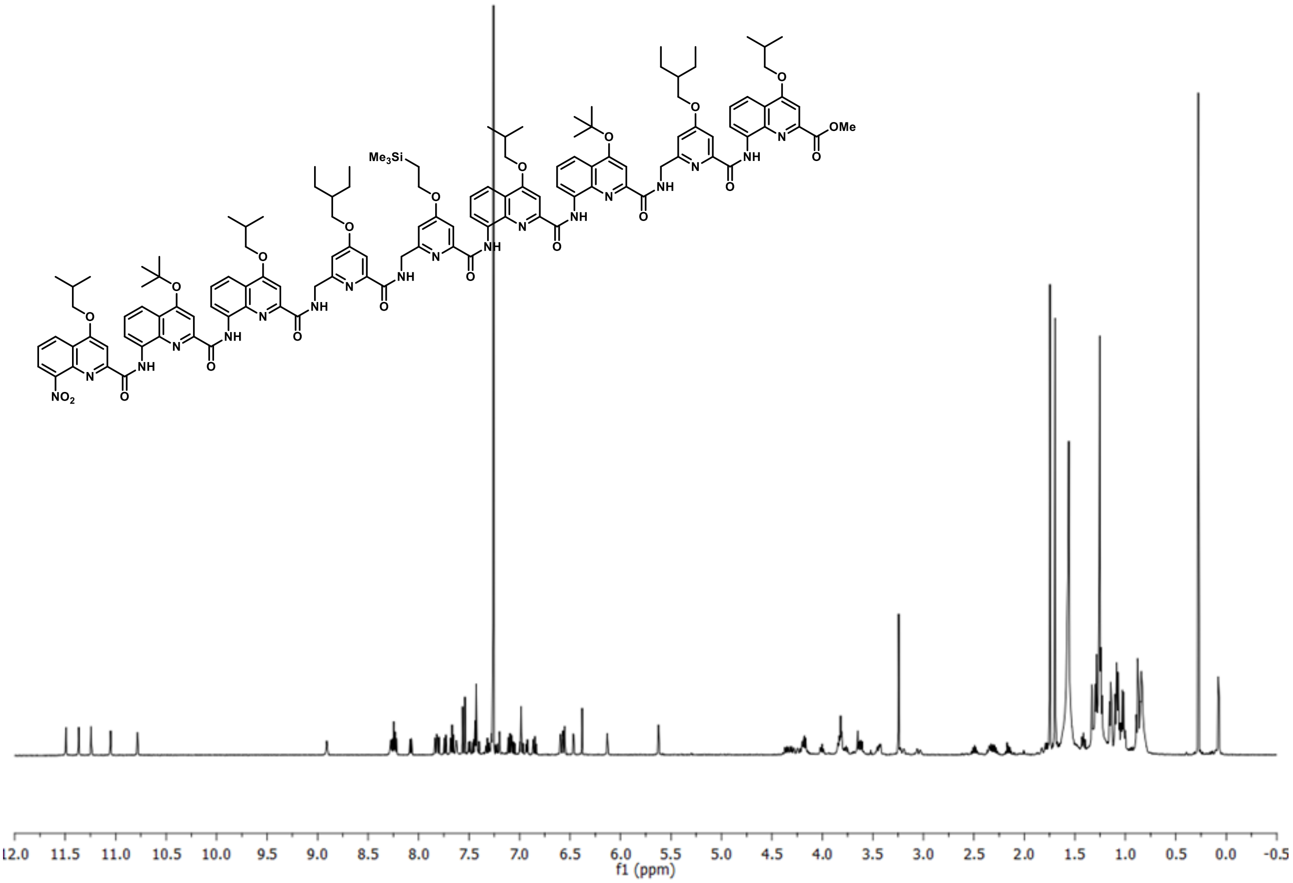

Figure S84 ^{1}H NMR spectrum (500 MHz, $CDCl_3$) of **2**.

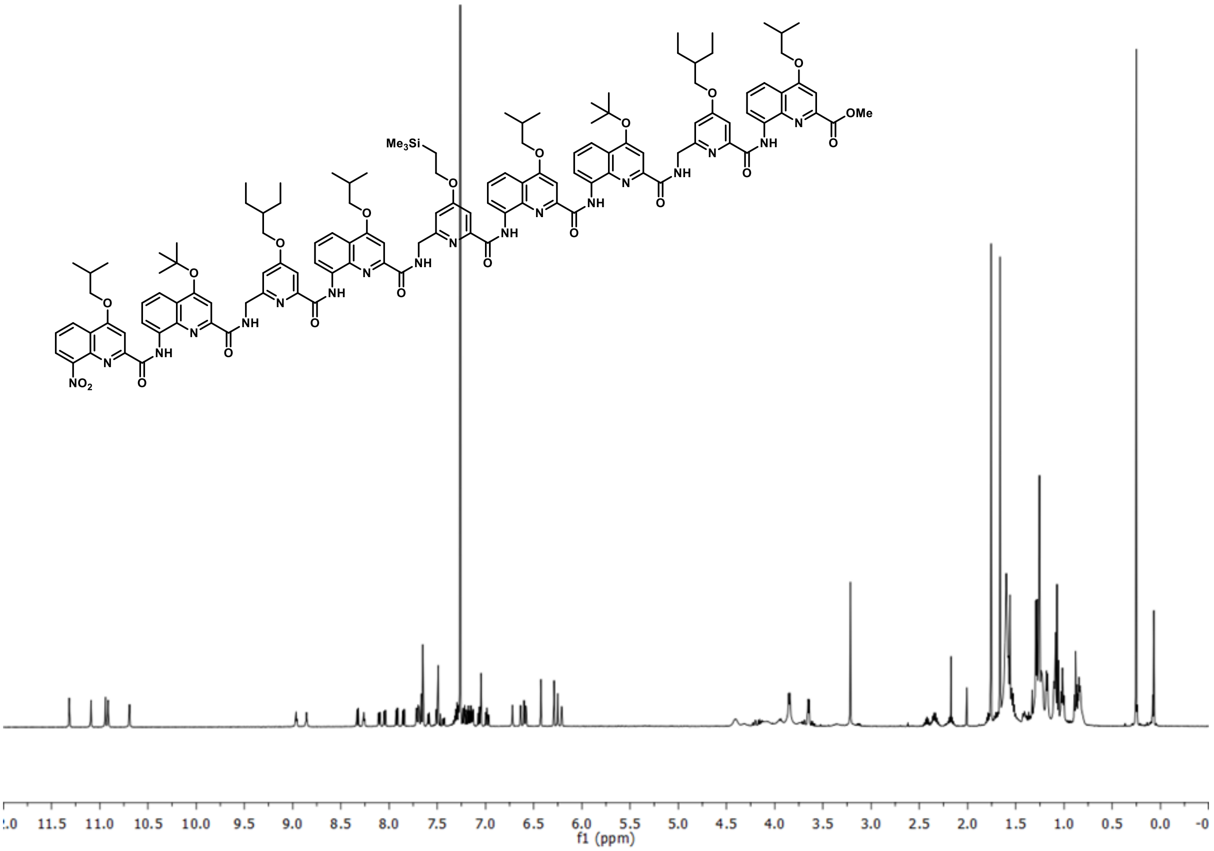

Figure S85 ^{1}H NMR spectrum (400 MHz, $CDCl_3$) of **3**.

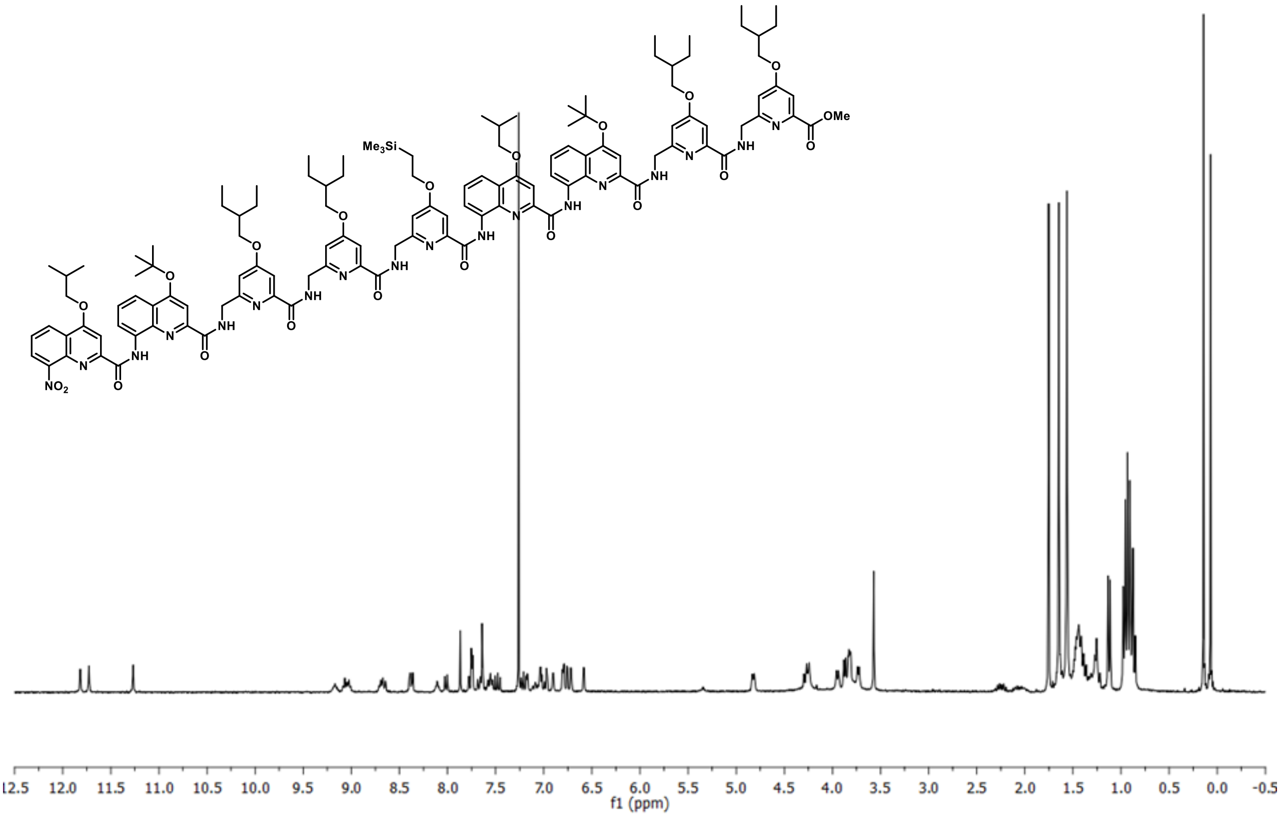

Figure S86 ^{1}H NMR spectrum (700 MHz, $CDCl_3$) of **4**.

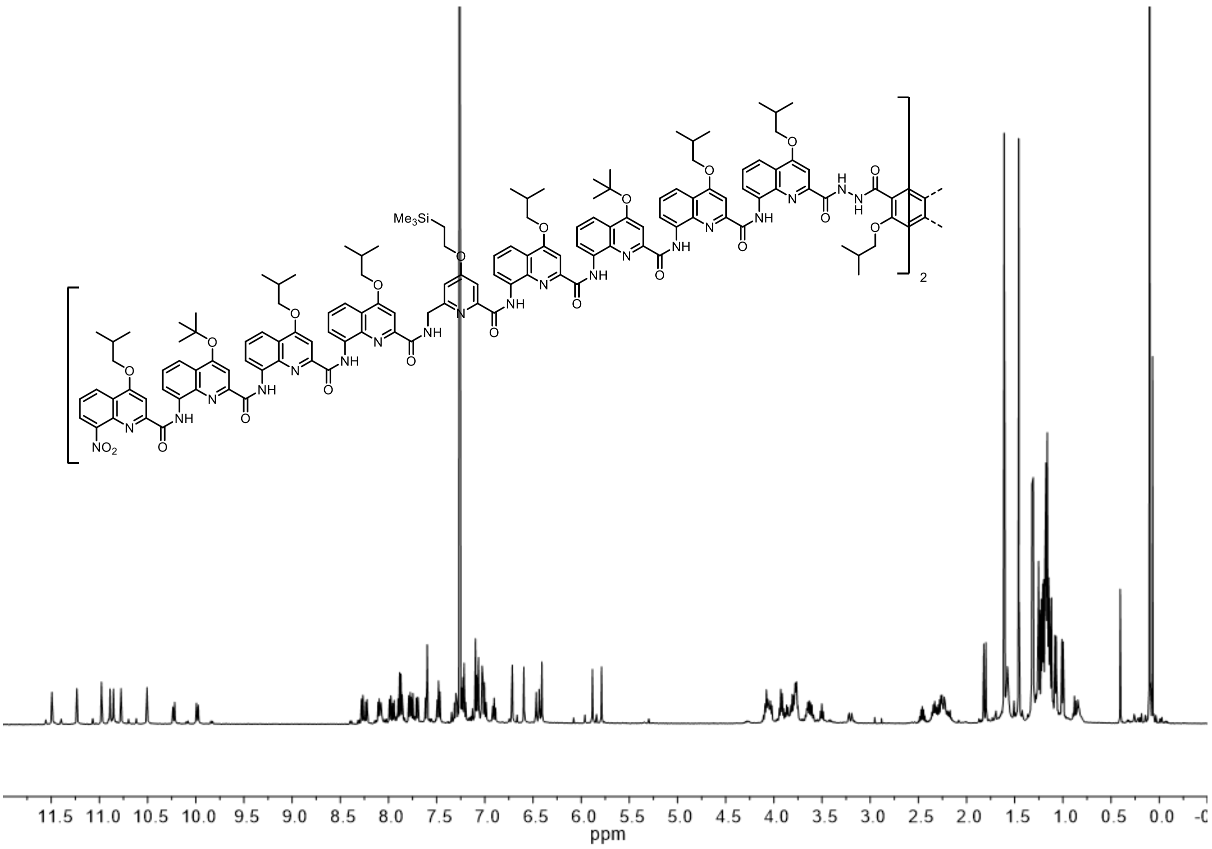

Figure S87 ^{1}H NMR spectrum (300 MHz, $CDCl_3$) of **5a**.

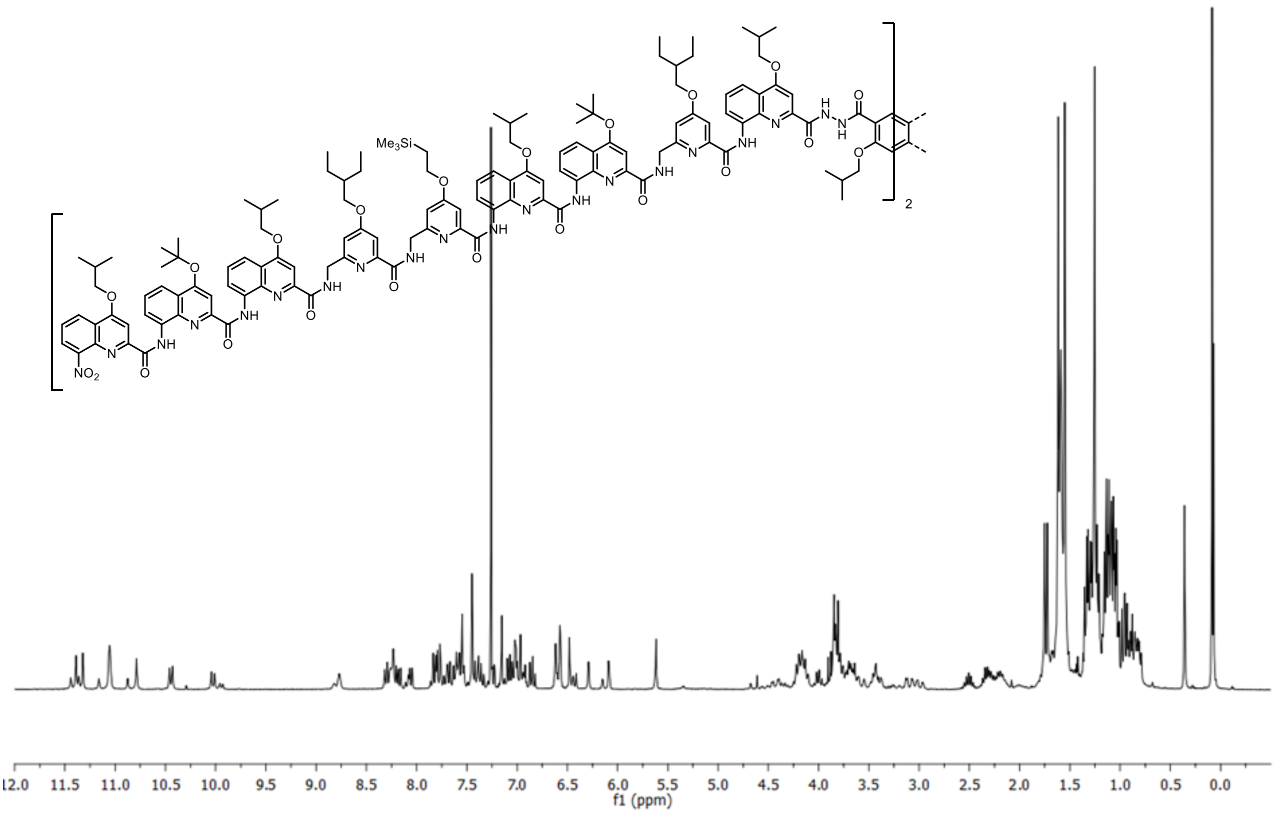

Figure S88 ^{1}H NMR spectrum (300 MHz, $CDCl_3$) of **6a**.

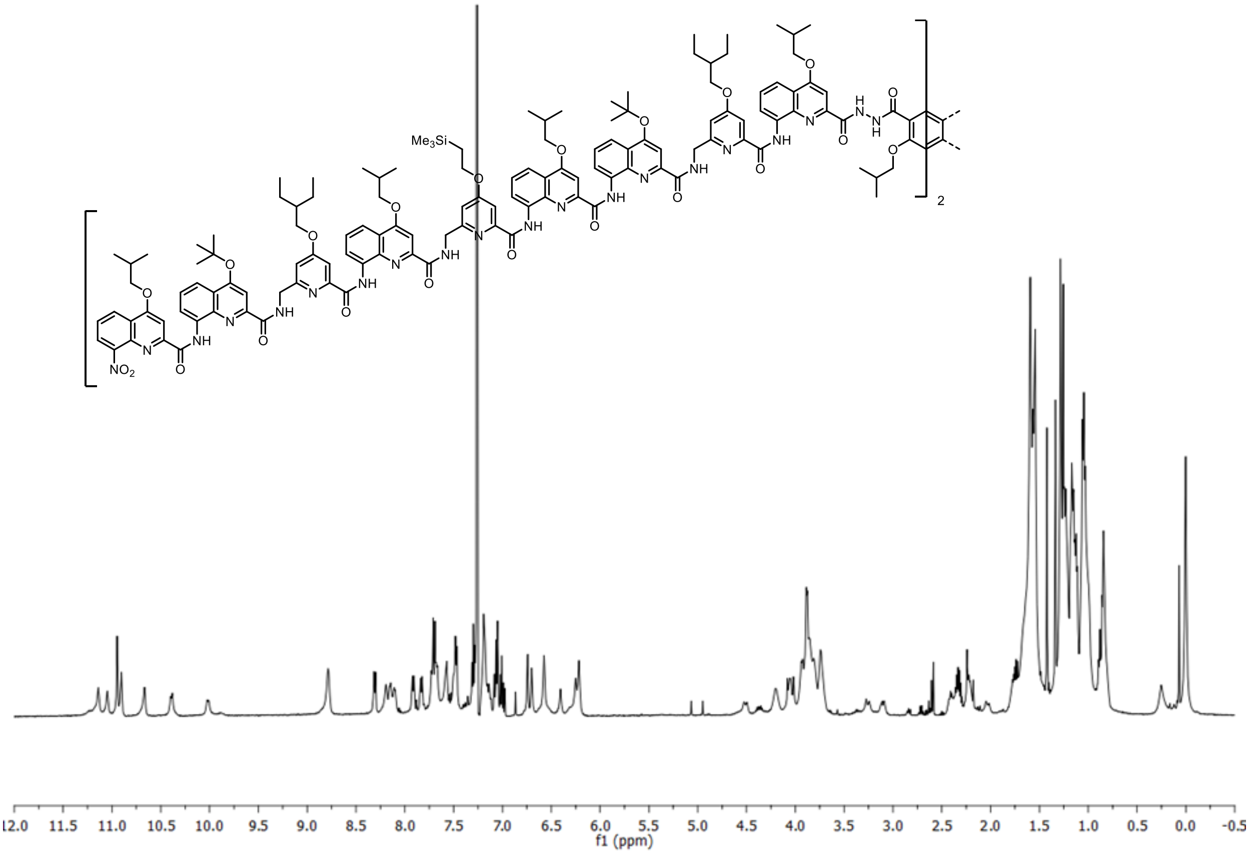

Figure S89 ^{1}H NMR spectrum (500 MHz, $CDCl_3$) of **7a**.

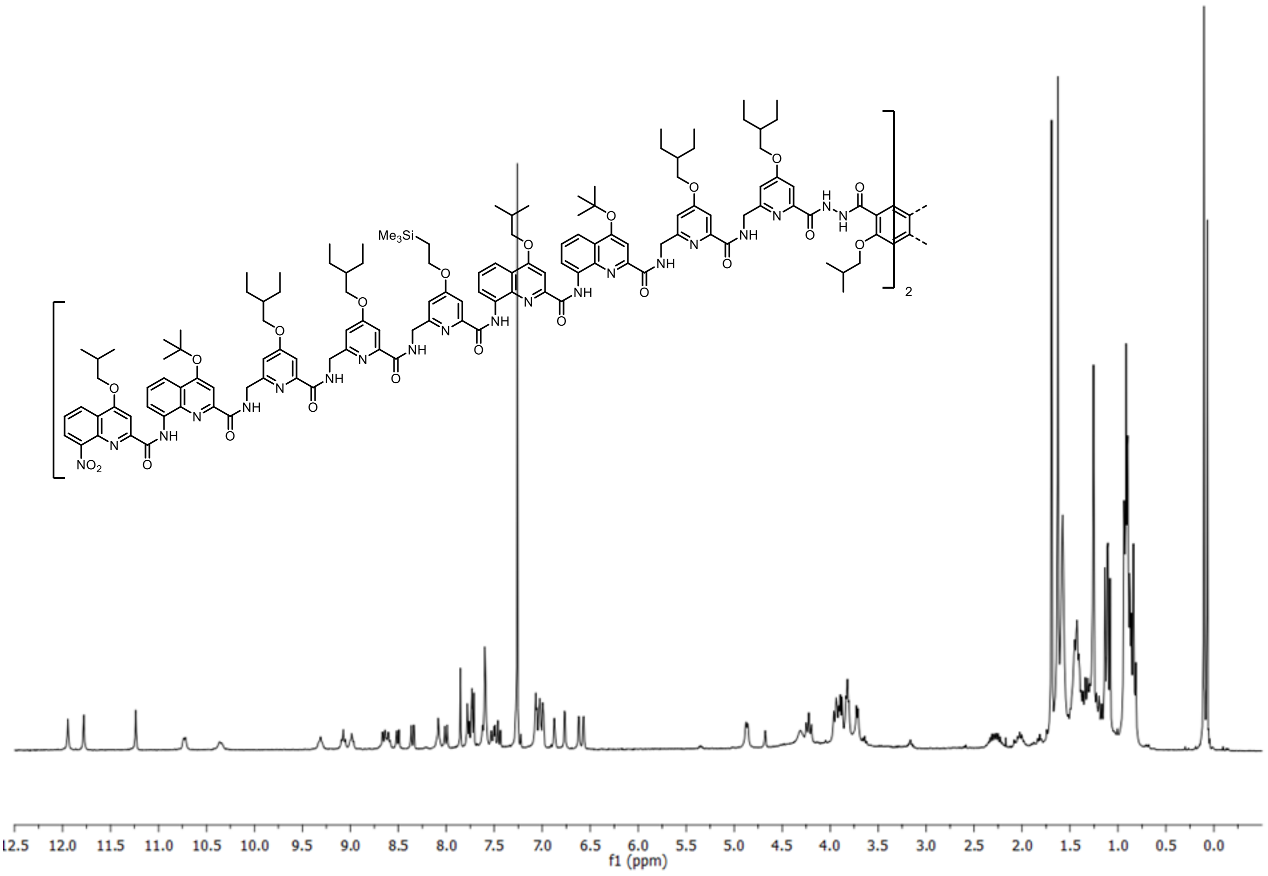

Figure S90 ^{1}H NMR spectrum (300 MHz, $CDCl_3$) of **8a**.

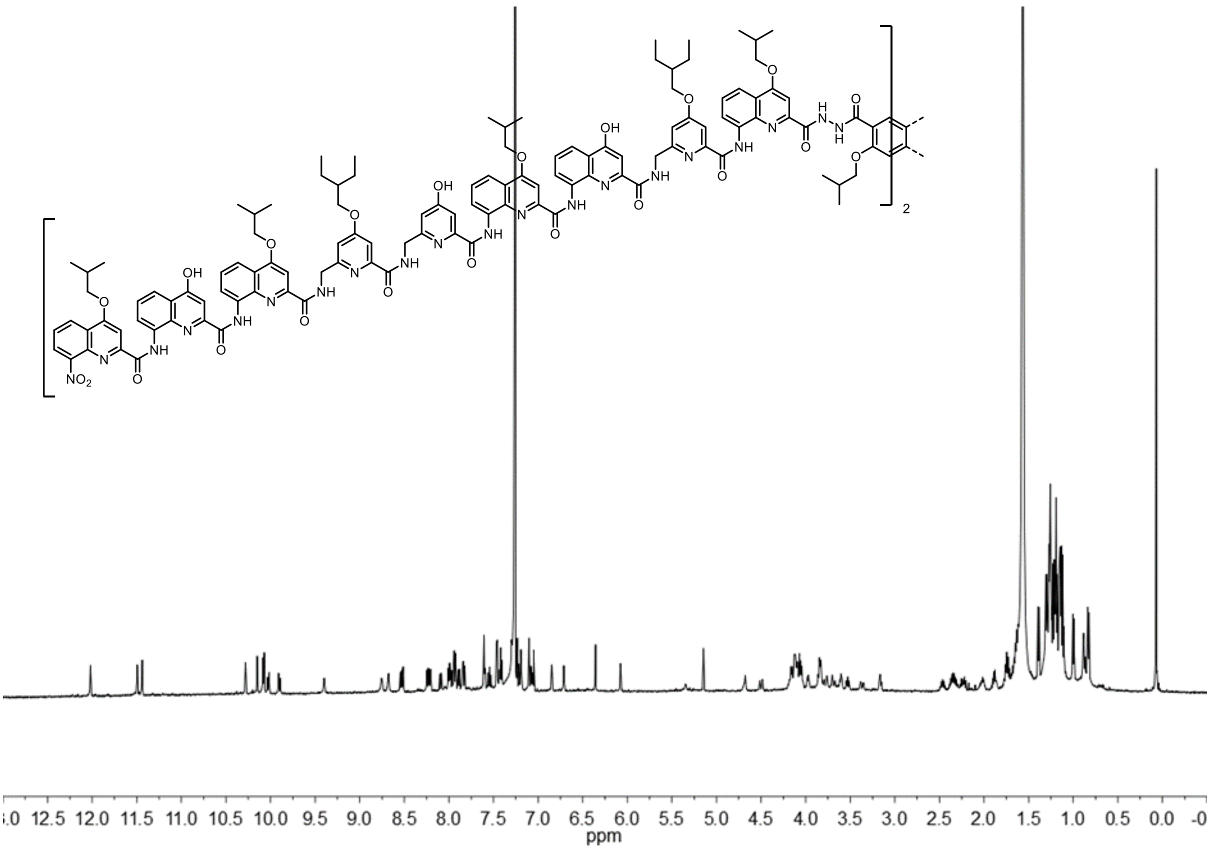

Figure S91 ^{1}H NMR spectrum (400 MHz, $CDCl_3$) of **6b**.

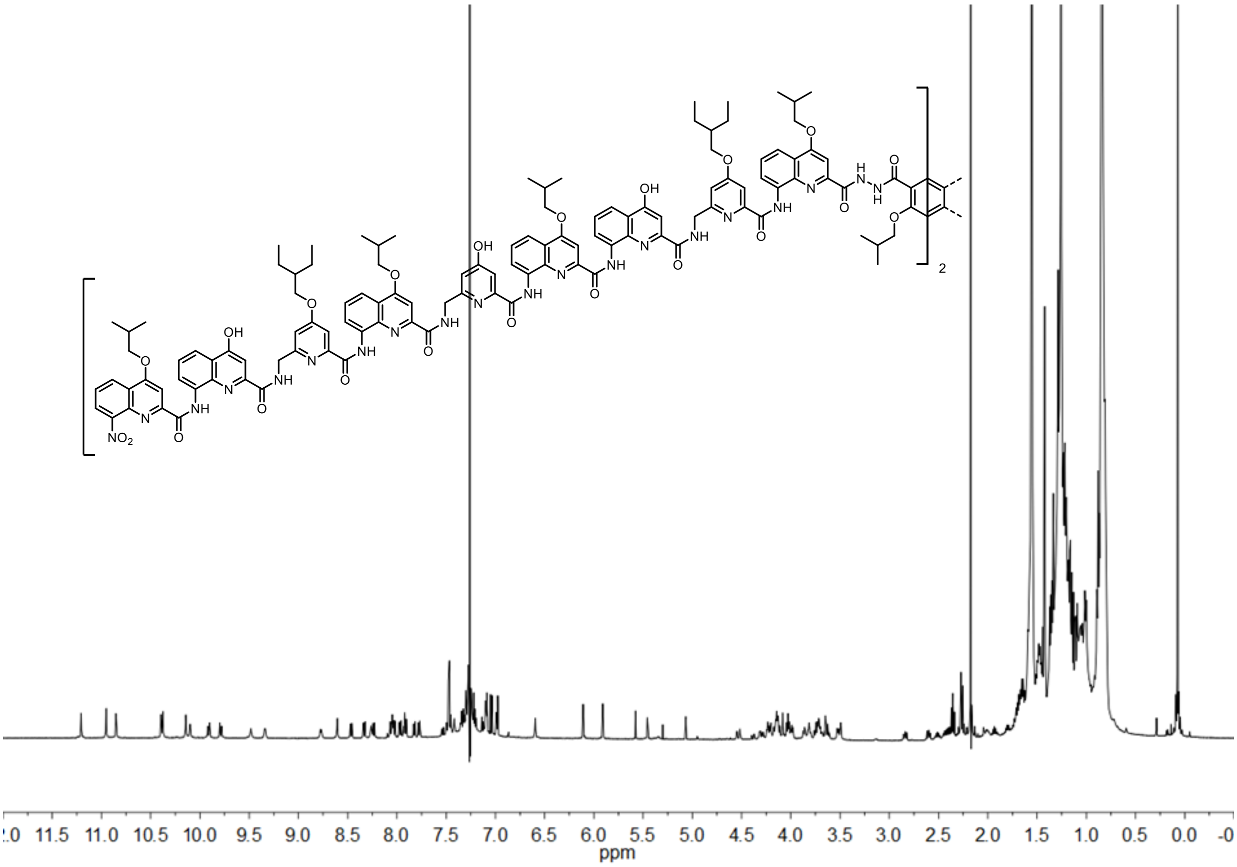

Figure S92 ^{1}H NMR spectrum (300 MHz, $CDCl_3$) of **7b**.

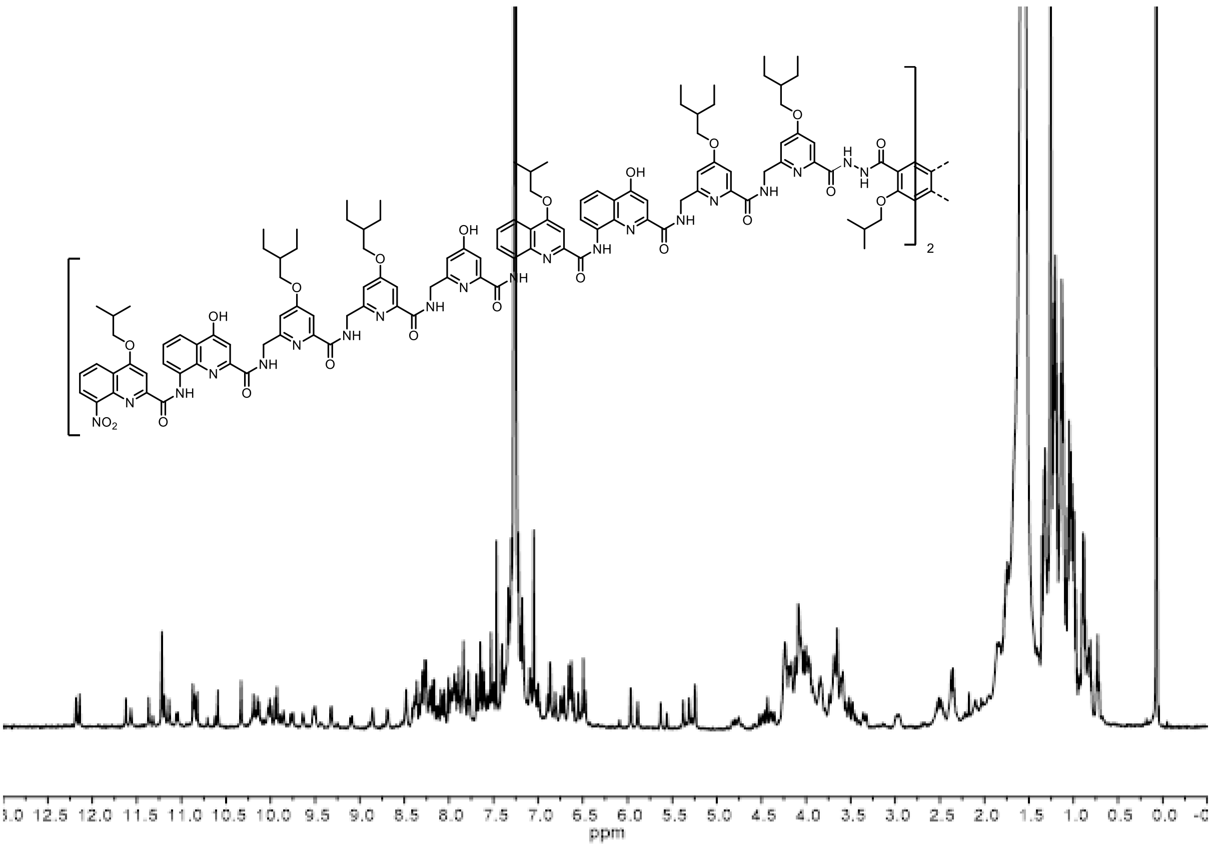

Figure S93 ^{1}H NMR spectrum (300 MHz, $CDCl_3$) of **8b**.

8. RP-HPLC analysis of oligomers

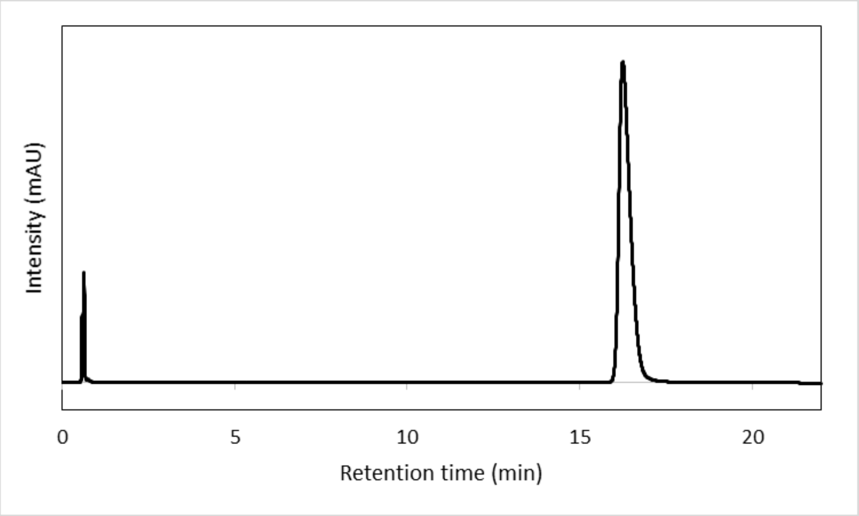

Figure S94 RP-HPLC profile of compound **1**.

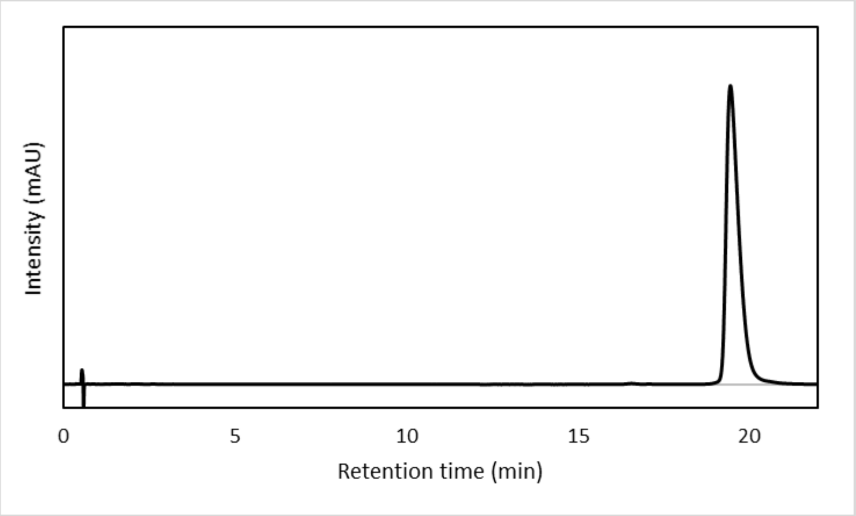

Figure S95 RP-HPLC profile of compound **2**.

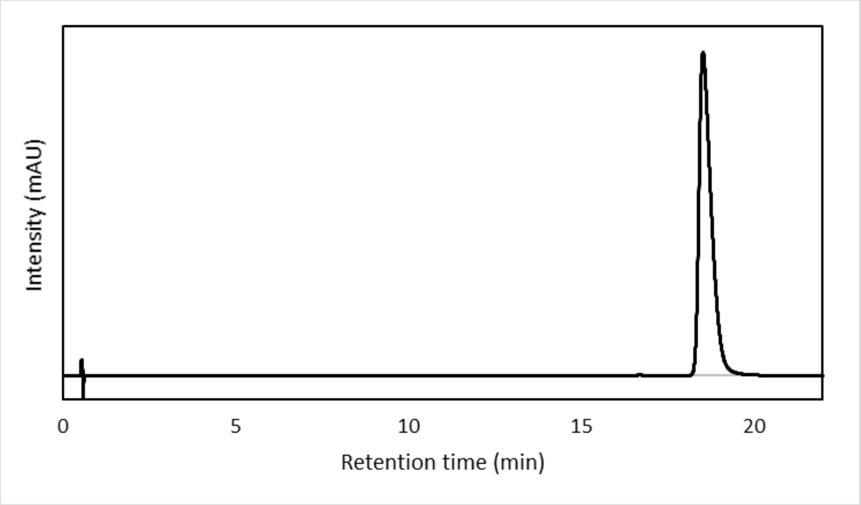

Figure S96 RP-HPLC profile of compound **3**.

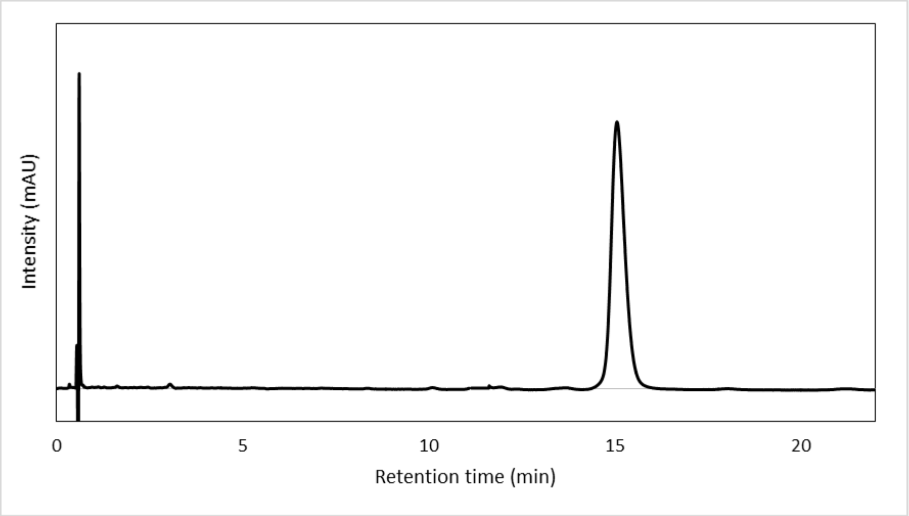

Figure S97 RP-HPLC profile of compound **4**.

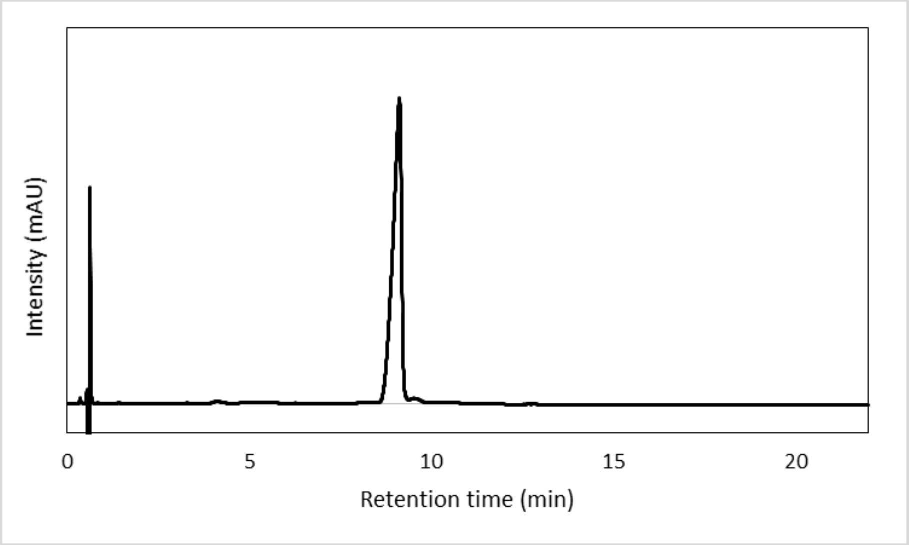

Figure S98 RP-HPLC profile of compound **9**.

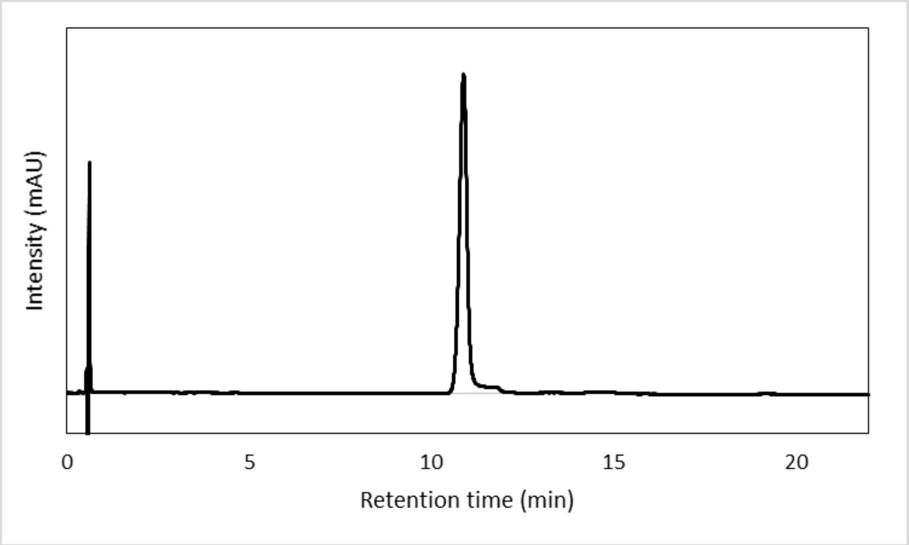

Figure S99 RP-HPLC profile of compound **10**.

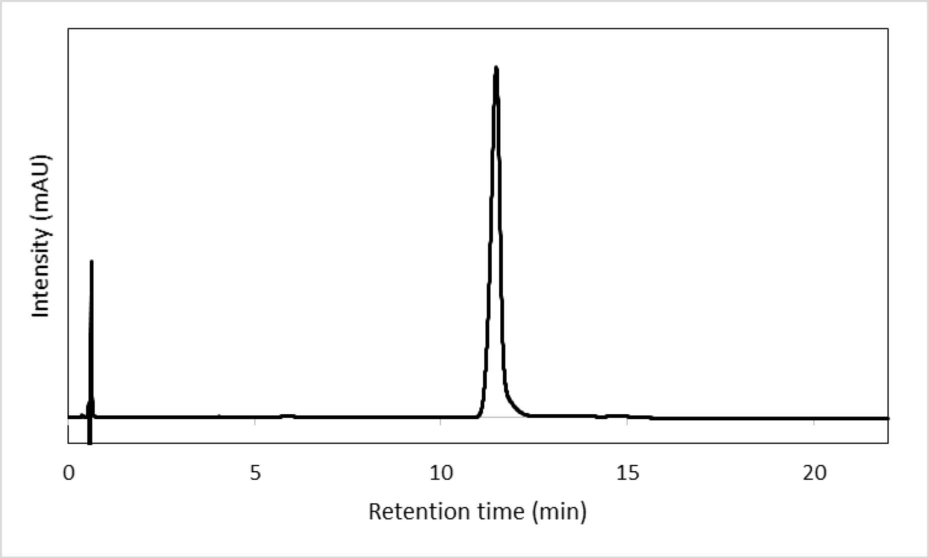

Figure S100 RP-HPLC profile of compound **11**.

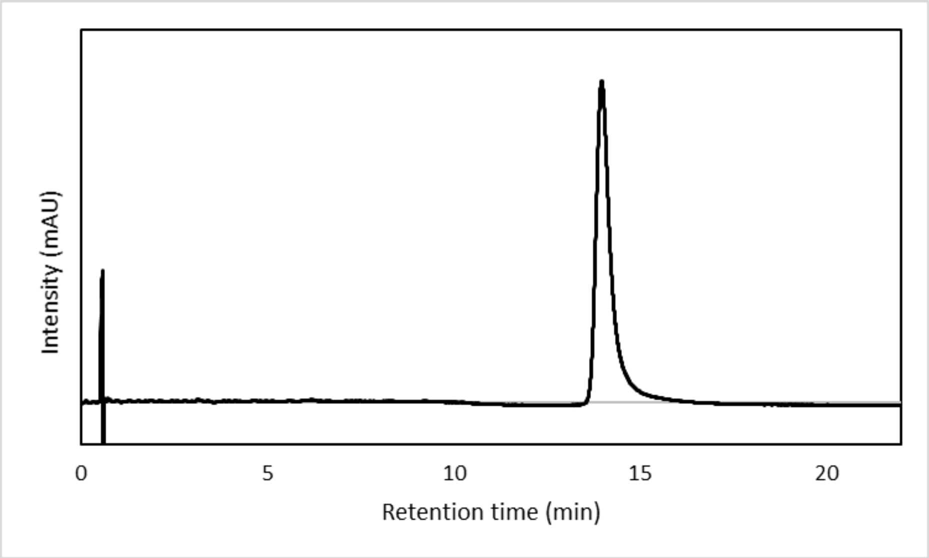

Figure S101 RP-HPLC profile of compound **12**.
